# CLDN6-specific CAR-T cells plus amplifying RNA vaccine in relapsed or refractory solid tumors: the phase 1 BNT211-01 trial

**DOI:** 10.1038/s41591-023-02612-0

**Published:** 2023-10-23

**Authors:** Andreas Mackensen, John B.A.G. Haanen, Christian Koenecke, Winfried Alsdorf, Eva Wagner-Drouet, Peter Borchmann, Daniel Heudobler, Barbara Ferstl, Sebastian Klobuch, Carsten Bokemeyer, Alexander Desuki, Florian Lüke, Nadine Kutsch, Fabian Müller, Eveline Smit, Peter Hillemanns, Panagiotis Karagiannis, Erol Wiegert, Ying He, Thang Ho, Qing Kang-Fortner, Anna Melissa Schlitter, Catrine Schulz-Eying, Andrew Finlayson, Carina Flemmig, Klaus Kühlcke, Liane Preußner, Benjamin Rengstl, Özlem Türeci, Uğur Şahin

**Affiliations:** 1 University Hospital Erlangen, Department of Internal Medicine 5, Hematology/Oncology, Erlangen, Germany; 2 Bavarian Cancer Research Center (BZKF), Erlangen, Germany; 3 Netherlands Cancer Institute, Division of Medical Oncology, Amsterdam, the Netherlands; 4 Leiden University Medical Center, Department of Oncology, Leiden, the Netherlands; 5 Hannover Medical School, Department of Hematology, Hemostasis, Oncology and Stem Cell Transplantation, Hannover, Germany; 6 University Medical Center Hamburg-Eppendorf, Department of Oncology, Hematology and Bone Marrow Transplantation with Division of Pneumology, Hamburg, Germany; 7 University Medical Center Mainz, 3rd Medical Department, Hematology and Oncology, Mainz, Germany; 8 University Hospital of Cologne, Department I of Internal Medicine and Center for Integrated Oncology Aachen Bonn Cologne Düsseldorf, Cologne, Germany; 9 University Hospital Regensburg, Department of Internal Medicine III, Hematology and Oncology, Regensburg, Germany; 10 Hannover Medical School, Department of Gynecology and Obstetrics, Hannover, Germany; 11 Bexon Clinical Consulting, Upper Montclair, NJ USA; 12 BioNTech SE, Mainz, Germany; 13 BioNTech US, Cambridge, MA USA; 14 BioNTech Innovative Manufacturing Services GmbH, Idar-Oberstein, Germany; 15 BioNTech Cell & Gene Therapies GmbH, Mainz, Germany

**Keywords:** Cancer immunotherapy, Phase I trials, Germ cell tumours, Vaccines, Drug development

## Abstract

The oncofetal antigen Claudin 6 (CLDN6) is highly and specifically expressed in many solid tumors, and could be a promising treatment target. We report dose escalation results from the ongoing phase 1/2 BNT211-01 trial evaluating the safety and feasibility of chimeric antigen receptor (CAR) T cells targeting the CLDN6 with or without a CAR-T cell-amplifying RNA vaccine (CARVac) at two dose levels (DLs) in relapsed/refractory CLDN6-positive solid tumors. The primary endpoints were safety and tolerability, maximum tolerated dose and recommended phase 2 dose (RP2D). Secondary endpoints included objective response rate (ORR) and disease control rate. We observed manageable toxicity, with 10 out of 22 patients (46%) experiencing cytokine release syndrome including one grade 3 event and 1 out of 22 (5%) with grade 1 immune effector cell-associated neurotoxicity syndrome. Dose-limiting toxicities occurred in two patients at the higher DL, resolving without sequelae. CAR-T cell engraftment was robust, and the addition of CARVac was well tolerated. The unconfirmed ORR in 21 evaluable patients was 33% (7 of 21), including one complete response. The disease control rate was 67% (14 of 21), with stable disease in seven patients. Patients with germ cell tumors treated at the higher DL exhibited the highest response rate (ORR 57% (4 of 7)). The maximum tolerated dose and RP2D were not established as the trial has been amended to utilize an automated manufacturing process. A repeat of the dose escalation is ongoing and will identify a RP2D for pivotal trials. ClinicalTrials.gov Identifier: NCT04503278.

## Abstract (web summary)

In the ongoing phase 1/2 BNT211-01 trial, CLDN6-specific chimeric antigen receptor (CAR)-T cells given with or without CARVac, a CAR-T cell-amplifying RNA vaccine, were well-tolerated and exhibited encouraging clinical activity in patients with relapsed or refractory CLDN6-positive solid tumors, with the highest response rate in patients with germ cell tumors.

## Main

Chimeric antigen receptor (CAR)-engineered T cell therapies have shown remarkable results in patients with relapsed/refractory (r/r) B-cell neoplasms and multiple myeloma^1–4^. In patients with solid cancers, however, the efficacy of CAR-T cell therapy has so far been limited.

A key obstacle for CAR-T cell approaches in solid tumors is the lack of highly cancer-specific cell-surface targets for efficient tumor eradication without on-target/off-tumor toxicity. Other common challenges are poor expansion and persistence, to which lack of antigenic stimulation due to inaccessibility of the target antigen in the periphery and an immunosuppressive tumor microenvironment may contribute^5,6^.

We have previously identified the primitive tight junction protein claudin 6 (CLDN6) as a potentially ideal immunotherapy target^7–9^. The gene encoding this oncofetal cell-surface antigen is fully silenced upon completion of fetal organogenesis, with expression strictly suppressed in healthy adult tissues; however, frequent aberrant cell-surface expression occurs in various solid tumor indications^10–14^. High-level CLDN6 expression is commonly detectable in germ cell tumors (GCT), epithelial ovarian cancer (EOC), endometrial carcinoma and additional solid tumor indications, including rare malignancies.

We have designed a highly sensitive, strictly CLDN6-specific second generation CAR with fusion of a CLDN6-specific single-chain variable fragment, the CD8α hinge and transmembrane region and 4-1BB and CD3ζ signaling moieties, which mediates rapid rejection of CLDN6-positive tumors by engineered T cells in xenograft and syngeneic mouse models^10^. We developed a nanoparticulate CLDN6-expressing CAR-T cell-amplifying RNA vaccine (CARVac), which contains uridine nucleoside messenger RNA (mRNA) encoding the target antigen recognized by the CAR-T cells. The RNA is formulated in lipids to form RNA-lipoplexes (RNA-LPX)^15,16^. Administration of the vaccine facilitates body-wide RNA delivery to antigen presenting cells (APCs) in lymphoid organs. In multiple preclinical models, uptake and translation of the natively folded CAR target protein by APCs mediates in vivo stimulation and controlled expansion of CAR-T cells, induces a memory T cell phenotype, increases target sensitivity and enables tumor control at subtherapeutic CAR-T cell doses^10^.

Based on this work, we initiated a phase 1/2 first-in-human clinical trial to evaluate CLDN6 CAR-T cells alone and in combination with CARVac in patients with relapsed or refractory (r/r) CLDN6-positive solid tumors in a dose escalation part followed by cancer type-specific dose expansion cohorts. The manufacturing process of the CAR-T product is being developed in parallel to its clinical testing to implement a mature process with favorable product characteristics to facilitate a transition from manual to automated manufacturing before starting potentially pivotal trials.

Here we present a non-prespecified interim analysis of 22 patients treated at two dose levels in the phase 1 dose escalation part of this trial, comprising the complete set of data obtained with the manual version of the manufacturing process. These data provide insights into the feasibility, safety and antitumor activity of the first-in-human concept of targeting the CLDN6 with a CAR-T therapy and combining with a CAR-amplifying vaccine.

## Results

### Study design and execution, patient selection and treatment

The study design comprises a dose escalation part and a dose expansion part with indication-specific cohorts including GCT, EOC and other indications to start when the recommended phase 2 dose (RP2D) obtained with the dedicated phase 2 manufacturing process is defined.

The primary endpoints for the dose escalation part are to characterize the safety and tolerability of CLDN6 CAR-T cells ± CARVac and to identify the maximum tolerated dose (MTD)/RP2D. Evaluation of the antitumor activity of CLDN6 CAR-T cells ± CARVac per Response Evaluation Criteria In Solid Tumors (RECIST) 1.1 and characterization of soluble immune factors (cytokines) induced by treatment are the secondary endpoints. An MTD/RP2D was not established, as dose escalation is being repeated with CAR-T cells manufactured with an automated manufacturing process.

The dose expansion part of the study enrolled adults diagnosed with advanced metastatic solid tumors of any type lacking further systemic treatment options, Eastern Cooperative Oncology Group (ECOG) 0–1 and measurable disease per RECIST 1.1. Patients with new or growing brain or spinal metastases during screening were excluded. Patients for the study were recruited from a CLDN6 prescreening. Additional eligibility criteria are noted in the Methods.

**Fig. 1:**
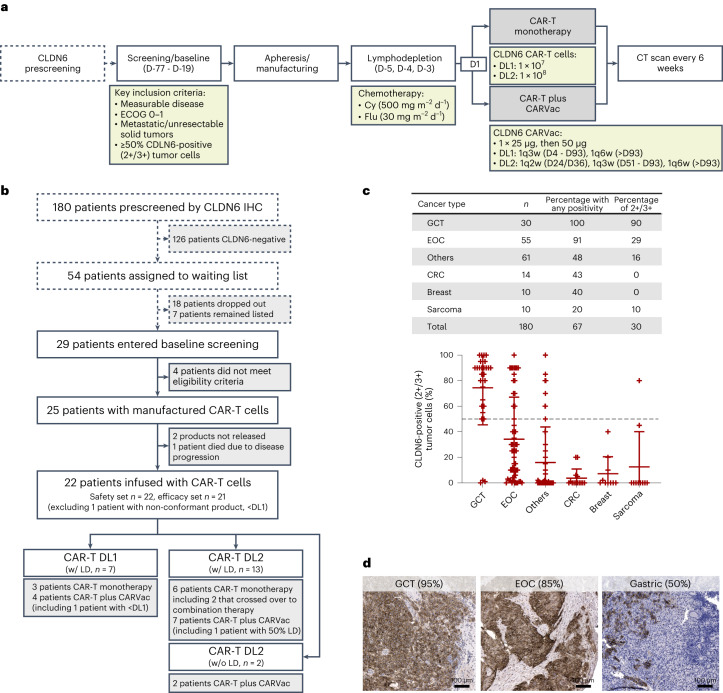
Trial design and enrollment. **a**, Phase 1 bifurcated 3 + 3 dose escalation design. Screened patients were enrolled into either CLDN6 CAR-T monotherapy or a combination with CARVac, receiving a single dose of either 1 × 10^7^ (DL1) or 1 × 10^8^ (DL2) CLDN6 CAR-T cells. CARVac was administered with a starting dose at 25 µg, followed by 50 µg doses if tolerated. **b**, As of 5 November 2021 (enrollment of last patient dosed), 180 patients were prescreened and 54 classified as CLDN6-positive (50% of tumor cells ≥2+ CLDN6 membrane staining). Eighteen patients dropped out before the screening visit due to death (*n* = 9), worsening of condition (*n* = 7), loss to follow-up (*n* = 1) or refusal of participation (*n* = 1). Twenty-nine patients underwent full screening for trial eligibility, of which four patients did not meet eligibility criteria. Seven patients remained listed to be screened for trial eligibility, but did not consent for full trial eligibility screening, for example, because of decision to undergo other treatment approaches. CAR-T cell products were manufactured for all 25 enrolled patients. Of those, 22 were treated and included in the safety set, while one patient, who received CAR-T cells at <DL1, was excluded from the efficacy set. **c**, CLDN6 prescreening by semiquantitative immunohistochemistry assay, showing individual data points with mean ± standard deviation. Frequency of patients with CLDN6 2+/3+ expression with mean ± s.e.m. (black dashed line represents cutoff). Indications with <10 cases are summarized as ‘others’. **d**, Examples of CLDN6-positive cases treated within the trial. Thirteen GCT, four EOC and one each of DSRCT, gastric adenocarcinoma, serous carcinoma of the fallopian tube, endometrial carcinoma and CUP have been treated within the study (Table 1 and Supplementary Fig. 1). CRC, colorectal cancer; LD, lymphodepleting chemotherapy; w/, with; w/o, without.

Of 180 patients prescreened for CLDN6 expression with a semiquantitative immunohistochemistry assay between September 2020 and November 2021, 54 (30%) met the inclusion criteria for tumor CLDN6 positivity defined as ≥50% of tumor cells displaying an intermediate (2+) or strong (3+) membrane staining intensity (Fig. 1a,b and Supplementary Fig. 1). The fraction of patients complying with the CLDN6-positivity threshold was highest for GCT (90%) and EOC (29%) (Fig. 1c). No significant differences in staining intensity were observed according to whether the specimens were obtained recently or were older paraffin-embedded tissue specimens; this was consistent for both primary and metastatic lesions (Supplementary Fig. 2).

Following successful prescreening for fulfillment of CLDN6 expression, twenty-nine patients entered screening for the other enrollment criteria of this trial. We manufactured autologous CLDN6 CAR-T cells for 25 patients who met all eligibility criteria, of which 22 were patients treated with CLDN6 CAR-T cells manufactured from autologous leukapheresis material collected between 15 December 2020 and 14 February 2022, with the day of leukapheresis defined as the day of enrollment to this trial (Fig. 1b). The drug products contained both CD4^+^ and CD8^+^ T cells, with the CD4^+^ subset dominating (Supplementary Fig. 3a). Overall, the proportion of naïve (T_N_) and effector memory phenotype (T_EM_) was similar within the CD4^+^ T cell population. (Supplementary Fig. 3b). CD8^+^ T cells had a predominantly T_N_-like phenotype, followed by an effector memory re-expressing CD45RA (T_EMRA_) phenotype (Supplementary Fig. 3c).

Dose escalation followed a 3 + 3 approach with patients receiving CLDN6 CAR-T cells at DL1 (1 × 10^7^) and DL2 (1 × 10^8^) ± CARVac. Of the 22 treated patients, 13 had GCTs (all testicular cancer with non-seminoma or mixed-type histology), four had EOCs (all serous carcinoma) and one patient each had endometrial carcinoma, serous carcinoma of the fallopian tube, desmoplastic small round cell tumor (DSRCT), gastric adenocarcinoma and cancer of unknown primary (CUP) (Table 1). GCT and EOC patients had the strongest CLDN6 expression, with an average of >80% tumor cells with 2+/3+ (intermediate/strong) staining intensity (Fig. 1d and Supplementary Fig. 1).

**Table 1:** Baseline characteristics

Baseline characteristics	DL1 (w/ LD, *n* = 3)	DL1 + CARVac (w/ LD, *n* = 4)	DL2 (w/ LD, *n* = 6)	DL2 + CARVac (w/ LD, *n* = 7)	DL2 + CARVac (w/o LD, *n* = 2)	Total (*n* = 22)
Median (range) age, years	33 (25–68)	46 (27–56)	56 (35–66)	42 (28–61)	51 (46–56)	46 (25–68)
Sex (male/female)	2/1	3/1	3/3	5/2	2/0	15/7
ECOG, *n* (%)						
0	1 (33)	1 (25)	2 (33)	5 (71)	1 (50)	10 (46)
1	2 (67)	3 (75)	4 (67)	2 (29)	1 (50)	12 (54)
Cancer type, *n*						
GCT	1	3	2	5	2	13^a^
EOC	1	0	1	2	0	4^b^
Endometrial cancer	0	0	1	0	0	1
Serous fallopian tube cancer	0	0	1	0	0	1
Sarcoma (DSRCT)	1	0	0	0	0	1
Gastric cancer	0	0	1	0	0	1
CUP	0	1	0	0	0	1^c^
Median (range) CLDN6 2+/3+ cells, %	60 (60–80)	90 (80–95)	82.5 (50–90)	90 (50–100)	67.5 (50–85)	85 (50–100)
Median (range) tumor sum at SCR, mm	28 (12–71)	63 (41–105)	47 (23–100)	49 (30–64)	22 (20–25)	49 (12–105)
Change in target sum until ACT						
Progressed, *n* (%)	1 (33)	3 (75)	2 (33)	1 (14)	0	7 (32)
Stable, *n* (%)	0	0	0	1 (14)	0	1 (5)
Reduced, *n* (%)	0	0	2 (33)	1 (14)	1 (50)	4 (18)
Unknown, *n* (%)	2 (66)	1 (25)	2 (33)	4 (57)	1 (50)	10 (45)
Patients with bridging CTX, *n* (%)	0	2 (50)	3 (50)	2 (29)	1 (50)	8 (36)
Disease location						
Lung involvement, *n* (%)	2 (66)	4 (100)	2 (33)	1 (14)	1 (50)	10 (45)
Liver involvement, *n* (%)	1 (33)	0	4 (66)	1 (14)	0	6 (27)
Peritoneal disease, *n* (%)	2 (66)	0	1 (17)	2 (29)	1 (50)	7 (32)
LN involvement, *n* (%)	1 (33)	3 (75)	5 (66)	3 (43)	2 (50)	13 (59)
Nodal disease, *n* (%)	0	0	0	1 (14)	1 (50)	2 (9)
Median (range) of prior treatment lines	3 (3–5)	3 (3–5)	4.5 (2–8)	5 (3–9)	3.5 (3–4)	4 (2–9)
Patients with history of:						
Platin-based treatment lines, *n*	3	4	6	7	2	22
HDCT/ASCT, *n*	1	3	2	5	2	13
RTX, *n*	1	1	2	1	0	5
CPI, *n*	0	1	1	1	0	3

Change in target sum between tumor assessment at screening and ACT was evaluable for 12 of 22 patients.

ASCT, autologous stem cell transplantation; Cy, cyclophosphamide; CPI, checkpoint inhibitor; CTX, chemotherapy; Flu, fludarabine; LN, lymph node; RTX, radiotherapy; SCR, screening (baseline).

^a^All GCT patients had testicular cancer with non-seminoma or mixed-type histology.

^b^All EOC patients had serous adenocarcinoma, three confirmed high-grade, one undetermined.

^c^The patient with a cancer of unknown primary has a non-small cell cancer suspected to be triple-negative breast cancer.

All patients (median age of 46 years) were r/r after standard of care treatment and were heavily pretreated with a median of four previous lines of treatment, predominantly platin-based chemotherapy and three had received checkpoint inhibitor therapy. All GCT patients had received high-dose chemotherapy (HDCT) plus autologous stem cell support. The four EOC patients were platinum-refractory. All patients had measurable disease, and eight patients required bridging chemotherapy between leukapheresis and CLDN6 CAR-T cell transfer. Almost half of the patients had lung metastases (including seven of the 13 GCT patients) and about 30% had either liver involvement or peritoneal carcinosis (including three of the four EOC patients). Seven of the 12 patients with two pretreatment computed tomography (CT) scans showed rapidly progressing disease from baseline visit until infusion of the CAR-T cells (5.9 weeks on average; Table 1). Those who did not progress had all been treated with bridging chemotherapy, further underlying the advanced disease status of patients recruited to this trial.

Of the 22 patients (Fig. 1b), 20 received LD (500 mg m^−^^2^ cyclophosphamide plus 30 mg m^−^^2^ fludarabine for three days) before infusion of CLDN6 CAR-T cells at DL1 (*n* = 7, including one patient with non-conformant product with lower yield) or DL2 (*n* = 13, including one patient with 50% reduced LD). Four patients at DL1 and seven patients at DL2 were treated in combination with CARVac. In two patients who were at risk of prolonged cytopenia, we explored omitting LD (Extended Data Tables 1 and 2).

Patients treated at DL1 or with dose-reduced LD received a median of 3.5 doses of CARVac (range 1–6) starting at day 4 post-adoptive cell transfer (ACT). For patients treated at DL2 with full dose LD, CARVac administration was delayed to reduce the risk of accelerated and high-grade cytokine release syndrome (CRS), with CARVac starting 23 days or more post-ACT with a median of five (range 2–9) doses. The two patients at DL2 without prior LD were treated with two and four doses of CARVac, respectively. Two patients at DL2 crossed over and received CARVac (≤3 doses) starting 65 and 79 days post-ACT, respectively. Five patients were redosed at their original DL 190–288 days after their first ACT in combination with CARVac starting on day 4 (DL1) or day 15–16 (DL2) post redosing, respectively. One patient at DL1 was redosed without prior LD (Extended Data Table 1).

Overall, assessment of CLDN6-positivity followed by manufacturing and administration of CLDN6 CAR-T cells ± CARVac was feasible in this population of heavily pretreated patients including individuals with rapidly progressing disease.

### Safety

All 22 patients treated within the study were included in the safety analysis. The data cutoff date was 6 October 2022, with a median follow-up of five months (range: 29–416 days, Extended Data Table 1).

Nineteen of 22 patients had treatment-emergent adverse events (TEAEs) greater than or equal to grade (G) 3 (Extended Data Table 2). Besides pyrexia, the most frequent TEAEs observed in >20% of treated patients were hematologic toxicities related to LD or transaminase/lipase elevations without clinical correlates (Table 2), observed more often in patients experiencing CRS. TEAEs ≥G3 attributed to the CAR-T cell treatment that occurred in >10% patients were neutropenia (23% G4) and leukopenia (5% G3, 14% G4) (Extended Data Table 3).

**Table 2:** TEAEs

Event, number of patients (%)	Any grade	Grades 1–2	Grade 3	Grade 4
All TEAEs (in >20% of patients)				
Any	22 (100)	22 (100)	17 (77)	16 (73)
Pyrexia	14 (64)	14 (64)	3 (14)	0
Anemia	10 (46)	3 (14)	7 (32)	2 (9)
White blood cell count decreased	9 (41)	1 (5)	5 (23)	4 (18)
Fatigue	7 (32)	7 (32)	0	0
Headache	7 (32)	7 (32)	0	0
Leukopenia	7 (32)	2 (9)	2 (9)	4 (18)
Lipase increased	7 (32)	0	4 (18)	3 (14)
Neutropenia	7 (32)	0	0	7 (32)
Platelet count decreased	7 (32)	3 (14)	3 (14)	3 (14)
Hypotension	6 (27)	5 (23)	1 (5)	0
Influenza like illness	6 (27)	6 (27)	0	0
Nausea	6 (27)	6 (27)	0	0
Alanine aminotransferase increased	5 (23)	1 (5)	4 (18)	0
Aspartate aminotransferase increased	5 (23)	2 (9)	3 (14)	0
Back pain	5 (23)	5 (23)	0	0
Lymphocyte count decreased	5 (23)	0	2 (9)	4 (18)
Neutrophil count decreased	5 (23)	0	2 (9)	4 (18)
Vomiting	5 (23)	5 (23)	0	0
CRS-linked TEAEs (all)				
Any	10 (46)	10 (46)	4 (18)	1 (5)
Pyrexia	9 (41)	9 (41)	2 (9)	0
Hypotension	3 (13)	2 (9)	1 (5)	0
Alanine aminotransferase increased	1 (5)	0	1 (5)	0
Aspartate aminotransferase increased	1 (5)	0	1 (5)	0
Blood alkaline phosphatase increased	1 (5)	0	0	1 (5)
Dyspnea	1 (5)	0	1 (5)	0
Generalized edema	1 (5)	1 (5)	0	0
Headache	1 (5)	1 (5)	0	0
Hepatotoxicity	1 (5)	1 (5)	0	0
Hypoxia	1 (5)	1 (5)	0	0
Lipase increased	1 (5)	0	0	1 (5)
Nausea	1 (5)	1 (5)	0	0
Vomiting	1 (5)	1 (5)	0	0
ICANS-linked TEAEs (all)				
Any	1 (5)	1 (5)	0	0
Depressed level of consciousness	1 (5)	1 (5)	0	0
Headache	1 (5)	1 (5)	0	0
Immune system disorder	1 (5)	1 (5)	0	0

AEs graded for severity using National Cancer Institute Common Terminology Criteria for Adverse Events (NCI CTCAE) v.5.0, with CRS graded according to American Society for Transplantation and Cellular Therapy consensus grading^29^.

TEAE is defined as any adverse event (AE) with an onset date on or after the first administration of CLDN6 CAR-T (if the AE was absent before the first administration of CLDN6 CAR-T) or that worsened after the first administration of CLDN6 CAR-T (if the AE was present before the first administration of CLDN6 CAR-T). AEs with an onset date more than 90 days after the last administration of any investigational medicinal product (IMP) were considered as treatment-emergent if assessed as related to any IMP by the investigator.

**Extended Data Fig. 1:**
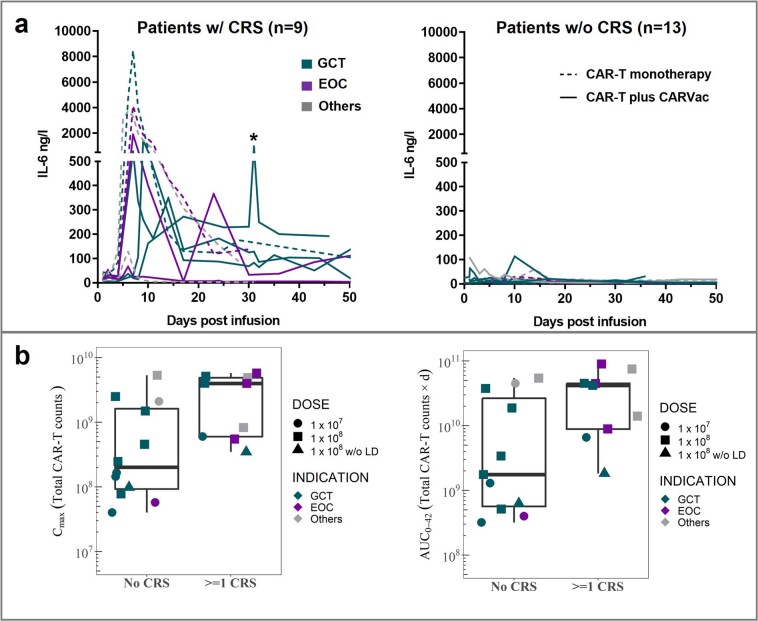
Cytokine release syndrome is directly correlated with CLDN6 CAR-T cell engraftment. **a**, IL-6 concentration post-ACT in patients who either experienced CRS (left panel) or did not (right panel). The only patient that developed G3 CRS is marked by ‘*’. One patient developed CRS G1 later than 50 days post-ACT, considered related to CARVac administration. **b**, Comparison of CAR-T cell peak concentration (C_max_, left panel) and AUC up to day 42 post-ACT (first tumor assessment, right panel) in patients with and without a CRS event. The patient experiencing G3 CRS is a GCT patient that did not receive LD (green triangle). Cancers of ‘other’ origin than GCT and EOC were each a single case of DSRCT, GC, serous carcinoma of the fallopian tube, endometrial carcinoma and CUP. Box plots show median and upper and lower quartiles, with whiskers indicating 1.5x the interquartile range. Individual data points are overlaid. ACT, adoptive cell transfer; AUC, area under curve; CRS, cytokine release syndrome; CUP, cancer of unknown primary; DL, dose level; DSRCT, desmoplastic small round cell tumor; EOC, epithelial ovarian cancer; G, grade; GC, gastric adenocarcinoma; GCT, germ cell tumor; LD, lymphodepleting chemotherapy.

CRS was seen in ten patients (46%), eight of whom were treated at DL2 (Extended Data Table 2). CRS typically occurred within 4–10 days post-ACT, was correlated with high IL-6 levels and peak CAR-T cell concentration post-ACT, was predominantly of G1–2, and was manageable by supportive care with antipyretics and the IL-6 receptor blocking antibody tocilizumab (Extended Data Table 2 and Extended Data Fig. 1). As CRS events were more frequently observed in the DL2 monotherapy cohort (Extended Data Table 2), the safety committee decided to delay CARVac dosing to day 24 in the DL2 combination cohort to avoid the risk of CAR-T cell-amplification related high-grade CRS.

One patient treated at DL1 plus CARVac had symptoms (headache and decreased level of alertness) classified as G1 immune effector cell-associated neurotoxicity syndrome (ICANS) that occurred in conjunction with G2 CRS and resolved spontaneously within 24 h (Extended Data Table 2).

Dose-limiting toxicities (DLTs) emerged in two patients treated at DL2, one with monotherapy and one in combination with CARVac (Extended Data Table 2). One event was G4 hemophagocytic lymphohistiocytosis (HLH) observed in an EOC patient, who had the highest CAR-T cell peak expansion (5.8 × 10^9^) observed in this trial. On day 5 post-ACT, the patient developed G2 CRS (treated with tocilizumab) and on day 23 experienced HLH defined by highly elevated ferritin levels and an aplastic bone marrow, which was successfully treated with high-dose steroids. CARVac treatment was postponed to day 51 until all CRS- and HLH-related AEs had resolved without sequelae.

The other DLT was prolonged G4 pancytopenia observed in a heavily pretreated GCT patient reported 21 days post-ACT. The patient was nonresponsive to granulocyte colony-stimulating factor, received an autologous peripheral blood stem cell support on day 32, upon which the patient’s bone marrow function recovered within 14 days. The patient was redosed with CAR-T cells 41 weeks after the first CAR-T cell administration. This DLT experience led to the decision to consider GCT patients who had recently undergone HDCT as at risk for treatment-related persistent cytopenia. Consequently, we introduced protocol amendments to make the availability of autologous peripheral blood stem cells a prerequisite for treatment of patients with HDCT within the last 12 months or with impaired bone marrow function with the full dose of LD. Alternatively, we allowed dose-reduced LD.

No further DLTs were observed after enrollment of three additional patients into each of these two DL2 cohorts. The Safety Review Committee (SRC) determined that the MTD (primary endpoint) was not reached. The recommended phase 2 dose of CLDN6 CAR-T cells was not further pursued as enrollment to the planned DL3 cohorts was canceled per the study protocol amendment to facilitate a repeat of the CAR-T dose escalation component with CAR-T cells manufactured with an automated process, including potential testing of higher dose levels. All eight deaths in the study were classified as disease progression and not attributed to CAR-T cell toxicity.

The safety profile of CARVac was in line with previous reports related to our RNA-LPX cancer vaccine platform^16,17^ and no unexpected toxicity was seen in combination with CAR-T cells (Extended Data Table 3). CARVac-related TEAEs were primarily of G1–2 flu-like symptoms occurring around 4 h after administration. TEAEs of ≥G3 that occurred with a frequency of ≥10% and were attributed to CARVac treatment were pyrexia (18% G3) and neutropenia (12% G4), which was also documented as related to LD. While dose reduction was allowed per protocol in the case of CARVac-related AEs, CARVac was generally well tolerated and no dose reductions occurred. After initial hospitalization, patients received CARVac in an outpatient setting and toxicities were managed with paracetamol.

**Extended Data Fig. 2:**
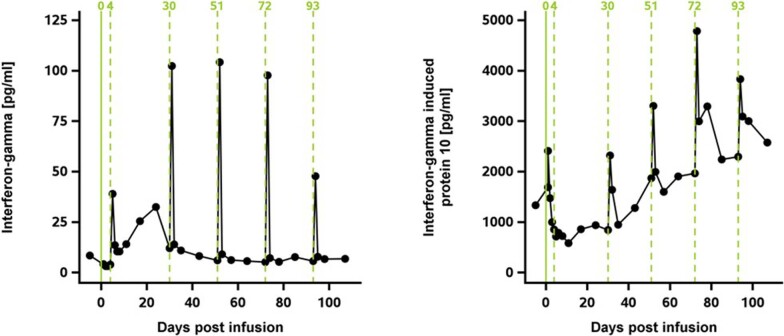
Transient cytokine release upon CARVac administration. Interferon-gamma and interferon-gamma inducible protein 10 serum levels were measured in a lymphodepleted patient prior to and following ACT of CAR-T cells (DL1, solid green line). CARVac (25 µg) dosing started 4 days post-ACT and was administered on d51, d72, and d93 (50 µg), denoted by green dashed lines. Cytokine measurements were taken prior to CARVac dosing, then 6 hr, 24 hr, and then weekly after each CARVac dose.

Transient release of IFNγ and IFNγ inducible protein 10, peaking 3–6 h post intravenous CARVac administration, was observed in serum cytokine measurements (Extended Data Fig. 2), as previously reported in patients treated with RNA-LPX mRNA cancer vaccines^15^.

In summary, the safety profile of both dose levels of CLDN6 CAR-T cells as monotherapy or in combination with CARVac was manageable (Table 2 and Extended Data Table 3) and largely in line with previous reports on approved CAR-T cell products^18^.

### Efficacy

**Fig. 2:**
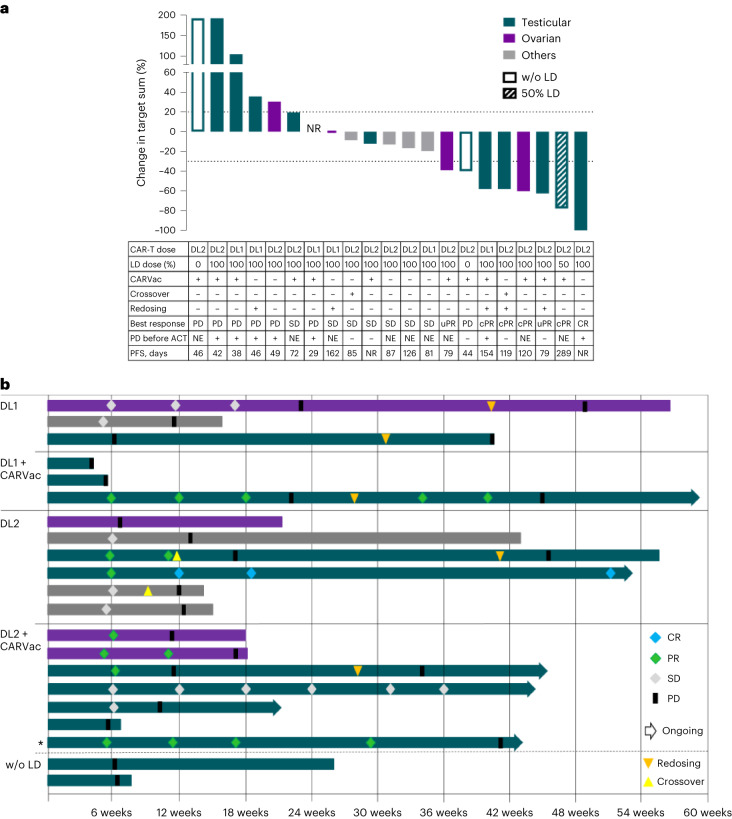
Clinical response to CLDN6 CAR-T infusion ± CARVac. **a**, Waterfall plot showing best percent change from baseline (screening, ~6 weeks before ACT) in sum of target lesion diameters. Efficacy evaluable population (*n* = 21) contains one patient that did not reach the first CT scan and was classified as PD (confirmed by X-ray). The table below indicates applied treatment, outcome and disease status at ACT (available for 12 patients). Redosing (*n* = 5) occurred after assessment of best response in all patients. **b**, Swimmer plots for all efficacy evaluable patients (*n* = 21). The patient with dose-reduced LD is marked with an asterisk. Diamonds represent outcome at tumor assessment (CR, PR, SD). Triangles indicate redosing with CAR-T cells at the same DL (orange) or crossover from monotherapy to combination therapy (yellow). Redosing was always performed as a combination treatment. All responses were assessed according to RECIST 1.1. The patient with CR had a residual radiographic abnormality interpreted as scar tissue, as there was no abnormal radiotracer uptake according to Positron Emission Tomography/Computed Tomography (PET-CT) 12 weeks post-ACT (Extended Data Fig. 5). Cancers of ‘other’ origin other than GCT and EOC were each a single case of DSRCT, GC, serous carcinoma of the fallopian tube, endometrial carcinoma and CUP. NE, non-evaluable; NR, not reached; PFS, progression-free survival.

**Extended Data Fig. 3:**
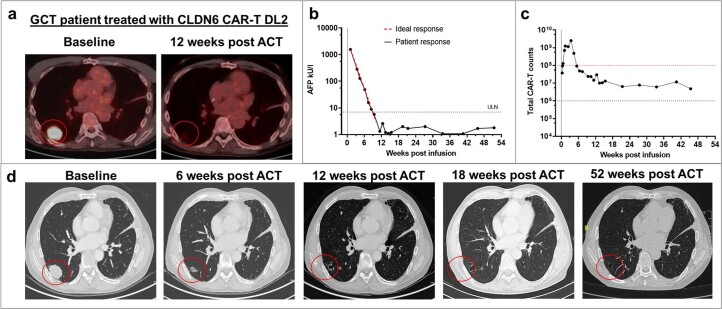
GCT patient with complete response and long-term CAR-T cell persistence after a single CAR-T cell dose. The patient was initially diagnosed with a mixed GCT, had been heavily pretreated (5 lines of chemotherapy in total) including cisplatinum-based chemotherapy, HDCT/ASCT,gemcitabine/oxaliplatin/paclitaxel, multiple surgeries and radiotherapy. After the 3^rd^ line chemotherapy consisting of HDCT carboplatine/etoposide, a late relapse 5 years later consisting of teratoma and yolk-sac tumor occurred. The patient experienced another relapse of a yolk-sac tumor component prior to trial entry and developed their first lung metastasis. The patient had rapidly progressing disease at accrual (increase in target sum of 37% between screening and ACT). 80% of their tumor cells had ≥2 + CLDN6 membrane staining positivity. **a**, PET-CT scans showing complete disappearance of the bulky lung metastasis and a complete metabolic response 12 weeks after treatment with a single dose of 1 × 10^8^ CLDN6 CAR-T cells following LD. **b**, Measurement of tumor marker AFP indicates an ideal response (50% reduction within 7 days) to treatment immediately starting after ACT, with control for the entirety of follow-up. **c**, CAR-T cell counts show strong expansion of CAR-T cells and persistence. **d**, CT scans show the complete rejection of the bulky lung metastasis with maintained CR up to the latest scan (52 weeks post-ACT). ACT, adoptive cell transfer; AFP, alpha-fetoprotein; CR, complete response; GCT, germ cell tumor.

**Extended Data Fig. 4:**
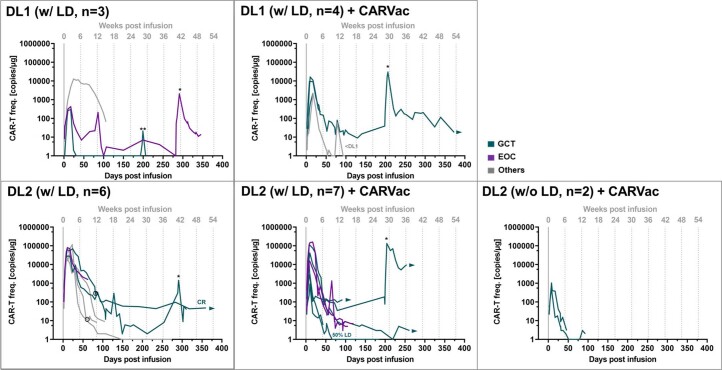
CLDN6 CAR-T cell frequency over time in all treated patients. CAR-T cell engraftment and kinetics was recorded in all treated patients (n = 22) from day of ACT until end of FU or data cut-off. Assessments ongoing at the time of data cut-off are marked by arrowhead. Patients are grouped by cohorts at indicated DL (solid line) including the two patients treated with ‘<DL1’ or with ‘50% LD’. Redosing of patients occurred at same DL in combination with CARVac either with (*, n = 4) or without (**, n = 1) LD. Crossover at DL2 (n = 2) is indicated by circle at start of additional CARVac treatment. CAR-T cells were detected in peripheral blood using quantitative PCR on DNA isolated from PBMCs. CAR-T frequency was calculated as CAR copy number divided by µg DNA of the sample. Cancers of ‘other’ origin than GCT and EOC were each a single case of DSRCT, GC, serous carcinoma of the fallopian tube, endometrial carcinoma and CUP. ACT, adoptive cell transfer; CUP, cancer of unknown primary; DL, dose level; DSRCT, desmoplastic small round cell tumor; EOC, epithelial ovarian cancer; FU, follow-up; GC, gastric adenocarcinoma; GCT, germ cell tumor; LD, lymphodepleting chemotherapy.

Twenty-one of the 22 patients were treated per protocol and qualified for efficacy analysis for the secondary endpoints ORR, disease control rate (DCR) and duration of response (DOR). Partial responses (PRs) in six patients and one complete response (CR) resulted in an unconfirmed ORR of 33% (Fig. 2a). Four patients with a best overall response (BOR) of PR remained in PR in subsequent scan(s), while two patients experienced progressive disease (PD) at the next assessment. We observed deepening of PRs over time, with further reductions in the sum of target lesions observed over repeat assessments. The median DOR for all seven responders was 2.8 months (range 1.1– 10.5 months) (Fig. 2b), with the CR ongoing at data cutoff for 10.5 months (Extended Data Fig. 3). Seven patients had stable disease (SD) as the best response, five with quantifiable target lesion shrinkage, resulting in a DCR of 67%. One SD was ongoing at data cutoff 8.7 months post-ACT. Five of the seven patients with PD had either received CLDN6 CAR-T cells at DL1 or had not received LD before DL2. The other two patients with PD were treated at DL2 with prior LD; however, they had rapid disease progression at accrual (61% and 86% increase in target sum between screening and ACT; Table 1). Five patients (three PRs, one SD, one PD as BOR to the first dosing) were redosed with CAR-T cells due to PD (Fig. 2b) resulting in one additional PR (Extended Data Fig. 4).

**Fig. 3:**
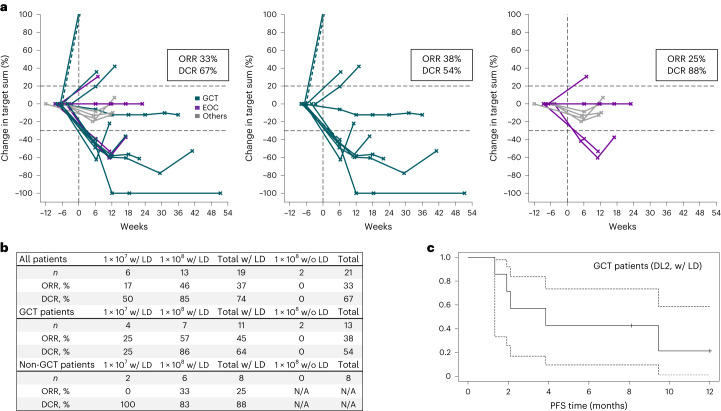
Clinical responses in the GCT and non-GCT patient subgroups. **a**, Spider plots indicating response durability in all patients (left, *n* = 21), GCT patients (middle, *n* = 13) and non-GCT patients (right, *n* = 8). Non-GCT patients include four EOC patients and one each of DSRCT, GC, serous carcinoma of the fallopian tube and endometrial carcinoma. Pretreatment CT scans are not included, as scans performed at screening (~6 weeks before ACT) served as baseline. **b**, Details on ORR and DCR of all patients as well as subgroups of GCT and non-GCT patients according to treatment (dose and LD). **c**, PFS analysis for *n* = 7 GCT patients treated at DL2 after LD with 95% confidence interval based on a log–log transformation of the survival function (dotted lines). ORR is unconfirmed.

All objective responses occurred in patients with either EOC (two of four patients with PR) or GCT (four PRs plus one CR in 13 patients; Fig. 3a,c). The five patients with other tumor entities all had SD as BOR (including the patient treated <DL1).

The ORR in the subgroup of GCT patients was 38% and depended on administered CLDN6 CAR-T DL and LD (Fig. 3a,b): 25% at DL1 after LD (one PR in four patients), 57% at DL2 after LD (four PRs in seven patients) and 0% at DL2 without LD (two patients). GCT patients were those with the longest DORs, leading to a PFS (exploratory endpoint) of 42% at six months for those treated with CLDN6 CAR-T cells at DL2 after LD (Fig. 3c). As the DL2 cohort had not reached median overall survival at the time of the data cutoff, and given the heterogeneity of the patients, indications and treatment schedules, we do not report overall survival (a further exploratory endpoint).

Of the eight non-responding GCT patients, five were treated at DL2. Two achieved SD as BOR, two were treated without prior LD and experienced PD, as did the fifth patient, who entered the study with a rapidly progressing tumor (86% increase in target sum from screening to ACT) having only 50% 2+/3+ CLDN6-positive tumor cells. Notably, of the two non-responding EOC patients, one was treated at DL2 and showed rapidly progressing disease (61% increase in target sum from screening to ACT) stabilizing after infusion (19% reduction from ACT to first assessment). Previous lines of treatment for patients are included in Supplementary Table 1.

Tumor responses were primarily observed at DL2. However, the late timing of CARVac dosing at this DL, combined with the diverse, small cohorts, prevented analysis of how CARVac influences the antitumor activity of CLDN6 CAR-T cells.

We observed encouraging signs of efficacy and disease control in a heterogenous cohort of hard-to-treat solid tumor patients, indicating GCT and EOC patients in particular as future target populations. In parallel, we developed an automated manufacturing process to scale up CAR-T production and increase the robustness of the manufacturing process. Recruitment of patients to a planned DL3 with CAR-T cells manufactured with the ‘manual’ manufacturing process was therefore canceled by protocol amendment, and we have initiated a repeat of dose escalation with CAR-T cells produced with the automated manufacturing process, introducing further dose levels for both CAR-T cells and CARVac.

**Fig. 4:**
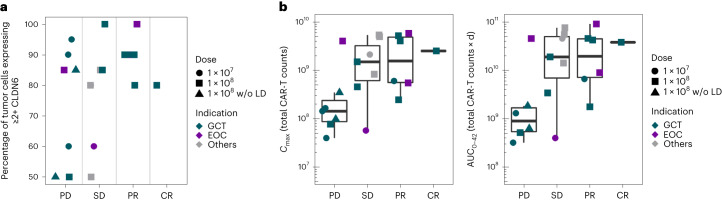
Clinical outcome is directly correlated with homogeneity of CLDN6 tumor expression and CLDN6 CAR-T cell expansion. **a**, Correlation of clinical outcome and CLDN6 expression of the corresponding tumor according to indication and treatment (dose and LD). **b**, Correlation analysis of CAR-T cell peak concentration (*C*_max_) (left) and AUC up to day 42 post-ACT (first tumor assessment) (right) with outcome. Cancers of ‘other’ origin other than GCT and EOC were single cases of DSRCT, GC, serous carcinoma of the fallopian tube and endometrial carcinoma. Box plots show median and upper and lower quartiles, with whiskers indicating 1.5× the interquartile range. Individual data points are overlaid.

Identification of predictive biomarkers was an exploratory endpoint. All responding patients had tumors with >80% of tumor cells expressing 2+/3+ CLDN6 at prescreening (Fig. 4a), suggesting that CLDN6 expression level may be predictive of outcome. Four patients had PD despite high CLND6 expression, three of which entered the trial with progressing disease (61%, 99% and 105% increase in target sum from start of screening to ACT). The fourth patient did not receive LD and experienced poor CAR-T cell engraftment. A positive correlation between CLDN6 CAR-T expansion and clinical response was detected for both peak expansion (CAR-T cell *C*_max_) and area under curve (AUC) from ACT to first staging with CT (Fig. 4b).

### Pharmacokinetics

**Extended Data Fig. 5:**
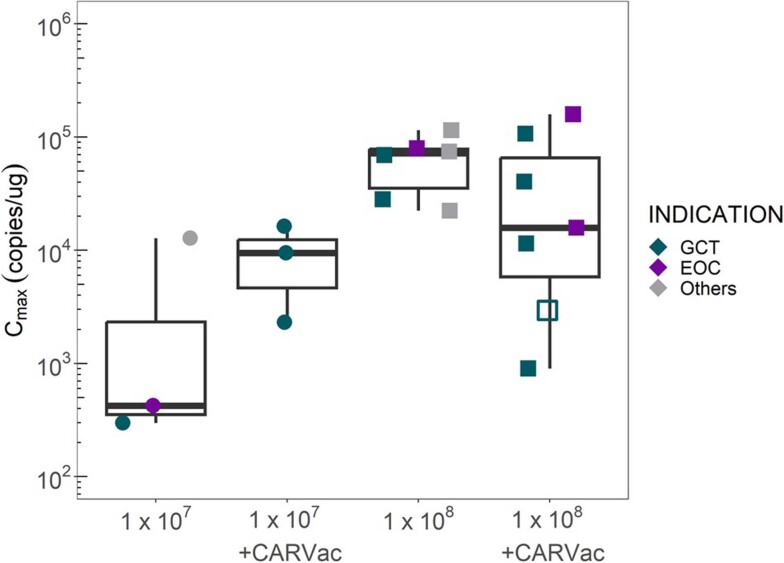
CAR-T cell peak numbers in patients treated with CLDN6 CAR-T cells +/- CARVac. Peak numbers were reached on average within 18 days (range 17–24) for patient treated at DL1 (circles) and 15.6 days (range 8–24) for patients treated at DL2 (squares). 3 patients treated at DL1 received CARVac 4 days after ACT, 7 patients treated at DL2 received CARVac on day ≥ 23 post-ACT. Numbers are presented as CAR copies per µg genomic DNA. Shown are patients treated with LD, peak CAR-T number for one patient treated with a 50% reduced course of LD is shown as a non-filled square. Box plots show median and upper and lower quartiles, with whiskers indicating 1.5x the interquartile range. Individual data points are overlaid.

**Extended Data Fig. 6:**
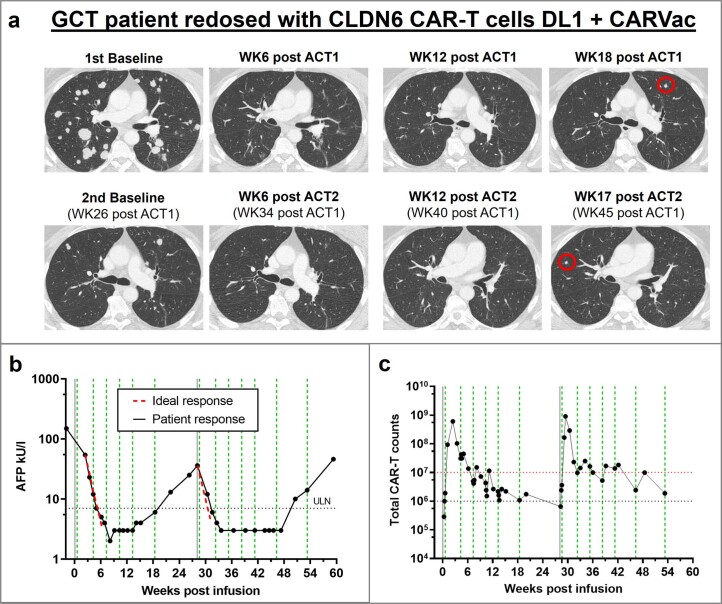
Multiply pretreated GCT patient with repeated response upon CLDN6 CAR-T cell + CARVac redosing. The GCT patient (non-seminoma, yolk-sac subtype) had multiple lung metastases and rapidly progressing disease at accrual (increase in target sum of 40% between screening and ACT). Tumor biopsy and immunohistochemical analysis revealed 90% positivity for ≥2 + CLDN6 membrane staining. Following LD and ACT with 1 × 10^7^ CLDN6 CAR-T cells in combination with multiple doses of CARVac (green dashes lines in B and C) he showed the only PR observed in patients treated at this lower dose level. First suspected new lesions occurred at week 18 post-ACT (red circle). Upon confirmed progression 22 weeks post-ACT (DOR of 3.7 m) and as an on-treatment biopsy was 100% CLDN6 positive, the patient was redosed (28 weeks post first ACT) at DL1 + CARVac after LD. The patient experienced another PR for multiple weeks until new lesions were confirmed (red circle)**. a**, CT scans showing response of lung lesions after the first (ACT1) and the redosing (ACT2). **b**, Local measurement of tumor marker AFP indicates an ideal response (50% reduction within 7 days) to treatment immediately starting after ACT, reaching normalization and persistence of control for ~18 weeks. **c**, CAR-T cell kinetics by quantitative PCR analysis of DNA extracted from peripheral blood mononuclear cells (PBMCs). Note transient elevations in CAR-T cell counts after CARVac administrations (dashed green lines). Total CAR-T cell counts were calculated based on white blood cell count on the days of blood drawing (if available) and an assumed blood volume of 5 L. **b** and **c**: vertical grey lines are (re)dosing with CAR-T cells, dashed green lines are dosing with CARVac. ACT, adoptive cell transfer; AFP, alpha-fetoprotein; GCT, germ cell tumor; PD, progressive disease; PR, partial response; WK, week.; ULN, upper limit of normal.

Characterization of the pharmacokinetics of CLDN6 CAR-T cells was an exploratory endpoint. For lymphodepleted patients at DL1 and DL2, CAR-T cell peak expansion (*C*_max_) detected in peripheral blood was reached on average within 18 days (range 17–24) and within 15.6 days (range 8–24), respectively, with higher peak engraftment seen at DL2 (Extended Data Fig. 5). The two patients treated at DL2 with CARVac but without LD displayed poor engraftment, and this cohort was therefore closed (Extended Data Fig. 6). Two patients treated at DL1 (29%) and six patients treated at DL2 (46%) showed CAR-T cell persistence for ≥100 days post-ACT, with CAR-T cells in one patient treated once at DL2 detectable for >1 year (Extended Data Fig. 3). Four patients (two at DL1, two at DL2) with decrease of CAR-T cells over time were preconditioned again and redosed with CAR-T cells in combination with CARVac 190–288 days after their initial ACT (exemplified by a patient at DL1 + CARVac in Extended Data Fig. 6). Robust engraftment with almost phenocopied kinetics of their first ACT was achieved in three patients; all three had detectable CAR-T cells at their last follow-up. One patient preconditioned with LD before their first ACT was redosed at DL1 without LD and reached a lower *C*_max_ compared to their first ACT.

CARVac was administered to 11 patients who had received CLDN6 CAR-T cells after LD and two patients who received CAR-T cells without prior LD (Extended Data Fig. 6). Within this group, four patients were given CARVac following CAR-T cells at DL1. A trend for greater expansion of CAR-T cells was observed at DL1 in patients receiving CARVac compared with patients receiving CLDN6 CAR-T cells alone (Extended Data Fig. 5). Notably, the patient who received CAR-T cells at <DL1 in conjunction with CARVac had a peak CAR-T cell count (*C*_max_) (Extended Data Fig. 6, top right panel) that exceeded those observed in two of the three patients treated at DL1 without vaccine (Extended Data Fig. 6, top left panel). While patients in the DL1 cohort received CARVac four days after ACT, the seven patients treated at DL2 received CARVac 23 days or more post-ACT (range 23–51, Extended Data Tables 1 and 4). The longer dosing interval was stipulated per trial protocol as a safety precaution in this first-in-human trial, as discussed in the safety section. Thus, patients in the DL2 cohort received CARVac post-CAR-T peak expansion, and the effects of CARVac on CAR-T cell engraftment could therefore not be assessed at this DL.

Given the limited sample size, the heterogeneity of patients and unequal initial engraftment within the subgroups at DL2, the impact of CARVac on persistence of CAR-T cells cannot be statistically evaluated with the data available at this initial cutoff. However, we noted a transient increase in CAR-T cells immediately following CARVac administration in some patients (Extended Data Fig. 4c). In two patients treated with CAR-T cells at DL2 who commenced CARVac at a later stage, CARVac appeared to halt an ongoing decline in CAR-T cell frequency (Extended Data Fig. 6, bottom left). A further four patients in monotherapy cohorts crossed over to combination therapy, two after redosing with CAR-T cells at DL1 and two (indicated with a circle in Extended Data Fig. 6) at DL2 that experienced PD before crossover. No conclusions can be reached from this heterologous group that had differing CARVac vaccination schemes.

Further analysis with a larger patient cohort treated with CARVac at an earlier time point is planned to provide a more conclusive understanding of the impact of CARVac on CAR-T cell dynamics.

## Discussion

Extending the success of CAR-T cells to solid tumors presents significant hurdles, primarily due to the scarcity of distinct, tumor-specific targets, complicating the development of efficacious CAR-T therapies for these cancers. Notwithstanding these obstacles, several early-phase clinical trials are underway targeting molecules including mesothelin, Muc16, Folate receptor and carcinoembryonic antigen^19^. Promising clinical data have been observed with CAR-T cells targeting IL13RA2 or EGFRv3 in glioblastoma patients^20^, the disialoganglioside GD2 in neuroblastoma^21^ and H3K27M-mutated diffuse midline gliomas^22^, Prostate-specific membrane antigen in metastatic prostate cancer^23^ and CLDN18.2 in patients with gastrointestinal cancers^24^. These treatments have led to notable reductions in tumor markers and observable responses in select patients.

The effectiveness and safety of CAR-T cell therapy largely hinges on on-target specificity. Targeting antigens such as her2neu, GD2 and CLDN18.2 that are also expressed by non-tumor tissue could lead to on-target, off-tumor toxicity where the antigen in healthy tissue is accessible to CAR-T cells^5^. On-target, off-tumor toxicity was reported in a clinical trial testing CAR-T cells directed against CLDN18.2, a tight junction protein expressed by differentiated gastric mucosal membrane epithelial cells with otherwise highly limited expression in healthy tissues that is also expressed in digestive system tumors. In addition to a minority (6 of 37) of patients experiencing predominantly grade 1–2 mucosal injury AEs, a grade 3 mucosal erosion event was reported^24^. Although the safety profile in this cohort was deemed acceptable, other first-in-human clinical trials testing CAR-T cells in solid tumors have uncovered sometimes unexpected instances of severe on-target, off-tumor toxicity^5^.

To our knowledge, this is the first report on the clinical performance of CAR-T cells directed against CLDN6, an oncofetal antigen with no detectable CLDN6 expression in healthy adult human tissues^10^. One of the key findings of our interim analysis of first-in-human data was the manageable AE profile, with a lack of on-target/off-tumor toxicity attributable to cognate CLDN6 CAR-T cell activity at the tested DLs.

The addition of the RNA-LPX-based CARVac at doses determined in previous trials did not alter the safety profile of CLDN6 CAR-T cells substantially.

The TEAEs we observed (for example, CRS and one case each of HLH (G4) and ICANS (G1)) have been reported for CAR-T cell products used in hematological malignancies, and are most likely associated with this treatment modality. CRS was mostly G1–2, well manageable and resolved without sequelae.

Other TEAEs ≥G3 were hematological ones, for example, cytopenia. GCT patients with impaired bone marrow function due to recent prior intensive myelosuppressive treatment were found to be especially at risk for treatment-related prolonged bone marrow suppression. Our observations indicate that omitting LD altogether may not be an option due to lack of CAR-T expansion without LD. To mitigate risks of enhanced hematotoxicity associated with prior treatments typical for the cancer types in this study, we continue to assess approaches such as dose-reduced LD protocols supported by CARVac-mediated CAR-T cell expansion. All reported safety-relevant findings (including asymptomatic transaminase/lipase elevations) had resolved by the time of this interim analysis.

Overall, the study supports CLDN6 as a safe target for cell therapy in solid tumors and does not raise concerns for either combining CAR-T cells with CARVac or for exploring higher doses of CLDN6 CAR-T cells. Given that CARVac dosing improved engraftment for patients treated at DL1 dosed with CARVac four days post-ACT but not for those at DL2 dosed following peak expansion (23–51 days post-ACT), future cohorts will be treated at the earlier time point regardless of the dosing level. With this trial being continued, the safety data set will be extended, allowing more detailed characterization of the preliminary observations we made in the first 22 patients.

This interim analysis showed encouraging clinical activity of CLDN6 CAR-T cells with and without CARVac in a heterogenous set of patients. Enrolled patients were heavily pretreated, had active disease with r/r solid tumors, and the baseline CT scans were performed on average almost six weeks before ACT, altogether being conditions that may underestimate the potential clinical activity of CLDN6 CAR-T cells.

In the higher DL of 1 × 10^8^ CAR-T cells, almost half of the patients experienced an objective response and disease control was observed in 85% of patients. Most of the seven patients who achieved PR at their first scan six weeks post-ACT experienced deepening of responses, with one GCT patient transitioning to a CR that has been ongoing for more than one year after a single dose of 1 × 10^8^ CLDN6 CAR-T cells without CARVac or any further treatment. Seven patients had SD; this was still ongoing for one patient at data cutoff (8.7 months post-ACT).

More than half of the patients suffered from GCT. Subgroup analysis of those GCT patients treated with 1 × 10^8^ CLDN6 CAR-T cells in conjunction with LD at full or reduced dose showed an ORR of 57% and a DCR of 85% (*n* = 7; one CR, three PRs, two SDs). As the PFS of GCT patients who are not eligible for or relapsed after HDCT and do not have further curative treatment options is reported to be ~10% at six months^25,26^, the PFS of 42% at six months achieved with CLDN6 CAR-T cells in the seven GCT patients is compelling. The treatment outcomes of the BNT211 testicular cancer cohort compare very favorably with many previous early-phase clinical trials that investigated targeted treatments and immunotherapies in r/r GCTs^27^. Our data support further development of CLDN6-targeted immunotherapies in this rare and difficult-to-treat disease setting.

The remaining nine enrolled patients were too heterogenous in terms of tumor types for indication-specific assessment of CAR-T cell activity. Of the three patients with EOC treated at the higher DL, two experienced an objective response warranting further exploration of this tumor type.

An important finding was the robust engraftment and expansion of CLDN6 CAR-T cells after a single administration, which has been a limiting factor for a number of clinical trials of CAR-T cells in solid tumors^28^. Over time we observed a steady decline in CAR-T cell counts in the majority of patients. One patient with a PR, who was redosed upon relapse, exhibited a renewed tumor marker response indicating that persistence of CAR-T cells is important for increasing the durability of clinical responses.

CARVac administration was well tolerated among trial participants, signifying its potential for combination therapy with CAR-T cells. Upon receiving CARVac, patients typically experienced a brief period of fever. Transient interferon-gamma release was observed post-CARVac administration indicating proper innate immune stimulation. In addition to these indicators of immune stimulation, we observed temporary increases in the frequency of CAR-T cells in some patients who received CARVac. While these preliminary findings indicate that CARVac may aid in boosting the expansion of CAR-T cells, potentially enhancing their therapeutic potency, the overall impact of CARVac on CAR-T cell pharmacokinetics is difficult to quantify based on the highly heterogeneous and small-sized set of patients analyzed. Given the importance of long-term persistence and robust expansion of CAR-T cells and motivated by these promising early findings, we continue to investigate the effects of higher doses of CARVac and assess the impact of repeated vaccine administration on prolonging persistence in this ongoing trial.

As this study is a single arm trial with no comparator or control group, selection bias cannot be ruled out. The study is further limited by the small size, a relatively short follow-up duration with patients still on trial at the time of data cutoff, and by the all-comer nature of the trial, which resulted in recruitment of individual patients with distinct tumor indications, meaning that the suitability of these indications for treatment with CLDN6-directed CAR-T cells remains unclear. Treatment of more patients with standardized CARVac treatment algorithms are required to draw clear conclusions about the potential role of CARVac in supporting CAR-T cell engraftment and persistence.

In conclusion, CLDN6 CAR-T cells resulted in robust engraftment, with a manageable safety profile and strong signals of clinical activity in patients with CLDN6-positive solid tumors with high unmet medical need. The encouraging response rates in these high medical need populations led us to develop an automated manufacturing process to scale up manufacturing, and we have initiated a repeat of dose escalation with this IMP, introducing further dose levels for both CAR-T cells and CARVac. Following identification of a recommended phase 2 dose, the trial will proceed to open expansion cohorts in select indications.

## Methods

### Trial design, patients and treatment

This first-in-human, open label phase 1/2 trial is being conducted at six sites in Germany (University Medical Center Hamburg-Eppendorf, University Hospital of Cologne, University Hospital Regensburg, Hannover Medical School, University Hospital Erlangen and the University Medical Center Mainz) and one site in the Netherlands (Netherlands Cancer Institute, Amsterdam) to assess dose levels of CLDN6 CAR-T cells with or without CARVac using a 3 + 3 dose escalation approach. We recruited adults with a diagnosis of metastatic or unresectable solid tumor of any indication with no further treatment options, intermediate or high CLDN6 membrane staining intensity in at least 50% of tumor cells as assessed by semiquantitative immunohistochemistry, and measurable disease per RECIST 1.1. Patients were screened for adequate coagulation, hepatic, renal and hematologic function. GCT patients without initial measurable disease per RECIST 1.1 were eligible for this trial if they had disease evaluable by cancer antigen-125, Alpha-fetoprotein (AFP) or human chorionic gonadotropin (hCG, as applicable), which was introduced by protocol amendment (note that all patients recruited to this trial had measurable disease). Men were not allowed to father a child or donate sperm, and women of child-bearing potential had to agree not to become pregnant. Patients previously treated with another CAR-T cell therapy, who had received vaccination with live virus vaccines within six weeks before the start of LD, who had an active autoimmune disease or human immunodeficiency virus/hepatitis C positivity, or who were receiving concurrent systemic steroid therapy >10 mg were not eligible. Evidence of new or growing brain or spinal metastases during screening was a further exclusion criteria. A history of another primary cancer within the two years before enrollment also precluded entry into the trial, except for the following: non-melanoma skin cancer, cervical carcinoma in situ, superficial bladder cancer, prostate cancer with currently undetectable prostate specific antigen, or other non-metastatic carcinoma that has been in complete remission without treatment for more than two years. The full list of inclusion/exclusion criteria can be found in the trial protocol, available online at 10.1038/s41591-023-02612-0.

As the decision was taken to repeat dose escalation using CAR-T cells manufactured using an automated process, a protocol amendment was introduced to cease recruitment to a planned third dose level using the manually produced CAR-T cells. This analysis of the 22 patients dosed between December 2020 and February 2022 with two DLs of CLDN6 CAR-T cells manufactured from autologous leukapheresis material by a manual process was therefore introduced by protocol amendment. All but two patients underwent LD (500 mg m^−^^2^ cyclophosphamide plus 30 mg m^−^^2^ fludarabine on days −5 to −3) before ACT. A third patient received LD at 50% reduced dose intensity. These modified LD regimes were introduced by protocol amendment. A single intravenous infusion of 1 × 10^7^ (DL1) or 1 × 10^8^ (DL2) CLDN6 CAR-T cells was administered. Redosing with CLDN6 CAR-T cells was allowed if endorsed by the SRC. All patients were hospitalized for LD and ACT; most patients were discharged within the first two weeks.

The dose for CARVac was selected based on prior clinical trial experience with the RNA-LPX vaccine platform in several hundred patients (for example, NCT04534205, NCT03289962, NCT03815058 and NCT04486378). In these trials, 25–100 µg RNA-LPX were determined as an active dose range, inducing strong in vivo activation of endogenous T cells with manageable safety and tolerability^16,30–33^. Accordingly, CARVac was administered as a single 25 µg dose, followed by repeated dosing at 50 µg. Dose reduction to 12.5 µg was allowed if necessary due to CARVac-related AEs. Bifurcation at any given dose level required that the SRC deemed the CAR-T dose level safe as a monotherapy. Allocation of patients to DL2 monotherapy and DL1 combination therapy, and thereafter to the DL2 cohorts with and without LD (for GCT patients), proceeded with staggered allocation according to the patients’ order of enrollment. Patients treated with CARVac in CLDN6 CAR-T DL1 and patients with dose-reduced LD in DL2 received dosing once every 3 weeks (1q3w) starting from day 4 post-ACT, switching to once every 6 weeks (1q6w) after five doses. Patients in DL2 were treated once every 2 weeks (1q2w) for three doses starting 23 days or more post-ACT, switching to 1q3w for two additional doses, and then to a 1q6w maintenance schedule. CARVac was administered in an outpatient setting for discharged patients. Dose reductions were allowed in case of severe toxicities. Crossover from monotherapy cohorts was introduced by protocol amendment and required SRC endorsement.

For further details, please refer to the trial protocol and statistical analysis plan in the Supplementary Information.

### Study oversight

The study protocol was approved by institutional review boards of the authorities in Germany (Paul-Ehrlich-Institute) and the Netherlands (Central Committee on Research involving Human Subjects). The study was conducted in accordance with the Declaration of Helsinki and International Conference on Harmonization guidelines for Good Clinical Practice. Written informed consent was obtained from all patients. Initial drafts of the manuscript were prepared by a subset of the authors and all authors contributed to the final manuscript. All authors made the decision to submit the manuscript for publication.

### Endpoints and assessments

The primary endpoints of the study were safety and tolerability of CLDN6 CAR-T cells with or without CARVac and identification of the MTD/recommended phase 2 dose. AEs and serious (S)AEs were collected from signing the informed consent form until 90 days after the last administration of the study drug. Key secondary endpoints included the ORR, the DCR and the DOR per RECIST 1.1. DOR was calculated as the time from first occurrence of CR or PR to the first occurrence of PD (recurrence or death from any cause, whichever occurs first). If patients were redosed with CAR-T cells, the time from first PR/CR to first PD was counted. Exploratory endpoints included overall survival and PFS, CLDN6 expression in tumor tissue, serum cytokines and CAR-T cell persistence.

Safety and efficacy parameters were reviewed weekly by the SRC. The DLT period was 28 days. Relatedness of AEs was captured for bridging therapy, LD, CAR-T cells and CARVac. CRS was graded according to American Society for Transplantation and Cellular Therapy guidelines, and ICANS was assessed by immune effector cell encephalopathy score^29^. Management of CRS and the grading and management of CAR‑T‑cell‑related encephalopathy syndrome and CAR-related HLH was in line with the recommendations of the CAR-T cell therapy-associated TOXicity Working Group^18^. Response monitoring included tumor marker assessments if applicable and CT scans every six weeks with evaluation per RECIST 1.1. Baseline was defined at screening visit.

CLDN6 CAR-T cell frequency in peripheral blood measured by quantitative polymerase chain reaction (qPCR) was used to calculate CAR-T cell counts, AUC and maximal concentration (*C*_max_) of CLDN6 CAR-T cell frequency.

### Statistical analysis

This interim analysis was conducted with a data cutoff date of 6 October 2022 and followed the statistical analysis plan. The primary efficacy analysis is defined as the ORR, but PFS, overall survival (OS) and DOR were included as secondary/explorative endpoints. The efficacy analyses were conducted with the modified intent to treat population (that is, any patients who had been treated with a conformant dose of the CAR-T product and who had a baseline and at least one on-treatment/posttreatment tumor assessment). The safety analysis set comprises all patients treated with a CAR-T cell product, while patients in the efficacy analysis set were treated with a conformant CLDN6 CAR-T product (≥1 × 10^7^ cells) and had at least one scan or died.

The rates of binary endpoints such as efficacy variables (ORR and DCR) were calculated by cohort along with the corresponding two-sided 95% confidence intervals using the Clopper–Pearson method.

Time-to-event endpoints (DOR and PFS) were analyzed using Kaplan–Meier methodology and censored in accordance with the Food and Drug Administration guidance *Clinical Trial Endpoints for the Approval of Cancer Drugs and Biologics*^34^.

The median event/survival time in days (including two-sided 95% confidence limits according to Brookmeyer and Crowley^35^) and the first and third quartile are presented for each cohort.

Correlative analyses are descriptive and exploratory. Data were analyzed and depicted using R programming (v.4.1.2) and GraphPad Prism 9.

#### CAR-T cell manufacturing

The CLDN6-CAR was constructed by linking the signal peptide sequence of an immunoglobulin heavy chain variable region (GenBank no. AAC18316.1) to a human CLDN6-specific single chain variable fragment (scFv) derived from the IMAB206-C46S antibody (WO2012/1560018). The scFv fragment is fused to a human CD8α hinge and transmembrane region (GenBank no. NP_001759.3, amino acids (aa) 138–206) followed by human 4-1BB (GenBank no. NP_001552.2, aa 214–255) and human CD3ζ (GenBank no. NP_000725, aa 52–163, Q65K) signaling moieties. The CAR-T (WO2020/161186) and CARVac (W2016/180778) technology is patented. Autologous peripheral blood mononuclear cells (PBMCs) were isolated from leukapheresis and cryopreserved until transduction. After thawing the PBMCs, T cells were enriched and activated by anti-CD3/CD28 beads and cultured with IL-7 (450 U ml^−1^) and IL-15 (50 U ml^−1^) for three days. T cell transduction was performed using the gamma-retroviral self-inactivating pES.12-6 retroviral vector encoding for the CLDN6 CAR at a multiplicity of infection of one. At day 7, cells were harvested and cryopreserved.

#### CAR-T cell immunophenotyping

Standard staining and flow cytometry techniques were used to perform immunophenotyping of surface markers on BNT211 CAR-T cell products at the manufacturing site. Cells were stained with CD3-FITC, CD4-APC and CD8-PE (BD Multitest catalog no. 342417; 1:11 dilution) in the bulk drug product, and CD3-PerCP, CD4-FITC, CD8-PE (BD, Tritest, catalog no. 342414; 1:6.25 dilution) and murine F(ab’)2-fragment-AF647 (anti-CAR antibody) (Jackson ImmunoResearch catalog no. 115-605-006; 1:100 dilution) in the drug substance. An AF647 conjugated murine isotype control (Jackson ImmunoResearch catalog no. 005-600-003; 1:143 dilution) was used as a control. The frequencies of naïve-like T cells, central memory T cells, effector memory T cells and effector memory re-expressing CD45RA T cells were defined by CD3-PerCP (BD catalog no. 345766; 1: 5 dilution), CD4-BV421(BD catalog no. 565997; 1:50 dilution), CD8-BB515 (BD catalog no. 564526; 1:20 dilution), CD197-PE (BD catalog no. 560765; 1:5 dilution) and CD45RA-BV510 (BD catalog no. 563031; 1:25 dilution) in the bulk drug product. All antibodies for analysis were purchased from BD Biosciences and Jackson ImmunoResearch. Samples were analyzed on a FACSVerse flow cytometer (BD Biosciences) using the gating strategies shown in Supplementary Fig. 4. Data were analyzed using the FACSuite Software v.1.0.6 (BD Biosciences).

#### CLDN6 CARVac manufacturing

RNA, liposomes and RNA-LPX were manufactured under good manufacturing practice conditions. The CLDN6 CARVac RNA is a highly purified, non-nucleoside-modified, uridine-containing RNA.

The single-stranded, 5′-capped mRNA was produced by in vitro transcription from the corresponding DNA template encoding CLDN6. The general structure of the CLDN6 RNA consists of 5′-cap, 5′-and 3′-untranslated regions, CLDN6 coding sequence and poly(A)-tail. In addition to the coding sequence, the RNA comprises sequence elements to enhance RNA stability and translational efficiency of the encoded antigen that have been optimized during development. The RNA backbone is optimized for translation of the coding sequence in human dendritic cells^36,37^ and subsequent induction of antigen presentation on human leukocyte antigen class I and II molecules^38^. Stimulation of Toll-like receptor-mediated, type-I-interferon-driven antiviral immune mechanisms then induces expansion of antigen-specific T cells^15^.

Liposomes with net cationic charges were used to complex the RNAs to form RNA-LPX. The cationic liposomes were manufactured using an adopted proprietary protocol based on the ethanol injection technique from the cationic synthetic lipid (R)-N,N,N trimethyl-2-3-dioleyloxy-1-propanaminium chloride (R-DOTMA) (Merck and Cie) and the phospholipid 1,2-dioleoyl-sn-glycero-3-phosphoethanolamine phospholipid (DOPE) (Corden Pharma)^39^. Release analysis for the liposomes included determination of appearance, lipid concentration, RNase presence, particle size, osmolality, pH, subvisible particles, pyrogen testing and sterility.

The injectable RNA-LPX drug products were prepared in a dedicated pharmacy by incubating the individual concentrated RNA drug products with an isotonic NaCl solution (0.9%) (Fresenius Kabi) and cationic liposomes according to a proprietary protocol^15,16^. The RNA-LPX preparation protocol was derived from previously published protocols for nucleotide lipoplex formation^15,40^. Before injection, the RNA-LPX was further diluted with an isotonic NaCl solution (0.9%) (Fresenius Kabi) to the intended concentration. Periodic quality control of RNA-LPX drug products included determination of RNA content, RNA integrity, particle size and polydispersity index.

#### Clinical assessments

Laboratory anomalies were screened by hematology testing, coagulation assay, serum chemistries, cytokine levels and urinalysis. Physical exams including 12-lead electrocardiograms and vital signs were performed daily during hospitalization periods for assessment of toxicities, including assessment of the immune effector cell encephalopathy score. Treatment responses were monitored by disease marker evaluation, measurement of serum cytokines, and assessment of tumor responses according to RECIST 1.1 (including time from first objective response to first occurrence of objective progression, recurrence, or death from any cause) using unidimensional measurement such as a CT scan or magnetic resonance imaging during pretreatment screening (average 5.9 weeks before ACT), at six weeks post-CLDN6 CAR-T cell infusion, then every six weeks for 50 weeks and then every 12 weeks thereafter. All patients had at least one tumor assessment post treatment, except for a treated patient who died before the first CT scan and was classified as PD.

#### Histology

Histological and immunohistochemical analyses of all cases were performed in the central histology lab at BioNTech SE on representative tissue slides of formalin-fixed, paraffin-embedded neoplastic tissues. In brief, slides were stained with hematoxylin and eosin on a Leica ST5020 multistainer and manually stained using a monoclonal mouse anti-human CLDN6 antibody (CLAUDENTIFY6, BioNTech Diagnostics) and a normal control reagent. Embryonal rabbit kidney tissue served as a positive control for each immunohistochemical staining. All samples were analyzed by board-certified pathologists regarding tumor content (hematoxylin and eosin) and CLND6 expression in neoplastic cells. CLDN6 staining positivity was classified as negative (0), weak intensity (1+), medium intensity (2+) and strong intensity (3+). Only membranous staining was considered.

#### Spearman rank correlation analysis

The Spearman’s rank correlation coefficient was calculated between the age of the archival tumor tissue and the percentage of tumor cells with 2+/3+ positive CLDN6 staining at prescreening for both EOC and GCT samples.

The figure was created with the SAS 9.4 Graph Template Language, using the SAS Enterprise Guide v.8. The Spearman coefficients and *P* values were computed using the SAS CORR Procedure.

#### Monitoring of CAR-T engraftment by PCR

CAR-T cell engraftment was assessed in peripheral blood by a duplex qPCR amplification assay targeting the Woodchuck Hepatitis Virus Posttranscriptional Regulatory Element (WPRE), a sequence part of the vector encoding the CLDN6-CAR and an intronic sequence from FOXP2, which serves as a reference for normalization of cell numbers. Genomic DNA (gDNA) was isolated from cell pellets, and DNA concentration was measured and corrected to a target concentration of isolated gDNA of 125 ng µl^−1^. CLDN6 CAR-T vector copies were then determined by qPCR duplex assay targeting WPRE and a FOXP2 intron. The copy numbers of WPRE and FOXP2 were calculated from CT values based on calibration curves of WPRE and FOXP2 gene copies. CAR-T copies per microgram gDNA were calculated by using the copy number of WPRE divided by the gDNA content in the sample. The gDNA content was calculated using the FOXP2 gene copies of the respective sample. The lower limit of detection was considered one copy per microgram, and 14 copies per microgram were considered the lower limit of quantification.

#### Cytokine multiplex assay

Quantification of cytokines (for example, IL-6, IFNɣ) in serum was performed using the Meso Scale Discovery (MSD) 10-plex assay using the Proinflammatory Panel 1 Multiplex Test Kit. IFNɣ inducible protein 10 was measured using the Chemokine Panel 1 Kit (MSD). Assays were performed per the manufacturer’s protocol with a seven-point standard curve generated using a fourfold dilution series in triplicate. The standards were used as described in the package insert. All samples were evaluated in duplicate at standard 1:2 dilution and maximal 1:8 dilution; calculated percentage coefficients of variation for the duplicate measures were less than 20%. Data were acquired on a Quickplex SQ120 Imager instrument (MSD) and analyzed using an MSD Discovery Workbench and a four-parameter logistic model. Reported values included those within the standard curve range and those calculated by the logistic regression analysis.

#### AFP quantification

Serum AFP levels were quantified locally using either the Siemens Atellica IM AFP Assay or the Roche Elecsys AFP immunoassay and a Cobas e801 analytical unit. Assays were performed as per the manufacturer’s protocol.

### Reporting summary

Further information on research design is available in the Nature Portfolio Reporting Summary linked to this article.

## Online content

Any methods, additional references, Nature Portfolio reporting summaries, source data, extended data, supplementary information, acknowledgements, peer review information; details of author contributions and competing interests; and statements of data and code availability are available at 10.1038/s41591-023-02612-0.

## Supplementary information

Supplementary Information Supplementary Figs. 1–4, clinical trial protocol and statistical analysis plan.

Reporting Summary

Supplementary Table 1 Previous lines of treatment for patients in the trial.

## Figures and Tables

**Extended Data Table 1:**
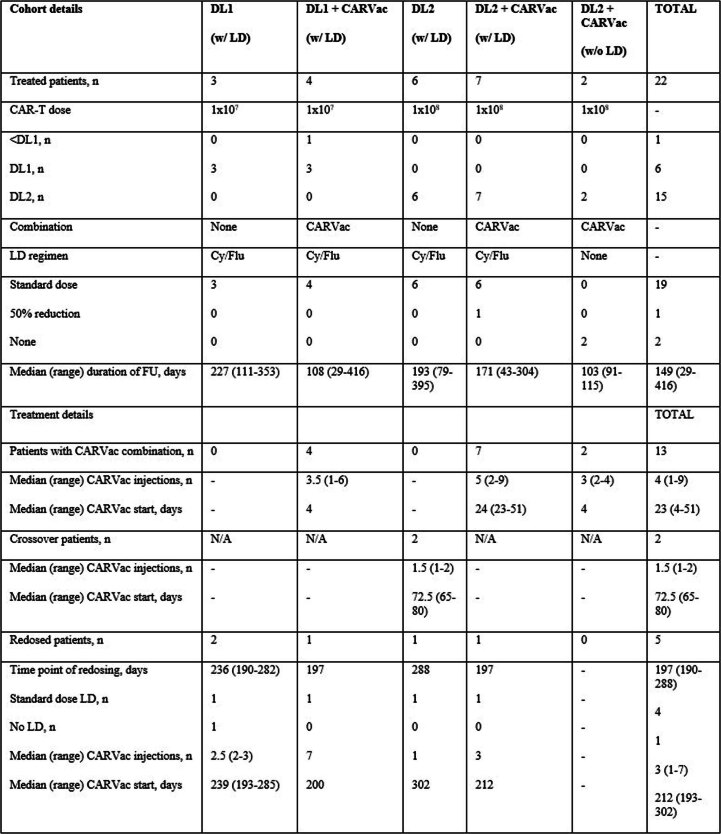
Cohort and treatment details

Cy, cyclophosphamide; DL, dose level; Flu, fludarabine; FU, follow-up; LD, lymphodepleting chemotherapy.

**Extended Data Table 2:**
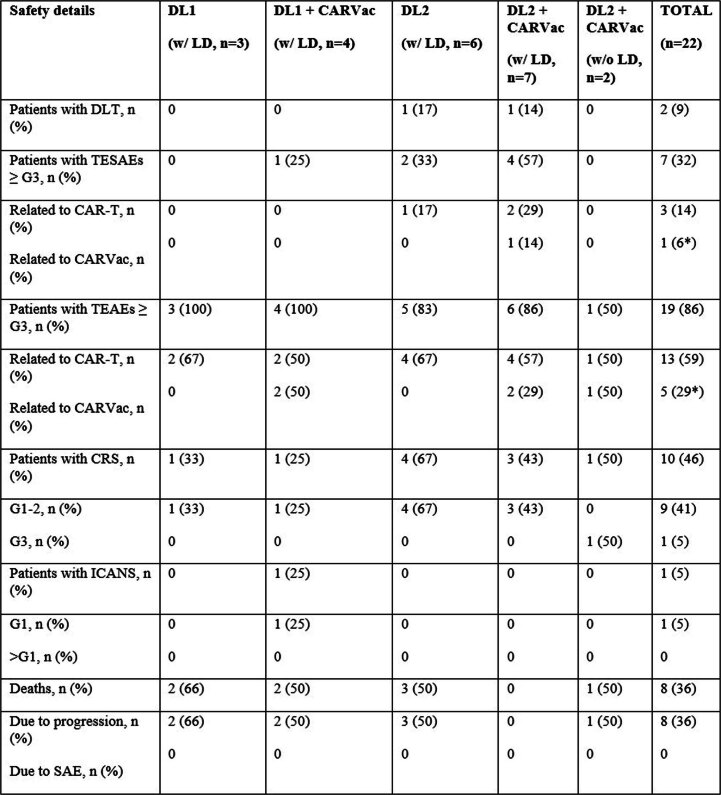
Safety summary

CRS, cytokine release syndrome; DL, dose level; DLT, dose-limiting toxicity; G, grade; ICANS, immune cell-associated neurotoxicity syndrome; LD, lymphodepleting chemotherapy; TEAE, treatment-emergent adverse event; (TE)SAE, (treatment-emergent) serious adverse event. *Percentages are calculated according to the number of patients treated with CARVac (n = 17).

**Extended Data Table 3:**
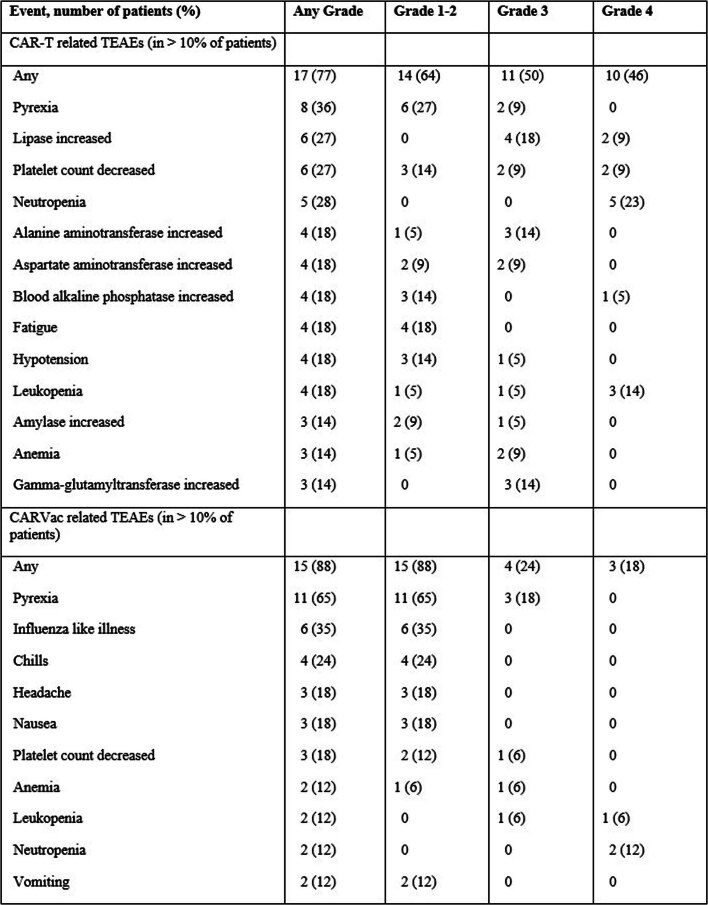
Related treatment-emergent adverse events

Percentages are calculated according to the number of patients treated with CAR-T cells (n = 22) or CAR-T cells and CARVac (n = 17).

**Extended Data Table 4:**
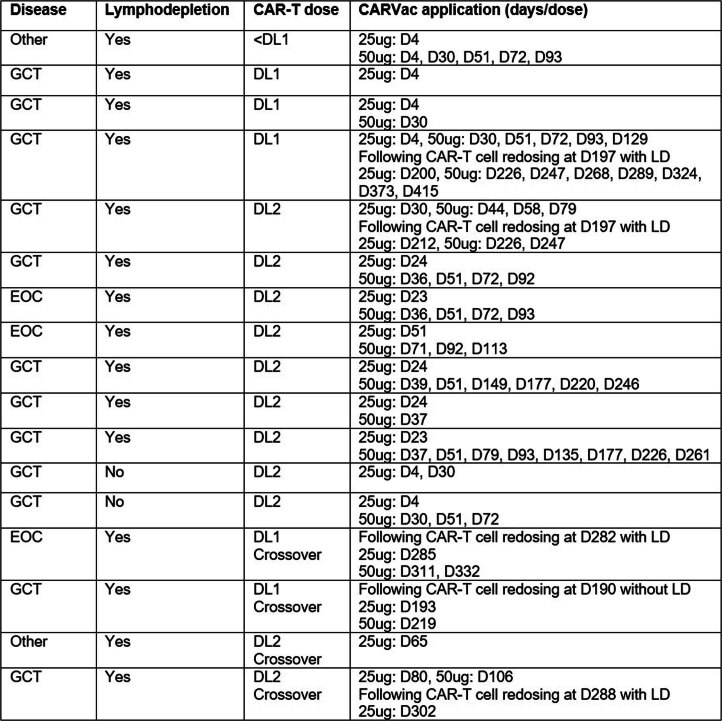
Patients that received CARVac treatment

*CARVac following redosing of CAR-T

EOC, epithelial ovarian cancer; GCT, germ cell tumor.

## Data Availability

This trial is currently ongoing. Upon completion of this clinical trial, summary-level results will be made public and shared in line with clinical data-sharing guidelines. Requests for access to aggregated data (the tables produced in line with the statistical analysis plan) will be reviewed and approved by the Safety Review Committee on the basis of scientific merit. The datasets generated and/or analyzed during the current study are not publicly available due to proprietary considerations beyond the data that were made available here. All data provided are anonymized to respect the privacy of patients who have participated in the trial, in line with applicable laws and regulations. Data requests pertaining to the manuscript may be made to the corresponding author (U.Ş.; ugur.sahin@biontech.de). Requests will be processed within 16 weeks. The CAR-T (WO2020/161186) and CARVac (W2016/180778) technology is patented.
