# Supplementary material for: CLDN6-specific CAR-T cells plus amplifying RNA vaccine in relapsed or refractory solid tumors: the phase 1 BNT211-01 trial
Source: Nat Med. 2023 Oct 23;29(11):2844–53. doi: 10.1038/s41591-023-02612-0 (PMC10667102; doi:10.1038/s41591-023-02612-0)
Supplement: Supplementary file 1 — Supplementary Figs. 1–4, clinical trial protocol and statistical analysis plan. [file 41591_2023_2612_MOESM1_ESM.pdf]

nature medicine

Article

https://doi.org/10.1038/s41591-023-02612-0

CLDN6-specific CAR-T cells plus amplifying RNA vaccine in relapsed or refractory solid tumors: the phase 1 BNT211-01 trial

In the format provided by the authors and unedited

SUPPLEMENTARY INFORMATION

Supplementary Figure 1

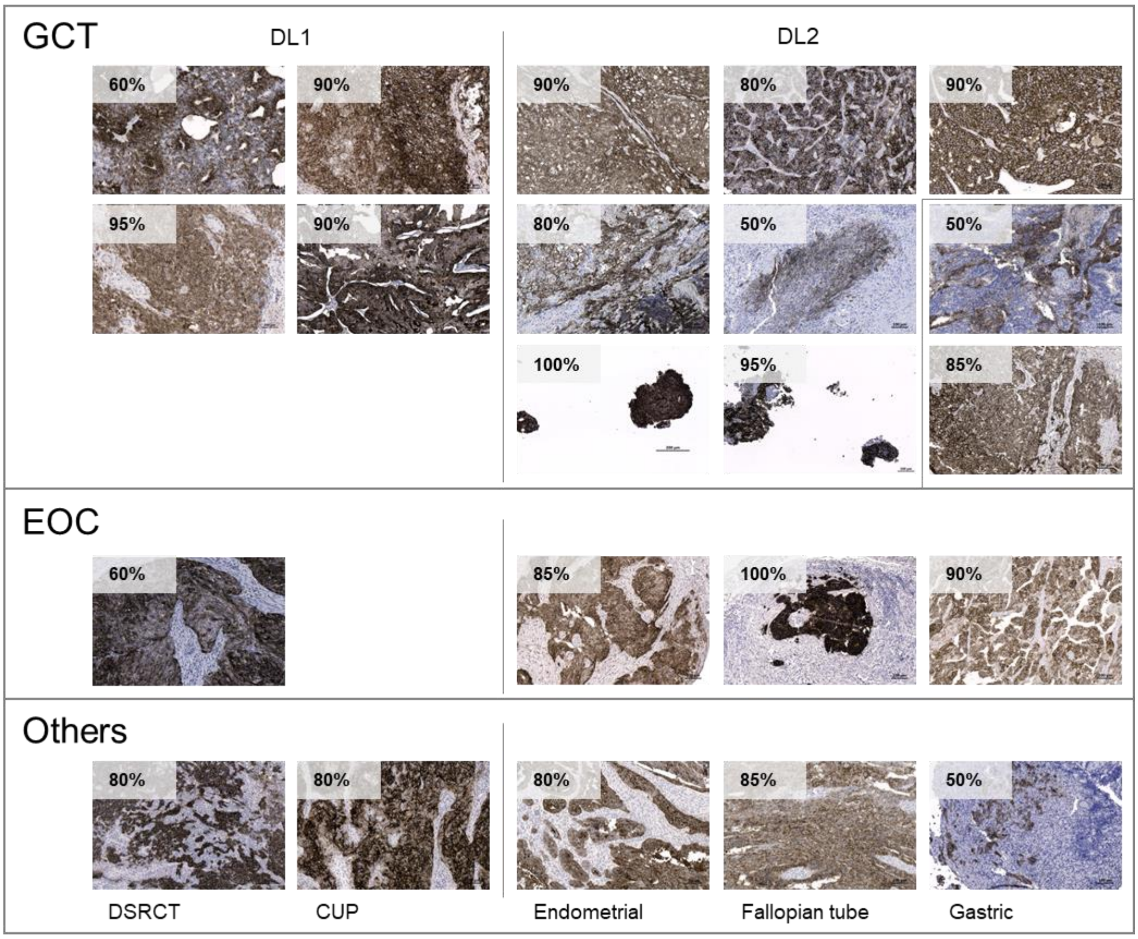

Supplementary Figure 1. CLDN6 pre-screening results of patients recruited to the trial.

Frequency of 2+/3+ CLDN6 expression revealed by immunohistochemical analysis of tumor tissue from patients treated with CLDN6 CAR-T cells grouped by indication and dose level. Cancers of "other" origin than GCT and EOC as indicated. Two patients at DL2 treated w/o LD are grouped.

Percentages indicate the number of tumor cells positive for intermediate (2+) or strong (3+) membrane expression of CLDN6. Immunohistochemical analysis was performed on two tumor samples per patient, with the stronger of the two values used to determine eligibility.

CLDN6, claudin 6; CUP, cancer of unknown primary; DSRCT, desmoplastic small round cell tumor; EOC, epithelial ovarian cancer; GCT, germ cell tumor; LD, lymphodepleting chemotherapy.

Supplementary Figure 2

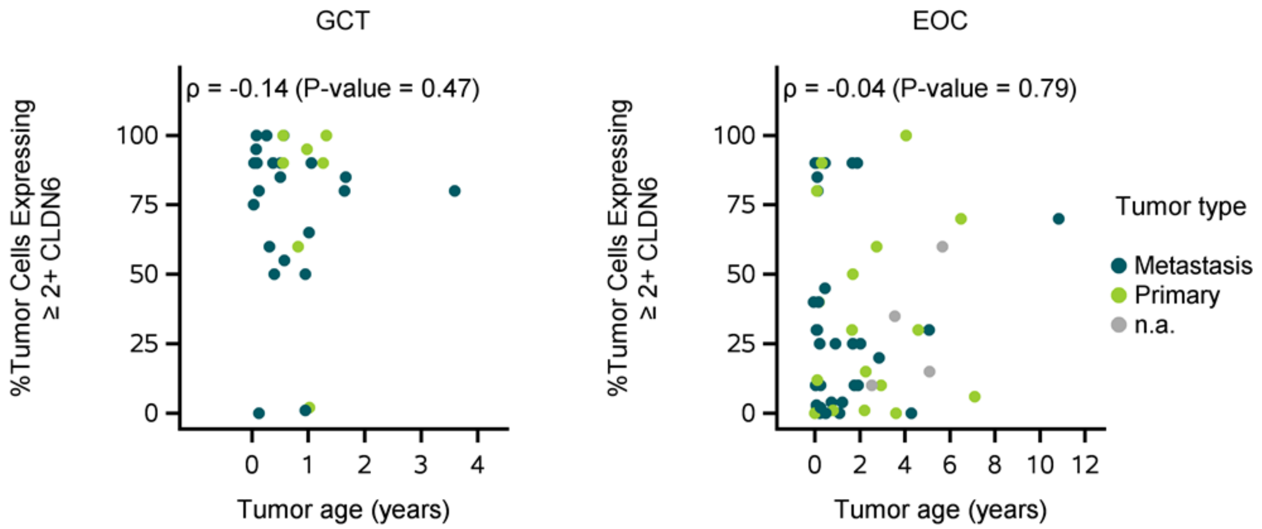

Supplementary Figure 2: The intensity of CLDN6 expression by IHC staining in tumor samples does not correlate with the age of the analyzed tumor sample for both GCT and EOC patients.

Spearman rank correlation analysis of the age of pre-screening tumor samples and frequency of 2+/3+ CLDN6 expression in tumor cells revealed by immunohistochemical analysis from GCT and EOC patients. Tumor age refers to the time period between tissue collection and IHC analysis. Each dot represents a tumor sample: GCT patients, n=30; EOC patients, n=53. 2 patients with tumor collection dates not available were excluded from the analysis. Tumor age and CLDN6 expression were analyzed with Spearman's rank correlation coefficient.
n.a., information on tumor type not available; IHC, immunohistochemistry.

Supplementary Figure 3

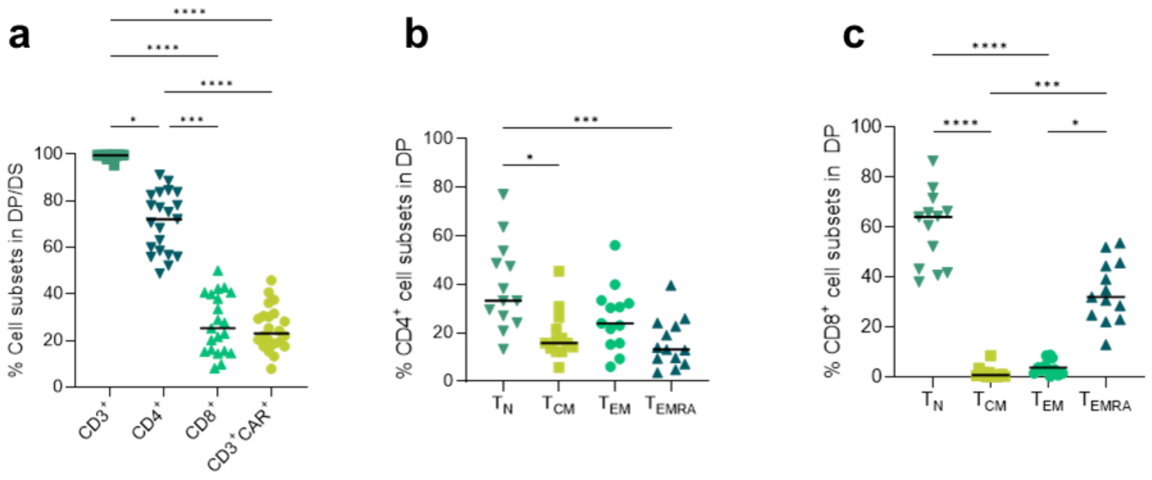

Supplementary Figure 3: Characterization of the drug products by flow cytometry (CD4/CD8 distribution, CAR expression, and memory phenotype).

a, $CD3^+$, $CD4^+$, and $CD8^+$ cells were analyzed from bulk DP (n=21), $CD3^+CAR^+$ was analyzed from DS (n=22). **b**, **c**, TN, TCM, TEM, and TEMRA were analyzed in $CD4^+$ and $CD8^+$ T cells from bulk DP (n=13), respectively, based on sample availabilities. Horizontal line represents median frequency. Kruskal-Wallis test with Dunn's multiple comparison post hoc test was applied. * p≤0.05; **p≤0.01; ***p≤0.001; **** p≤0.0001, with the exact adjusted P values as follows:

For %cell subsets in DP/DS:

**Dunn's multiple comparisons test**	**Summary**	**Adjusted P Value**
$CD3^+CAR^+$ vs. $CD3^+$	****	<0.0001
$CD3^+CAR^+$ vs. $CD4^+$	****	<0.0001
$CD3^+$ vs. $CD4^+$	*	0.0343
$CD3^+$ vs. $CD8^+$	****	<0.0001
$CD4^+$ vs. $CD8^+$	***	0.0002

For %CD4+ cell subsets in DP:

**Dunn's multiple comparisons test**	**Summary**	**Adjusted P Value**
T_{CM} vs. T_N	*	0.0157
T_N vs. T_{EMRA}	***	0.0010

For %CD8+ cell subsets in DP:

**Dunn's multiple comparisons test**	**Summary**	**Adjusted P Value**
T_{CM} vs. T_N	****	<0.0001
T_{CM} vs. T_{EMRA}	***	0.0002
T_N vs. T_{EM}	****	<0.0001
T_{EMRA} vs. T_{EM}	*	0.0264

CAR, Chimeric antigen receptor; DP, Drug Product; DS, Drug Substance; TN, naïve-like T cells; TCM, central memory T cells; TEM, effector memory T cells; TEMRA: effector memory re-expressing CD45RA T cells.

Supplementary Figure 4

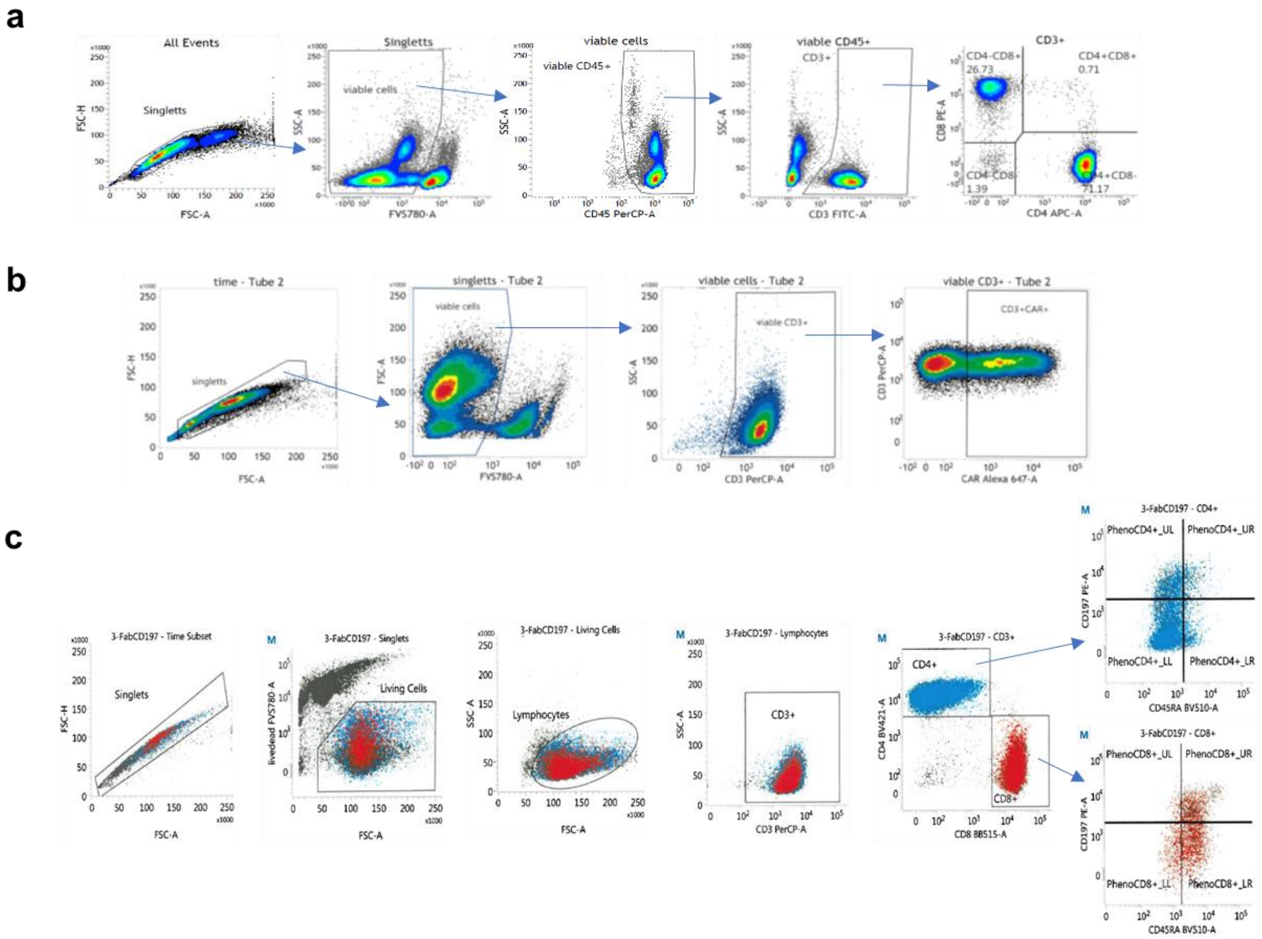

Supplementary Figure 4: Gating strategies for immunophenotyping analysis.

a, Gating strategies to determine the percentage of $CD3^+$, $CD3^+CD4^+CD8^-$ cells, and CD3+CD4-CD8+ cells in bulk DP. **b**, Gating strategies to determine the percentage of $CD3^+CAR^+$ cells in DS. **c**, Gating strategies to determine the percentage of $CD45RA^+/CCR7^+$ (TN) cells, $CD45RA^-/CCR7^+$ (TCM) cells, $CD45RA^-/CCR7^-$ (TEM) cells, and $CD45RA^+/CCR7^-$ (TEMRA) cells in the $CD4^+$ and $CD8^+$ cell subsets in bulk DP.

DP, drug product; DS, drug substance; TN, naïve-like T cells; TCM, central memory T cells; TEM, effector memory T cells; TEMRA: effector memory re-expressing CD45RA T cells.

SUPPLEMENTARY INFORMATION

Supplementary Figure 1

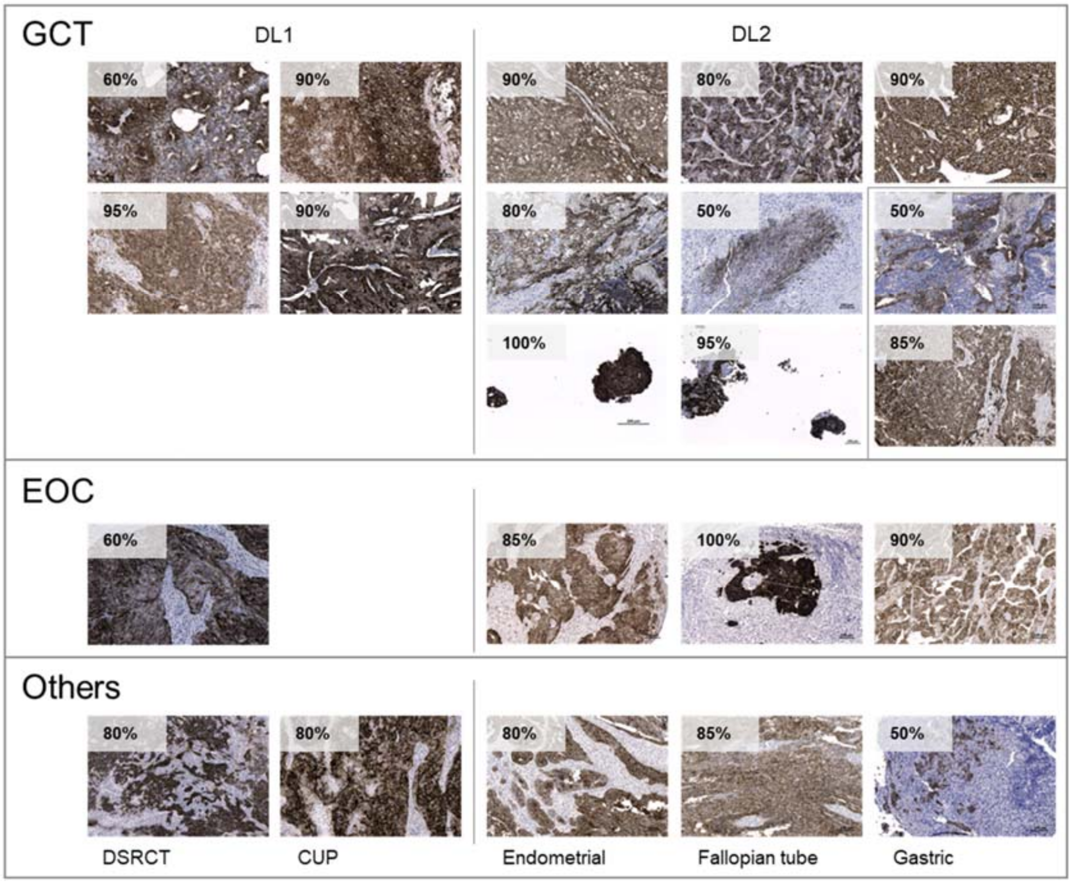

Supplementary Figure 1. CLDN6 pre-screening results of patients recruited to the trial.

Frequency of 2+/3+ CLDN6 expression revealed by immunohistochemical analysis of tumor tissue from patients treated with CLDN6 CAR-T cells grouped by indication and dose level. Cancers of "other" origin than GCT and EOC as indicated. Two patients at DL2 treated w/o LD are grouped.

Percentages indicate the number of tumor cells positive for intermediate (2+) or strong (3+) membrane expression of CLDN6. Immunohistochemical analysis was performed on two tumor samples per patient, with the stronger of the two values used to determine eligibility.

CLDN6, claudin 6; CUP, cancer of unknown primary; DSRCT, desmoplastic small round cell tumor; EOC, epithelial ovarian cancer; GCT, germ cell tumor; LD, lymphodepleting chemotherapy.

Supplementary Figure 2

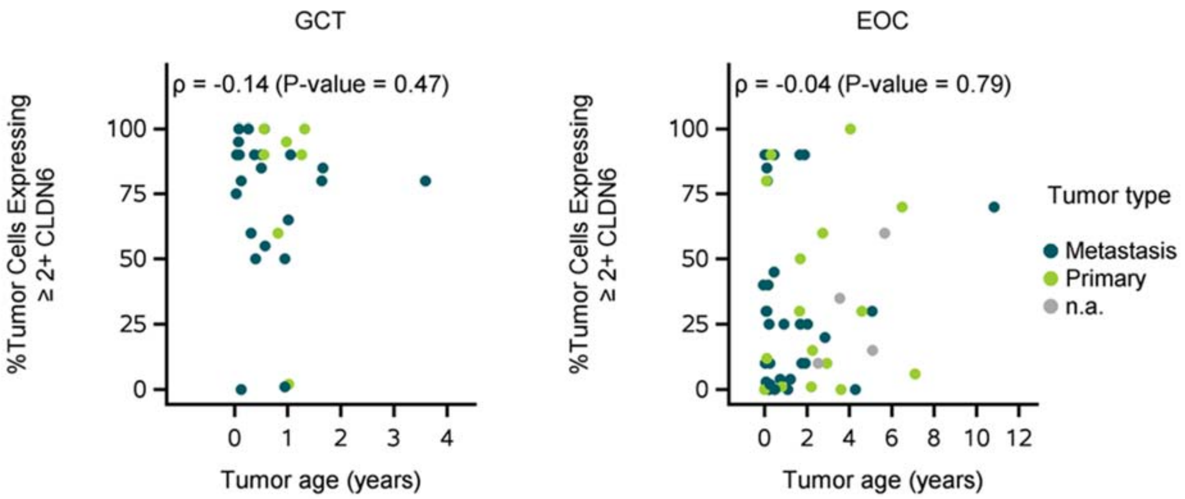

Supplementary Figure 2: The intensity of CLDN6 expression by IHC staining in tumor samples does not correlate with the age of the analyzed tumor sample for both GCT and EOC patients.

Spearman rank correlation analysis of the age of pre-screening tumor samples and frequency of 2+/3+ CLDN6 expression in tumor cells revealed by immunohistochemical analysis from GCT and EOC patients. Tumor age refers to the time period between tissue collection and IHC analysis. Each dot represents a tumor sample: GCT patients, n=30; EOC patients, n=53. 2 patients with tumor collection dates not available were excluded from the analysis. Tumor age and CLDN6 expression were analyzed with Spearman's rank correlation coefficient.

n.a., information on tumor type not available; IHC, immunohistochemistry.

Supplementary Figure 3

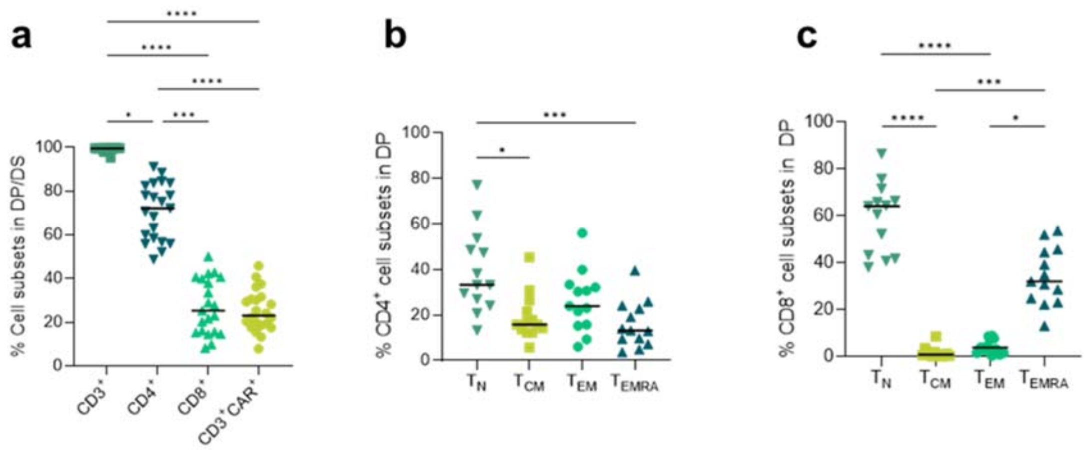

Supplementary Figure 3: Characterization of the drug products by flow cytometry (CD4/CD8 distribution, CAR expression, and memory phenotype).

a, $CD3^+$, $CD4^+$, and $CD8^+$ cells were analyzed from bulk DP (n=21), $CD3^+CAR^+$ was analyzed from DS (n=22). **b**, **c**, TN, TCM, TEM, and TEMRA were analyzed in $CD4^+$ and $CD8^+$ T cells from bulk DP (n=13), respectively, based on sample availabilities. Horizontal line represents median frequency. Kruskal-Wallis test with Dunn's multiple comparison post hoc test was applied. * $p \leq 0.05$; ** $p \leq 0.01$; *** $p \leq 0.001$; **** $p \leq 0.0001$, with the exact adjusted P values as follows:

For %cell subsets in DP/DS:

**Dunn's multiple comparisons test**	**Summary**	**Adjusted P Value**
$CD3^+CAR^+$ vs. $CD3^+$	****	<0.0001
$CD3^+CAR^+$ vs. $CD4^+$	****	<0.0001
$CD3^+$ vs. $CD4^+$	*	0.0343
$CD3^+$ vs. $CD8^+$	****	<0.0001
$CD4^+$ vs. $CD8^+$	***	0.0002

For %CD4+ cell subsets in DP:

**Dunn's multiple comparisons test**	**Summary**	**Adjusted P Value**
T_{CM} vs. T_N	*	0.0157
T_N vs. T_{EMRA}	***	0.0010

For %CD8+ cell subsets in DP:

**Dunn's multiple comparisons test**	**Summary**	**Adjusted P Value**
T_{CM} vs. T_N	****	<0.0001
T_{CM} vs. T_{EMRA}	***	0.0002
T_N vs. T_{EM}	****	<0.0001
T_{EMRA} vs. T_{EM}	*	0.0264

CAR, Chimeric antigen receptor; DP, Drug Product; DS, Drug Substance; TN, naïve-like T cells; TCM, central memory T cells; TEM, effector memory T cells; TEMRA: effector memory re-expressing CD45RA T cells.

Supplementary Figure 4

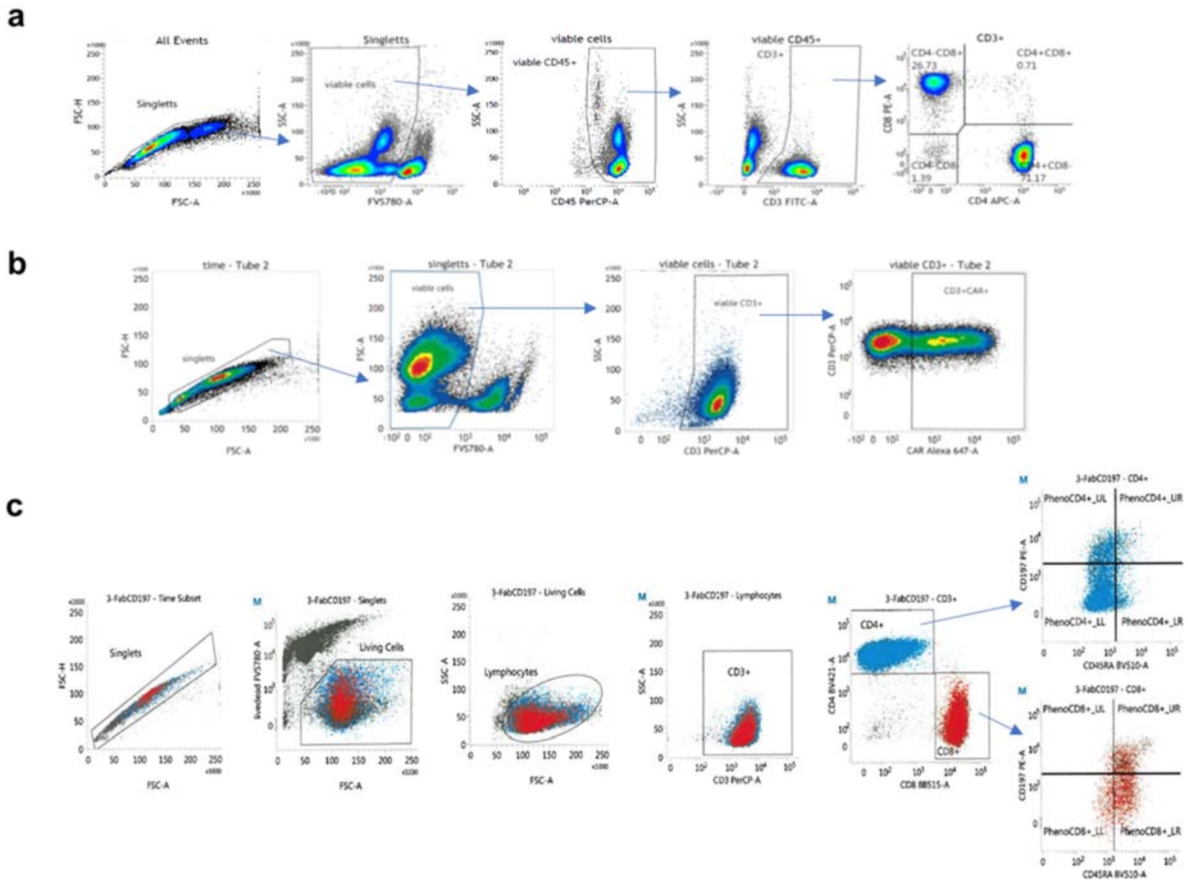

Supplementary Figure 4: Gating strategies for immunophenotyping analysis.

a, Gating strategies to determine the percentage of $CD3^+$, $CD3^+CD4^+CD8^-$ cells, and CD3+CD4-CD8+ cells in bulk DP. **b**, Gating strategies to determine the percentage of $CD3^+CAR^+$ cells in DS. **c**, Gating strategies to determine the percentage of $CD45RA^+/CCR7^+$ (TN) cells, $CD45RA^-/CCR7^+$ (TCM) cells, $CD45RA^-/CCR7^-$ (TEM) cells, and $CD45RA^+/CCR7^-$ (TEMRA) cells in the $CD4^+$ and $CD8^+$ cell subsets in bulk DP.

DP, drug product; DS, drug substance; TN, naïve-like T cells; TCM, central memory T cells; TEM, effector memory T cells; TEMRA: effector memory re-expressing CD45RA T cells.

CLINICAL TRIAL PROTOCOL

Phase I/IIa, first-in-human, open-label, dose escalation trial with expansion cohorts to evaluate safety and preliminary efficacy of CLDN6 CAR-T with or without CLDN6 RNA-LPX in patients with CLDN6-positive relapsed or refractory advanced solid tumors

Name of IMPs:	IMP-1: CLDN6 CAR-T (CG001.2)<br>IMP-1(A): CLDN6 CAR-T(A) (CG001.2(A))<br>IMP-2: CLDN6 RNA-LPX (RBL005.4)
Protocol number:	BNT211-01
Protocol version:	Version 5.0
Amendment number:	5
Indication studied:	CLDN6-positive relapsed or refractory advanced solid tumors
Trial design:	Open-label, dose escalation
Trial phase:	Phase I/IIa
Sponsor:	BioNTech Cell & Gene Therapies GmbH,<br>An der Goldgrube 12, 55131 Mainz,<br>Germany (Phone: +49 (0)6131 9084 – 0; Telefax: +49 (0)6131 9084 – 392010)
Sponsor's responsible person:	Dr. med. Özlem Türeci
Sponsor's contact person:	Dr. Liane Monika Preußner
Clinical research organization:	ICON Plc,<br>South County Business Park,<br>Leopardstown, Dublin 18,<br>d18 X5R3, Ireland
Coordinating investigator:	Prof. Dr. med. Andreas Mackensen<br>Direktor Medizinische Klinik 5- Hämatologie und Internistische Onkologie<br>Universitätsklinikum Erlangen<br>Ulmenweg 18, 91054 Erlangen<br>Phone: +49 (0)9131 85-[redacted]<br>Telefax: +49 (0)9131 85-[redacted]<br>Email: [redacted]
Regulatory agency number:	EudraCT number: 2019-004323-20
Approval date:	23 NOV 2021

Document history	Date	Version number	Valid for
First submitted version	29-NOV-2019	1.0	All countries
New version	13-MAY-2020	2.0	All countries
New version	19-FEB-2021	3.0	All countries
New version	03-MAY-2021	4.0	All countries
New version	23-NOV-2021	5.0	All countries

STATEMENT OF COMPLIANCE
Good Clinical Practice (GCP) Compliance:
This trial will be conducted in compliance with International Conference on Harmonisation (ICH) GCP E6 R2, and applicable regulatory requirements.

CONFIDENTIALITY STATEMENT
The information contained in this document is the property and copyright of BioNTech Cell & Gene Therapies GmbH. Therefore, this document is provided in confidence to the recipient (e.g., regulatory authorities, Independent Ethics Committees [IECs]/Institutional Review Board [IRBs], investigators, auditors, inspectors). No information contained herein shall be published, disclosed, or reproduced without prior written approval of the proprietor(s).

TABLE OF CONTENTS

1 PROTOCOL SUMMARY ... 12
1.1 SYNOPSIS ... 12
1.2 SCHEMA ... 25
1.3 SCHEDULES OF ACTIVITIES ... 30
1.3.1 Part 1 SoA (CLDN6 CAR-T/CLDN6 CAR-T(A)) ... 30
1.3.2 Part 1 cross-over SoA (CLDN6 CAR-T/CLDN6 CAR-T(A) + CLDN6 RNA-LPX) ... 37
1.3.3 Standard Part 2 SoA (CLDN6 CAR-T/CLDN6 CAR-T(A) + CLDN6 RNA-LPX)41
1.3.4 Optional Part 2 SoA (CLDN6 CAR-T/CLDN6 CAR-T(A) + CLDN6 RNA-LPX)49
1.3.5 Optional Part 2 SoA (CLDN6 CAR-T/CLDN6 CAR-T(A) + CLDN6 RNA-LPX without LD chemotherapy) ... 57
2 INTRODUCTION ... 64
2.1 CLDN6 as target for CAR-T therapy ... 66
2.1.1 CLDN6 expression in healthy tissues ... 67
2.1.2 CLDN6 expression in cancer ... 69
2.2 BACKGROUND ... 70
2.2.1 Overview of the diseases ... 70
2.2.2 Introduction to the investigational treatment ... 77
2.3 TRIAL RATIONALE ... 79
2.3.1 Rationale for the trial ... 79
2.3.2 Rationale for administration of CLDN6 CAR-T/CLDN6 CAR-T(A) and CLDN6 RNA-LPX ... 80
2.3.3 Rationale for lymphodepleting chemotherapy choice and dose ... 81
2.4 BENEFIT/RISK ASSESSMENT ... 83
2.4.1 Risk assessment ... 83
2.4.2 Benefit assessment ... 87
2.4.3 Overall benefit over risk conclusion ... 88
3 OBJECTIVES AND ENDPOINTS ... 90
4 TRIAL DESIGN ... 93
4.1 OVERALL DESIGN ... 93
4.1.1 Dose escalation ... 95
4.1.2 Dose-limiting toxicity ... 98
4.1.3 Expansion (Part 3) ... 101
4.2 PLANNED NUMBER OF PATIENTS ... 101
4.2.1 Replacement of patients ... 101
4.3 TRIAL DESIGN RATIONALE ... 101
4.3.1 Rationale for bifurcated design ... 102
4.4 DOSE AND SCHEDULE RATIONALE ... 103
4.4.1 CAR-T dose rationale ... 103

4.4.2 CLDN6 RNA-LPX dose rationale 107
4.5 END OF TRIAL DEFINITION 107
5 TRIAL POPULATION 107
5.1 INCLUSION CRITERIA 108
5.2 EXCLUSION CRITERIA 111
5.3 PRE-SCREENING 113
5.4 SCREEN FAILURES 113
6 TRIAL TREATMENTS 114
6.1 TRIAL TREATMENTS ADMINISTERED 114
6.1.1 Lymphodepleting chemotherapy regimen 114
6.1.2 IMP-1 and IMP-1(A): CLDN6 CAR-T/CLDN6 CAR-T(A) 115
6.1.3 IMP-2: CLDN6 RNA-LPX 118
6.2 PREPARATION/HANDLING/STORAGE/ACCOUNTABILITY 120
6.3 MEASURES TO MINIMIZE BIAS: RANDOMIZATION AND BLINDING . 121
6.4 TRIAL TREATMENT COMPLIANCE 121
6.5 CONCOMITANT THERAPY 121
6.5.1 Prohibited medication 121
6.5.2 Permitted medication 122
6.5.3 Rescue medication and further supportive measures 122
6.6 DOSE MODIFICATIONS 123
6.6.1 Dose modification for CLDN6 CAR-T/CLDN6 CAR-T(A) 123
6.6.2 Dose modification for CLDN6 RNA-LPX 123
6.6.3 Mitigation plans for specific adverse events 125
6.6.4 Clinical observation of patients receiving IMP-1, IMP-1(A), or IMP-2 125
6.6.5 Safety stopping criteria 132
6.7 TREATMENT AFTER THE END OF TRIAL 133
7 DISCONTINUATION OF TRIAL TREATMENT AND PATIENT DISCONTINUATION/WITHDRAWAL 133
7.1 DISCONTINUATION OF TRIAL TREATMENT 133
7.2 PATIENT EARLY DISCONTINUATION/WITHDRAWAL FROM THE TRIAL 134
7.3 LOST TO FOLLOW-UP 135
8 TRIAL ASSESSMENTS AND PROCEDURES 135
8.1 EFFICACY ASSESSMENTS 136
8.1.1 Tumor response 136
8.1.2 Performance status 136
8.2 SAFETY ASSESSMENTS 136
8.2.1 Physical examinations 136
8.2.2 Vital signs 137
8.2.3 Electrocardiograms 137
8.2.4 Clinical safety laboratory assessments 137

8.3 ADVERSE EVENTS AND SERIOUS ADVERSE EVENTS 138
8.3.1 Time period and frequency for collecting AE and SAE information 138
8.3.2 Method of detecting AEs and SAEs 139
8.3.3 Follow-up of AEs and SAEs 139
8.3.4 Regulatory reporting requirements for SAEs 139
8.3.5 Pregnancy 140
8.3.6 Disease-related events and/or disease-related outcomes not qualifying as AEs or SAEs 140
8.4 TREATMENT OF OVERDOSE 140
8.5 PHARMACOKINETICS 141
8.6 PHARMACODYNAMICS 141
8.7 GENETICS 141
8.8 BIOMARKERS 141
8.8.1 CLDN6 expression at pre-screening 142
8.8.2 Pharmacodynamic biomarkers 142
8.8.3 Exploratory assessments of blood biomarkers 142
8.8.4 Exploratory assessment of tumor tissue 142
8.8.5 Biomarker sample collection 143
8.8.6 Other biomarkers 144
8.9 IMMUNOGENICITY ASSESSMENTS 144
8.10 HEALTH ECONOMICS OR MEDICAL RESOURCE UTILIZATION AND HEALTH ECONOMICS 144
9 STATISTICAL CONSIDERATIONS 144
9.1 STATISTICAL HYPOTHESES 144
9.2 SAMPLE SIZE DETERMINATION 144
9.3 ANALYSIS SETS 144
9.4 STATISTICAL ANALYSES 145
9.4.1 General considerations 146
9.4.2 Primary endpoints 146
9.4.3 Secondary endpoints 147
9.4.4 Exploratory endpoints 147
9.4.5 Safety endpoints 148
9.4.6 Other analyses 149
9.5 Interim analyses 149
9.6 Safety Review Committee 149
10 INVESTIGATORS AND STUDY ADMINISTRATIVE STRUCTURE 149
10.1 INVESTIGATORS AND TRIAL SITE PERSONNEL 149
10.1.1 Investigators 149
10.1.2 Trial site personnel assigned trial-related duties 150
10.2 CONTRACT RESEARCH ORGANIZATIONS 150
10.3 THE SPONSOR AND SPONSOR'S PERSONNEL 150

10.4 OTHER BODIES (STEERING COMMITTEES) ... 150
11 REFERENCES ... 151
12 SUPPORTING DOCUMENTATION AND OPERATIONAL CONSIDERATIONS (APPENDICES) ... 164
12.1 APPENDIX 1: REGULATORY, ETHICAL, AND TRIAL OVERSIGHT CONSIDERATIONS ... 164
12.1.1 Regulatory and ethical considerations ... 164
12.1.2 Financial disclosure ... 164
12.1.3 Informed consent process ... 164
12.1.4 Data protection ... 165
12.1.5 Committees structure ... 165
12.1.6 Data quality assurance ... 166
12.1.7 Source documents ... 166
12.1.8 Trial and site start and closure ... 167
12.1.9 Publication policy ... 167
12.1.10 Trial results reporting ... 168
12.2 APPENDIX 2: CLINICAL LABORATORY TESTS ... 169
12.3 APPENDIX 3: ADVERSE EVENTS: DEFINITIONS AND PROCEDURES FOR RECORDING, EVALUATING, FOLLOW-UP, AND REPORTING ... 171
12.3.1 Definition of AE ... 171
12.3.2 Definition of SAE and suspected unexpected serious adverse reaction (SUSAR)172
12.3.3 Recording and follow-up of any AE ... 174
12.3.4 Reporting of SAEs ... 181
12.4 APPENDIX 4: CONTRACEPTIVE GUIDANCE AND COLLECTION OF PREGNANCY INFORMATION ... 183
12.5 APPENDIX 5 RECIST 1.1 DEFINITIONS AND RESPONSE CRITERIA ... 185
12.6 APPENDIX 6 iRECIST DEFINITIONS AND RESPONSE CRITERIA ... 188
12.7 APPENDIX 7 COUNTRY-SPECIFIC REQUIREMENTS ... 191
12.8 APPENDIX 8 LONG-TERM FOLLOW-UP ... 192
12.8.1 Introduction and rationale ... 192
12.8.2 Objectives and endpoints ... 192
12.8.3 Trial design ... 193
12.8.4 Trial population ... 194
12.8.5 Trial treatments ... 194
12.8.6 Further therapies ... 194
12.8.7 Patient discontinuation and withdrawal ... 194
12.8.8 Trial assessments and procedures ... 195

LIST OF FIGURES

Figure 1: Trial design ... 25
Figure 2: Timeline of trial treatment administration in Part 1 – CLDN6 CAR-T/CLDN6 CAR-T(A) dose escalation ... 26
Figure 3: Timeline of trial treatment in Standard Part 2 CLDN6 CAR-T/CLDN6 CAR-T(A) + CLDN6 RNA-LPX (dose escalation) ... 27
Figure 4: Timeline of trial treatment in Optional Part 2 CLDN6 CAR-T/CLDN6 CAR-T(A) + CLDN6 RNA-LPX (dose escalation) ... 28
Figure 5: Timeline of trial treatment in Optional Part 2 CLDN6 CAR-T/CLDN6 CAR-T(A) + CLDN6 RNA-LPX without LD chemotherapy (dose escalation) ... 29
Figure 6: Concept of CLDN6 CAR-T/CLDN6 CAR-T(A) therapy combined with RNA-LPX-mediated *in vivo* expansion of CAR-T ... 65
Figure 7: Schematic representation of CLDN6 ... 66
Figure 8: Schematic representation of the IMP-1 and IMP-1(A) autologous CLDN6 CAR-T/CLDN6 CAR-T(A) ... 78
Figure 9: Schematic representation of the IMP-2, CLDN6-encoding liposome-formulated RNA ... 79
Figure 10: Concept of the Sponsor's CLDN6 CAR-T/CLDN6 CAR-T(A) therapy with CLDN6 RNA-LPX-mediated *in vivo* expansion of CLDN6 CAR-T/CLDN6 CAR-T(A) ... 81
Figure 11: Guidance for staggered enrollment ... 97
Figure 12: Handling of adverse events fulfilling the DLT criteria ... 123
Figure 13: Management of CAR-T-related fulminant hemophagocytic lymphohistiocytosis (macrophage activation syndrome) ... 130

LIST OF TABLES

Table 1: Part 1 SoA (CLDN6 CAR-T/CLDN6 CAR-T(A)) pre-screening, screening, apheresis, pre-treatment ... 30
Table 2: Part 1 SoA (CLDN6 CAR-T/CLDN6 CAR-T(A)) treatment, primary follow-up 34
Table 3: Part 1 cross-over SoA – treatment, primary follow-up for patients crossing over from Part 1 (CLDN6 CAR-T/CLDN6 CAR-T(A)) to additional cohort in Part 1 (CLDN6 CAR-T/CLDN6 CAR-T(A) + CLDN6 RNA-LPX) ... 37
Table 4: Standard Part 2 SoA - pre-screening, screening, apheresis, pre-treatment ... 41
Table 5: Standard Part 2 SoA - treatment, primary follow-up ... 45
Table 6: Optional Part 2 SoA - pre-screening, screening, apheresis, pre-treatment ... 49
Table 7: Optional Part 2 SoA - treatment, primary follow-up ... 53
Table 8: Optional Part 2 SoA - pre-screening, screening, apheresis, pre-treatment (without LD chemotherapy) ... 57
Table 9: Optional Part 2 SoA - treatment, primary follow-up (without LD chemotherapy)60

Table 10: Summary of CLDN6 target characteristics 66
Table 11: Summary of CLDN6 mRNA and protein expression in normal human adult tissues 68
Table 12: Summary of CLDN6 mRNA and protein expression in human cancers 69
Table 13: Risk assessment 83
Table 14: Objectives and endpoints 90
Table 15: Dose escalation 96
Table 16: Review of CAR-T trials on solid tumors 105
Table 17: American Society for Transplantation and Cellular Therapy cytokine release syndrome consensus grading 126
Table 18: Guidelines for management of cytokine release syndrome 127
Table 19: Grading and management of immune effector cell-associated neurotoxicity syndrome 128
Table 20: Vital signs 137
Table 21: Analysis sets 145
Table 22: Protocol-required safety laboratory assessments 169
Table 23: Biomarker assays to monitor CAR-T mediated effects 170
Table 24: Assessment of causality 176
Table 25: Comparison of RECIST 1.1 and iRECIST 188
Table 26: Assignment of timepoint response using iRECIST 189
Table 27: Objectives and endpoints 192
Table 28: Schedule of activities for long-term follow-up 198

LIST OF ABBREVIATIONS AND DEFINITION OF TERMS

AE	Adverse event
ALK	Anaplastic lymphoma kinase
Anti-PD-1	Antibody against Programmed cell death protein 1 (e.g., nivolumab, pembrolizumab)
AFP	Alpha-fetoprotein
APC	Antigen-presenting cell
ASTCT	American Society for Transplantation and Cellular Therapy
BRAF	Protooncogene -B-Raf
CAR	Chimeric antigen receptor
CAR-T	Chimeric antigen receptor T cell
CLDN6	Claudin 6
CR	Complete response
CRO	Contract research organization
CT	Computer tomography
CTCAE	Common terminology criteria for adverse events
d	Day
DCR	Disease control rate
DL-1	Dose level minus 1
DL-2	Dose level minus 2
DLT	Dose-limiting toxicity
DOR	Duration of response
ECG	Electrocardiogram
(e)CRF	(Electronic) case report form
EGFR	Epidermal growth factor receptor
EMA	European Medicines Agency
EMEA	European Medicines Agency (now EMA)
EoT	End of trial
EU	European Union
FDA	Food and Drug Administration
FFPE	Formalin-fixed, paraffin-embedded
FIH	First-in-human
G-CSF	Granulocyte-colony-stimulating factor
GCP	Good clinical practice
GFR	Glomerular filtration rate
GM-CSF	Granulocyte-macrophage colony-stimulating factor
HCV	Hepatitis C virus
HER2	Human epidermal growth factor receptor 2
HRT	Hormone replacement therapy
IB	Investigator’s brochure

ICANS	Immune effector cell-associated neurotoxicity syndrome
ICE	Immune effector cell-associated encephalopathy
ICF	Informed consent form
ICH	International Council for Harmonisation of Technical Requirements for Pharmaceuticals for Human Use
iCPD	Disease progression per iRECIST
iCR	Complete response per iRECIST
ICU	Intensive care unit
iDCR	Disease control rate per iRECIST
IEC	Independent Ethics Committee
IHC	Immunohistochemistry
IL	Interleukin
IMP	Investigational medicinal product
iORR	Objective response rate per iRECIST
iPR	Partial response per iRECIST
IRB	Institutional Review Board
iRECIST	Immune response evaluation criteria in solid tumors
IRR	Infusion-related reaction
iSD	Stable disease per iRECIST
iUPD	Unconfirmed progressive disease per iRECIST
i.v.	Intravenous
LD	Lymphodepletion
LTFU	Long-term follow-up
m	Month
MAS	Macrophage activation syndrome
MedDRA	Medical Dictionary for Regulatory Activities
MMR	Mismatch repair
MRI	Magnetic resonance imaging
mRNA	Messenger RNA
MSI	Microsatellite instability
MTD	Maximum tolerated dose
n	Number of patients
NCCN	National Comprehensive Cancer Network
NCI	National Cancer Institute
NOS	Not otherwise specified
NSAID	Non-steroidal anti-inflammatory drug
NSCLC	Non-small cell lung cancer
ORR	Objective response rate
OS	Overall survival
PARP	Poly adenosine diphosphate ribose polymerase
PD	Progressive disease

PFS	Progression-free survival
PK	Pharmacokinetics
PR	Partial response
PT	Preferred term
qPCR	Quantitative polymerase chain reaction
qRT-PCR	Quantitative reverse transcription polymerase chain reaction
RCR	Replication-competent retroviruses
RECIST	Response evaluation criteria in solid tumors
RECIST 1.1	Response evaluation criteria in solid tumors version 1.1
RNA	Ribonucleic acid
RNA-LPX	Vaccine formulated encoding ribonucleic acid lipoplexes
ROS-1	Receptor tyrosine kinase encoded by gene ROS-1
RP2D	Recommended Phase 2 dose
RSI	Reference safety information
RT-PCR	Reverse transcription polymerase chain reaction
SAE	Serious adverse event
SAP	Statistical analysis plan
scFv	Single chain variable fragment
SCr	Serum creatinine
SD	Stable disease
SDF-1	Stromal cell-derived factor 1
SOC	System organ class
SoA	Schedule of activities
SRC	Safety Review Committee
TCR	T cell receptor
TD	Tolerated dose
TEAE	Treatment-emergent adverse event
TLS	Tumor lysis syndrome
T_{max}	Time to maximum concentration
TNF	Tumor necrosis factor
ULN	Upper limit of normal
US	United States (of America)
WBC	White blood cell
wk	Week
WOCBP	Women of childbearing potential

1 PROTOCOL SUMMARY

1.1 SYNOPSIS

**Title**<br>Phase I/IIa, first-in-human (FIH), open-label, dose escalation trial with expansion cohorts to evaluate safety and preliminary efficacy of CLDN6 CAR-T with or without CLDN6 RNA-LPX in patients with CLDN6-positive relapsed or refractory advanced solid tumors
**Brief title**<br>A Phase I/IIa trial to evaluate the safety and efficacy of CLDN6 CAR-T +/- CLDN6 RNA-LPX
**Clinical phase**<br>Phase I/IIa
**Purpose and rationale**<br>The main purpose of the trial is to determine a safe and potentially efficacious dose of claudin 6 (CLDN6) chimeric antigen receptor T cells (CAR-T) +/- CLDN6-encoding ribonucleic acid lipoplexes (RNA-LPX). This will be achieved by running a Phase I dose escalation with CLDN6 CAR-T (Part 1) and CLDN6 CAR-T with CLDN6 RNA-LPX (Part 2) according to the classical 3+3 design. The trial will use CLDN6 CAR-T manufactured using a manual process first (CLDN6 CAR-T (CG001.2)) followed by a dose escalation with CLDN6 CAR-T manufactured using an automated process (CLDN6 CAR-T(A) (CG001.2(A)). The recommended Phase 2 dose (RP2D) of the CLDN6 CAR-T/CLDN6 CAR-T(A) +/- CLDN6 RNA-LPX will then be tested further in Phase 2a expansion cohorts (Part 3) to seek a potential preliminary efficacy signal. Establishing a safe and efficacious dose is crucial for the successful development of new anti-cancer therapies, which will ultimately benefit patients. As far as the sponsor is aware, no drug has been approved that targets CLDN6 for the treatment of cancer patients. The target-specific autologous cellular therapy offered by our CLDN6 CAR-T/CLDN6 CAR-T(A) +/- CLDN6 RNA-LPX could potentially confer meaningful clinical benefit in the absence of on-target off-tumor toxicity.<br><br>Outcome remains poor for patients with relapsed or refractory advanced solid tumors. Treatment options include further palliative chemotherapy or best supportive care. Therapy in this population is not curative with an expected overall survival (OS) of a few months. The oncofetal antigen, CLDN6, has emerged as an attractive therapeutic target because it is absent from toxicity-relevant adult healthy tissues but is highly expressed in cancers with high unmet medical need. Thus, CLDN6 represents an ideal target antigen for the development of a CAR-T therapy to treat these malignancies. Furthermore, the combination of CLDN6 CAR-

T/CLDN6 CAR-T(A) with CLDN6 RNA-LPX mediates controlled *in vivo* expansion and improved CAR-T persistence and anti-tumor efficacy in pre-clinical models. Hence, combining CLDN6 CAR-T/CLDN6 CAR-T(A) with CLDN6 RNA-LPX constitutes an innovative treatment concept for the specific and effective treatment of CLDN6-expressing tumors.

Objectives and endpoints

**Objectives**	**Endpoints**
**Primary**	
To assess the safety and tolerability of CLDN6 CAR-T/CLDN6 CAR-T(A) +/- CLDN6 RNA-LPX and to assess the comparability of CLDN6 CAR-T and CLDN6 CAR-T(A)	• Occurrence of treatment-emergent adverse events (TEAEs) within a patient including ≥ Grade 3, serious, fatal TEAEs by relationship<br>• Occurrence of dose reduction and discontinuation of investigational medicinal product (IMP) within a patient due to TEAEs
To identify the maximum tolerated dose (MTD)/RP2D for each IMP (i.e. CLDN6 CAR-T/CLDN6 CAR-T(A) +/- CLDN6 RNA-LPX) based on the occurrence of dose-limiting toxicities (DLT) using the following definitions:<br>• MTD is defined as the highest tolerated dose of CLDN6 CAR-T/CLDN6 CAR-T(A) +/- CLDN6 RNA-LPX where less than 33% of the patients experience a DLT<br>• RP2D of CLDN6 CAR-T/CLDN6 CAR-T(A) +/- CLDN6 RNA-LPX based on integrated evaluation of safety and other data for all dose levels tested	• Occurrence of DLTs within a patient during the DLT evaluation period
**Secondary**	
To describe the profile of soluble immune factors in CLDN6 CAR-T/CLDN6 CAR-T(A) +/- CLDN6 RNA-LPX	• Change from baseline in the levels and kinetics of soluble immune factors measured by cytokine multiplex assay

To evaluate anti-tumor activity of CLDN6 CAR-T/CLDN6 CAR-T(A) +/- CLDN6 RNA-LPX according to response evaluation criteria in solid tumors version 1.1 (RECIST 1.1)	• Objective response rate (ORR) defined as the proportion of patients in whom a complete response (CR) or partial response (PR) (per RECIST 1.1) is observed as best overall response<br>• Disease control rate (DCR) defined as the proportion of patients in whom a CR or PR or stable disease (SD) (per RECIST 1.1, SD assessed at least 6 weeks after the first dose) is observed as best overall response<br>• Duration of response (DOR) defined as the time from first objective response (CR or PR per RECIST 1.1) to first occurrence of objective tumor progression (PD per RECIST 1.1)/recurrence, or death from any cause, whichever occurs first
**Exploratory**	
To evaluate anti-tumor activity of CLDN6 CAR-T/CLDN6 CAR-T(A) +/- CLDN6 RNA-LPX according to immune response evaluation criteria in solid tumors (iRECIST)	• Objective response rate per iRECIST (iORR) defined as the proportion of patients in whom a complete response or partial response per iRECIST (iCR or iPR) is observed as best overall response<br>• Disease control rate (iDCR per iRECIST) defined as the proportion of patients in whom an iCR or iPR or stable disease (iSD) per iRECIST (iSD assessed at least 6 weeks after the first dose), is observed as best overall response

	• iDOR defined as the time from first objective response (iCR or iPR per iRECIST) to first occurrence of objective tumor progression (iCPD per iRECIST) or death from any cause, whichever occurs first
To evaluate efficacy of CLDN6 CAR-T/CLDN6 CAR-T(A) +/- CLDN6 RNA-LPX	• Progression-free survival (PFS) defined as the time from first dose of CLDN6 CAR-T/CLDN6 CAR-T(A) to first objective PD per RECIST 1.1, or death from any cause, whichever occurs first<br>• PFS defined as the time from first dose of CLDN6 CAR-T/CLDN6 CAR-T(A) to first objective iCPD, or death from any cause, whichever occurs first<br>• OS defined as the time from first dose of CLDN6 CAR-T/CLDN6 CAR-T(A) to death from any cause<br>• Time to treatment failure (TTF) in patients treated with CLDN6 RNA-LPX, defined as time from first injection to discontinuation of treatment for any reason including disease progression, treatment toxicity, add-on of new anti-cancer therapy, and death
Preliminary assessment of biomarkers that might act as pharmacodynamic, anti-tumor, and safety indicators of activity of CLDN6 CAR-T/CLDN6 CAR-T(A) +/- CLDN6 RNA-LPX	• Detection, phenotypic characterization and functionality of CLDN6 CAR-T/CLDN6 CAR-T(A) detected by flow cytometry in blood (and ascites, pleural effusion, if available)<br>• Assessment of blood tumor markers<br>• Change of CLDN6 expression in tumor biopsy<br>• Immune signature of the tumor

	• Anti-CAR antibodies, if clinically indicated
To characterize the *in vivo* cellular pharmacokinetics (PK) profile (levels, persistence, trafficking) of CLDN6 CAR-T/CLDN6 CAR-T(A) in target tissues (blood and other tissues if available)	• Presence of CLDN6 CAR-T/CLDN6 CAR-T(A) in blood and other tissues, if available<br>• Maximum concentration (C_{max}), time to maximum concentration (T_{max}), area under the time-concentration curve (AUC) and other relevant PK parameters of CLDN6 CAR-T/CLDN6 CAR-T(A) in blood<br>• Additionally for patients crossing over from Part 1 to additional cohort in Part 1 (CLDN6 CAR-T/CLDN6 CAR-T(A) + CLDN6 RNA-LPX): re-expansion of CLDN6 CAR-T/CLDN6 CAR-T(A) in blood and other tissues

Trial design

This is a Phase I/IIa, FIH, open-label, multicenter, dose escalation trial with expansion cohorts to evaluate safety and preliminary efficacy of CLDN6 CAR-T +/- CLDN6 RNA-LPX in patients with CLDN6-positive relapsed or refractory advanced solid tumors.

All patients will undergo leukapheresis for collection of blood product to manufacture the CLDN6 CAR-T.

The trial consists of three parts:

Part 1 will be a CLDN6 CAR-T dose escalation in lymphodepleted patients until the MTD and/or RP2D of CLDN6 CAR-T are defined.

In case the MTD for CLDN6 CAR-T is identified at dose level 1, the sponsor together with the Safety Review committee (SRC) may explore lower dose levels for CLDN6 CAR-T dose escalation and for bifurcation from CLDN6 CAR-T to CLDN6 CAR-T + CLDN6 RNA-LPX dose escalation based on safety and other available data.

Part 2 will be a vaccine-modulated dose escalation using a bifurcated design until the MTD and/or RP2D of CLDN6 CAR-T + CLDN6 RNA-LPX are defined. Bifurcation is planned to start when Part 1 CLDN6 CAR-T dose level 1 (1×10^7 CLDN6 CAR-T) is deemed safe. At this point, Part 2 will start with the cleared CLDN6 CAR-T dose level. CLDN6 RNA-LPX will be administered at fixed step-up doses. Cohorts testing CLDN6 +/- CLDN6 RNA-LPX without lymphodepletion (LD) may also be activated in this part to inform the decision on the preferred RP2D.

Once a dose level in Part 1 is deemed safe by the SRC, additional treatment with CLDN6 RNA-LPX will be possible upon request by the responsible PI and subject to approval by the SRC. A separate ICF will be signed by the patient.

The dose level of CLDN6 CAR-T in Part 2 at any given time will not exceed that in Part 1. This approach will allow for rapid and safe vaccine-modulated dose escalation. This will lead to the timely optimization of the schedule that will be taken to the expansion phase (Part 3). At the same time, patient safety is safeguarded by generating data on CLDN6 CAR-T before bifurcation, and clear rules for parallel dose escalations are outlined. The enrollment of patients into either CLDN6 CAR-T or vaccine-modulated dose escalation cohorts will be alternated. More patients can be enrolled into select dose levels for backfill in both Parts 1 and 2 to further explore safety and anti-tumor activity after discussion with the SRC.

After completion of Part 1 and Part 2 with CLDN6 CAR-T manufactured with a manual process (CLDN6 CAR-T [IMP-1]), the dose escalation will be repeated with CLDN6 CAR-T manufactured with an automated process (CLDN6 CAR-T(A) [IMP-1(A)]).

In Part 3, when the RP2D of CLDN6 CAR-T +/- CLDN6 RNA-LPX is established and endorsed by the SRC, pre-defined expansion cohorts will be activated. Additional cohorts can be implemented by a protocol amendment. Clinical assessments will follow the Schedule of Trial Procedures according to the treatment chosen (CLDN6 CAR-T +/- CLDN6 RNA-LPX).

Population

Patients with any of the following tumor types will be screened to determine appropriate CLDN6 status: Testicular, ovarian, gastric, endometrial, non-squamous non-small cell lung cancer (NSCLC), advanced or metastatic cancer that fulfills other inclusion criteria and for whom there is no available standard therapy likely to confer clinical benefit, or patient is not a candidate for such available therapy. Only patients with ≥ 50% positive tumor cells expressing ≥ 2+ CLDN6 protein as determined centrally are eligible to undergo further screening to meet the remaining eligibility criteria for enrollment on this trial.

Key inclusion criteria

Patients who meet the following inclusion criteria will be eligible for trial entry:

For Parts 1, 2, and 3:

- Each patient enrolled in the trial must have CLDN6-positive tumor regardless of tumor histology defined as ≥ 50% of tumor cells expressing ≥ 2+ CLDN6 protein using a semi-quantitative immunohistochemistry (IHC) assay for specific detection of CLDN6 protein expression in formalin-fixed, paraffin-embedded neoplastic tissues.
- Must have measurable disease per RECIST 1.1 (except for germ cell tumors).
- Germ cell cancer patients without initial measurable disease per RECIST 1.1 and evaluable by cancer antigen (CA)-125, Alpha-fetoprotein (AFP) or hCG (as applicable) are eligible for the trial.

For Parts 1 and 2:

- Must have a histologically confirmed solid tumor that is metastatic or unresectable and for whom there is no available standard therapy likely to confer clinical benefit, or patient who is not a candidate for such available therapy.

For Part 3 only:

Cohort 1: Testicular cancer

- Histologically or cytologically confirmed tumor of the testis of any histological subtype that has relapsed and/or is refractory to standard therapy. There is no limit on the number of prior treatment regimens.

Cohort 2: Ovarian cancer<br>• Histologically or cytologically confirmed ovarian cancer of any histology type including primary peritoneal or fallopian tube tumor that is resistant to a platinum-based chemotherapy regimen. There is no available standard therapy likely to confer clinical benefit to the patient, or they are not a candidate for such available therapy. There is no limit on the number of prior treatment regimens.<br>Cohort 3: Gastric cancer<br>• Histologically or cytologically confirmed inoperable or metastatic gastric cancer that has failed or demonstrated intolerance to standard therapy - which includes platinum or fluoropyrimidine or taxane-based chemotherapy. Human epidermal growth factor receptor 2 (HER2)-positive gastric cancer patients must have failed or demonstrated intolerance to HER2-targeting treatment. There is no limit on the number of prior treatment regimens.<br>Cohort 4: Endometrial cancer<br>• Histologically or cytologically confirmed endometrial cancer of any histology type that is resistant to a platinum-based chemotherapy regimen, or for whom there is no available standard therapy likely to confer clinical benefit, or patient is not a candidate for such available therapy. There is no limit on the number of prior treatment regimens.<br>Cohort 5: NSCLC<br>• Histological or cytological diagnosis of metastatic non-squamous NSCLC and must have progressed on the standard therapy, including platinum-based chemotherapy and/or checkpoint inhibitor therapy. Patients with epidermal growth factor receptor (EGFR), anaplastic lymphoma kinase (ALK), receptor tyrosine kinase encoded by gene ROS-1 (ROS-1) or proto-oncogene B-Raf (BRAF) mutations must have progressed on standard treatment options including EGFR, ALK, ROS-1, and BRAF directed therapies. There is no limit on the number of prior treatment regimens.<br>Cohort 6: Tumors not otherwise specified, including rare tumors and cancers of unknown primary<br>• Advanced or metastatic cancer that fulfills other inclusion criteria and for whom there is no available standard therapy likely to confer clinical benefit, or patient is not a candidate for such available therapy. There is no limit on the number of prior treatment regimens.
**Key exclusion criteria**<br>**Patients who meet at least one of the following exclusion criteria will not be eligible for trial entry:**

- Have received prior CAR-T therapy, except CLDN6 CAR-T/CLDN6 CAR-T(A) therapy.
- Have received vaccination with live virus vaccines within 6 weeks prior to the start of lymphodepletion (LD).
- Receives concurrent systemic (oral or intravenous [i.v.]) steroid therapy > 10 mg prednisolone daily, or its equivalent.
- Current evidence of new or growing brain or spinal metastases during screening. Patients with known brain or spinal metastases may be eligible if they:
    - Have had radiotherapy or another appropriate therapy for the brain or spinal metastases,
    - Have no neurological symptoms,
    - Have stable brain or spinal disease on the computer tomography or magnetic resonance imaging scan within 4 weeks before signing of the informed consent,
    - Are not undergoing acute corticosteroid therapy or steroid taper. Chronic steroid therapy is acceptable provided that the dose is stable for the last 14 d prior to screening (≤ 10 mg prednisolone daily or equivalent),
    - Do not require steroid therapy within 7 d before the first dose of CLDN6 CAR-T,
    - Have anticipated imminent fracture or cord compression due to spinal bone metastases.
- Has a history of another primary cancer within the 2 years prior to enrollment except for the following: non-melanoma skin cancer, cervical carcinoma *in situ*, superficial bladder cancer, prostate cancer with currently undetectable prostate specific antigen, or other non-metastatic carcinoma that has been in complete remission without treatment for more than 2 years.

Lymphodepleting chemotherapy

- Cyclophosphamide: 500 mg/m^2/d infused i.v. according to institutional standard on d-5, d-4, and d-3,

  and

- Fludarabine: 30 mg/m^2/d infused i.v. according to institutional standard on d-5, d-4, and d-3. In case of moderate impairment of renal function, the dose should be reduced to 50% and the patient's renal function should be monitored daily.

A lower dose of the lymphodepleting chemotherapy may be used based on the decision of the SRC:

- Cyclophosphamide: 250 mg/m^2/d infused i.v. according to institutional standard on d-5, d-4, and d-3,

and

- Fludarabine: 25 mg/m^2/d infused i.v. according to institutional standard on d-5, d-4, and d-3. In case of moderate impairment of renal function, the dose should be reduced to 50% and the patient's renal function should be monitored daily.

Investigational therapy

CLDN6 CAR-T (IMP-1) and CLDN6 CAR-T(A) (IMP-1(A)):

CLDN6 CAR-T and CLDN6 CAR-T(A) are autologous CD4$^+$ and CD8$^+$ T cells engineered with a CAR specific for CLDN6 manufactured either by a manual process (IMP-1) or by an automated process (IMP-1(A)).

CLDN6 CAR-T/CLDN6 CAR-T(A) are cryopreserved in infusible cryomedia and will be administered as a single i.v. infusion. Each bag will contain an aliquot (volume dependent upon dose) of CLDN6 CAR-T in cryomedia containing the following infusible grade reagents (% v/v): 50% CryoStor CS10, 50% sodium chloride (0.9%). The total amount of dimethylsulfoxide is 5%.

Flat dosing via the i.v. route of administration is planned for this trial. For CLDN6 CAR-T manufactured with the manual process, the selected starting dose is 10^7 CLDN6 CAR-T, followed by two additional dose levels of 10^8 and 10^9 CLDN6 CAR-Ts.

For CLDN6 CAR-T(A) manufactured with the automated process, the selected starting dose is 10^6 CLDN6 CAR-T(A), followed by three additional dose levels of 10^7, 10^8, and $2x10^8$ to $5\ x10^8$ CLDN6 CAR-T(A).

CLDN6 RNA-LPX (IMP-2):

CLDN6 RNA-LPX is a cancer vaccine, which is liposomally formulated messenger ribonucleic acid (mRNA) to systemically deliver CLDN6-encoding mRNA to antigen-presenting cells (APC) such as dendritic cells in secondary lymphoid tissues.

The CLDN6 RNA-LPX will be injected as a bolus into a peripheral vein using a peripheral venous catheter. The CLDN6 RNA-LPX injection should be performed as slowly as possible or over a time period of 2 minutes. Prior to and after the CLDN6 RNA-LPX injection, 5 mL of isotonic sodium chloride solution (0.9%) should be administered (flushing).

The starting dose of CLDN6 RNA-LPX cancer vaccine is 25 µg, followed by 50 µg for all subsequent administrations.

**Safety Review Committee**<br>An SRC will be established to review the safety data. The SRC will act according to its own written standard operating procedure described in a charter, and will prepare written minutes of its meetings.
**Statistics**<br><u>Statistical hypotheses</u><br>The primary objectives of Parts 1 and 2 are to assess the safety profile and to identify the MTD and/or RP2D. Hence, no statistical hypothesis is under test for Parts 1 and 2. For Part 3, hypothesis testing for each expansion cohort may be introduced through protocol amendment.<br><u>Sample size determination</u><br>The sample size for Part 1 and Part 2 is driven by the 3+3 trial design. In Part 1 and Part 2 with CLDN6 CAR-T, the sample size will be up to 18 DLT-evaluable patients in each part, depending on the number of DLTs that may occur. According to the 3+3 trial design with CLDN6 CAR-T(A), up to 96 patients can be enrolled into the automated cohorts in Part 1 and Part 2.<br>The objective for Part 3 is to further investigate the safety profile and to assess the efficacy of CLDN6 CAR-T/CLDN6 CAR-T(A) +/- CLDN6 RNA-LPX in different indications. The final sample size calculations will be introduced using a protocol amendment.<br><u>General considerations</u><br>Continuous variables will be summarized by cohort using the following descriptive statistics: number of patients (n), mean, standard deviation, median, minimum and maximum.<br>Categorical variables will be summarized by cohort, presenting absolute and relative frequencies (n and %) of patients in each category.<br>Time-to-event-endpoints (DOR, PFS and OS) will be analyzed using Kaplan-Meier methodology.<br>The median survival time (including 95% confidence limits) and the first and third quartile will be presented for each cohort. Survival rates (including two-sided 95% confidence interval) as well as the number and percentage of patients with events, censored and under risk will be displayed for selected time points (e.g., at 3, 6, 12 months).<br>The time-to-event analysis will be illustrated using Kaplan-Meier plots.<br><u>Primary endpoints</u><br>Adverse events (AEs) will be coded using the most recent version of Medical Dictionary for Regulatory Activities (MedDRA®) coding system to get a System Organ Class (SOC) and Preferred Term (PT) for each AE and graded for severity using National Cancer Institute Common Terminology Criteria for Adverse Events (NCI CTCAE) v5.0.

A TEAE is defined as any AE with an onset date on or after the first administration of CLDN6 CAR-T/CLDN6 CAR-T(A) (if the AE was absent before the first administration of CLDN6 CAR-T/CLDN6 CAR-T(A)) or that worsened after the first administration of CLDN6 CAR-T/CLDN6 CAR-T(A) (if the AE was present before the first administration of CLDN6 CAR-T/CLDN6 CAR-T(A)). AEs with an onset date more than 90 d after the last administration of any IMP will be considered as treatment-emergent only if assessed as related to any IMP by the Investigator. TEAEs will be summarized overall and by cohort.

The number and percentage of patients reporting at least one AE will be summarized by PT nested within SOC for each of the following AE types:

- Any AE
- Related AE
- Grade ≥ 3 AE
- Related Grade ≥ 3 AE
- Any serious adverse event (SAE)
- Related SAE
- SAE leading to death
- AE leading to dose reduction
- AE leading to permanent discontinuation of treatment
- DLT

The number and percentage of patients with any AE will be summarized by worst NCI CTCAE grade by PT nested within SOC.

DLTs will be presented in terms of listings presenting the reported term and MedDRA PT and SOC, its time of onset, duration, and outcome, relationship, NCI CTCAE grade, and seriousness including dose exposure data.

<u>Secondary endpoints</u>

Objective Response Rate

ORR is defined as the proportion of patients in whom a CR or PR (per RECIST 1.1) is observed as best overall response. Patients not meeting the criteria for CR or PR, including those without any post-baseline tumor assessments, will be considered as non-responders.

ORR will be summarized with absolute and relative frequencies along with two-sided 95% Clopper-Pearson confidence intervals by cohort.

Disease Control Rate

DCR is defined as the proportion of patients in whom a CR or PR or SD (per RECIST 1.1, SD assessed at least 6 weeks after first dose) is observed as best overall response. Patients not

meeting the criteria for CR or PR or SD, including those without any post-baseline tumor assessments, will be considered as non-responders.<br>DCR will be summarized with absolute and relative frequencies along with two-sided Clopper-Pearson 95% confidence intervals by cohort.<br>*Duration of Response*<br>DOR is defined as the time from first objective response (CR or PR per RECIST 1.1) to first occurrence of PD/recurrence, or death from any cause, whichever occurs first. Only patients in whom a CR or PR is observed will be analyzed for DOR.<br>DOR will be analyzed using Kaplan-Meier methodology by cohort.<br>Exploratory endpoints<br>The exploratory endpoints iORR, iDCR and iDOR will be analyzed in the same way as ORR, DCR and DOR.<br>*Progression-Free Survival*<br>PFS is defined as the time from first dose of CLDN6 CAR-T/CLDN6 CAR-T(A) to first confirmed PD (per RECIST 1.1, iCPD per iRECIST), or death from any cause, whichever occurs first.<br>PFS will be analyzed using Kaplan-Meier methodology by cohort.<br>*Overall Survival*<br>OS is defined as the time from first dose of CLDN6 CAR-T/CLDN6 CAR-T(A) to death from any cause.<br>OS will be analyzed using Kaplan-Meier methodology by cohort. Patients alive or patients lost to follow-up at date of analysis cut-off will be censored at the day of their last date known to be alive.

Abbreviations not defined in text: CAR, chimeric antigen receptor, CAR-T, chimeric antigen receptor T cell; RNA-LPX, liposomally formulated ribonucleic acid encoding the vaccine.

1.2 SCHEMA

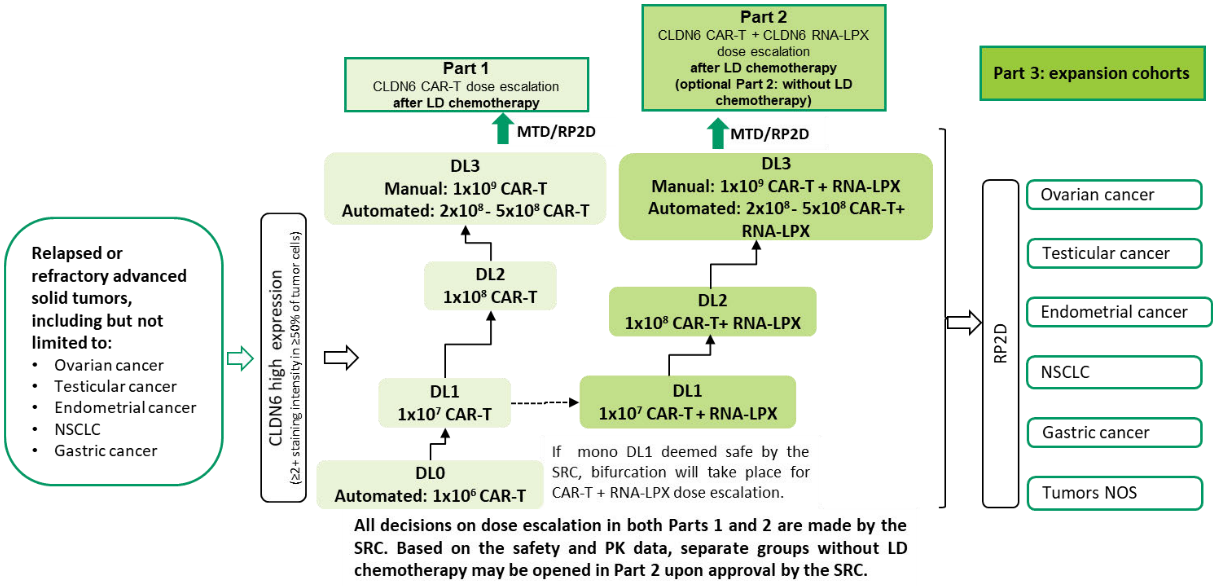

Figure 1: Trial design

Abbreviations: CAR-T, chimeric antigen receptor T cell; CLDN6, claudin 6; DL, dose level; LD, lymphodepletion; MTD, maximum tolerated dose; NOS, not otherwise specified; NSCLC, non-small- cell lung cancer; RNA-LPX, liposomally formulated ribonucleic acid encoding the vaccine; RP2D, recommended Phase 2 dose; SRC, Safety Review Committee.

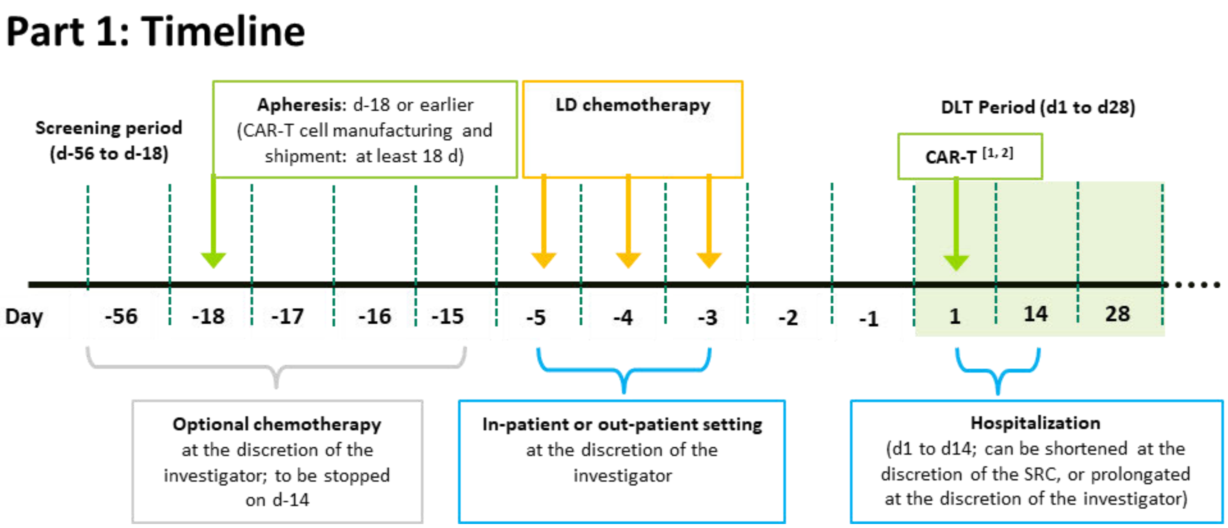

Figure 2: Timeline of trial treatment administration in Part 1 – CLDN6 CAR-T/CLDN6 CAR-T(A) dose escalation

Abbreviations: CAR-T, chimeric antigen receptor T cell; CLDN6, claudin 6; DLT, dose-limiting toxicity; IMP, investigational medicinal product; SRC, Safety Review Committee.

[1] Re-dosing with CAR-T is allowed if requested by the investigator, the IMP is available, and endorsed by the SRC. Hospitalization and other follow-up procedures will be the same as during the first dosing. During dose escalation, CLDN6 CAR-T/CLDN6 CAR-T(A) re-dosing may only occur after the patient has completed the DLT period without any DLTs being observed. Furthermore, the SRC together with the investigator will review all available safety and efficacy data plus CAR-T numbers assessed in peripheral blood by a validated assay. CAR-T re-dosing will be allowed if available data suggest that clinical benefit may be expected from re-dosing.

[2] Pre-medication recommended for CAR-T therapy is described in Section 6.1.2.1.

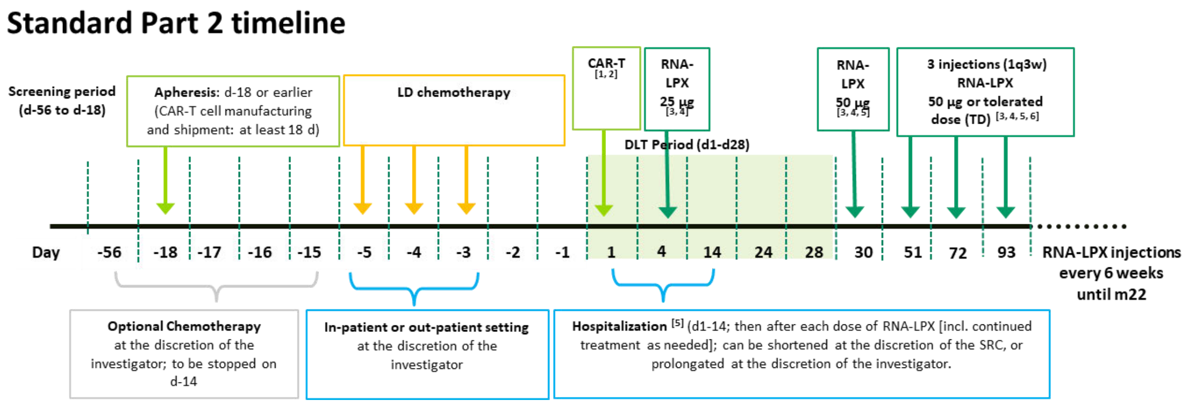

Figure 3: Timeline of trial treatment in Standard Part 2 CLDN6 CAR-T/CLDN6 CAR-T(A) + CLDN6 RNA-LPX (dose escalation)

Abbreviations: 1q3w, once every 3 weeks; CAR-T, chimeric antigen receptor T cell; d, day; DLT, dose-limiting toxicity; IMP, investigational medicinal product; LD, lymphodepleting; RNA-LPX, liposomally formulated ribonucleic acid encoding the vaccine; SRC, safety review committee; TD, tolerated dose.

[1] Re-dosing with CAR-T is allowed if requested by the investigator, the IMP is available, and endorsed by the SRC. Hospitalization and other follow-up procedures will be the same as during the first dosing or otherwise as indicated by the SRC. During dose escalation, CLDN6 CAR-T/CLDN6 CAR-T(A) re-dosing may only occur after the patient has completed the DLT period without any DLTs being observed. Furthermore, the SRC together with the investigator will review all available safety and efficacy data plus CAR-T numbers assessed in peripheral blood by a validated assay. CAR-T re-dosing will be allowed if available data suggest that clinical benefit may be expected from re-dosing.

[2] Pre-medication recommended for CAR-T therapy is described in Section 6.1.2.1.

[3] Pre-medication recommended for RNA-LPX is described in Section 6.1.3.1.

[4] Dose modifications and delays for RNA-LPX dosing are described in Section 6.6.2.

[5] Continued treatment with RNA-LPX (once every 6 weeks) is allowed after d93 at the discretion of the investigator.

[6] Hospitalization from d1 until d14. Thereafter, after each administration of CLDN6 RNA-LPX, the patient will be hospitalized as needed, but at least until improvement of any adverse event to Grade 2 or lower. Hospitalization can be shortened at the discretion of the SRC or prolonged at the discretion of the investigator.

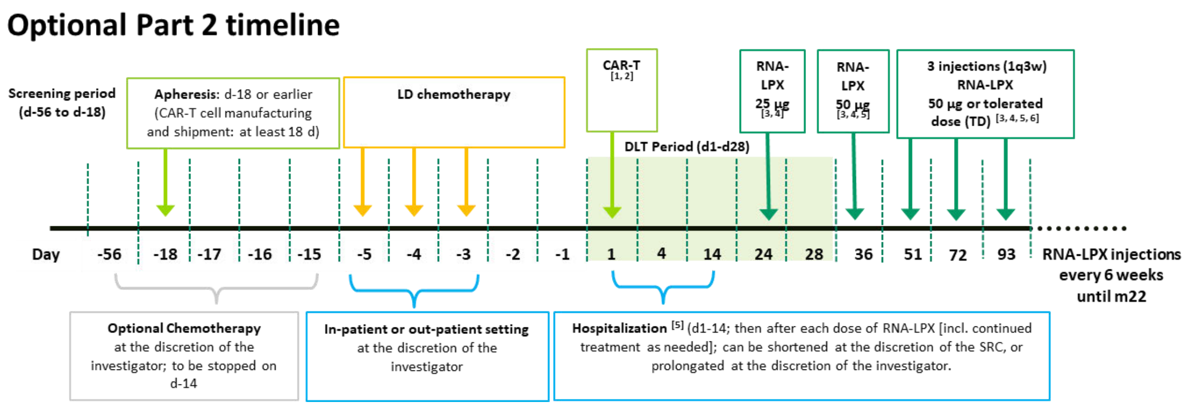

Figure 4: Timeline of trial treatment in Optional Part 2 CLDN6 CAR-T/CLDN6 CAR-T(A) + CLDN6 RNA-LPX (dose escalation)

Abbreviations: 1q3w, once every 3 weeks; CAR-T, chimeric antigen receptor T cell; d, day; DLT, dose-limiting toxicity; IMP, investigational medicinal product; LD, lymphodepleting; RNA-LPX, liposomally formulated ribonucleic acid encoding the vaccine; SRC, safety review committee; TD, tolerated dose.

[1] Re-dosing with CAR-T is allowed if requested by the investigator, the IMP is available, and endorsed by the SRC. Hospitalization and other follow-up procedures will be the same as during the first dosing or otherwise as indicated by the SRC. During dose escalation, CLDN6 CAR-T/CLDN6 CAR-T(A) re-dosing may only occur after the patient has completed the DLT period without any DLTs being observed. Furthermore, the SRC together with the investigator will review all available safety and efficacy data plus CAR-T numbers assessed in peripheral blood by a validated assay. CAR-T re-dosing will be allowed if available data suggest that clinical benefit may be expected from re-dosing.

[2] Pre-medication recommended for CAR-T therapy is described in Section 6.1.2.1.

[3] Pre-medication recommended for RNA-LPX is described in Section 6.1.3.1.

[4] Dose modifications and delays for RNA-LPX dosing are described in Section 6.6.2.

[5] Continued treatment with RNA-LPX (once every 6 weeks) is allowed after d93 at the discretion of the investigator.

[6] Hospitalization from d1 until d14. Thereafter, after each administration of CLDN6 RNA-LPX, the patient will be hospitalized as needed, but at least until improvement of any adverse event to Grade 2 or lower. Hospitalization can be shortened at the discretion of the SRC or prolonged at the discretion of the investigator.

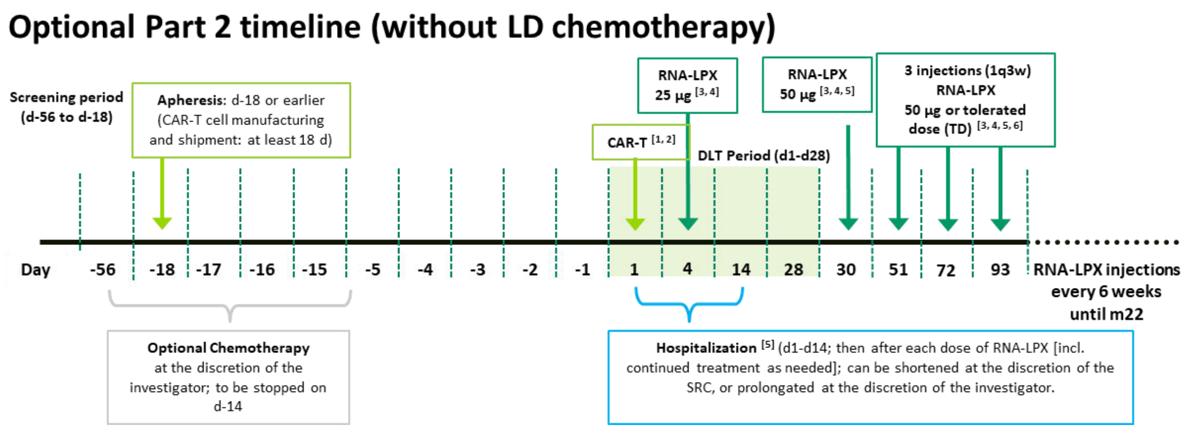

Figure 5: Timeline of trial treatment in Optional Part 2 CLDN6 CAR-T/CLDN6 CAR-T(A) + CLDN6 RNA-LPX without LD chemotherapy (dose escalation)

Abbreviations: 1q3w, once every 3 weeks; CAR-T, chimeric antigen receptor T cell; d, day; DLT, dose-limiting toxicity; IMP, investigational medicinal product; RNA-LPX, liposomally formulated ribonucleic acid encoding the vaccine; SRC, Safety Review Committee; TD, tolerated dose.
[1] Re-dosing with CAR-T is allowed if requested by the investigator, the IMP is available, and endorsed by the SRC. Hospitalization and other follow-up procedures will be the same as during the first dosing or otherwise as indicated by the SRC. During dose escalation, CLDN6 CAR-T/CLDN6 CAR-T(A) re-dosing may only occur after the patient has completed the DLT period without any DLTs being observed. Furthermore, the SRC together with the investigator will review all available safety and efficacy data plus CAR-T numbers assessed in peripheral blood by a validated assay. CAR-T re-dosing will be allowed if available data suggest that clinical benefit may be expected from re-dosing.
[2] Pre-medication recommended for CAR-T therapy is described in Section 6.1.2.1.
[3] Pre-medication recommended for RNA-LPX is described in Section 6.1.3.1.
[4] Dose modifications and delays for RNA-LPX dosing are described in Section 6.6.2.
[5] Continued treatment with RNA-LPX (every 6 weeks) is allowed after d93 at the discretion of the investigator.
[6] Hospitalization from d1 until d14. Thereafter, after each administration of CLDN6 RNA-LPX, the patient will be hospitalized as needed, but at least until improvement of any adverse event to Grade 2 or lower. Hospitalization can be shortened to d7 at the discretion of the SRC in case the patient shows no signs of CRS/ICANS. Prolonged hospitalization is allowed at the discretion of the investigator.

1.3 SCHEDULES OF ACTIVITIES

1.3.1 Part 1 SoA (CLDN6 CAR-T/CLDN6 CAR-T(A))

Table 1: Part 1 SoA (CLDN6 CAR-T/CLDN6 CAR-T(A)) pre-screening, screening, apheresis, pre-treatment

Phase	Pre-screening for CLDN6 expression	Screening [1]	Apheresis		Pre-treatment		
Visit name			Leukapheresis	Post-leukapheresis FU	LD chemotherapy		
Trial period	Before screening	d-56 to d-18 [1]	d-18 (or earlier)	Within 3 d of leukapheresis	d-5	d-4	d-3
Informed consent [2]	X	X					
**Patient history**							
Inclusion/exclusion criteria		X					
Demography [3]	X	X					
Medical history		X					
Diagnosis and extent of cancer	X	X					
Prior antineoplastic therapies		X					
Prior/concomitant therapies [4]		X	X	X	X	X	X
Height		X					
Body weight		X					
Physical examination [5]		X	X	X	X	X	X
Vital signs [6]		X	X		X	X	X
ECG		X			(X)	(X)	(X)
ECOG performance status		X	X				
Eligibility		X					
**Intervention**							
Leukapheresis screening (according to local standards)		X					
Leukapheresis			X				
LD chemotherapy [7]					X	X	X
Optional chemotherapy [8]		As clinically indicated					

Phase	Pre-screening for CLDN6 expression	Screening [1]	Apheresis		Pre-treatment		
Visit name			Leukapheresis	Post-leukapheresis FU	LD chemotherapy		
Trial period	Before screening	d-56 to d-18 [1]	d-18 (or earlier)	Within 3 d of leukapheresis	d-5	d-4	d-3
**Disease assessments**							
Archival tumor tissue [9]	X						
Fresh biopsy [10]	(X)	As clinically indicated					
Ascites/pleural effusion sample [11]		As clinically indicated					
Tumor assessment [12]		X			X [13]		
**Safety**							
Adverse events [14]		X	X	X	X	X	X
**Laboratory assessments**							
Serology/virology (HIV test, Hep B+C) (central lab)		X					
Hematology (central lab) [15]		X			X (pre)		
Clinical chemistry (central lab) [15]		X			X (pre)		
Coagulation (central lab)		X					
Endocrine tests (TSH, T3, and T4) (central lab)		X					
Lab test of special interest for CRS/ICANS (IL-6, CRP, ferritin, fibrinogen, procalcitonin) (central lab) [15,16]					X (pre)		
Urinalysis (local lab)		X					
Serum pregnancy test (local lab) [17]		X			X		
FSH and estradiol tests (central lab) [18]		X					

Phase	Pre-screening for CLDN6 expression	Screening [1]	Apheresis		Pre-treatment		
Visit name			Leukapheresis	Post-leukapheresis FU	LD chemotherapy		
Trial period	Before screening	d-56 to d-18 [1]	d-18 (or earlier)	Within 3 d of leukapheresis	d-5	d-4	d-3
**Biomarker**							
Flow-phenotype [19]					X		
Tumor biomarker [19]					X		
Tumor tissue (molecular profiling) [20]		X					
HLA typing		X					

Abbreviations: CAR, chimeric antigen receptor; CAR-T, chimeric antigen receptor T cell; CLDN6, claudin 6; CRP, C-reactive protein; CRS, cytokine release syndrome; d, day; ECG, electrocardiogram; ECOG, Eastern Cooperative Oncology Group; EoT, End of Trial; FSH, follicle-stimulating hormone; FU, follow-up; h, hour; Hep B, hepatitis B virus; Hep C, hepatitis C virus; HIV, human immunodeficiency virus; ICANS, immune effector cell-associated neurotoxicity syndrome; ICF, informed consent form; IL 6, interleukin 6; LD, lymphodepleting; LDH, lactate dehydrogenase; m, month; T3, triiodothyronine; T4, thyroxine; TSH, thyroid-stimulating hormone; (X), as clinically indicated.

1) All screening assessments must be completed prior to leukapheresis: completion of screening assessments within a 4-week window is preferred.
2) The patient will sign one pre-screening ICF for CLDN6 expression evaluation at the Pre-screening Visit and the main ICF at the Screening Visit.
3) Year of birth/age, sex, ethnicity, race.
4) All therapies (including medications, non-drug therapies) being taken by the patient from screening until 90 d after last IMP treatment will be regarded as prior or concomitant therapies (newly started or ongoing during this time period). Afterwards only therapies corresponding to related AEs will be recorded.
5) A complete physical examination will be performed at screening and the EoT Visit. At other visits, an abbreviated examination will be performed and new or worsened clinically significant abnormalities will be recorded. Patients should be examined daily when hospitalized. All physical examinations should include the immune effector cell-associated encephalopathy (ICE) score assessment.
6) Vital signs (systolic and diastolic blood pressure, heart rate, body temperature, and oxygen saturation) will be measured in a seated or recumbent position after at least 5 min rest (please refer to Section 8.2.2). Vital signs should be checked daily when the patient is hospitalized.
7) LD chemotherapy should be completed at least three days prior to the CLDN6 CAR-T/CLDN6 CAR-T(A) infusion. Prior to LD chemotherapy initiation, infections, cardiac functions, glomerular filtration rate, and neurological assessments should be measured (please refer to Section 6.1.1). Only if the criteria apply, can the LD chemotherapy be initiated.
8) Refer to Section 6.5.2 for further information about documenting optional chemotherapy.
9) The formalin-fixed, paraffin-embedded (FFPE) sample should be from the most recent tumor tissue obtained. If no archival tumor tissue is available, the patient must be biopsied for CLDN6 staining at pre-screening. In addition, a baseline pre-treatment tumor sample (archival or fresh) is required.
10) At the Pre-screening Visit, patients must be biopsied if the most recent FFPE sample obtained is not available anymore. During the course of the trial fresh tumor biopsies should be taken at the discretion of the investigator, if this does not pose a significant risk for the patient.
11) Ascites and pleural effusion samples should be taken during the course of the trial at the discretion of the investigator, if this does not pose a significant risk for the patient.
12) Tumor assessment will be performed during screening, at 6 weeks (±7d) post infusion, then every 6 weeks (±7 d) for 50 weeks and every 12 weeks (±7 d) thereafter. Tumor response will be evaluated according to RECIST v1.1 and iRECIST.
13) Tumor assessment will be performed at d-5, if screening was more than 4 weeks ago and in case of bridging chemotherapy.
14) All AEs will be reported from signing of main ICF until 90 d after last IMP treatment. Afterwards only related AEs will be reported. Prior to signing of main ICF only SAEs related to a trial procedure will be reported.
15) At visit d-5, blood samples will be collected prior to the start of LD chemotherapy.
16) In case the lab test of special interest blood draw is not performed on the same visit as blood draw for hematology and clinical chemistry, blood sample for hematology and clinical chemistry need to be collected in addition.

17) Pregnancy tests will be performed for women of childbearing potential only. CAR-T dosing will only occur with a repeated negative pregnancy test (performed 5 d prior to CLDN6 CAR-T/CLDN6 CAR-T(A) administration).
18) FSH and estradiol tests will be performed at screening if indicated in post-menopausal females.
19) At visit d-5, blood samples will be collected prior to LD chemotherapy administration.
20) Collection of tumor tissue before treatment start, either the most recent archival FFPE material or a fresh biopsy is mandatory for molecular profiling (e.g., NGS, RNAseq, multiplex immune-profiling).

Table 2: Part 1 SoA (CLDN6 CAR-T/CLDN6 CAR-T(A)) treatment, primary follow-up

Phase	Treatment and primary follow-up												EoT & Primary FU [1]
Visit name	Infusion of CAR-T	Post infusion											
Trial period	d1	d2	d4 ±1d	d8 ±1d	d11 ±1d	d14 ±1d	d17 ±1d	d24 ±1d	d28 ±1d	d59, d87, d108 (=week16/m4) ±3d	d150, d192, d234, d276, d318, d360 ±7d	m15, m18, m21, m24 ±14d	m25 ±14d [2]
**Patient history**													
Prior/concomitant therapies [3]	X	X	X	X	X	X	X	X	X	X	X	X	(X)
Body weight	X								X	Only d87 (m3)			X
Physical examination [4]	X	X	X	X	X	X	X	X	X	X	X	X	X
Vital signs [5]	X	X	X	X	X	X	X	X	X	X	X	X	X
ECG	(X)	(X)	(X)	(X)	(X)	(X)	(X)	(X)	(X)	(X)	(X)	(X)	(X)
ECOG Performance Status	X	X		X		X		X	X	X	X	X	X
Hospitalization status[6]	Hospitalization from d1-d14												
**Intervention**													
CLDN6 CAR-T/CLDN6 CAR-T(A) cell infusion [7]	X												
Documentation of antineoplastic therapy after CLDN6 infusion or trial discontinuation	X	X	X	X	X	X	X	X	X	X	X	X	X
**Disease assessment**													
Fresh biopsy [8]	As clinically indicated												
Ascites/pleural effusion sample [9]	As clinically indicated												
Tumor assessment [10]										Every 6 weeks (±7d) for 50 weeks, and every 12 weeks (±7d) thereafter			
**Safety**													
Adverse events [11]	X	X	X	X	X	X	X	X	X	X	X	X	(X)
**Laboratory assessments**													
Hematology (central lab) [12]	X (pre)	X	X	X	X	X	X	X	X	X	X	X	X
Clinical chemistry (central lab) [12]	X (pre)	X	X	X	X	X	X	X	X	X	X	X	X
Coagulation (central lab)	X (pre)												X

**Phase**	**Treatment and primary follow-up**												**EoT & Primary FU** [1]
**Visit name**	**Infusion of CAR-T**	**Post infusion**											
**Trial period**	**d1**	**d2**	**d4 ±1d**	**d8 ±1d**	**d11 ±1d**	**d14 ±1d**	**d17 ±1d**	**d24 ±1d**	**d28 ±1d**	**d59, d87, d108 (=week16/m4) ±3d**	**d150, d192, d234, d276, d318, d360 ±7d**	**m15, m18, m21, m24 ±14d**	**m25 ±14d** [2]
Endocrine tests (TSH, T3, and T4) (central lab)									X	X	X	X	X
Lab test of special interest for CRS/ICANS (IL-6, CRP, ferritin, fibrinogen, procalcitonin) (central lab) [13]	X (pre+6h post)	X	X	X	X	X	X	X	X				
Serum pregnancy test (local lab) [14]										X	X	X	X
**Biomarker** [15]													
Cytokines [15, 16]	X (pre)	X	d3&d4	X	X		X	X		d38, d45, d52, d59, d66, d73, d80, d94, d108 ±3d [16]	X		
qPCR incl. hematology [15,17]		X	d3&d4	X	X		X	X		d38, d45, d52, d59, d66, d73, d80, d94, d108 ±3d [17]	X	X	
Flow-phenotype [15]					X		X	X			X	X	
CAR characterization [15,18]							X			d45 (±3d), d87 (±3d)			
Tumor biomarker [15]					X		X	X		d38, d52, d66, d80, d94, d108	X	X	
CAR immunogenicity assay [15,19]	X (pre)							X		d94			
Tumor tissue (fresh) [20]		As clinically indicated											
**Survival status**	**For all patients who receive a CLDN6 CAR-T/CLDN6 CAR-T(A) infusion, patients may be enrolled into the long-term follow-up period for survival status as per schedule in Appendix 8 after the EoT Visit. If a patient misses a scheduled visit where survival status is required, survival status can be obtained via phone contact.**												

Abbreviations: CAR, chimeric antigen receptor; CAR-T, chimeric antigen receptor T cell; CLDN6, claudin 6; CRP, C-reactive protein; CRS, cytokine release syndrome; d, day; ECG, electrocardiogram; ECOG, Eastern Cooperative Oncology Group; EoT, End of Trial; FSH, follicle-stimulating hormone; FU: follow-up; h, hour; Hep B, hepatitis B virus; Hep C, hepatitis C virus; HIV, human immunodeficiency virus; ICANS, immune effector cell-associated neurotoxicity syndrome; ICF, informed consent form; IL 6, interleukin 6; INR, International normalized ratio; LDH, lactate dehydrogenase; (LT)FU, (long-term) follow-up; m, month; qPCR, quantitative polymerase chain reaction; T3, triiodothyronine; T4, thyroxine; TSH, thyroid-stimulating hormone; (X), as clinically indicated.

1) Refer to LTFU appendix for follow-up after trial completion.

2) All patients (if alive) who discontinue trial treatment will be evaluated at a Safety/EoT Follow-up Visit, at least 90 d and no more than 97 d after the last trial treatment administration. This visit will replace a regular visit if it is in the same visit window.
3) All therapies (including medications, non-drug therapies) being taken by the patient from screening until 90 d after last IMP treatment will be regarded as prior or concomitant therapies (newly started or ongoing during this time period). Afterwards only therapies corresponding to related Aes will be recorded.
4) A complete physical examination will be performed at screening and the EoT Visit. At other visits, an abbreviated examination will be performed and new or worsened clinically significant abnormalities will be recorded. Patients should be examined daily when hospitalized. All physical examinations should include the immune effector cell-associated encephalopathy (ICE) score assessment; the ICE score must be assessed daily within the hospitalization period.
5) Vital signs (systolic and diastolic blood pressure, heart rate, body temperature, and oxygen saturation) will be measured in a seated or recumbent position after at least 5 min rest (please refer to Section 8.2.2). Vital signs should be checked daily when the patient is hospitalized.
6) Hospitalization can be shortened to d7 at the discretion of the SRC in case the patient shows no signs of CRS/ICANS. Prolonged hospitalization is allowed at the discretion of the investigator.
7) The CAR-T dose will be administered by i.v. infusion. At visit d1, assessments according to Section 6.1.2.2 should be made before CLDN6 CAR-T/CLDN6 CAR-T(A) administration. Only if the criteria apply, can the infusion of CLDN6 CAR-T/CLDN6 CAR-T(A) take place.
8) During the course of the trial fresh tumor biopsies should be taken at the discretion of the investigator, if this does not pose a significant risk for the patient.
9) Ascites and pleural effusion samples should be taken during the course of the trial at the discretion of the investigator, if this does not pose a significant risk for the patient.
10) Tumor assessment will be performed during screening, at 6 weeks (±7d) post infusion, then every 6 weeks (±7 d) for 50 weeks and every 12 weeks (±7 d) thereafter. Tumor response will be evaluated according to RECIST v1.1 and iRECIST.
11) All AEs will be reported from signing of main ICF until 90 d after last IMP treatment. Afterwards only related AEs will be reported. Prior to signing of main ICF only SAEs related to a trial procedure will be reported.
12) At visit d1, blood samples will be collected prior to infusion of CAR-T. At visit d4, blood samples will not be collected in case the visit falls on a weekend.
13) At visit d1, blood samples will be collected prior to infusion of CAR-T, and 6 h post infusion (± 1 h). If CRS/ICANS is suspected, parameters should be assessed daily by the central laboratory for the whole duration of the CRS/ICANS. As soon as the CRS/ICANS is over, parameters will be analyzed at the visits indicated in the SoA again (refer to Section 6.6.4.1). At visit d4, blood samples will not be collected in case the visit falls on a weekend (blood collection on a weekend only in case of a CRS/ICANS).
14) Pregnancy tests will be performed for women of childbearing potential only. CAR-T dosing will only occur with a repeated negative pregnancy test (performed 5 d prior to CLDN6 CAR-T/CLDN6 CAR-T(A) administration).
15) After one year: The indicated biomarker assessments will only be performed if clinically indicated (after discussion with sponsor) – if CLDN6 CAR-T/CLDN6 CAR-T(A) are not detectable, biomarker assessments would cease with the exception of the qPCR assessment, and tumor biomarkers where relevant.
16) At visit d1, blood samples will be collected prior to infusion of CAR-T. Samples will be collected every 6 weeks after d108 until d356. At visit d4, blood samples will not be collected in case the visit falls on a weekend.
17) At visit d1, blood samples will be collected prior to infusion of CAR-T. A blood tube for hematology assessments must be taken with all samples for qPCR, if not already specified for this time point in the SoA. Samples will be collected every 6 weeks after d108 until d356, then every 3 m until d712. At visit d4, blood samples will not be collected in case the visit falls on a weekend.
18) For CAR characterization, the second and third time points should be aligned with the tumor assessments at 6 and 12 weeks. Up to three unscheduled additional blood sample collections are allowed if indicated by qPCR.
19) At visit d1, blood samples will be collected prior to infusion of CAR-T.
20) An on treatment fresh tumor biopsy should be taken at the discretion of the investigator, if this does not pose a significant risk for the patient, ideally around d17 and at progression for molecular profiling and to determine CLDN6 expression and CAR-T infiltration into the tumor tissue.

1.3.2 Part 1 cross-over SoA (CLDN6 CAR-T/CLDN6 CAR-T(A) + CLDN6 RNA-LPX)

Table 3: Part 1 cross-over SoA – treatment, primary follow-up for patients crossing over from Part 1 (CLDN6 CAR-T/CLDN6 CAR-T(A)) to additional cohort in Part 1 (CLDN6 CAR-T/CLDN6 CAR-T(A) + CLDN6 RNA-LPX)

**Phase**	**Treatment and primary follow-up**																**EoT & Primary FU** [1]
**Visit name**	**CLDN6 RNA-LPX treatment phase**																
**Trial period**	**dx+ d4**	**dx+ d5 d6 d7**	**dx+ d8**	**dx+ d10**	**dx+ d17**	**dx+ d24 ±1d**	**dx+ d30-d35**	**dx+ d36, d43 ±1d**	**dx+ d51-d56**	**dx+ d57 ±1d**	**dx+ d72-d77**	**dx+ d78, d85 ±1d**	**dx+ d93-d98**	**dx+ d99 ±1d**	**dx+ d135, d177, d219, d261, d303, d345** [2]**, d387, d429, d471, d513, d555, d597, d639, d681 ±7d**	**Safety Visit** [3]	**dx+ m25 ±14d**
**Patient history**																	
Prior/concomitant therapies [4]	X	X	X	X	X	X	X	X	X	X	X	X	X	X	(X)	X	(X)
Body weight							dx+d30									X	X
Physical examination [5]	X	X	X	X	X	X	X	X	X	X	X	X	X	X	X	X	X
Vital signs [6]	X	X	X	X	X	X	X	X	X	X	X	X	X	X	X	X	X
ECG	(X)	(X)	(X)	(X)	(X)	(X)	(X)	(X)	(X)	(X)	(X)	(X)	(X)	(X)	(X)	(X)	(X)
ECOG Performance Status	X				X		dx+d30	dx+d36	dx+d51	X	dx+d72	dx+d85	dx+d93	X	X	X	X
Hospitalization status [7]	dx+d4-d5 (24 h)																
**Intervention**																	
Vaccination of CLDN6 RNA-LPX [8]	X						dx+d30		dx+d51		dx+d72		dx+d93		Vaccinations every 6 weeks (±7d) until m22		
Documentation of antineoplastic therapy after CLDN6 infusion or trial discontinuation	X	X	X	X	X	X	X	X	X	X	X	X	X	X	X	X	X
**Disease assessment**																	
Fresh biopsy [9]		As clinically indicated															

Phase	Treatment and primary follow-up																EoT & Primary FU [1]
**Visit name**	**CLDN6 RNA-LPX treatment phase**																
**Trial period**	**dx+ d4**	**dx+ d5 d6 d7**	**dx+ d8**	**dx+ d10**	**dx+ d17**	**dx+ d24 ±1d**	**dx+ d30-d35**	**dx+ d36, d43 ±1d**	**dx+ d51-d56**	**dx+ d57 ±1d**	**dx+ d72-d77**	**dx+ d78, d85 ±1d**	**dx+ d93-d98**	**dx+ d99 ±1d**	**dx+ d135, d177, d219, d261, d303, d345 [2], d387, d429, d471, d513, d555, d597, d639, d681 ±7d**	**Safety Visit [3]**	**dx+ m25 ±14d**
Ascites/pleural effusion sample [10]		As clinically indicated															
Tumor assessment [11]								dx+d43 (±7d)	Week 6 (±7d) post infusion, every 6 weeks (±7d) for 50 weeks, and every 12 weeks (±7d) thereafter								
**Safety**																	
Adverse events [12]	X	X	X	X	X	X	X	X	X	X	X	X	X	X	(X)	(X)	(X)
**Laboratory assessments**																	
Hematology (central lab) [13]	X (pre)		X	X	X	X	dx+d30 (pre)	X	dx+d51 (pre)	X	dx+d72 (pre)	X	dx+d93 (pre)	X	X	X	X
Clinical chemistry (central lab) [13]	X (pre)		X	X	X	X	dx+d30 (pre)	X	dx+d51 (pre)	X	dx+d72 (pre)	X	dx+d93 (pre)	X	X	X	X
Coagulation (central lab)																	X
Endocrine tests (TSH, T3, and T4) (central lab)							dx+d30 (pre)						dx+d93 (pre)		X	X	X
Lab test of special interest for CRS/ICANS (IL 6, CRP, ferritin, fibrinogen, procalcitonin) (central lab) [14]	X (pre, 6h)		X	X	X	X	dx+d30 (pre)	X	dx+d51 (pre)	X	dx+d72 (pre)	X	dx+d93 (pre)	X			
Serum pregnancy test (local lab) [15]										X				X	X	X	X
**Biomarker [16]**																	
Cytokines [16,17]	X (pre, 6h, 24h)		X	X	X	X	dx+d30(pre, 6h, 24h)	dx+d36 (±1d)	dx+d51 (pre, 6h, 24h)	X	dx+d72 (pre, 6h, 24h)	dx+d78 (±1d)	dx+d93 (pre, 6h, 24h)	X	dx+d135 (pre, 6h, 24h), dx+d141, then every 6/7 weeks (±7d) [18]		

Phase	Treatment and primary follow-up															EoT & Primary FU [1]
**Visit name**	**CLDN6 RNA-LPX treatment phase**															
**Trial period**	**dx+ d4**	**dx+ d5 d6 d7**	**dx+ d8**	**dx+ d10**	**dx+ d17**	**dx+ d24 ±1d**	**dx+ d30- d35**	**dx+ d36, d43 ±1d**	**dx+ d51- d56**	**dx+ d57 ±1d**	**dx+ d72-d77**	**dx+ d78, d85 ±1d**	**dx+ d93- d98**	**dx+ d99 ±1d**	**dx+ d135, d177, d219, d261, d303, d345 [2], d387, d429, d471, d513, d555, d597, d639, d681 ±7d**	**Safety Visit [3]**	**dx+ m25 ±14d**
qPCR (incl. hematology) [17,18]	X (pre)		X	X	X	X	dx+d30(pre)	dx+d36 (±1d) (±1d)	dx+d51 (pre)	X	dx+d72 (pre)	dx+d78 (±1d)	dx+d93 (pre)	X	dx+d135 (pre), dx+d141, then every 6/7 weeks (±7d) [19]		
Flow-phenotype [17,19]				X	X	X											
CAR characterization [17,20]					X			dx+d43 (±1d)				dx+d85 (±1d)					
Tumor biomarker [17,21]				X		X		dx+d36 (±1d)	dx+d51 (pre)		dx+d72 (pre)		dx+d93 (pre)		dx+d135 (pre) (±7d)		
CAR immunogenicity assay [17,21]						X							dx+d94				
Tumor tissue (fresh) [22]	As clinically indicated																
**Survival status**	**For all patients who receive a CLDN6 CAR-T/CLDN6 CAR-T(A) infusion, patients are enrolled into the long-term follow-up period for survival status as per schedule in Appendix 8 after the EoT Visit. If a patient misses a scheduled visit where survival status is required, survival status can be obtained via phone contact.**																

Abbreviations: AE, adverse event; CAR, chimeric antigen receptor; CAR-T, chimeric antigen receptor T cell; CLDN6, claudin 6; CRP, C-reactive protein; CRS, cytokine release syndrome; d, day; ECG, electrocardiogram; ECOG, Eastern Cooperative Oncology Group; EoT, End of Trial; FSH, follicle-stimulating hormone; FU, follow-up; h, hour; Hep B, hepatitis B virus; Hep C, hepatitis C virus; HIV, human immunodeficiency virus; ICANS, immune effector cell-associated neurotoxicity syndrome; ICF, informed consent form; IL 6, interleukin 6; INR, international normalized ratio; LDH, lactate dehydrogenase; (LT)FU, (long-term) follow-up; m, month; qPCR, quantitative polymerase chain reaction; RNA-LPX, liposomally formulated ribonucleic acid encoding the vaccine; T3, triiodothyronine; T4, thyroxine; TSH, thyroid-stimulating hormone; (X), as clinically indicated.

1) Refer to LTFU appendix for follow-up after trial completion.
2) If at m12/d345 vaccinations cease, visits switch from every 6 weeks to quarterly (m15, m18, m21, m24).
3) All patients (if alive) who discontinue trial treatment will be evaluated at a Safety/EoT Follow-up Visit at least 90 d and no more than 97 d after the last trial treatment administration. This visit will replace a regular visit if it is in the same visit window.

4) All therapies (including medications, non-drug therapies) being taken by the patient from screening until 90 d after last IMP treatment will be regarded as prior or concomitant therapies (newly started or ongoing during this time period). Afterwards only therapies corresponding to related AEs will be recorded.
5) A complete physical examination will be performed at screening and the EoT Visit. At other visits, an abbreviated examination will be performed and new or worsened clinically significant abnormalities will be recorded. Patients should be examined daily when hospitalized. On CLDN6 RNA-LPX vaccination days, patients should be examined prior to and 4-6 h following vaccination. All physical examinations should include the ICE score; the ICE score must be assessed daily within the hospitalization period.
6) Vital signs (systolic and diastolic blood pressure, heart rate, body temperature, and oxygen saturation) will be measured in a seated or recumbent position after at least 5 min rest (please refer to Section 8.2.2), and should be measured daily if the patient is hospitalized. On CLDN6 RNA-LPX vaccination days, vital signs should be checked prior to and 4-6 h following vaccination.
7) Hospitalization from dx+d4 until dx+d5 after cross-over (24 h). Thereafter, after each administration of CLDN6 RNA-LPX, the patient will be hospitalized as needed, but at least until improvement of any adverse event to Grade 2 or lower. Prolonged hospitalization is allowed at the discretion of the investigator.
8) The vaccination will be administered as a slow i.v. bolus injection. Further vaccinations after dx+d93 are given every 6 weeks until m22. Refer to Section 6.1.3 for further information.
9) During the course of the trial fresh tumor biopsies should be taken at the discretion of the investigator, if this does not pose a significant risk for the patient.
10) Ascites and pleural effusion samples should be taken during the course of the trial at the discretion of the investigator, if this does not pose a significant risk for the patient..
11) Tumor assessment will be performed during screening, at 6 weeks (±7 d) post infusion, then every 6 weeks (±7 d) for 50 weeks and every 12 weeks (±7 d) thereafter. Tumor response will be evaluated according to RECIST v1.1 and iRECIST.
12) All AEs will be reported from signing of the main ICF until 90 d after last IMP treatment. Afterwards only related AEs will be reported. Prior to signing of main ICF only SAEs related to a trial procedure will be reported.
13) At all RNA-LPX vaccination visits, blood samples will be collected prior to vaccination. At visit d5,6,7 blood samples will not be collected in case the visit falls on a weekend (blood collection on a weekend only in case of a CRS/ICANS).
14) At visit dx+d4 blood samples will be collected prior to infusion of CAR-T, and 6 h (±1 h) post infusion. If CRS/ICANS is suspected, the parameters should be assessed daily by the central laboratory for the whole duration of the CRS/ICANS. As soon as the CRS/ICANS is over, parameters will be analyzed at the visits indicated in the SoA again (refer to Section 6.6.4.1).
15) Pregnancy tests will be performed for women of childbearing potential only.
16) After one year: The indicated biomarker assessments will only be performed if clinically indicated (after discussion with sponsor) – if CLDN6 CAR-T/CLDN6 CAR-T(A) are not detectable, biomarker assessments would cease with the exception of the qPCR assessment, and tumor biomarkers where relevant.
17) On RNA-LPX administration visits, blood samples will be collected prior to administration of RNA-LPX. After dx+d141, every 6 weeks (pre- and 6 h, and 1 week after) RNA-LPX vaccination, if no RNA-LPX vaccination, then together with tumor assessment.
18) On RNA-LPX administration visits, blood samples will be collected prior to administration of RNA-LPX. A blood tube for hematology assessments must be taken with all samples for qPCR, if not already specified for this time point in the SoA. After dx+d141, every 6 weeks (pre- and 1 week after) RNA-LPX vaccination, if no RNA-LPX vaccination, then together with tumor assessment.
19) On RNA-LPX administration visits, blood samples will be collected prior to administration of RNA-LPX. After dx+d141, every 6 weeks (pre- and 1 week after) RNA-LPX vaccination, if no RNA-LPX vaccination, then together with tumor assessment.
20) For CAR characterization, the second and third time points should be aligned with the tumor assessments at 6 and 12 weeks. Up to three unscheduled additional blood sample collections are allowed if indicated by qPCR.
21) On RNA-LPX administration visits, blood samples will be collected prior to administration of RNA-LPX.
22) An on treatment fresh tumor biopsy should be taken at the discretion of the investigator, if this does not pose a significant risk for the patient, ideally around d17 and at progression for molecular profiling and to determine CLDN6 expression and CAR-T infiltration into the tumor tissue.

1.3.3 Standard Part 2 SoA (CLDN6 CAR-T/CLDN6 CAR-T(A) + CLDN6 RNA-LPX)

Table 4: Standard Part 2 SoA - pre-screening, screening, apheresis, pre-treatment

Phase	Pre-screening for CLDN6 expression	Screening [1]	Apheresis		Pre-treatment		
Visit name			Leukapheresis	Post-leukapheresis FU	LD chemotherapy		
Trial period	Before screening	d-56 to d-18 [1]	d-18 (or earlier)	Within 3 d of leukapheresis	d-5	d-4	d-3
Informed consent [2]	X	X					
**Patient history**							
Inclusion/exclusion criteria		X					
Demography [3]	X	X					
Medical history		X					
Diagnosis and extent of cancer	X	X					
Prior antineoplastic therapies		X					
Prior/concomitant therapies [4]		X	X	X	X	X	X
Height		X					
Body weight		X					
Physical examination [5]		X	X	X	X	X	X
Vital signs [6]		X	X		X	X	X
ECG		X			(X)	(X)	(X)
ECOG performance status		X	X				
Eligibility		X					
**Intervention**							
Leukapheresis screening (according to local standards)		X					
Leukapheresis			X				

**Phase**	**Pre-screening for CLDN6 expression**	**Screening [1]**	**Apheresis**		**Pre-treatment**		
**Visit name**			**Leukapheresis**	**Post-leukapheresis FU**	**LD chemotherapy**		
**Trial period**	**Before screening**	**d-56 to d-18 [1]**	**d-18 (or earlier)**	**Within 3 d of leukapheresis**	**d-5**	**d-4**	**d-3**
LD chemotherapy [7]					X	X	X
Optional chemotherapy [8]		As clinically indicated					
**Disease assessments**							
Archival tumor tissue [9]	X						
Fresh biopsy [10]	(X)	As clinically indicated					
Ascites/pleural effusion sample [11]		As clinically indicated					
Tumor assessment [12]		X			X [13]		
**Safety**							
Adverse events [14]		X	X	X	X	X	X
**Laboratory assessments**							
Serology/virology (HIV test, Hep B+C) (central lab)		X					
Hematology (central lab) [15]		X			X		
Clinical chemistry (central lab) [15]		X			X		
Coagulation (central lab)		X					
Endocrine tests (TSH, T3, and T4) (central lab)		X					
Lab test of special interest for CRS/ICANS (IL-6, CRP, ferritin, fibrinogen,					X		

Phase	Pre-screening for CLDN6 expression	Screening [1]	Apheresis		Pre-treatment		
Visit name			Leukapheresis	Post-leukapheresis FU	LD chemotherapy		
Trial period	Before screening	d-56 to d-18 [1]	d-18 (or earlier)	Within 3 d of leukapheresis	d-5	d-4	d-3
procalcitonin) (central lab) [15,16]							
Urinalysis (local lab)		X					
Serum pregnancy test (central lab) [17]		X			X		
FSH and estradiol tests (central lab) [18]		X					
**Biomarker**							
Flow-phenotype [19]					X (pre)		
Tumor biomarker [19]					X (pre)		
Tumor tissue (molecular profiling) [20]		X					
HLA typing		X					

Abbreviations: AE, adverse event; CAR, chimeric antigen receptor; CAR-T, chimeric antigen receptor T cell; CLDN6, claudin 6; CRP, C-reactive protein; CRS, cytokine release syndrome; d, day; ECG, electrocardiogram; ECOG, Eastern Cooperative Oncology Group; EoT, End of Trial; FSH, follicle-stimulating hormone; FU, follow-up; h, hour; Hep B, hepatitis B virus; Hep C, hepatitis C virus; HIV, human immunodeficiency virus; ICANS, immune effector cell-associated neurotoxicity syndrome; ICF, informed consent form; IL 6, interleukin 6; INR, International normalized ratio; LD, lymphodepleting; LDH, lactate dehydrogenase; (LT)FU, (long-term) follow-up; m, month; qPCR, quantitative polymerase chain reaction; RNA-LPX, liposomally formulated ribonucleic acid encoding the vaccine; T3, triiodothyronine; T4, thyroxine; TSH, thyroid-stimulating hormone; (X), as clinically indicated.

1) All screening assessments must be completed prior to leukapheresis: completion of screening assessments within a 4-week window is preferred.
2) The patient will sign one pre-screening ICF for CLDN6 expression evaluation at the Pre-screening Visit and the main ICF at the Screening Visit.
3) Year of birth/age, sex, ethnicity, race.
4) All therapies (including medications, non-drug therapies) being taken by the patient from screening until 90 d after last IMP treatment will be regarded as prior or concomitant therapies (newly started or ongoing during this time period). Afterwards only therapies corresponding to related AEs will be recorded.
5) A complete physical examination will be performed at screening and the EoT Visit. At other visits, an abbreviated examination will be performed and new or worsened clinically significant abnormalities will be recorded. Patients should be examined daily when hospitalized.. All physical examinations should include the ICE score assessment.
6) Vital signs (systolic and diastolic blood pressure, heart rate, body temperature, and oxygen saturation) will be measured in a seated or recumbent position after at least 5 min rest (refer to Section 8.2.2), and should be checked daily when the patient is hospitalized.
7) LD chemotherapy should be completed at least three days prior to the CLDN6 CAR-T/CLDN6 CAR-T(A) infusion. Prior to LD chemotherapy initiation, infections, cardiac functions, glomerular filtration rate, and neurological assessments should be measured (refer to Section 6.1.1). Only if the criteria apply, can the LD chemotherapy be initiated.
8) Refer to Section 6.5.2 for information about documenting optional chemotherapy.
9) The FFPE sample should be from the most recent tumor tissue obtained. If no archival tumor tissue is available, the patient must be biopsied for CLDN6 staining at pre-screening. In addition, a baseline pre-treatment tumor sample (archival or fresh) is required.

10) At the Pre-screening Visit, patients must be biopsied if the most recent FFPE sample obtained is not available anymore. During the course of the trial fresh tumor biopsies should be taken at the discretion of the investigator, if this does not pose a significant risk for the patient..
11) Ascites and pleural effusion samples should be taken during the course of the trial at the discretion of the investigator, if this does not pose a significant risk for the patient..
12) Tumor assessment will be performed during screening, at 6 weeks (±7 d) post infusion, then every 6 weeks (±7 d) for 50 weeks and every 12 weeks (±7 d) thereafter. Tumor response will be evaluated according to RECIST v1.1 and iRECIST.
13) Tumor assessment will be performed at d-5, if screening was more than 4 weeks ago and in case of bridging chemotherapy.
14) All AEs will be reported from signing of the main ICF until 90 d after last IMP treatment. Afterwards only related AEs will be reported. Prior to signing of main ICF only SAEs related to a trial procedure will be reported.
15) At visit d-5, blood samples will be collected prior to the start of LD chemotherapy.
16) In case the lab test of special interest blood draw is not performed on the same visit as blood draw for hematology and clinical chemistry, blood sample for hematology and clinical chemistry need to be collected in addition.
17) Pregnancy tests will be performed for women of childbearing potential only. CAR-T dosing will only occur with a repeated negative pregnancy test (performed 5 d prior to CLDN6 CAR-T/CLDN6 CAR-T(A) administration).
18) FSH and estradiol tests will be performed at screening if indicated in post-menopausal females.

19) At visit d-5, blood samples will be collected prior to LD chemotherapy administration.
20) Collection of tumor tissue before treatment start, either the most recent archival FFPE material or a fresh biopsy is mandatory for molecular profiling (e.g., NGS, RNAseq, multiplex immuno-profiling).

Table 5: Standard Part 2 SoA - treatment, primary follow-up

Phase	Treatment and primary follow-up																				LTFU [1]
**Visit name**	**Infusion of CAR-T**	**CLDN6 RNA-LPX treatment phase**																		**Safety visit [3]**	**EoT & Primary FU**
**Trial period**	**d1**	**d2**	**d3**	**d4**	**d5 d6 d7**	**d8**	**d10**	**d17**	**d24 ±1d**	**d30-d35**	**d36, d43 ±1d**	**d51-d56**	**d57 ±1d**	**d72-d77**	**d78, d85, ±1d**	**d93-d98**	**d99 ±1d**	**d135, d177, d219, d261, d303, d345 2, d387, d429, d471, d513, d555, d597, d639, d681 ±7d**			**m25 ±14d**
**Patient history**																					
Prior/ concomitant therapies [4]	X	X	X	X	X	X	X	X	X	X	X	X	X	X	X	X	X	(X)		X	(X)
Body weight	X									d30										X	X
Physical examination [5]	X	X	X	X	X	X	X	X	X	X	X	X	X	X	X	X	X	X		X	X
Vital signs [6]	X	X	X	X	X	X	X	X	X	X	X	X	X	X	X	X	X	X		X	X
ECG	(X)	(X)	(X)	(X)	(X)	(X)	(X)	(X)	(X)	(X)	(X)	(X)	(X)	(X)	(X)	(X)	(X)	(X)		(X)	(X)
ECOG performance status	X	X	X					X		d30	d36	d51	X	d72	d85	d93	X	X		X	X
Hospitalization status [7]	d1-d14																				
**Intervention**																					
CLDN6 CAR-T/CLDN6 CAR-T(A) infusion [8]	X																				
Vaccination of CLDN6 RNA-LPX [9]				d4						d30		d51		d72		d93		Vaccinations every 6 weeks (±7d) until m22			
Documentation of antineoplastic therapy after CLDN6 infusion or trial discontinuation	X	X	X	X	X	X	X	X	X	X	X	X	X	X	X	X	X	X		X	X
**Disease assessments**																					
Fresh tumor biopsy [10]		As clinically indicated																			
Ascites/pleural effusion sample [11]		As clinically indicated																			

Phase	Treatment and primary follow-up																			LTFU [1]
**Visit name**	**Infusion of CAR-T**	**CLDN6 RNA-LPX treatment phase**																	**Safety visit [3]**	**EoT & Primary FU**
**Trial period**	**d1**	**d2**	**d3**	**d4**	**d5 d6 d7**	**d8**	**d10**	**d17**	**d24 ±1d**	**d30-d35**	**d36, d43 ±1d**	**d51-d56**	**d57 ±1d**	**d72-d77**	**d78, d85, ±1d**	**d93-d98**	**d99 ±1d**	**d135, d177, d219, d261, d303, d345 2, d387, d429, d471, d513, d555, d597, d639, d681 ±7d**		**m25 ±14d**
Tumor assessment [12]												Week 6 (±7d) post infusion, every 6 weeks (±7d) for 50 weeks, and every 12 weeks (±7d) thereafter								
**Safety**																				
Adverse events [13]	X	X	X	X	X	X	X	X	X	X	X	X	X	X	X	X	X	(X)	(X)	(X)
**Laboratory assessments**																				
Hematology (central lab) [14]	X (pre)			X (pre)		X	X	X	X	d30 (pre)	X	d51 (pre)	X	d72 (pre)	X	d93 (pre)	X	X	X	X
Clinical chemistry (central lab) [14]	X (pre)			X (pre)		X	X	X	X	d30 (pre)	X	d51 (pre)	X	d72 (pre)	X	d93 (pre)	X	X	X	X
Coagulation (central lab)	X (pre)																			X
Endocrine tests (TSH, T3, and T4) (central lab)										d30 (pre)						d93 (pre)		X	X	X
Lab test of special interest for CRS/ICANS (IL-6, CRP, ferritin, fibrinogen, procalcitonin,) (central lab) [15]	X (pre+6h post)	X	X	X (pre)		X	X	X	X	d30 (pre)	X	d51 (pre)	X	d72 (pre)	X	d93 (pre)	X			
Serum pregnancy test (local lab) [16]													X				X	X	X	X
**Biomarker [17]**																				
Cytokines [17,18]	X pre	X	X	X (pre, 6h, 24h)		X	X	X	X	d30 (pre, 6h, 24h)	X	d51 (pre, 6h, 24h)	X	d72, (pre, 6h, 24h)	d78 (±1d)	d93, (pre, 6h, 24h)	X	d135 (pre, 6h, 24h), d141, then every 6/7 weeks (±7d) [19]		

Phase	Treatment and primary follow-up																			LTFU [1]
Visit name	Infusion of CAR-T	CLDN6 RNA-LPX treatment phase																	Safety visit [3]	EoT & Primary FU
Trial period	d1	d2	d3	d4	d5 d6 d7	d8	d10	d17	d24 ±1d	d30-d35	d36, d43 ±1d	d51-d56	d57 ±1d	d72-d77	d78, d85, ±1d	d93-d98	d99 ±1d	d135, d177, d219, d261, d303, d345 2, d387, d429, d471, d513, d555, d597, d639, d681 ±7d		m25 ±14d
qPCR (incl. hematology) [17,19]		X	X	X (pre)		X	X	X	X	d30 (pre)	X	d51 (pre)	X	d72 (pre)	d78 (±1d)	d93 (pre)	X	d135 (pre), d141, then every 6/7 weeks (±7d) [20]		
Flow-phenotype [17,20]							X	X	X											
CAR characterization [17,21]								X			d43 (±1d)				d85 (±1d)					
Tumor biomarker [17,20]							X		X		d36 (±1d)	d51 (pre)		d72 (pre)		d93 (pre)		d135 (pre) (±7d)		
CAR immunogenicity assay [17,22]	X (pre)								X							d93 (pre)				
Tumor tissue (fresh) [23]								X												
**Survival status**	**For all patients who receive a CLDN6 CAR-T/CLDN6 CAR-T(A) infusion, patients are enrolled into the long-term follow-up period for survival status as per schedule in Appendix 8 after the EoT Visit. If a patient misses a scheduled visit where survival status is required, survival status can be obtained via phone contact.**																			

Abbreviations: AE, adverse event; CAR, chimeric antigen receptor; CAR-T, chimeric antigen receptor T cell; CLDN6, claudin 6; CRP, C-reactive protein; CRS, cytokine release syndrome; d, day; ECG, electrocardiogram; ECOG, Eastern Cooperative Oncology Group; EoT, End of Trial; FSH, follicle-stimulating hormone; FU, follow-up; h, hour; Hep B, hepatitis B virus; Hep C, hepatitis C virus; HIV, human immunodeficiency virus; ICANS, immune effector cell-associated neurotoxicity syndrome; ICF, informed consent form; IL 6, interleukin 6; INR, international normalized ratio; LDH, lactate dehydrogenase; (LT)FU, (long-term) follow-up; m, month; qPCR, quantitative polymerase chain reaction; RNA-LPX, liposomally formulated ribonucleic acid encoding the vaccine; T3, triiodothyronine; T4, thyroxine; TSH, thyroid-stimulating hormone; (X), as clinically indicated.

1) Refer to LTFU appendix for follow-up after trial completion.
2) If at m12/d345 vaccinations cease, visits switch from every 6 weeks to quarterly (m15, m18, m21, m24).
3) All patients (if alive) who discontinue trial treatment will be evaluated at a Safety/EoT Follow-up Visit at least 90 d and no more than 97 d after the last trial treatment administration. This visit will replace a regular visit if it is in the same visit window.
4) All therapies (including medications, non-drug therapies) being taken by the patient from screening until 90 d after last IMP treatment will be regarded as prior or concomitant therapies (newly started or ongoing during this time period). Afterwards only therapies corresponding to related AEs will be recorded.

5) A complete physical examination will be performed at screening and the EoT Visit. At other visits, an abbreviated examination will be performed and new or worsened clinically significant abnormalities will be recorded. Patients should be examined daily when hospitalized. On CLDN6 RNA-LPX vaccination days, patients should be examined prior to and 4-6 h following vaccination. All physical examinations should include the ICE score; the ICE score must be assessed daily within the hospitalization period.
6) Vital signs (systolic and diastolic blood pressure, heart rate, body temperature, and oxygen saturation) will be measured in a seated or recumbent position after at least 5 min rest (refer to Section 8.2.2), and should be measured daily if the patient is hospitalized. On CLDN6 RNA-LPX vaccination days, vital signs should be checked prior to and 4-6 h following vaccination.
7) Hospitalization can be shortened to d7 at the discretion of the SRC in case the patient shows no signs of CRS/ICANS. Thereafter, after each administration of CLDN6 RNA-LPX, the patient will be hospitalized as needed, but at least until improvement of any adverse event to Grade 2 or lower. Prolonged hospitalization is allowed at the discretion of the investigator.
8) The CAR-T dose will be administered by i.v. infusion. At visit d1, assessments according to Section 6.1.2.2 should be made before CLDN6 CAR-T/CLDN6 CAR-T(A) administration. Only if the criteria apply, can the infusion of CLDN6 CAR-T/CLDN6 CAR-T(A) take place.
9) The vaccination will be administered as a slow i.v. bolus injection. Further vaccinations after d93 are given every 6 weeks until m22. Refer to Section 6.1.3 for further information.
10) At the Pre-screening Visit, patients must be biopsied if the most recent FFPE sample obtained is not available anymore. During the course of the trial fresh tumor biopsies should be taken at the discretion of the investigator, if this does not pose a significant risk for the patient.
11) Ascites and pleural effusion samples should be taken during the course of the trial at the discretion of the investigator, if this does not pose a significant risk for the patient.
12) Tumor assessment will be performed during screening, at 6 weeks (±7 d) post infusion, then every 6 weeks (±7 d) for 50 weeks and every 12 weeks (±7 d) thereafter. Tumor response will be evaluated according to RECIST v1.1 and iRECIST.
13) All AEs will be reported from signing of the main ICF until 90 d after last IMP treatment. Afterwards only related AEs will be reported. Prior to signing of main ICF only SAEs related to a trial procedure will be reported.
14) At visit d1, blood samples will be collected prior to infusion of CAR-T. On RNA-LPX vaccination visits, blood samples will be collected prior to vaccination (blood collection on a weekend only in case of a CRS/ICANS).
15) At visit d1 blood samples will be collected prior to infusion of CAR-T, and 6 h (±1 h) post infusion. If CRS/ICANS is suspected, the parameters should be assessed daily by the central laboratory for the whole duration of the CRS/ICANS. As soon as the CRS/ICANS is over, parameters will be analyzed at the visits indicated in the SoA again (refer to Section 6.6.4.1).
16) Pregnancy tests will be performed for women of childbearing potential only. CAR-T dosing will only occur with a repeated negative pregnancy test (performed 5 d prior to CLDN6 CAR-T/CLDN6 CAR-T(A) administration).
17) After one year: The indicated biomarker assessments will only be performed if clinically indicated (after discussion with sponsor) – if CLDN6 CAR-T/CLDN6 CAR-T(A) are not detectable, biomarker assessments would cease with the exception of the qPCR assessment, and tumor biomarkers where relevant.
18) At visit d1, blood samples will be collected prior to infusion of CAR-T. On RNA-LPX administration visits, blood samples will be collected prior to and 6h and 24 h after administration of RNA-LPX. After d141, every 6 weeks (pre-, 6 h, 24 h, and 1 week after) RNA-LPX vaccination, if no RNA-LPX vaccination, then together with tumor assessment.
19) On RNA-LPX administration visits, blood samples will be collected prior to administration of RNA-LPX. A blood tube for hematology assessments must be taken with all samples for qPCR, if not already specified for this time point in the SoA. After d141, every 6 weeks (pre- and 1 week after) RNA-LPX vaccination, if no RNA-LPX vaccination, then together with tumor assessment.
20) On RNA-LPX administration visits, blood samples will be collected prior to administration of RNA-LPX.
21) For CAR characterization, the second and third time points should be aligned with the tumor assessments at 6 and 12 weeks. Up to three unscheduled additional blood sample collections are allowed if indicated by qPCR.
22) At visit d1, blood samples will be collected prior to CAR-T infusion.
23) An on treatment fresh tumor biopsy should be taken at the discretion of the investigator, if this does not pose a significant risk for the patient, ideally around d17 and at progression for molecular profiling and to determine CLDN6 expression and CAR-T infiltration into the tumor tissue.

1.3.4 Optional Part 2 SoA (CLDN6 CAR-T/CLDN6 CAR-T(A) + CLDN6 RNA-LPX)

Table 6: Optional Part 2 SoA - pre-screening, screening, apheresis, pre-treatment

Phase	Pre-screening for CLDN6 expression	Screening [1]	Apheresis		Pre-treatment		
Visit name			Leukapheresis	Post-leukapheresis FU	LD chemotherapy		
Trial period	Before screening	d-56 to d-18 [1]	d-18 (or earlier)	Within 3 d of leukapheresis	d-5	d-4	d-3
Informed consent [2]	X	X					
**Patient history**							
Inclusion/exclusion criteria		X					
Demography [3]	X	X					
Medical history		X					
Diagnosis and extent of cancer	X	X					
Prior antineoplastic therapies		X					
Prior/concomitant therapies [4]		X	X	X	X	X	X
Height		X					
Body weight		X					
Physical examination [5]		X	X	X	X	X	X
Vital signs [6]		X	X		X	X	X
ECG		X			(X)	(X)	(X)
ECOG performance status		X	X				
Eligibility		X					
**Intervention**							
Leukapheresis screening (according to local standards)		X					
Leukapheresis			X				

Phase	Pre-screening for CLDN6 expression	Screening [1]	Apheresis		Pre-treatment		
Visit name			Leukapheresis	Post-leukapheresis FU	LD chemotherapy		
Trial period	Before screening	d-56 to d-18 [1]	d-18 (or earlier)	Within 3 d of leukapheresis	d-5	d-4	d-3
LD chemotherapy [7]					X	X	X
Optional chemotherapy [8]		As clinically indicated					
**Disease assessments**							
Archival tumor tissue [9]	X						
Fresh biopsy [10]	(X)	As clinically indicated					
Ascites/pleural effusion sample [11]		As clinically indicated					
Tumor assessment [12]		X			X [13]		
**Safety**							
Adverse events [14]		X	X	X	X	X	X
**Laboratory assessments**							
Serology/virology (HIV test, Hep B+C) (central lab)		X					
Hematology (central lab) [15]		X			X		
Clinical chemistry (central lab) [15]		X			X		
Coagulation (central lab)		X					
Endocrine tests (TSH, T3, and T4) (central lab)		X					
Lab test of special interest for CRS/ICANS (IL-6, CRP, ferritin, fibrinogen,					X		

Phase	Pre-screening for CLDN6 expression	Screening [1]	Apheresis		Pre-treatment		
**Visit name**			**Leukapheresis**	**Post-leukapheresis FU**	**LD chemotherapy**		
**Trial period**	**Before screening**	**d-56 to d-18 [1]**	**d-18 (or earlier)**	**Within 3 d of leukapheresis**	**d-5**	**d-4**	**d-3**
procalcitonin) (central lab) [15,16]							
Urinalysis (local lab)		X					
Serum pregnancy test (local lab) [17]		X			X		
FSH and estradiol tests (central lab) [18]		X					
**Biomarker**							
Flow-phenotype [19]					X (pre)		
Tumor biomarker [19]					X (pre)		
Tumor tissue (molecular profiling) [20]		X					
HLA typing		X					

Abbreviations: AE, adverse event; CAR, chimeric antigen receptor; CAR-T, chimeric antigen receptor T cell; CLDN6, claudin 6; CRP, C-reactive protein; CRS, cytokine release syndrome; d, day; ECG, electrocardiogram; ECOG, Eastern Cooperative Oncology Group; EoT, End of Trial; FSH, follicle-stimulating hormone; FU, follow-up; h, hour; Hep B, hepatitis B virus; Hep C, hepatitis C virus; HIV, human immunodeficiency virus; ICANS, immune effector cell-associated neurotoxicity syndrome; ICF, informed consent form; IL 6, interleukin 6; INR, International normalized ratio; LD, lymphodepleting; LDH, lactate dehydrogenase; (LT)FU, (long-term) follow-up; m, month; qPCR, quantitative polymerase chain reaction; RNA-LPX, liposomally formulated ribonucleic acid encoding the vaccine; T3, triiodothyronine; T4, thyroxine; TSH, thyroid-stimulating hormone; (X), as clinically indicated.

1) All screening assessments must be completed prior to leukapheresis: completion of screening assessments within a 4-week window is preferred.
2) The patient will sign one pre-screening ICF for CLDN6 expression evaluation at the Pre-screening Visit and the main ICF at the Screening Visit.
3) Year of birth/age, sex, ethnicity, race.
4) All therapies (including medications, non-drug therapies) being taken by the patient from screening until 90 d after last IMP treatment will be regarded as prior or concomitant therapies (newly started or ongoing during this time period). Afterwards only therapies corresponding to related AEs will be recorded.
5) A complete physical examination will be performed at screening and the EoT Visit. At other visits, an abbreviated examination will be performed and new or worsened clinically significant abnormalities will be recorded. Patients should be examined daily when hospitalized.. All physical examinations should include the ICE score assessment.
6) Vital signs (systolic and diastolic blood pressure, heart rate, body temperature, and oxygen saturation) will be measured in a seated or recumbent position after at least 5 min rest (refer to Section 8.2.2), and should be checked daily when the patient is hospitalized.
7) LD chemotherapy should be completed at least three days prior to the CLDN6 CAR-T/CLDN6 CAR-T(A) infusion. Prior to LD chemotherapy initiation, infections, cardiac functions, glomerular filtration rate, and neurological assessments should be measured (refer to Section 6.1.1). Only if the criteria apply, can the LD chemotherapy be initiated.
8) Refer to Section 6.5.2 for information about documenting optional chemotherapy.
9) The FFPE sample should be from the most recent tumor tissue obtained. If no archival tumor tissue is available, the patient must be biopsied for CLDN6 staining at pre-screening. In addition, a baseline pre-treatment tumor sample (archival or fresh) is required.

10) At the Pre-screening Visit, patients must be biopsied if the most recent FFPE sample obtained is not available anymore. During the course of the trial fresh tumor biopsies should be taken at the discretion of the investigator, if this does not pose a significant risk for the patient..
11) Ascites and pleural effusion samples should be taken during the course of the trial at the discretion of the investigator, if this does not pose a significant risk for the patient..
12) Tumor assessment will be performed during screening, at 6 weeks (±7 d) post infusion, then every 6 weeks (±7 d) for 50 weeks and every 12 weeks (±7 d) thereafter. Tumor response will be evaluated according to RECIST v1.1 and iRECIST.
13) Tumor assessment will be performed at d-5, if screening was more than 4 weeks ago and in case of bridging chemotherapy.
14) All AEs will be reported from signing of the main ICF until 90 d after last IMP treatment. Afterwards only related AEs will be reported. Prior to signing of main ICF only SAEs related to a trial procedure will be reported.
15) At visit d-5, blood samples will be collected prior to the start of LD chemotherapy.
16) In case the lab test of special interest blood draw is not performed on the same visit as blood draw for hematology and clinical chemistry, blood sample for hematology and clinical chemistry need to be collected in addition.
17) Pregnancy tests will be performed for women of childbearing potential only. CAR-T dosing will only occur with a repeated negative pregnancy test (performed 5 d prior to CLDN6 CAR-T/CLDN6 CAR-T(A) administration).
18) FSH and estradiol tests will be performed at screening if indicated in post-menopausal females.

19) At visit d-5, blood samples will be collected prior to LD chemotherapy administration.
20) Collection of tumor tissue before treatment start, either the most recent archival FFPE material or a fresh biopsy is mandatory for molecular profiling (e.g., NGS, RNAseq, multiplex immuno-profiling).

Table 7: Optional Part 2 SoA - treatment, primary follow-up

Phase	Treatment and primary follow-up																			LTFU [1]
**Visit name**	**Infusion of CAR-T**	**CLDN6 RNA-LPX treatment phase**																	**Safety visit [3]**	**EoT & Primary FU**
**Trial period**	**d1**	**d2**	**d3**	**d4**	**d5 d6 d7**	**d8**	**d10**	**d17**	**d24 ±1d**	**d30-d35**	**d36, d43 ±1d**	**d51-d56**	**d57 ±1d**	**d72-d77**	**d78, d85, ±1d**	**d93-d98**	**d99 ±1d**	**d135, d177, d219, d261, d303, d345 2, d387, d429, d471, d513, d555, d597, d639, d681 ±7d**		**m25 ±14d**
**Patient history**																				
Prior/ concomitant therapies [4]	X	X	X	X	X	X	X	X	X	X	X	X	X	X	X	X	X	(X)	X	(X)
Body weight	X									d30									X	X
Physical examination [5]	X	X	X	X	X	X	X	X	X	X	X	X	X	X	X	X	X	X	X	X
Vital signs [6]	X	X	X	X	X	X	X	X	X	X	X	X	X	X	X	X	X	X	X	X
ECG	(X)	(X)	(X)	(X)	(X)	(X)	(X)	(X)	(X)	(X)	(X)	(X)	(X)	(X)	(X)	(X)	(X)	(X)	(X)	(X)
ECOG performance status	X	X	X					X		d30	d36	d51	X	d72	d85 (±1d)	d93	X	X	X	X
Hospitalization status [7]	d1-d14																			
**Intervention**																				
CLDN6 CAR-T/CLDN6 CAR-T(A) infusion [8]	X																			
Vaccination of CLDN6 RNA-LPX [9]									X		d36	d51		d72		d93		Vaccinations every 6 weeks (±7d) until m22		
Documentation of antineoplastic therapy after CLDN6 infusion or trial discontinuation	X	X	X	X	X	X	X	X	X	X	X	X	X	X	X	X	X	X	X	X
**Disease assessments**																				
Fresh tumor biopsy [10]		As clinically indicated																		
Ascites/pleural effusion sample [11]		As clinically indicated																		

**Phase**	**Treatment and primary follow-up**																			**LTFU [1]**
**Visit name**	**Infusion of CAR-T**	**CLDN6 RNA-LPX treatment phase**																	**Safety visit [3]**	**EoT & Primary FU**
**Trial period**	**d1**	**d2**	**d3**	**d4**	**d5 d6 d7**	**d8**	**d10**	**d17**	**d24 ±1d**	**d30-d35**	**d36, d43 ±1d**	**d51-d56**	**d57 ±1d**	**d72-d77**	**d78, d85, ±1d**	**d93-d98**	**d99 ±1d**	**d135, d177, d219, d261, d303, d345 2, d387, d429, d471, d513, d555, d597, d639, d681 ±7d**		**m25 ±14d**
Tumor assessment [12]												Week 6 (±7d) post infusion, every 6 weeks (±7d) for 50 weeks, and every 12 weeks (±7d) thereafter								
**Safety**																				
Adverse events [13]	X	X	X	X	X	X	X	X	X	X	X	X	X	X	X	X	X	(X)	(X)	(X)
**Laboratory assessments**																				
Hematology (central lab) [14]	X (pre)			X		X	X	X	X (pre)	d30	d36 (pre), d43 (±1d)	d51 (pre)	X	d72 (pre)	X	d93 (pre)	X	X	X	X
Clinical chemistry (central lab) [14]	X (pre)			X		X	X	X	X (pre)	d30	d36 (pre), d43 (±1d)	d51 (pre)	X	d72 (pre)	X	d93 (pre)	X	X	X	X
Coagulation (central lab)	X (pre)																			X
Endocrine tests (TSH, T3, and T4) (central lab)										d30						d93 (pre)		X	X	X
Lab test of special interest for CRS/ICANS (IL-6, CRP, ferritin, fibrinogen, procalcitonin,) (central lab) [15]	X (pre+6h post)	X	X	X		X	X	X	X (pre)	d30	d36 (pre), d43 (±1d)	d51 (pre)	X	d72 (pre)	X	d93 (pre)	X			
Serum pregnancy test (local lab) [16]													X				X	X	X	X
**Biomarker [17]**																				
Cytokines [17,18]	X pre	X	X	X		X	X	X	X (pre, 6h, 24h)	d30	d36 (pre),	d51 (pre,	X	d72, (pre,	d78 (±1d)	d93, (pre,	X	d135 (pre, 6h, 24h), d141, then every 6/7 weeks (±7d) [19]		

Phase	Treatment and primary follow-up																			LTFU [1]
**Visit name**	**Infusion of CAR-T**	**CLDN6 RNA-LPX treatment phase**																	**Safety visit [3]**	**EoT & Primary FU**
**Trial period**	**d1**	**d2**	**d3**	**d4**	**d5 d6 d7**	**d8**	**d10**	**d17**	**d24 ±1d**	**d30-d35**	**d36, d43 ±1d**	**d51-d56**	**d57 ±1d**	**d72-d77**	**d78, d85, ±1d**	**d93-d98**	**d99 ±1d**	**d135, d177, d219, d261, d303, d345 [2], d387, d429, d471, d513, d555, d597, d639, d681 ±7d**		**m25 ±14d**
											d43 (±1d)	6h, 24h)		6h, 24h)		6h, 24h)				
qPCR (incl. hematology) [17,19]		X	X	X		X	X	X	X (pre)	d30	d36 (pre), d43 (±1d)	d51 (pre)	X	d72 (pre)	d78 (±1d)	d93 (pre)	X	d135 (pre), d141, then every 6/7 weeks (±7d) [20]		
Flow-phenotype [17,20]							X	X	X (pre)											
CAR characterization [17,21]								X			d43 (±1d)				d85 (±1d)					
Tumor biomarker [17,20]							X		X (pre)		d36 (pre)	d51 (pre)		d72 (pre)		d93 (pre)		d135 (pre) (±7d)		
CAR immunogenicity assay [17,22]	X (pre)								X (pre)							d93 (pre)				
Tumor tissue (fresh) [23]								X												
**Survival status**	**For all patients who receive a CLDN6 CAR-T/CLDN6 CAR-T(A) infusion, patients are enrolled into the long-term follow-up period for survival status as per schedule in Appendix 8 after the EoT Visit. If a patient misses a scheduled visit where survival status is required, survival status can be obtained via phone contact.**																			

Abbreviations: AE, adverse event; CAR, chimeric antigen receptor; CAR-T, chimeric antigen receptor T cell; CLDN6, claudin 6; CRP, C-reactive protein; CRS, cytokine release syndrome; d, day; ECG, electrocardiogram; ECOG, Eastern Cooperative Oncology Group; EoT, End of Trial; FSH, follicle-stimulating hormone; FU, follow-up; h, hour; Hep B, hepatitis B virus; Hep C, hepatitis C virus; HIV, human immunodeficiency virus; ICANS, immune effector cell-associated neurotoxicity syndrome; ICF, informed consent form; IL 6, interleukin 6; INR, international normalized ratio; LDH, lactate dehydrogenase; (LT)FU, (long-term) follow-up; m, month; qPCR, quantitative polymerase chain reaction; RNA-LPX, liposomally formulated ribonucleic acid encoding the vaccine; T3, triiodothyronine; T4, thyroxine; TSH, thyroid-stimulating hormone; (X), as clinically indicated.

1) Refer to LTFU appendix for follow-up after trial completion.
2) If at m12/d345 vaccinations cease, visits switch from every 6 weeks to quarterly (m15, m18, m21, m24).
3) All patients (if alive) who discontinue trial treatment will be evaluated at a Safety/EoT Follow-up Visit at least 90 d and no more than 97 d after the last trial treatment administration. This visit will replace a regular visit if it is in the same visit window.
4) All therapies (including medications, non-drug therapies) being taken by the patient from screening until 90 d after last IMP treatment will be regarded as prior or concomitant therapies (newly started or ongoing during this time period). Afterwards only therapies corresponding to related AEs will be recorded.

5) A complete physical examination will be performed at screening and the EoT Visit. At other visits, an abbreviated examination will be performed and new or worsened clinically significant abnormalities will be recorded. Patients should be examined daily when hospitalized. On CLDN6 RNA-LPX vaccination days, patients should be examined prior to and 4-6 h following vaccination. All physical examinations should include the ICE score; the ICE score must be assessed daily within the hospitalization period.
6) Vital signs (systolic and diastolic blood pressure, heart rate, body temperature, and oxygen saturation) will be measured in a seated or recumbent position after at least 5 min rest (refer to Section 8.2.2), and should be measured daily if the patient is hospitalized. On CLDN6 RNA-LPX vaccination days, vital signs should be checked prior to and 4-6 h following vaccination.
7) Hospitalization can be shortened to d7 at the discretion of the SRC in case the patient shows no signs of CRS/ICANS. Thereafter, after each administration of CLDN6 RNA-LPX, the patient will be hospitalized as needed, but at least until improvement of any adverse event to Grade 2 or lower. Prolonged hospitalization is allowed at the discretion of the investigator.
8) The CAR-T dose will be administered by i.v. infusion. At visit d1, assessments according to Section 6.1.2.2 should be made before CLDN6 CAR-T/CLDN6 CAR-T(A) administration. Only if the criteria apply, can the infusion of CLDN6 CAR-T/CLDN6 CAR-T(A) take place.
9) The vaccination will be administered as a slow i.v. bolus injection. Further vaccinations after d93 are given every 6 weeks until m22. Refer to Section 6.1.3 for further information.
10) At the Pre-screening Visit, patients must be biopsied if the most recent FFPE sample obtained is not available anymore. During the course of the trial fresh tumor biopsies should be taken at the discretion of the investigator, if this does not pose a significant risk for the patient.
11) Ascites and pleural effusion samples should be taken during the course of the trial at the discretion of the investigator, if this does not pose a significant risk for the patient.
12) Tumor assessment will be performed during screening, at 6 weeks (±7 d) post infusion, then every 6 weeks (±7 d) for 50 weeks and every 12 weeks (±7 d) thereafter. Tumor response will be evaluated according to RECIST v1.1 and iRECIST.
13) All AEs will be reported from signing of the main ICF until 90 d after last IMP treatment. Afterwards only related AEs will be reported. Prior to signing of main ICF only SAEs related to a trial procedure will be reported.
14) At visit d1, blood samples will be collected prior to infusion of CAR-T. On RNA-LPX vaccination visits, blood samples will be collected prior to vaccination (blood collection on a weekend only in case of a CRS/ICANS).
15) At visit d1 blood samples will be collected prior to infusion of CAR-T, and 6 h (±1 h) post infusion. If CRS/ICANS is suspected, the parameters should be assessed daily by the central laboratory for the whole duration of the CRS/ICANS. As soon as the CRS/ICANS is over, parameters will be analyzed at the visits indicated in the SoA again (refer to Section 6.6.4.1).
16) Pregnancy tests will be performed for women of childbearing potential only. CAR-T dosing will only occur with a repeated negative pregnancy test (performed 5 d prior to CLDN6 CAR-T/CLDN6 CAR-T(A) administration).
17) After one year: The indicated biomarker assessments will only be performed if clinically indicated (after discussion with sponsor) – if CLDN6 CAR-T/CLDN6 CAR-T(A) are not detectable, biomarker assessments would cease with the exception of the qPCR assessment, and tumor biomarkers where relevant.
18) At visit d1, blood samples will be collected prior to infusion of CAR-T. On RNA-LPX administration visits, blood samples will be collected prior to and 6h and 24 h after administration of RNA-LPX. After d141, every 6 weeks (pre-, 6 h, 24 h, and 1 week after) RNA-LPX vaccination, if no RNA-LPX vaccination, then together with tumor assessment.
19) On RNA-LPX administration visits, blood samples will be collected prior to administration of RNA-LPX. A blood tube for hematology assessments must be taken with all samples for qPCR, if not already specified for this time point in the SoA. After d141, every 6 weeks (pre- and 1 week after) RNA-LPX vaccination, if no RNA-LPX vaccination, then together with tumor assessment.
20) On RNA-LPX administration visits, blood samples will be collected prior to administration of RNA-LPX.
21) For CAR characterization, the second and third time points should be aligned with the tumor assessments at 6 and 12 weeks. Up to three unscheduled additional blood sample collections are allowed if indicated by qPCR.
22) At visit d1, blood samples will be collected prior to CAR-T infusion.
23) An on treatment fresh tumor biopsy should be taken at the discretion of the investigator, if this does not pose a significant risk for the patient, ideally around d17 and at progression for molecular profiling and to determine CLDN6 expression and CAR-T infiltration into the tumor tissue.

1.3.5 Optional Part 2 SoA (CLDN6 CAR-T/CLDN6 CAR-T(A) + CLDN6 RNA-LPX without LD chemotherapy)

Table 8: Optional Part 2 SoA - pre-screening, screening, apheresis, pre-treatment (without LD chemotherapy)

Phase	Pre-screening for CLDN6 expression	Screening [1]	Apheresis	
Visit name			Leukapheresis	Post-leukapheresis FU
Trial period	Before screening	d-56 to d-18 [1]	d-18 (or earlier)	Within 3 d of leukapheresis
Informed consent [2]	X	X		
**Patient history**				
Inclusion/exclusion criteria		X		
Demography [3]	X	X		
Medical history		X		
Diagnosis and extent of cancer	X	X		
Prior antineoplastic therapies		X		
Prior/concomitant therapies [4]		X	X	X
Height		X		
Body weight		X		
Physical examination [5]		X	X	X
Vital signs [6]		X	X	
ECG		X		
ECOG performance status		X	X	
Eligibility		X		
**Intervention**				
Leukapheresis screening (according to local standards)		X		
Leukapheresis			X	
Optional chemotherapy [7]		As clinically indicated		
**Disease assessments**				
Archival tumor tissue [8]	X			

Phase	Pre-screening for CLDN6 expression	Screening [1]	Apheresis	
Visit name			Leukapheresis	Post-leukapheresis FU
Trial period	Before screening	d-56 to d-18 [1]	d-18 (or earlier)	Within 3 d of leukapheresis
Fresh biopsy [9]	(X)	As clinically indicated		
Ascites/pleural effusion sample [10]		As clinically indicated		
Tumor assessment [11]		X		
**Safety**				
Adverse events [12]		X	X	X
**Laboratory assessments**				
Serology/virology (HIV test, Hep B+C) (central lab)		X		
Hematology (central lab)		X		
Clinical chemistry (central lab)		X		
Coagulation (central lab)		X		
Endocrine tests (TSH, T3, and T4) (central lab)		X		
Urinalysis (local lab)		X		
Serum pregnancy test (local lab) [14]		X		
FSH and estradiol tests (central lab) [15]		X		
**Biomarker**				
Tumor tissue (molecular profiling) [16]		X		
HLA typing		X		

Abbreviations: AE, adverse event; CAR, chimeric antigen receptor; CAR-T, chimeric antigen receptor T cell; CLDN6, claudin 6; CRP, C-reactive protein; CRS, cytokine release syndrome; d, day; ECG, electrocardiogram; ECOG, Eastern Cooperative Oncology Group; EoT, End of Trial; FSH, follicle-stimulating hormone; FU, follow-up; h, hour; Hep B, hepatitis B virus; Hep C, hepatitis C virus; HIV, human immunodeficiency virus; ICANS, immune effector cell-associated neurotoxicity syndrome; ICF, informed consent form; IL 6, interleukin 6; INR, international normalized ratio; LD, lymphodepleting; LDH, lactate dehydrogenase; (LT)FU, (long-term) follow-up; m, month; qPCR, quantitative polymerase chain reaction; RNA-LPX, liposomally formulated ribonucleic acid encoding the vaccine; T3, triiodothyronine; T4, thyroxine; TSH, thyroid-stimulating hormone; (X), as clinically indicated.

1) All screening assessments must be completed prior to leukapheresis: completion of screening assessments within a 4-week window is preferred.
2) The patient will sign one pre-screening ICF for CLDN6 expression evaluation at the Pre-screening Visit and the main ICF at the Screening Visit.
3) Year of birth/age, sex, ethnicity, race.
4) All therapies (including medications, non-drug therapies) being taken by the patient from screening until 90 d after last IMP treatment will be regarded as prior or concomitant therapies (newly started or ongoing during this time period). Afterwards only therapies corresponding to related AEs will be recorded.
5) A complete physical examination will be performed at screening and the EoT Visit. At other visits, an abbreviated examination will be performed and new or worsened clinically significant abnormalities will be recorded. Patients should be examined daily when hospitalized. All physical examinations should include the ICE score.

6) Vital signs (systolic and diastolic blood pressure, heart rate, body temperature, and oxygen saturation) will be measured in a seated or recumbent position after at least 5 min rest (refer to Section 8.2.2), and should be measured daily if the patient is hospitalized.
7) Refer to Section 6.5.2 for information about documenting optional chemotherapy.
8) The FFPE sample should be from the most recent tumor tissue obtained. If no tumor tissue is available, the patient must be biopsied for CLDN6 staining at pre-screening. In addition, a baseline pre-treatment tumor sample (archival or fresh) is required.
9) At the Pre-screening Visit, patients must be biopsied if the most recent FFPE sample obtained is not available anymore. During the course of the trial fresh tumor biopsies should be taken at the discretion of the investigator, if this does not pose a significant risk for the patient.
10) Ascites and pleural effusion samples should be taken during the course of the trial at the discretion of the investigator, if this does not pose a significant risk for the patient.
11) Tumor assessment will be performed at d-5, if screening was more than 4 weeks ago and in case of bridging chemotherapy.
12) All AEs will be reported from signing of the main ICF until 90 d after last IMP treatment. Afterwards only related AEs will be reported. Prior to signing of main ICF only SAEs related to a trial procedure will be reported.
13) In case of CRS/ICANS, blood sample for hematology and clinical chemistry also need to be collected and analyzed.
14) Pregnancy tests will be performed for women of childbearing potential only. CAR-T dosing will only occur with a repeated negative pregnancy test (performed 5 d prior to CLDN6 CAR-T/CLDN6 CAR-T(A) administration).
15) FSH and estradiol tests will be performed at screening if indicated in post-menopausal females.
16) Collection of tumor tissue before treatment start, either the most recent archival FFPE material or a fresh biopsy is mandatory for molecular profiling (e.g., NGS, RNAseq, multiplex immuno-profiling).

Table 9: Optional Part 2 SoA - treatment, primary follow-up (without LD chemotherapy)

**Phase**	**Treatment and primary follow-up**																			**LTFU** [1]
**Visit name**	**Infusion of CAR-T**	**CLDN6 RNA-LPX treatment phase**																	**Safety Visit** [3]	**EoT & Primary FU**
**Trial period**	**d1**	**d2**	**d3**	**d4**	**d5 d6 d7**	**d8**	**d10**	**d17**	**d24 ±1d**	**d30-d35**	**d36, d43 ±1d**	**d51-d56**	**d57 ±1d**	**d72-d77**	**d78, d85, ±1d**	**d93-d98**	**d99 ±1d**	**d135, d177, d219, d261, d303, d345** [2]**, d387, d429, d471, d513, d555, d597, d639, d681 ±7d**		**m25 ±14d**
**Patient history**																				
Prior/ concomitant therapies [4]	X	X	X	X	X	X	X	X	X	X	X	X	X	X	X	X	X	(X)	X	(X)
Body weight	X									d30									X	X
Physical examination [5]	X	X	X	X	X	X	X	X	X	X	X	X	X	X	X	X	X	X	X	X
Vital signs [6]	X	X	X	X	X	X	X	X	X	X	X	X	X	X	X	X	X	X	X	X
ECG	(X)	(X)	(X)	(X)	(X)	(X)	(X)	(X)	(X)	(X)	(X)	(X)	(X)	(X)	(X)	(X)	(X)	(X)	(X)	(X)
ECOG performance status	X	X	X					X		d30	d36 (±1d)	d51	X	d72	d85 (±1d)	d93	X	X	X	X
Hospitalization status [7]	d1-d14																			
**Intervention**																				
CLDN6 CAR-T/CLDN6 CAR-T(A) infusion [8]	X																			
Vaccination of CLDN6 RNA-LPX [9]				d4						d30		d51		d72		d93		Vaccinations every 6 weeks (±7d) until m22		
Documentation of antineoplastic therapy after CLDN6 infusion or trial discontinuation	X	X	X	X	X	X	X	X	X	X	X	X	X	X	X	X	X	X	X	X
**Disease assessments**																				
Fresh tumor biopsy [10]		As clinically indicated																		
Ascites/pleural effusion sample [11]		As clinically indicated																		

**Phase**	**Treatment and primary follow-up**																			**LTFU [1]**
**Visit name**	**Infusion of CAR-T**	**CLDN6 RNA-LPX treatment phase**																	**Safety Visit [3]**	**EoT & Primary FU**
**Trial period**	**d1**	**d2**	**d3**	**d4**	**d5 d6 d7**	**d8**	**d10**	**d17**	**d24 ±1d**	**d30-d35**	**d36, d43 ±1d**	**d51-d56**	**d57 ±1d**	**d72-d77**	**d78, d85, ±1d**	**d93-d98**	**d99 ±1d**	**d135, d177, d219, d261, d303, d345 [2], d387, d429, d471, d513, d555, d597, d639, d681 ±7d**		**m25 ±14d**
Tumor assessment [12]	X [13]											W6 (±7d) post infusion, every 6 weeks (±7d) for 50 weeks, and every 12 weeks (±7d) thereafter								
**Safety**																				
Adverse events [14]	X	X	X	X	X	X	X	X	X	X	X	X	X	X	X	X	X	(X)	(X)	(X)
**Laboratory assessments**																				
Hematology (central lab) [15]	X (pre)	X	X	X (pre)	X	X	X	X	X	d30 (pre)	X	d51 (pre)	X	d72 (pre)	X	d93 (pre)	X	X	X	X
Clinical chemistry (central lab) [15]	X (pre)	X	X	X (pre)	X	X	X	X	X	d30 (pre)	X	d51 (pre),	X	d72, (pre)	X	d93 (pre)	X	X	X	X
Coagulation (central lab)	X (pre)																			X
Endocrine tests (TSH, T3, and T4) (central lab)										d30 (pre)						d93 (pre)		X	X	X
Lab test of special interest for CRS/ICANS (IL-6, CRP, ferritin, fibrinogen, procalcitonin) (central lab) [16]	X (pre, 6h)	X	X	X (pre)	X	X	X	X	X	d30 (pre)	X	d51 (pre)	X	d72 (pre)	X	d93 (pre)	X			
Serum pregnancy test (local lab) [17]													X				X	X	X	X
**Biomarker [18]**																				
Cytokines [18,19]	X (pre)	X	X	X (pre, 6h, 24h)		X	X	X	X	d30 (pre, 6h, 24h)	d36 (±1d)	d51 (pre, 6h, 24h)	X	d72 (pre, 6h, 24h)	d78 (±1d)	d93 (pre, 6h, 24h)	X	d135 (pre, 6h, 24h), d141 (±7d)		
qPCR (incl. hematology) [18,20]		X	X	X (pre)		X	X	X	X	d30 (pre)	X	d51 (pre)	X	d72 (pre)	d78 (±1d)	d93 (pre)	X	d135 (pre), d141 (±7d)		

**Phase**	**Treatment and primary follow-up**																			**LTFU** [1]
**Visit name**	**Infusion of CAR-T**	**CLDN6 RNA-LPX treatment phase**																	**Safety Visit** [3]	**EoT & Primary FU**
**Trial period**	**d1**	**d2**	**d3**	**d4**	**d5 d6 d7**	**d8**	**d10**	**d17**	**d24 ±1d**	**d30-d35**	**d36, d43 ±1d**	**d51-d56**	**d57 ±1d**	**d72-d77**	**d78, d85, ±1d**	**d93-d98**	**d99 ±1d**	**d135, d177, d219, d261, d303, d345** [2]**, d387, d429, d471, d513, d555, d597, d639, d681 ±7d**		**m25 ±14d**
Flow-phenotype [18,21]	X (pre)						X	X	X											
CAR characterization [18,22]								X			d43 (±1d)				d85 (±1d)					
Tumor biomarker [18,21]	X (pre)						X		X	d30 (pre)		d51 (pre)		d72 (pre)		d93 (pre)		d135 (pre)		
CAR immunogenicity assay [18,23]	X (pre)								X								X			
Tumor tissue (fresh) [24]		As clinically indicated																		
**Survival status**	**For all patients who receive a CLDN6 CAR-T/CLDN6 CAR-T(A) infusion, patients are enrolled into the long-term follow-up period for survival status as per schedule in Appendix 8 after the EoT Visit. If a patient misses a scheduled visit where survival status is required, survival status can be obtained via phone contact.**																			

Abbreviations: AE, adverse event; CAR, chimeric antigen receptor; CAR-T, chimeric antigen receptor T cell; CLDN6, claudin 6; CRP, C-reactive protein; CRS, cytokine release syndrome; d, day; ECG, electrocardiogram; ECOG, Eastern Cooperative Oncology Group; EoT, End of Trial; FSH, follicle-stimulating hormone; FU, follow-up; h, hour; Hep B, hepatitis B virus; Hep C, hepatitis C virus; HIV, human immunodeficiency virus; ICANS, immune effector cell-associated neurotoxicity syndrome; ICF, informed consent form; IL 6, interleukin 6; INR, international normalized ratio; LDH, lactate dehydrogenase; (LT)FU, (long-term) follow-up; m, month; qPCR, quantitative polymerase chain reaction; RNA-LPX, liposomally formulated ribonucleic acid encoding the vaccine; T3, triiodothyronine; T4, thyroxine; TSH, thyroid-stimulating hormone; (X), as clinically indicated.

1) Refer to LTFU appendix for follow-up after trial completion.
2) If at m12/d345 vaccinations cease, visits switch from every 6 weeks to quarterly (m15, m18, m21, m24).
3) All patients (if alive) who discontinue trial treatment will be evaluated at a Safety/EoT Follow-up Visit at least 90 d and no more than 97 d after the last trial treatment administration. This visit will replace a regular visit if it is in the same visit window.
4) All therapies (including medications, non-drug therapies) being taken by the patient from screening until 90 d after last IMP treatment will be regarded as prior or concomitant therapies (newly started or ongoing during this time period). Afterwards only therapies corresponding to related AEs will be recorded.
5) A complete physical examination will be performed at screening and the EoT Visit. At other visits, an abbreviated examination will be performed and new or worsened clinically significant abnormalities will be recorded. Patients should be examined daily when hospitalized. On CLDN6 RNA-LPX vaccination days, patients should be examined prior to and 4-6 h following vaccination. All physical examinations should include the ICE score; the ICE score must be assessed daily within the hospitalization period.
6) Vital signs (systolic and diastolic blood pressure, heart rate, body temperature, and oxygen saturation) will be measured in a seated or recumbent position after at least 5 min rest (refer to Section 8.2.2), and should be measured daily if the patient is hospitalized. On CLDN6 RNA-LPX vaccination days, vital signs should be checked prior to and 4-6 h, following vaccination.

7) Hospitalization can be shortened to d7 at the discretion of the SRC in case the patient shows no signs of CRS/ICANS. Thereafter, after each administration of CLDN6 RNA-LPX, the patient will be hospitalized as needed, but at least until improvement of any adverse event to Grade 2 or lower. Prolonged hospitalization is allowed at the discretion of the investigator.

8) The CAR-T dose will be administered by i.v. infusion. At visit d1, assessments according to Section 6.1.2.2 should be made before CLDN6 CAR-T/CLDN6 CAR-T(A) administration. Only if the criteria apply, can the infusion of CLDN6 CAR-T/CLDN6 CAR-T(A) take place.

9) The vaccination will be administered as a slow i.v. bolus injection. Further vaccinations after d93 are given every 6 weeks until m22. Refer to Section 6.1.3 for further information.

10) At the Pre-screening Visit, patients must be biopsied if the most recent FFPE sample obtained is not available anymore. During the course of the trial fresh tumor biopsies should be taken at the discretion of the investigator, if this does not pose a significant risk for the patient.

11) Ascites and pleural effusion samples should be taken during the course of the trial at the discretion of the investigator, if this does not pose a significant risk for the patient.

12) Tumor assessment will be performed during screening, at 6 weeks (±7 d) post infusion, then every 6 weeks (±7 d) for 50 weeks and every 12 weeks (±7 d) thereafter. Tumor response will be evaluated according to RECIST v1.1 and iRECIST.

13) Tumor assessment will be performed prior to or at d1, if screening was more than 4 weeks ago and in case of bridging chemotherapy.

14) All AEs will be reported from signing of the main ICF until 90 d after last IMP treatment. Afterwards only related AEs will be reported. Prior to signing of main ICF only SAEs related to a trial procedure will be reported.

15) At visit d1, blood samples will be collected prior to infusion of CAR-T. On RNA-LPX vaccination visits, blood samples will be collected prior to vaccination (blood collection on a weekend only in case of a CRS/ICANS).

16) At visit d1 blood samples will be collected prior to infusion of CAR-T, and 6 h (±1 h) post infusion. On RNA-LPX vaccination visits, blood samples will be collected prior to vaccination. If CRS/ICANS is suspected, the parameters should be assessed daily by the central laboratory for the whole duration of the CRS/ICANS. As soon as the CRS/ICANS is over, parameters will be analyzed at the visits indicated in the SoA again (refer to Section 6.6.4.1).

17) Pregnancy tests will be performed for women of childbearing potential only. CAR-T dosing will only occur with a repeated negative pregnancy test (performed 5 d prior to CLDN6 CAR-T/CLDN6 CAR-T(A) administration).

18) After one year: The indicated biomarker assessments will only be performed if clinically indicated (after discussion with sponsor) – if CLDN6 CAR-T/CLDN6 CAR-T(A) are not detectable, biomarker assessments would cease with the exception of the qPCR assessment, and tumor biomarkers where relevant.

19) At visit d1, blood samples will be collected prior to infusion of CAR-T. On RNA-LPX administration visits, blood samples will be collected prior to, and 6h, and 24 h after administration of RNA-LPX. After d141, every 6 weeks (pre- and 6 h, 24 h, and 1 week) after) RNA-LPX vaccination, if no RNA-LPX vaccination, then together with tumor assessment.

20) On RNA-LPX administration visits, blood samples will be collected prior to administration of RNA-LPX. A blood tube for hematology assessments must be taken with all samples for qPCR, if not already specified for this time point in the SoA. After d141, every 6 weeks (pre-, and 1 week) after) RNA-LPX vaccination, if no RNA-LPX vaccination, then together with tumor assessment.

21) At visit d1, blood samples will be collected prior to infusion of CAR-T.

22) For CAR characterization, the second and third time points should be aligned with the tumor assessments at 6 and 12 weeks. Up to three unscheduled additional blood sample collections are allowed if indicated by qPCR.

23) At visit d1, blood samples will be collected prior to infusion of CAR-T.

24) An on treatment fresh tumor biopsy should be taken at the discretion of the investigator, if this does not pose a significant risk for the patient, ideally around d17 and at progression for molecular profiling and to determine CLDN6 expression and CAR-T infiltration into the tumor tissue.

2 INTRODUCTION

Chimeric antigen receptors (CARs) are engineered receptors that combine a single chain variable fragment (scFv) derived from a monoclonal antibody with a spacer domain and an intracellular part consisting of one or more signaling domains for T cell activation. CARs redirect T cells to eradicate tumors through specific recognition of native surface proteins expressed on tumor cells in a non-MHC-restricted manner. Therefore, CAR-T can be used for the treatment of all individuals whose tumor expresses the respective target, independent of the individual's human leukocyte antigen genotype.

CAR-T therapy has shown potent anti-tumor responses, especially in patients with B-cell malignancies (Neelapu et al. 2017; Maude et al. 2018). The clinical efficacy of CAR-T approaches in solid tumors, however, has so far been limited (summarized in Table 16).

Two key hurdles of CAR-T therapy in solid cancers are (i) the lack of highly cancer-cell selective targets for high precision targeting and, (ii) the insufficient expansion and lack of long-term persistence of CAR-T *in vivo*. The latter is presumably attributable to the fact that adoptively transferred CAR-T against solid cancer targets do not encounter their antigens in the patient´s circulation, which would provide survival and expansion signals. In hematological malignancies CAR-T are directed against lineage antigens of B cells and encounter their targets on the host's normal and malignant B cells. These act as APCs providing strong proliferation signals and promote persistence of CAR-T (Kalos et al. 2011; Porter et al. 2015).

In contrast, the frequency of CAR-T against solid tumors typically declines rapidly (Gargett et al. 2016; Feng et al. 2016; O'Rourke et al. 2017) due to the impaired accessibility of tumor cells within solid lesions and the absence of co-stimulation when CAR-T encounter their target in the context of an immunosuppressive tumor microenvironment.

The sponsor has developed a targeted immunotherapy, which addresses the main challenges of CAR-T therapies in solid tumors, by combining:

1) autologous T cells genetically engineered to express a 2nd generation CAR directed against the exquisitely cancer cell-specific antigen claudin-6 (CLDN6); and
2) controlled *in vivo* expansion of adoptively transferred CAR-T using liposome-formulated RNA (RNA-LPX) encoding the CAR target antigen.

A schematic overview of the treatment concept is depicted in Figure 6.

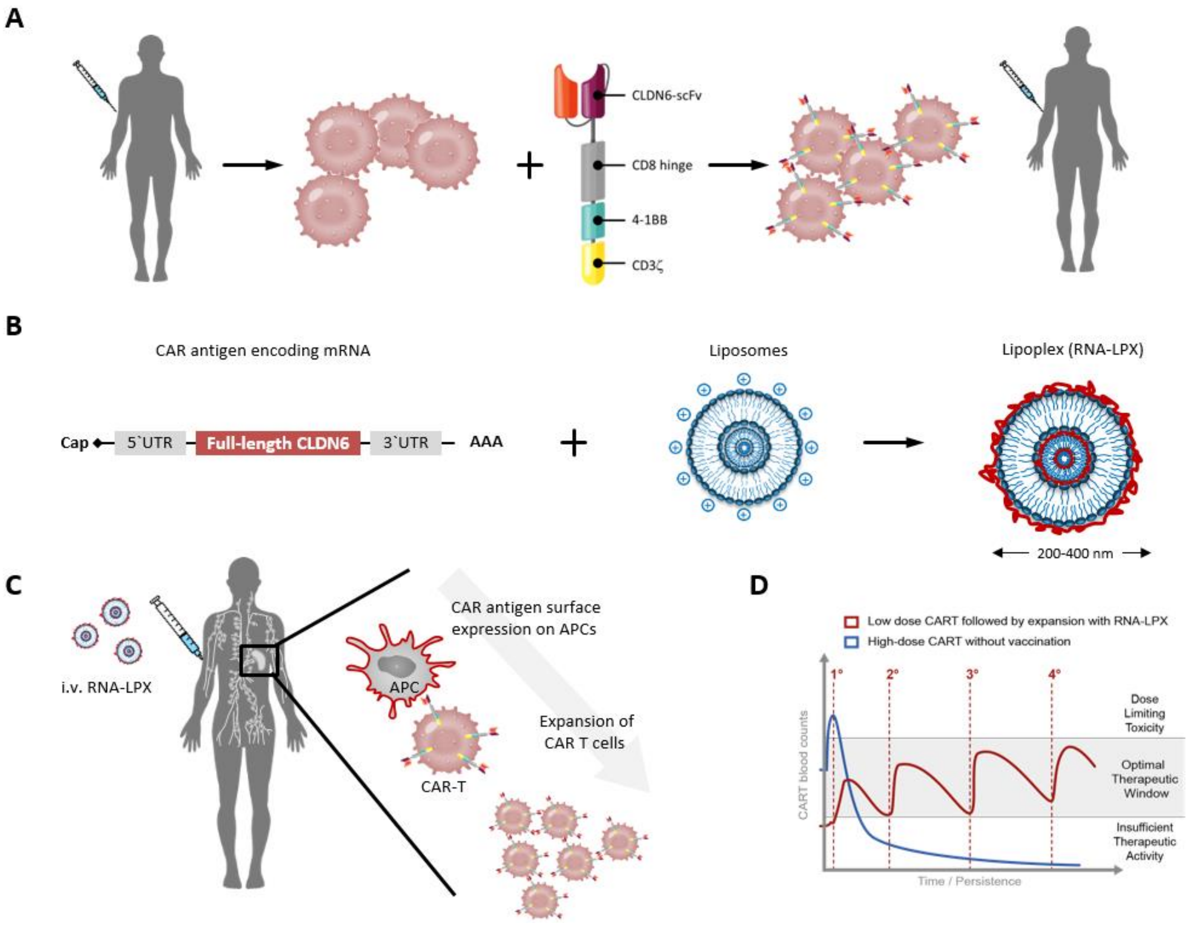

Figure 6: Concept of CLDN6 CAR-T/CLDN6 CAR-T(A) therapy combined with RNA-LPX-mediated *in vivo* expansion of CAR-T

Abbreviations: APC, antigen-presenting- cell; CAR, chimeric antigen receptor; CAR-T, chimeric antigen receptor T cell; CLDN6, claudin 6; RNA-LPX, liposome-formulated ribonucleic acid.

(A) Autologous T cells are engineered to express the CLDN6-CAR using retroviral transduction and are reinfused into the lymphodepleted patient. (B) Full-length CLDN6-encoding RNA is complexed with liposomes to form lipoplexes (RNA-LPX). (C) Intravenously administered RNA-LPX selectively targets APCs in secondary lymphoid organs, which leads to uptake, antigen expression and maturation of APCs. Exposure of CAR-T to their target in the context of co-stimulation provided by APCs in secondary lymphatic organs results in CAR-T expansion *in vivo*. (D) CAR target antigen-encoding RNA-LPX can be repeatedly administered to achieve controlled expansion and persistence of CAR-T *in vivo*.

Preclinical proof-of-concept studies in mice demonstrated that the combination of CLDN6 CAR-T/CLDN6 CAR-T(A) with RNA-LPX mediated controlled *in vivo* expansion improved CAR-T persistence and anti-tumor efficacy and, thus, constitutes an innovative treatment concept for the specific and effective treatment of CLDN6-expressing tumors. Therefore, the sponsor will clinically implement the concept in this Phase I/IIa trial evaluating the safety and tolerability of CLDN6 CAR-T/CLDN6 CAR-T(A) with or without RNA-LPX (with or without prior lymphodepleting (LD) chemotherapy) in patients with CLDN6 high expressing relapsed or refractory advanced solid tumors.

2.1 CLDN6 AS TARGET FOR CAR-T THERAPY

CLDN6, a tetraspan membrane protein that is involved in tight junction formation (Turksen and Troy 2001), is as a strictly oncofetal cell surface antigen with an ideal expression profile for CAR-T targeting. In humans, CLDN6 protein is only expressed during early organogenesis in fetal development and silenced in adult healthy tissues (Turksen and Troy 2004). Consequently, it was not detectable in any of the analyzed adult human normal tissue types assessed by IHC staining (Section 2.1.1). In contrast, silencing of expression in mice is less complete and some adult tissues do still express CLDN6 (Abuazza et al. 2006; Matsubara et al. 2012).

In line with previous studies (Ushiku et al. 2012; Micke et al. 2014), high CLDN6 transcript levels were frequent in various human solid cancers such as testicular, ovarian, endometrial and lung adenocarcinoma (Section 2.1.2).

The CLDN6 molecule is schematically represented in Figure 7 and characteristics of CLDN6 are summarized in Table 10. For detailed information please also see the Investigator's Brochure (IB).

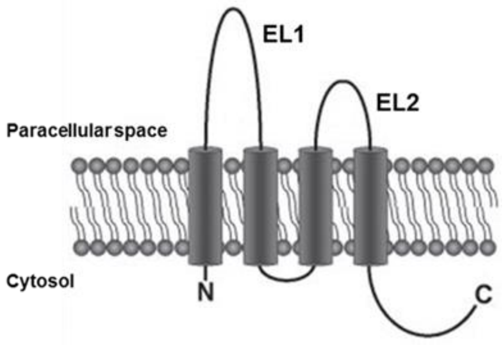

Figure 7: Schematic representation of CLDN6

Source: Colegio et al. 2002.
Abbreviations: EL1, first extracellular loop; EL2, second extracellular loop. Barrels represent transmembrane helices.

Table 10: Summary of CLDN6 target characteristics

Category	Characteristics of CLDN6
**Molecular and functional characteristics of the target**	• Member of the claudin family of tight junction proteins.<br>• Plasma membrane protein with four membrane-spanning domains and two extracellular loops (23 kDa).
**Target expression in normal human tissue**	• CLDN6 mRNA expression is absent from the vast majority of normal tissue samples as assessed by qRT-PCR. Only placenta and umbilical cord as well as single samples out of several measured cerebellum, testes and lung samples expressed transcript levels slightly above the defined cut-off (Section 2.1.1).<br>• Absence of protein expression confirmed in a comprehensive panel of normal human tissue (EMA/FDA tissue cross-reactivity panel) by IHC (Table 11).

Category	Characteristics of CLDN6
	• Protein expression restricted to embryonic stem cells and embryonal tissues (epithelial structures of fetal lung, gastrointestinal tract, liver, kidney, heart and brain) (Anderson et al. 2008; Sullivan et al. 2012; Hewitt et al. 2006).<br>• CLDN6 EST profiling demonstrates gene predictions in embryonic tissues (Morita et al. 1999).
**Target expression in relevant cancers**	• Cancer-cell-specific CLDN6 expression is demonstrated for a wide range of cancers including ovarian, testicular, uterine, gastric, and lung cancers by qRT-PCR and IHC (Ushiku et al. 2012; Sullivan et al. 2012; Micke et al. 2014; Rendón-Huerta et al. 2010; Birks et al. 2010).<br>• Detection of CLDN6 expression in both primary and metastatic lesions (Section 2.1.2).
**Tumor promoting function**	• In ovarian cancer, tumors which are more advanced and cancer specimens with higher grade of dedifferentiation express CLDN6 more frequently (Section 2.2.1.2).<br>• Frequency of CLDN6 expression is higher in metastatic lesions.

Abbreviations: CLDN6, claudin 6; IHC, Immunohistochemistry; EMA, European Medicines Agency; EST, expressed sequence tag; FDA, Food and Drug administration; kDa, kilodaltons; mRNA, messenger ribonucleic acid; qRT-PCR, quantitative real-time polymerase chain reaction.

2.1.1 CLDN6 expression in healthy tissues

In a comprehensive set of normal tissue samples CLDN6 mRNA expression was evaluated by two systems, conventional real-time polymerase chain reaction (RT-PCR), and a microfluidic chip-based RT-PCR.

In the chip-based PCR assay system, CLDN6 expression in 48 different normal adult tissue samples derived from up to 17 different donors was investigated. CLDN6 mRNA expression was considered positive when the ΔΔCt value was greater than 30,000. Low expression (not necessarily resulting in detectable protein expression) was considered in the range between 5,000 and 30,000. The analysis confirmed the absence of CLDN6 in all normal tissue samples. Only 1/6 cerebellum, 1/3 lung, 1/6 placenta, 1/4 testes and 1/1 umbilical cord samples showed levels slightly above the defined cut-off.

Conventional RT-PCR was performed in 28 different normal adult human tissues derived from one to three different donors per tissue. The expression of CLDN6 was considered positive when the relative expression value was above or equal to 1,000 compared to CLDN6-negative expressing tissues. In none (including lung tissues) of the tissue samples, with the exception of 2/3 placenta samples, was CLDN6 mRNA detected.

To assess CLDN6 protein expression, normal human adult tissues (FDA/EMA recommended panel) were investigated by IHC using a commercially available polyclonal rabbit antibody. CLDN6 was not detected in any of the normal tissue samples. The complete analysis is summarized in Table 11.

Table 11: Summary of CLDN6 mRNA and protein expression in normal human adult tissues

Tissue	qRT-PCR (conventional)	qRT-PCR (chip-based)	IHC
Appendix	-	-	0/1
Adrenal	0/2	0/3	0/3
Bile duct	-	0/14	-
Blood cells	0/3	0/5	-
Blood vessels	-	0/3 [1]	0/4
Bone marrow	-	0/1	0/1
Brain	0/2	0/19 [2]	- [5]
Breast	0/3	0/5	0/6
Cerebellum	-	**1/6 (weak)**	0/3
Cerebral cortex	-	0/1	0/6
Colon	0/3	0/1	0/6
Duodenum	0/3	0/2	0/2 [6]
Epididymis	-	0/1	-
Esophagus	0/3	0/1	0/3
Eye	-	0/1	0/2
Fallopian tube	0/2	0/2	0/4
Gall bladder	0/2	0/1	0/3[6]
Heart	-	0/1	0/5
Inner ear	-	-	0/3
Intestine, small	0/2	0/3 [3]	0/3
Kidney	0/3	0/2	0/22
Liver	0/3	0/2	0/3
Lung	0/3	**1/3 (weak)**	0/4
Lymph node	0/3	0/2	0/2 [6]
Nerve	-	0/2 [4]	0/11
Ovary	0/3	0/2	0/22
Pancreas	0/3	0/4	0/8
Parathyroid	-	-	0/1
Parotid gland	-	0/2	0/1
Pituitary	-	0/2	0/2
Placenta	**2/3 (weak)**	**1/6 (weak)**	0/6
Prostate	0/3	0/1	0/4
Rectum	0/2	0/2	0/3
Salivary gland	-	0/1	-
Skin	0/3	0/1	0/4
Spinal cord	-	0/1	0/2
Spleen	0/2	0/1	0/2
Stomach	0/3	0/4	0/12
Striated muscle	-	0/1	0/3
Testis	0/2	1/4	0/7
Thymus	0/2	0/2	0/4
Thyroid	0/3	0/4	0/8
Tonsil	-	0/3	0/3
Trachea	-	0/1	-
Umbilical cord	-	**1/1 (weak)**	-
Ureter	-	0/1	0/4
Urinary bladder	0/2	0/3	0/3
**Uterus**	-	0/1	-
Uterus, cervix	-	0/1	0/5
Uterus, endometrium	0/3	0/2	0/4

qRT-PCR was performed with -target-specific -intron-spanning- primers using a conventional real-time cycler or microfluidic chip-based system. CLDN6 expression was scored as positive when the relative expression value was ≥1000 (conventional qRT-PCR) or ≥5000 (chip-based qRT-PCR). IHC of CLDN6 expression in tissue sections was done using a polyclonal rabbit anti-CLDN6 antibody.

1) Blood vessel: aorta, artery and saphenous vein (one sample of each);
2) Brain: accumbens, cerebral meninges, corpus callosum, diencephalon, frontal lobe, hippocampus, medulla oblongata, occipital lobe, olfactory (region), parietal lobe, pineal gland, pons, putamen, substantia nigra, temporal lobe, thalamus (one sample of each), postcentral gyrus (two samples);
3) Ileum (two samples), jejunum (one sample);
4) Nerve: dorsal root ganglion and peripheral (one sample of each);
5) Brain (immunohistochemistry): see cerebellum cerebellar cortex;
6) Cryo tissues.

2.1.2 CLDN6 expression in cancer

Prevalence of CLDN6 in human cancers was assessed in tissue sections from different tumor types by conventional RT-PCR and microfluidic chip-based RT-PCR. Moreover, multiple cancer entities were analyzed for CLDN6 expression by IHC using paraffin-embedded tissue sections. A commercial polyclonal rabbit anti-CLDN6 antibody was used to detect CLDN6 expression. The complete analysis is summarized in Table 12.

A subset of these CLDN6-positive patients have intermediate/strong expression of CLDN6 defined as ≥ 2+ staining intensity in IHC analysis. Only these patients are eligible for this trial.

Table 12: Summary of CLDN6 mRNA and protein expression in human cancers

Cancer type	qRT-PCR (conventional)			qRT-PCR (chip-based)			IHC		
	Total	CLDN6 positive	%	Total	CLDN6 positive	%	Total	CLDN6 positive	%
Testicular cancer	19	17	90	26	24	92	104	97	93
Ovarian cancer	47	27	57	70	33	47	834	469	56
Uterine cancer	9	3	33	-	-	-	487	114	23
Lung cancer	54	16	30	171	11	6	728	79	11
Gastric cancer	59	4	7	82	15	18	70	6	9
Liver cancer	22	2	9	27	1	4	11	1	9
Urinary bladder cancer	1	0	0	-	-	-	13	1	8
Breast cancer	-	-	-	150	30	20	129	3	3
Cervical cancer	5	0	0	-	-	-	360	10	3
Colon cancer	10	0	0	35	3	9	36	1	3
Skin cancer	4	2	2	-	-	-	130	3	2
Pancreatic cancer	10	3	30	19	6	32	96	1	1
Duodenal cancer	-	-	-	-	-	-	4	0	0
Head & neck cancer	2	1	1	-	-	-	60	0	0
Kidney cancer	37	5	14	34	0	0	12	0	0
Melanoma	12	0	0	8	0	0	54	0	0
Parotid cancer	-	-	-	-	-	-	6	0	0
Prostate cancer	16	0	0	16	0	0	83	0	0
Sarcoma	19	2	11	-	-	-	89	0	0
Bile duct cancer	-	-	-	16	1	6	-	-	-
Blood cancer	-	-	-	3	1	33	-	-	-
Brain cancer	-	-	-	75	0	0	-	-	-

Cancer type	qRT-PCR (conventional)			qRT-PCR (chip-based)			IHC		
	Total	CLDN6 positive	%	Total	CLDN6 positive	%	Total	CLDN6 positive	%
Gallbladder cancer	-	-	-	3	0	0	-	-	-
Rectal cancer	-	-	-	6	0	0	-	-	-
Thyroid cancer	5	0	0	-	-	-	-	-	-

qRT-PCR was performed with target-specific intron-spanning primers using a conventional real-time cycler or microfluidic chip-based system. CLDN6 expression was scored as positive when the relative expression value was >1000 (conventional qRT-PCR) or >5000 (chip-based qRT-PCR). IHC of CLDN6 expression in tissue sections was performed using a polyclonal rabbit anti-CLDN6 antibody or a monoclonal mouse anti-CLDN6 antibody.

2.2 BACKGROUND

2.2.1 Overview of the diseases

2.2.1.1 Testicular cancer

In 2019, there were an estimated 9500 new cases of testicular cancer in the United States (US) and 410 deaths. Although testicular cancer is very rare (only 1% of all male tumors), the incidence has been steadily increasing from 5.7 per 100,000 in 1992 to 6.8 per 100,000 in 2009 (NCI PDQ 2019a). Testicular cancer most often develops in young and middle-aged men and is usually curable (Ries et al. 2007; Krege et al. 2008; Groll et al. 2007; Neill et al. 2007; Tandstad et al. 2009). Most (> 95%) testicular cancers are germ cell tumors (Smith et al. 2018). The non-germ cell tumors comprise the remaining 5% of testicular cancers, which are generally gonadal stromal tumors (Leydig cell, Sertoli cell, granulosa cell), and they tend to be benign (Smith et al. 2018; Oldenburg et al. 2013).

Germ cell tumors are broadly divided into seminomas and non-seminomas based on different prognosis and treatment algorithms.

The seminomatous types are more sensitive to radio- and chemotherapy and less prone to metastasize. Moreover, non-seminomas may include teratomatous elements, which tend to be resistant to chemotherapy and often require surgery for cure. By definition, pure seminomas do not contain elements of teratoma. Therefore, surgery plays a larger role in the management of non-seminomas than in the management of seminomas (Smith et al. 2018).

Above 93% of testicular cancers across all subtypes express CLDN6, mostly homogenously across all tumor cells and at high level (Table 12, IB Non-clinical Section 4).

2.2.1.1.1 Treatment of testicular seminomas

Patients with clinical Stage I seminomas are typically offered three management options: surveillance, radiotherapy, or adjuvant chemotherapy. Surveillance avoids unnecessary treatment, but has a relapse rate of up to 20%, most commonly in the retroperitoneum (Chovanec et al. 2016). Patients who relapse typically require full induction chemotherapy

with bleomycin, etoposide, and cisplatin or radiotherapy (Chovanec et al. 2016). The relapse rate following adjuvant radiotherapy is around 3% to 5% (Jones et al. 2005; Kollmannsberger et al. 2015). The third option is adjuvant chemotherapy with one cycle of carboplatin, for a relapse rate of approximately 4% to 5% (Oliver et al. 2011).

Patients with Stage II persistently elevated post orchiectomy tumor markers or disease in the retroperitoneum are offered either radiotherapy to the retroperitoneum (plus/minus ipsilateral iliac lymph nodes) or chemotherapy. Full induction chemotherapy (four cycles of etoposide and cisplatin [EP], or three cycles of bleomycin, etoposide, and cisplatin [BEP]) are preferred options for patients with Stage IIB or elevated serum markers. This approach has a cure rate of greater than 95% (Chovanec et al. 2016).

Patients with more extensive metastatic disease receive induction chemotherapy: three cycles of BEP or four cycles of EP for good-risk disease and four cycles of BEP for intermediate-risk disease. Five-year survival is approximately 90% and 80% for good- and intermediate-risk patients, respectively (Albers et al. 2015; Rajpert-De Meyts et al. 2016).

Even though cure rates are very high, there is a need to develop effective treatments for patients with relapsed disease. As described above, CLDN6 is expected to be expressed in above 90% of patients at a sufficiently high level to be eligible for this trial.

2.2.1.1.2 Treatment of non-seminomatous testicular cancer

Patients with Stage I non-seminomatous germ cell tumors have three management options: surveillance, adjuvant chemotherapy, and retroperitoneal lymph node dissection. All three options have a 5-year survival of approximately 99% (van Dijk et al. 2006). The overall relapse rate for primary retroperitoneal lymph node dissection is around 10%. Almost all relapses are salvaged with chemotherapy (Beck et al. 2005). Chemotherapy typically includes one or two cycles of BEP. Chemotherapy risk of relapse is around 3%, with relapses typically occurring in the retroperitoneum (Chovanec et al. 2016).

Patients with limited retroperitoneal disease but no metastasis outside of the retroperitoneum receive retroperitoneal lymph node dissection or chemotherapy (three cycles of BEP or four cycles of EP). The National Comprehensive Cancer Network (NCCN) guidelines recommend chemotherapy for Stage IIB patients because of a higher risk of micro-metastases (NCCN 2019a). Survival remains excellent in this patient population (> 95%) with either choice (van Dijk et al. 2006).

Patients with high-volume retroperitoneal disease, distant metastases, and/or highly elevated tumor markers get induction chemotherapy. Good-risk patients receive three cycles of BEP or four cycles of EP, whereas intermediate- or poor-risk patients receive four cycles of BEP. The 5 year OS is approximately 95%, 80%, and 50% for good, intermediate, and poor risk, respectively (van Dijk et al. 2006; Sheinfeld et al. 2003).

Patients with a persistent or recurrent retroperitoneal mass greater than 1 cm following chemotherapy and negative tumor markers undergo a post-chemotherapy retroperitoneal lymph node dissection (Daneshmand et al. 2012). If tumor markers remain elevated, patients receive salvage chemotherapy.

Salvage chemotherapy typically consists of four cycles of etoposide, ifosfamide, and cisplatin; vinblastine, ifosfamide, and cisplatin; paclitaxel, ifosfamide, and cisplatin; or paclitaxel, ifosfamide, carboplatin, and etoposide. Around 50% achieve a complete radiographic or tumor marker response; however, half will relapse again (Feldman et al. 2008).

Patients with persistent elevations in tumor markers and growing tumor mass who have exhausted all chemotherapy options, typically have very aggressive tumors, often with distant metastases. Long-term survival has been reported in only one to two-thirds of these patients (Beck et al. 2005). As such, this group is in need of novel treatment options.

2.2.1.2 Ovarian cancer

The majority of ovarian cancers (> 95%) are of epithelial derivation (Chan et al. 2006; Prat 2012; Jelovac and Armstrong. 2011). However, the less common of these histologies, such as carcinosarcomas, clear cell tumors, mucinous carcinomas, low-grade serous/endometrioid epithelial carcinomas and borderline epithelial tumors, which together comprise around 25% of all cases, can be considered to be treated similarly to the epithelial subtypes in general and for the purposes of this document should be considered to have a similar treatment algorithm to that covered in Section 2.2.1.2.1 for recurrent or metastatic disease. The non-epithelial subtypes comprise the sex cord tumors and the germ cell tumors, and together make up the remaining 5% of cases (Prat 2012).

In 2019, 22,530 women were expected to be diagnosed with ovarian cancer, of whom 13,980 were expected to die of their disease (NCI PDQ 2019b). Most patients have widespread disease at diagnosis and the majority will die of ovarian cancer. The 5-year survival for Stage 4 disease is 28% (ACS 2019a).

About 56% of analyzed ovarian cancers across all subtypes were shown to express CLDN6 (Table 12, IB Section 4). In 67% of serious carcinomas, the most prevalent subtype of epithelial ovarian cancer, CLDN6 expression was positive. A high prevalence of CLDN6 positivity could also be observed in undifferentiated/poorly differentiated epithelial ovarian cancers (75%) and germ cell tumors (75-100%). Notably, the frequency of CLDN6+ cases increased with the progression of the disease and directly correlated with both the International Federation of Gynecology and Obstetrics (FIGO) stage and the grade of ovarian tumors. Furthermore, compared to primary ovarian cancer, the frequency of CLDN6-positive samples was significantly increased in metastasis lesions.

2.2.1.2.1 Treatment of epithelial ovarian cancer

After surgical staging and maximal de-bulking of the tumor, standard treatments range from observation only for very early stage, low-grade disease to taxane/carboplatin chemotherapy, with high initial response rates, for more advanced stages (NCCN 2019b). The tumor usually recurs, and choice of treatment is based on the platinum-free interval: either re-challenge with platinum-based chemotherapy in case of platinum-sensitive disease, or single-agent treatment such as topotecan, doxorubicin or paclitaxel if the disease is considered platinum-resistant or refractory. Poly adenosine diphosphate ribose polymerase (PARP) inhibitors and bevacizumab are also approved as maintenance therapies after chemotherapy induction, but should not be used simultaneously (Avastin® USPI 2019; Lynparza® USPI 2019; Rubraca® USPI 2019). Most patients fail many lines of therapy and ultimately succumb to their disease (Ledermann et al. 2013).

Early phase clinical trials of immune checkpoint inhibitors/blockers, such as anti-cytotoxic T lymphocyte associated protein 4 (anti-CTLA-4), anti-programmed cell death protein 1 (anti PD-1) antibody and/or anti-programmed death ligand 1 (anti-PD-L1) antibodies, have shown minimal anti-tumor activity in patients with ovarian cancer, with ORRs from 11% to 15% (Mittica et al. 2016).

Despite the approval of new therapeutic agents and improved treatment strategies, there continues to be a substantial unmet need for patients with advanced ovarian cancer. There are limited treatment options for women who have failed two or more lines of chemotherapy and have received or were ineligible to receive bevacizumab or a PARP inhibitor.

2.2.1.2.2 Sex cord tumors

Sex cord ovarian tumors can be divided into granulosa and Sertoli-Leydig cell tumors (NCCN 2019b), which generally occur in adolescents and younger women. Both are slowgrowing- with relatively low metastatic rates (< 30%; Bryk et al. 2016; Dridi et al. 2018; Durmuş et al. 2019). Initial management includes surgery, chemo- and radiotherapy. Chemotherapy tends to be platinum-based, with a weak evidence base to support any specific regimen or approach (NCCN 2019b).

Upon recurrence, due to the lack of randomized controlled trial data, it is considered acceptable to offer patients participation in a clinical trial (NCCN 2019b). Otherwise, taxane/platinum-based therapies, etoposide and ifosfamide are frequently used, as for epithelial tumors. For tumors expressing steroid receptors, common hormone therapies include aromatase inhibitors, leuprolide and tamoxifen (Teoh et al. 2010). Bevacizumab is also used in granulosa cell tumors (Tao et al. 2009). Treatment of recurrent and metastatic sex cord tumors reveals no systemic standard therapy and a high medical need for novel approaches.

2.2.1.2.3 Germ cell tumors

Malignant germ cell tumors of the ovary are diagnosed at an early stage in young girls and women, with a median age of 16 to 20 years, and the vast majority are cured, even the late-stage tumors (Mangili et al. 2011). There is no standard systemic treatment approach to metastasis, with strategies borrowed from clinical experience with testicular cancer (NCCN 2019b; Gershenson 2007). For patients with persistent, refractory, or platinum-resistant (relapse within 6 weeks of completing platinum-based chemotherapy) recurrent tumors, the prognosis is much worse, and high-dose chemotherapy with stem cell rescue may be used, although randomized data suggest that this approach is no better than conventional chemotherapy (Motzer et al. 2007). Platinum refractory and resistant disease, although rare, thus remains an area of high unmet medical need and novel therapies are needed.

2.2.1.3 Endometrial cancer

Endometrial carcinomas include the common and readily treatable well-differentiated endometrioid adenocarcinoma (85%), as well as the more aggressive non-endometrioid endometrial adenocarcinomas (15%): Papillary serous carcinoma (10%) followed by clear cell (2-4%), mucinous (0.6-5%), and squamous cell (0.1-0.5%) (Bokhman 1983; Mendivil et al. 2009). Malignant mixed Müllerian tumors are rare endometrial tumors which show both glandular (carcinomatous) and stromal (sarcomatous) differentiation.

About 23% of analyzed uterine cancers across most subtypes were shown to express CLDN6 (Table 12, IB Section 4).

2.2.1.3.1 Treatment of endometrial cancer

Endometrial cancer is one of the most common cancers of the female genital tract, with 382,069 new cases diagnosed, and 89,929 deaths worldwide in 2018 (Globocan 2019a). In the US, estimated new cases and deaths for 2019 were 61,880 and 12,160 respectively (ACS 2019b). Localized endometrial cancer may be treated by hysterectomy and bilateral salpingo-oophorectomy, leading to good long-term survival outcomes (Ott et al. 2017). Other options include radiation therapy and brachytherapy. Adjuvant systemic therapy with carboplatin paclitaxel is used in patients with high-grade histology and hormone therapy in those with lower grade disease (NCCN 2019c).

Once disease spreads, the prognosis is poor, with a 5-year survival rate of less than 50% for patients with lymph node metastases and less than 20% for patients with peritoneal or distant metastases (Dowdy 2014). Pembrolizumab may be prescribed for microsatellite instability high/deficient mismatch repair (MSI; high/dMMR) tumors after progression on chemotherapy, although the response rate in a small Phase 2 study of PD-(L)1 positive tumors was only 13% (NCCN 2019c; Ott et al. 2017). No curative treatments are currently available for recurrent or metastatic endometrial cancer. Since no agent has a proven impact on survival, advanced and metastatic disease is an area of high unmet medical need, and novel treatment approaches are needed.

2.2.1.4 Non-small cell lung cancer

In the US, it was estimated that approximately 228,000 patients would be diagnosed with lung cancer and that 80 to 85% of these cases would be one of the NSCLC histologies (ACS 2019d). The diagnosis of NSCLC is most often made when the disease is advanced or metastatic (Stage IIIB/IV), beyond the time when it can be controlled with localized therapies, leaving only systemic treatment options. In 2019, lung cancer was estimated to account for approximately 24% of all cancer-related deaths in the US.

About 11% of all analyzed lung cancers were CLDN6 positive, all belonging to the NSCLC subtype (Table 12). Notably, 18% of the adenocarcinomas, the most common subtype of lung cancer, were CLDN6 positive. About 10% of large cell carcinoma samples were confirmed to express CLDN6.

2.2.1.4.1 Treatment of metastatic non-small cell lung cancer

The approved anti-PD-(L)1 inhibitors nivolumab, pembrolizumab, durvalumab and atezolizumab have demonstrated substantial anti-tumor activity, and are changing the treatment paradigm in driver mutation-negative NSCLC. Anti-PD-(L)1 therapies are now approved as monotherapy or combination therapy with chemotherapy in first-line, and as monotherapy in second-line (Opdivo® USPI 2019; Keytruda® USPI 2019; Tecentriq® USPI 2019; Imfinzi® USPI 2019).

Anti-PD-(L)1 therapy given after progression following platinum-based chemotherapy demonstrates an ORR of approximately 15%, regardless of PD-L1 status in driver mutation-negative NSCLC. The ORR in patients with a Tumor Proportion Score of > 50% was approximately 30%. Of note, the ORR of the standard comparator, docetaxel, was approximately 9% in this trial (Herbst et al. 2016). Now that anti-PD-(L)1 therapy is approved as first-line therapy in driver mutation-negative NSCLC, second-line therapy is in flux as standard chemotherapy regimens have not been rigorously evaluated after anti-PD-(L)1 failures, and there is no standard therapy for patients who have progressed on or after checkpoint inhibitors.

Epidermal growth factor (EGFR) mutations play a significant role in driving tumor development and progression in approximately 20% of NSCLC adenocarcinomas (mutant NSCLC [mNSCLC]). Unlike driver mutation-negative NSCLC, mNSCLC does not respond well to checkpoint inhibition after anti-EGFR therapy, for reasons that remain unclear. One theory for lack of anti-PD-(L)1 efficacy is that mNSCLC does not carry as high a mutational load as driver mutation-negative NSCLC, making these tumors less amenable to native adaptive immune attack by T cells (Miura et al. 2018).

Despite the significant advances in NSCLC treatment with anti-PD-(L)1 therapy, advanced disease remains largely incurable. NSCLC is a very good candidate for evaluation of novel approaches, with a demonstrated susceptibility to immune modulation with monotherapy checkpoint inhibitors.

2.2.1.5 Gastric cancer

Gastric cancer represents the fifth most common malignancy and, despite a steady decline, it is still the third-leading cause of cancer mortality worldwide. The highest incidence (> 20 per 100,000 in men) is seen in China, Japan, Latin America, and Eastern Europe, whereas the lowest incidence (< 10 per 100,000 in men) is seen in North America, parts of Africa and Northern Europe (Globocan 2019b). Only 28% of newly diagnosed gastric cancers are localized with a 5-year OS rate of 31.5%, which remains stable over the last 30 to 40 years (SEER 2019). Surgery is still the only chance for cure and a multimodality treatment approach is commonly utilized. Advanced disease carries a dismal prognosis and treatment remains challenging, with a 5-year OS rate around 5%. Thus, despite decreasing incidence, gastric cancer remains a serious health burden globally with high mortality rate (Charalampakis et al. 2018).

About 9% of analyzed gastric cancers were CLDN6 positive (Table 12).

2.2.1.5.1 Treatment of gastric cancer

Although treatment algorithms differ worldwide, with more focus on the use of radiotherapy in North America vs chemotherapy in Europe and Asia, there is general consensus on the use of (neo)adjuvant treatment with initial surgery. The chemotherapy regimens vary, but most are based on fluoropyrimidines, platinums, and anthracyclines or taxanes (Charalampakis et al. 2018).

Once disease has spread, the 5-year survival is near-zero. However, several regimens have proven to impact OS in the first- and second-line settings (Van Cutsem et al. 2006; Cunningham et al. 2008; Koizumi et al. 2008; Ford et al. 2014; Thuss-Patience et al. 2011), although all patients' disease progresses and the impact of systemic therapy in second-line remains questionable. In addition, several targeted therapies have recently been shown to impact survival to some extent, although the increments are generally of the order of 1 to 2 months only (Bang et al. 2010; Fuchs et al. 2014; Wilke et al. 2014; Li et al. 2016; Kang et al. 2017).

Despite these recent advances, the response rate for PD-(L)1 inhibitors in the general metastatic gastric cancer population does not exceed 15% (Charalampakis et al. 2018), illustrating that the majority of patients do not benefit. Taken together with the very small increments shown in OS with targeted therapies and overall poor prognosis, these data suggest that there is an abiding high medical need for novel mechanisms and new agents in the treatment of advanced or metastatic gastric cancer.

2.2.1.6 Rare tumors

Rare cancers are those that affect fewer than 40,000 people per year in the US. As a group, these approximate 200 indications, making up a quarter of all cancers, and a quarter of all cancer deaths each year (NCI 2019). Many of these cancers are highly dedifferentiated and

cannot be clearly allocated to any histological group, and thus have a substantial likelihood of being CLDN6 positive. More than 4 million people in the EU are affected by rare cancers, and more than 50% of them can expect to die of their cancer within 5 years (Gatta et al. 2017).

2.2.1.6.1 Treatment of rare tumors

Although new treatments are continually under development for common cancers, finding and establishing new treatments for rare cancers is very difficult. Treatment for advanced or metastatic rare cancers usually involves chemotherapy in the first-line setting, and upon progression or recurrence, treatment choice is very limited due to a lack of data and a lack of clinical consensus (Gatta et al. 2017). The main challenges in the treatment of rare cancers include lack of standard treatment guidelines (66%), insufficient personal experience with rare cancer treatments (65%), and lack of evidence regarding treatments (54%), according to a recent survey of oncologists (Shin et al. 2015).

The American Society of Clinical Oncology recently published a report which cites rare cancers as an area of specific progress, with new therapies shown to improve outcomes in anaplastic thyroid cancer (dabrafenib/trametinib), desmoid tumors (sorafenib), midgut neuroendocrine tumors (177Lu-Dotatate), human epidermal growth factor receptor 2 (HER2)-positive uterine serous adenocarcinoma (trastuzumab) and tenosynovial giant cell tumors (pexidartinib) (Pal et al. 2019). The recent approvals of larotrectinib in tumors with neurotrophic tyrosine kinase fusion and checkpoint inhibitors in tumors with MMR defects or MSI unstable tumors also address a number of rare cancers (Vitrakvi® USPI 2019; Luchini et al. 2019). However, these are small improvements in the context of nearly 200 indications with high unmet medical need. Therefore, rare cancers represent an area in dire need of new and innovative treatment options.

2.2.2 Introduction to the investigational treatment

2.2.2.1 CLDN6 CAR-T/CLDN6 CAR-T(A) (IMP-1 and IMP-1(A))

The investigational medicinal product 1 (IMP-1, manual process) and IMP-1(A)(automated process) are composed of autologous patient-derived $CD4^+$ and $CD8^+$ T cells expressing a CAR with specificity for the oncofetal antigen CLDN6. The CAR-T doses planned to be tested with the manual process are 10^7, 10^8, and 10^9 CAR-T. The CAR-T doses planned to be tested with the automated process are 10^6, 10^7, 10^8, and 2-5x10^8 CAR-T(A).

CLDN6 CAR-T/CLDN6 CAR-T(A) are autologous T cells enriched from peripheral blood mononuclear cells and transduced with a gamma-retroviral self-inactivating vector expressing a second generation CAR composed of an extracellular single chain variable fragment (scFv) with specificity for CLDN6, linked via a CD8 hinge domain to intracellular 4-1BB and CD3ζ signaling domains (Figure 8). The scFv fragment is derived from the monoclonal antibody IMAB206-C46S. This antibody recognizes a conformational region in the first and second

extracellular loop of CLDN6, and displays both high binding affinity and high antigen specificity.

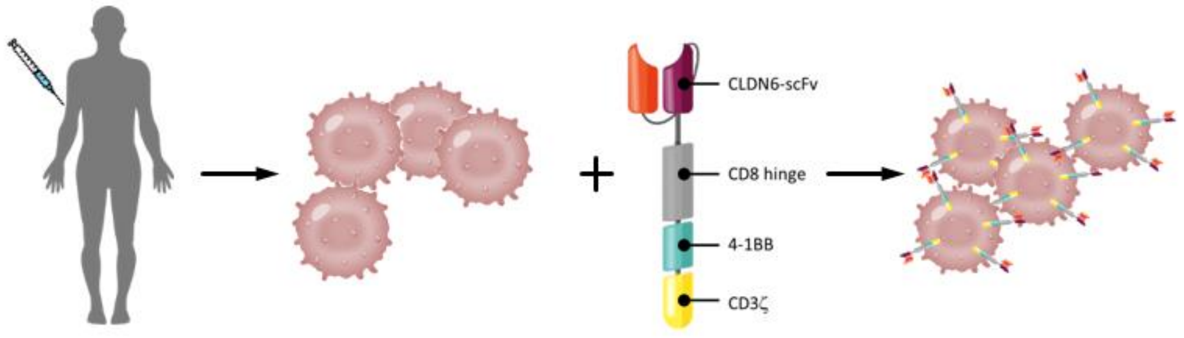

Figure 8: Schematic representation of the IMP-1 and IMP-1(A) autologous CLDN6 CAR-T/CLDN6 CAR-T(A)

For generation of IMP-1 and IMP-1(A), autologous T cells are engineered to express the CLDN6 CAR/CLDN6 CAR-T(A). A schematic representation of the second generation CLDN6 CAR is shown.

CLDN6 CAR-T/CLDN6 CAR-T(A) are cryopreserved in infusible cryomedia and will be administered as a single intravenous (i.v.) infusion. Each bag will contain an aliquot (volume dependent upon dose) of CLDN6 CAR-T/CLDN6 CAR-T(A) in cryomedia containing the following infusible grade reagents (% v/v): 50% CryoStor CS10, 50% sodium chloride (0.9%). The total amount of dimethyl sulfoxide is 5%.

Please refer to the IB for detailed information regarding the available preclinical data on pharmacology and toxicology of CLDN6 CAR-T/CLDN6 CAR-T(A). Further guidance on administration can be found in the Investigator Manual.

2.2.2.2 CLDN6 RNA-LPX (IMP-2)

The CLDN6 RNA-LPX (IMP-2), an i.v. liposome-formulated mRNA vaccine encoding the CAR target antigen CLDN6 will be used to stimulate and expand adoptively transferred CLDN6 CAR-T/CLDN6 CAR-T(A) *in vivo*. An intra-patient dose escalation schedule will be used starting with 25 µg for the first vaccination after CAR-T infusion, and increasing to 50 µg for the next vaccination. If 50 µg is tolerated, then this tolerated dose (TD) will be administered every 3 weeks. For further guidance on dose modifications, see Section 6.6.

For preparation of the i.v. injectable RNA-LPX product, highly purified single-stranded, 5'-capped mRNA is formulated with liposomes composed of the synthetic cationic lipid DOTMA (R-1,2-di-O-oleoyl-3-trimethylammonium propane [chloride salt]) and the phospholipid DOPE (1,2-Dioleoyl-sn-glycero-3-phosphoethanolamine).

A schematic illustration of the general structure of the RNA, liposomes and lipoplexes is given in Figure 9.

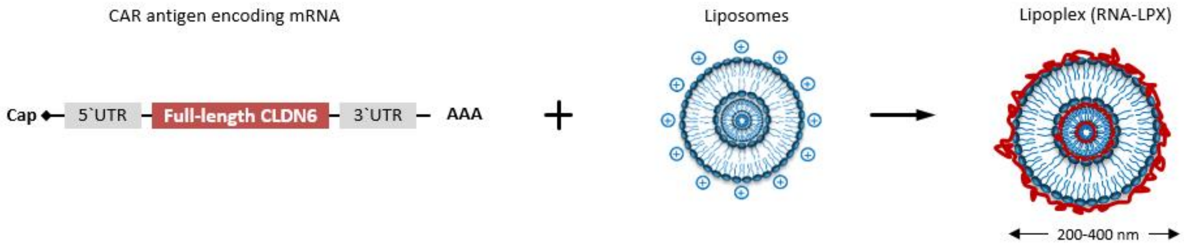

Figure 9: Schematic representation of the IMP-2, CLDN6-encoding liposome-formulated RNA

Full-length CLDN6-encoding mRNA is complexed with cationic liposomes to form lipoplexes IMP-2, CLDN6 RNA-LPX. A schematic illustration of the structure of the RNA with 5'-cap, 5'- and 3'-untranslated regions (UTRs), coding sequences and A30L70 poly(A)-tail, the liposomes and the resulting lipoplexes is shown.

Please refer to the IB for detailed information concerning the available pharmacology, toxicology, and AE profile of CLDN6 RNA-LPX. Further guidance on administration can be found in Section 6.1.3, the Investigator Manual, and in the Pharmacy Manual.

2.3 TRIAL RATIONALE

2.3.1 Rationale for the trial

Outcome remains poor for patients with relapsed or refractory advanced solid tumors. Treatment options include further targeted therapies, immunotherapies, radiotherapy or palliative chemotherapy, which might be less tolerated after previous repeated exposure to cytotoxic compounds, or best supportive care. Therapy in this population is not curative with an expected OS of a few months. The oncofetal antigen CLDN6 has emerged as an attractive therapeutic target because of its absence from toxicity relevant, adult, healthy tissues, and high expression in cancers with high unmet medical need. Thus, CLDN6 is a suitable target antigen for the development of a CAR-T against these cancers. The combination of CLDN6 CAR-T/CLDN6 CAR-T(A) with CLDN6 encoding RNA-LPX mediates controlled *in vivo* expansion, improved CAR-T persistence and anti-tumor efficacy in preclinical models, and constitutes an innovative concept for the specific and effective treatment of CLDN6-expressing tumors.

The main purpose of the trial is to determine a safe and potentially efficacious dose of CLDN6 CAR-T/CLDN6 CAR-T(A) +/- CLDN6 RNA-LPX. In a Phase I dose escalation with CLDN6 CAR-T/CLDN6 CAR-T(A) (Part 1) and CLDN6 CAR-T/CLDN6 CAR-T(A) with CLDN6 RNA-LPX (Part 2) according to the classical 3+3 design, the RP2D of the CLDN6 CAR-T/CLDN6 CAR-T(A) +/- CLDN6 RNA-LPX will be determined. The RP2D will be further explored in Phase 2a (Part 3) for efficacy signal seeking. So far, no drug targeting CLDN6 in cancer patients has been approved.

2.3.2 Rationale for administration of CLDN6 CAR-T/CLDN6 CAR-T(A) and CLDN6 RNA-LPX

CLDN6 CAR-T/CLDN6 CAR-T(A) is a CLDN6-targeting second generation CAR-T (Figure 10, 1A) that, in preclinical models, exerts highly specific and sensitive recognition and killing of CLDN6-positive tumor cells, translating into complete eradication of advanced human CLDN6-expressing tumors in ovarian xenograft mouse models. To further improve the CLDN6 CAR-T/CLDN6 CAR-T(A) activity, a novel approach was developed for stimulation of CLDN6 CAR-T/CLDN6 CAR-T(A) *in vivo* by presentation of the CAR target antigen on professional APCs resident in secondary lymphoid compartments. To this end, a liposomally formulated CLDN6 encoding mRNA (CLDN6 RNA-LPX) is used (Figure 10, 1B) to systemically deliver the CAR target antigen to APCs such as dendritic cells in secondary lymphoid tissues (Figure 10, 1C). By repetitive RNA-LPX treatment, CAR-T can be stimulated and expanded *in vivo* in a controlled manner resulting in persistence (Figure 10, 1D). Stimulation of CLDN6 CAR-T/CLDN6 CAR-T(A) by CLDN6 RNA-LPX treatment accelerates anti-tumor activity even at low CAR-T doses. The trial design aims at assessing the capacity of this concept to safely, efficaciously, and specifically target CLDN6 expressing tumor cells, strengthened by improved persistence and function of CLDN6 CAR-T/CLDN6 CAR-T(A).

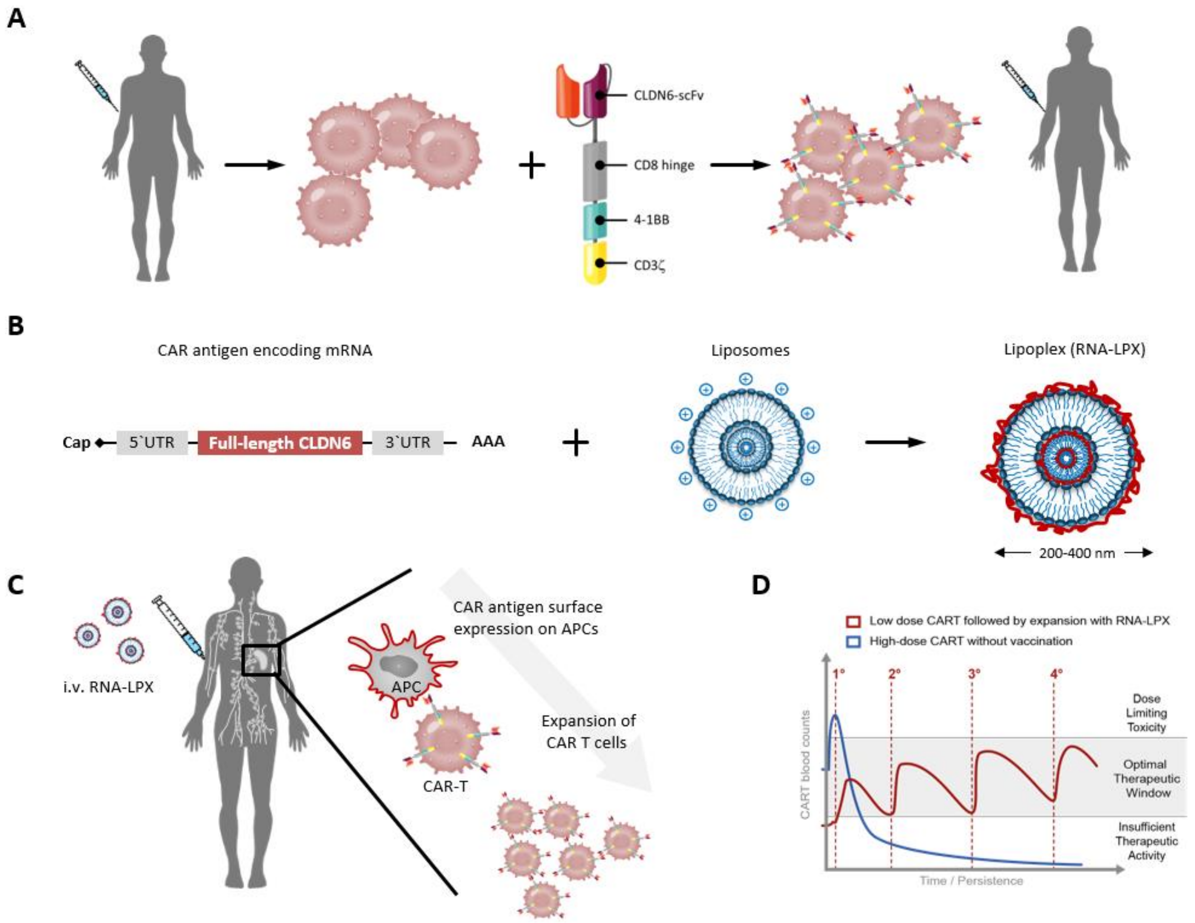

Figure 10: Concept of the Sponsor's CLDN6 CAR-T/CLDN6 CAR-T(A) therapy with CLDN6 RNA-LPX-mediated *in vivo* expansion of CLDN6 CAR-T/CLDN6 CAR-T(A)

(A) Autologous T cells are engineered to express the CLDN6-CAR and are reinfused into the (lymphodepleted) patient. (B) Full-length CLDN6-encoding mRNA is complexed with cationic liposomes to form lipoplexes (CLDN6 RNA-LPX). (C) RNA-LPX selectively targets antigen-presenting- cells in secondary lymphoid organs. The RNA sequence contains naturally occurring sequence elements at 3´- and 5´-untranslated regions (UTRs) that significantly increase the intracellular half-life and the translational efficiency of the molecule. Engagement of CAR-T with the CAR target results in CAR-T activation and expansion. (D) CAR target-encoding RNA-LPX can be repeatedly administered to prolong the life-time of CAR-T.

2.3.3 Rationale for lymphodepleting chemotherapy choice and dose

In order to increase the potency of the transferred CAR-T, patients are conditioned with lymphodepleting (LD) chemotherapy regimens prior to adoptive cellular therapy (ACT) (Majzner et al. 2019). Patients (except those in the optional Part 2 without LD chemotherapy) will receive three consecutive days of LD chemotherapy, which includes i.v. administration of fludarabine 30 mg/m^2 and cyclophosphamide 500 mg/m^2 daily starting on d-5. The rationale for combining gene modified T cell therapy with LD is that clinical experience has shown that LD improves functional engraftment of adoptively transferred T cells in general, with various suggested mechanisms involved. The use of the non-myeloablative LD regimen containing fludarabine and cyclophosphamide improved overall response rates in patients after ACT of autologous tumor infiltrating lymphocytes in melanoma patients and this regimen was

adopted in the majority of subsequent engineered ACT studies (Dudley et al. 2002). Improved response rates to ACT therapy in melanoma patients could be correlated with enhanced lymphodepleting regimens (Dudley et al. 2008). Enhanced anti-tumor efficacy of CAR-T after LD was demonstrated in mouse models (Cheadle et al. 2009; Kochenderfer et al. 2010). Furthermore, the most effective treatment protocols using CD19-specific CAR-T for treatment of B cell lymphomas have employed pre-infusion chemotherapy (Zhang et al. 2015). The mechanism responsible for improved persistence of adoptively transferred T cells after LD comprise: (i) the elimination of cellular cytokine 'sinks' for homeostatic γc-cytokines such as IL-7 and IL-15, which activate and expand tumor-reactive T cells, (ii) the impairment of $CD4^{+}CD25^{+}$ T regulatory cells and myeloid derived suppressor cells that suppress tumor-reactive T cells, and (iii) the induction of tumor cell apoptosis and necrosis in conjunction with APC activation (Klebanoff et al. 2005). Lymphodepletion has, however, several disadvantages including the side effects and risks associated with respective chemotherapeutics as well as the risk for infections and sepsis (Brentjens et al. 2010; Robbins et al. 2015).

Fludarabine, a nucleoside analog, has been associated with neurological toxicity. Somnolence and peripheral neuropathy during and immediately following fludarabine infusion were frequently observed but usually reversible. The late-onset neurological symptoms that manifested in a pattern of progressive visual disturbances, ataxia, paresis, sometimes leading to coma and death, were of great concern and are usually associated with high doses and in patients with advanced disease or elderly patients, especially in patients with renal insufficiency (Cheson et al. 1994). Fludarabine also carries a risk of prolonged neutropenia and bone marrow failure, a more prominent risk in heavily pretreated patients (Mackall et al. 2017), and has been reported to induce autoimmune phenomena such as hemolytic anemia and/or autoimmune thrombocytopenia. Patients should be closely monitored for cytopenias, infections and hemolysis.

Cyclophosphamide may reduce or eliminate $CD4^{+}$ $CD25^{+}$ regulatory T cells, which can suppress the function of tumor-targeted adoptively transferred T cells (Galon and Bruni 2019; Kershaw et al. 2004). Adding fludarabine to cyclophosphamide has been shown to increase the persistence of CAR-T in the treatment of adult patients suffering from acute B lymphoblastic leukemia. Fludarabine is included in most of the lymphodepleting regimes today (Turtle et al. 2016a), but dose reductions should be considered for patients suffering from renal impairment and/or history of radiation therapy to the brain. Refer to the current product label for guidance on management of toxicity associated with the administration of fludarabine and cyclophosphamide.

The LD dose was chosen based on the literature cited above, summaries of product characteristics of registered CAR-T products, discussions with physicians experienced in cell therapy as well as the doses used in several trials investigating different CAR-T treatments in solid tumors (NCT00019136, NCT01583686, NCT02349724).

While LD can be of critical importance for optimal engraftment and persistence of transferred CAR-T, there are also risks associated with this approach specifically when used in solid tumors. Selection of an appropriate conditioning method appears to be crucial, as dose intensification increases regimen-associated toxicities. Drawing direct analogies with the regimens used for treating hematologic malignancies might not be optimal, as the goal of immune ablation prior to CAR-T therapy for solid tumors is different. In fact, the aim of complete myeloablation for hematologic disease is not only the creation of appropriate conditions for engraftment, but also the direct elimination of the malignant population, whereas in CAR-T for solid tumors the objective is to induce LD, opening a CAR-T niche and perhaps an inflammatory state. Experience from auto-stem and allo-stem cell transplantation in the setting of hematologic malignancy or autoimmune disease clearly demonstrates that high-intensity conditioning can be associated with multiple toxicities, including prolonged neutropenia and the associated risk of infection, mucositis, graft failure, engraftment syndrome, and early and late effects associated with lymphodepleting chemotherapy such as secondary myelodysplastic syndrome, secondary malignancy, renal insufficiency, etc. (Sorror et al. 2004; Scott et al. 2006; Griffith et al. 2005).

Taking the above mentioned toxicities into account as well as the fact that patients may have been pretreated with myelotoxic agents, patients may be treated with a reduced-dose LD regimen or without LD, both options will be decided upon by the SRC per SRC charter.

2.4 BENEFIT/RISK ASSESSMENT

2.4.1 Risk assessment

Table 13: Risk assessment

Risk	Risk description	Risk mitigation
**Target-related risks**		
Off-tumor/on-target effect.	CLDN6 is expressed by embryonic stem cells and during fetal organogenesis. CLDN6 protein expression was absent from all analyzed adult healthy tissues (IB Section 4.1.1.1.1), but very weak mRNA signals have been detected in placenta, umbilical cord and in single samples out of several tested samples of cerebellum, testes and lung tissue. As of September 16th, 2021, no off-tumor/on-target effects have been confirmed in the BNT211-01 trial.	Pregnant women are excluded from trial participation. Birth control methods are described in Section 12.4. Pregnancies should be avoided even after active trial participation.<br>Other on-target, off-tumor toxicities are not expected, however cannot be excluded at this stage of clinical development. Patients will be observed closely for the first two weeks after administration of CLDN6 CAR-T/CLDN6 CAR-T(A).
**General CAR-T and LD-related potential risks**		
Myelosuppression.	Risk of myelosuppression and prolonged cytopenias related to conditioning chemotherapy (ELIANA study of Kymriah, Grade ≥ 3 cytopenias not resolved by d28 included neutropenia [40%] and	Patients in monitored setting with frequent assessment of complete blood cell counts (CBCs).<br><br>Patients will receive transfusion of blood products per institutional guidelines.

Risk	Risk description	Risk mitigation
	thrombocytopenia [27%] among 52 responding patients.<br>JULIET study of Kymriah, Grade ≥ 3 cytopenias not resolved by d28 included thrombocytopenia [40%] and neutropenia [25%] among 106 treated patients).<br>In BNT211-01, as of September 16th, 2021, pronounced cytopenia (especially thrombocytopenia) have occurred in patients with testicular cancer with a recent high-dose chemotherapy/autologous stem cell transplantation.	Patients may be entered into the LD-free cohort or receive a reduced dose of fludarabine/cyclophosphamide.<br>Patients with HDCT/ASCT in the medical history should have an autologous stem cell back-up if receiving LD chemotherapy.
Febrile reaction.	Transient fever and chills.<br>As of September 16th, 2021, no febrile reactions have been confirmed in the BNT211-01 trial.	An evaluation for infection will be initiated and patients will be managed appropriately with antibiotics, fluids, and other supportive care as medically indicated and per the discretion of the treating physician.<br>In the event the patient develops sepsis or bacteremia following CLDN6 CAR-T/CLDN6 CAR-T(A) infusion, appropriate cultures and medical management should be initiated.
Febrile neutropenia.	Risk of febrile neutropenia related to conditioning chemotherapy.<br>As of September 16th, 2021, no cases of febrile neutropenias have been confirmed in the BNT211-01 trial.	Patients will be monitored while an in-patient for signs and symptoms of infection.<br>Local institutional guidelines will be followed for evaluation of any suspected infection.
Cytokine release syndrome (CRS).	CRS has been observed in patients after treatment with CAR-T targeting B-cell lineage markers. Severity has ranged from mild to life-threatening (requiring ICU admission, intubation, dialysis, etc.) and fatal in some cases. Disease burden predictive of severe CRS.<br>Incidence of Grade 3 CRS in CD19 CAR-T studies has ranged from 49% (ELIANA Study in childhood ALL) to 13% (ZUMA-1 Study in adult DLBCL). Concurrent neurotoxicity and overlap with HLH/MAS has also been reported.<br>Symptoms typically occur 1 to14 d after CLDN6 CAR-T/CLDN6 CAR-T(A) infusion with median time of 2 to 3 d after CAR-T infusion.	Study conducted with investigators at healthcare facilities experienced with the administration of cellular therapies.<br>In-patient hospitalization/observation for initial infusion of CLDN6 CAR-T/CLDN6 CAR-T(A) with first patient in each dose escalation cohort staggered by 27 d.<br>Patients and caregiver counseled on CRS symptoms and to return to clinic if any symptoms occur.<br>Patients will monitor fever daily for the first 28 d.<br>CRS has been abrogated in many cases with anti-cytokine directed therapy including tocilizumab and steroids.<br>Protocol includes CRS management guidelines (Section 6.6.4.1) and sites will be required to have tocilizumab available on site prior to administration of CLDN6 CAR-T/CLDN6 CAR-T(A).

Risk	Risk description	Risk mitigation
	As of September 16th, 2021, CRS Grade 1-2 have been described in 1/3 patients receiving 10^7 CLDN6 CAR-T in Part 2 and 3/3 patients receiving 10^8 CAR-T in Part 1 in the BNT211-01 trial.	
Hemophagocytic lymphohistiocytosis (HLH) or macrophage activation syndrome (MAS).	HLH/MAS has been described in patients receiving ACT and may overlap with CRS. As of September 16th, 2021, no cases of HLH/MAS have been confirmed in the BNT211-01 trial.	Study conducted with investigators at centers experienced with the administration of cellular therapies and management of HLH.
Immune effector cell-associated neurotoxicity syndrome (ICANS).	Neurotoxicity has been described with CAR-T therapies targeting B-cell lineage markers. Incidence of Grade 3 neurotoxicity in CD19 CAR-T studies has ranged from 31% (ZUMA-1 Study in adult DLBCL) to 18% (JULIET study in adult DLBCL) Symptoms include word-finding difficulties, delirium, encephalopathy, aphasia, seizure, hallucinations, tremor/myoclonus and in rare cases, diffuse cerebral edema.<br>Cerebral edema has been a major toxicity in some studies of CAR-T (i.e., JCAR015 ROCKET trial, NCT02535364).<br>As of September 16th, 2021, no cases of ICANS have been confirmed in the BNT211-01 trial.	Study conducted with investigators at centers experienced with the administration of cellular therapies and management of neurotoxicity related to cellular therapy.<br>In-patient hospitalization/observation for initial infusion of CLDN6 CAR-T/CLDN6 CAR-T(A) with first patient in each dose escalation cohort staggered by 28 d includes neurotoxicity management guidelines (Modified CARTOX) that include anti-cytokine directed therapy including steroids and regular monitoring of mental status using a validated instrument (ICE modification of CARTOX-10).<br>After each escalation of dose, an SRC will meet to review any relevant safety event.
Tumor Lysis Syndrome (TLS).	Based upon an expert TLS consensus panel, the risk of TLS is dependent on the disease type and burden of disease. This risk is deemed to be low for patients with non-bulky solid tumors that are not highly sensitive to chemotherapy (highly sensitive solid tumors include neuroblastoma, germ cell cancer, small cell lung cancer; Cairo et al. 2010). As of September 16th, 2021, no cases of TLS have been confirmed in the BNT211-01 trial.	In-patient monitoring before and after chemotherapy and infusion of CLDN6 CAR-T/CLDN6 CAR-T(A) including blood tests for potassium and uric acid.<br>Patients with bulky tumors will receive hydration and allopurinol prophylactically per the discretion of the investigator.<br>TLS resulting in renal insufficiency, rapidly rising uric acid or evidence of organ dysfunction will be managed with i.v. fluids and rasburicase as needed and determined by the treating physician.
Clonality and insertional mutagenesis.	Risk that people who receive gene-edited cells may develop new tumors derived from their genetically modified cells. However, the risk is low as the gammaretroviral vector is replication deficient and does not possess any pathogenic properties. Further, the self-inactivating configuration leads to the loss of	Patients will be followed yearly for up to 15 years clinically to assess for clonal outgrowth and secondary malignancies.

Risk	Risk description	Risk mitigation
	promotor/enhancer functions of the vector.<br>As of September 16th, 2021, such cases have not been confirmed in the BNT211-01 trial.	
**Potential risks relating to trial procedures**		
Leukapheresis risks.	Hypocalcemia, vasovagal response, venous access/need for catheter, blood loss, discomfort at venipuncture site, and infection.	Patients will be monitored during and after leukapheresis.
Tumor biopsy risks.	Bleeding, infection and depending upon location (e.g., lung) could include pneumothorax.	Biopsy by trained physicians.<br>IR guided biopsies should be obtained when needed.
**Potential risks related to CLDN6 RNA-LPX**		
Transient cytokine increase	The class-intrinsic safety profile of RNA-LPX *per se* appears to be dominated by mild-to-moderate, transient and manageable flu-like drug-related AEs reported as arthralgia, body temperature increased, chills, dizziness, feeling cold, headache, heart rate increased, hot flush, hyperhidrosis, influenza like illness, myalgia, nausea, pyrexia, tachycardia, and vomiting. These AEs are anticipated and correlate with the laboratory changes observed in the clinical trials with RNA-LPX, namely the distinct range of cytokines released by RNA-LPX (IFN-α, IFN-γ, IP-10, IL-12, IL-6, and TNF-α) and transient lymphopenia mediated by type 1 IFN (by sequestration). The clinically manifested AEs are manageable by analgesics or antipyretics at recommended commonly used doses.	Regular safety monitoring of laboratory and clinical parameters in all clinical trials with RNA-LPX.<br>Regular safety data review (by sponsor and/or SRC) for identification and evaluation of potential safety concerns.<br>Treatment recommendation:<br>• Treat fever with acetaminophen or non-steroidal anti-inflammatory drugs (NSAIDs) with a dose per institution's recommendation.<br>• After the first occurrence of flu-like symptomatology, patients can be pre-medicated with standard therapeutic dose of acetaminophen, or NSAIDs, at least 60 min before RNA-LPX administration followed by a second dose 5-8 h after RNA-LPX.<br>• Corticosteroid should be avoided as either prophylaxis or treatment as it counteracts the effects of RNA-LPX.<br>Ensure adequate hydration of patients on the day of RNA-LPX administration. Consider administering i.v. isotonic fluid (e.g., balanced crystalloids 500 - 1000 mL) within approximately 2 hours following the dose of RNA-LPX per institutional standard.
Lipid accumulation in the liver and/or other organs.	Please refer to the Investigator's Brochure.	Regular safety monitoring of laboratory and clinical parameters in all clinical trials with RNA-LPX.<br>Regular safety data review (by sponsor and/or SRC) for identification and evaluation of potential safety concerns.
**General risks for parenteral products**		

Risk	Risk description	Risk mitigation
Infusion-related reactions and anaphylaxis	Signs and symptoms may include pruritus, urticaria, fever, rigors/chills, diaphoresis, bronchospasms, and cardiovascular collapse. Severe hypersensitivity reactions to parenterally administered anti-cancer treatments are quite rare, occurring < 5% of the time across all agents. Patients with history of previous drug-induced allergic or hypersensitivity reactions may be predisposed to such reactions.	Infusion-related reactions.<br>Carefully evaluate patients with previous drug-induced- allergic reactions that can lead to an increased risk of hypersensitivity reactions during infusion.<br>Educate patients on the possible infusion-related reactions that may occur during their course of therapy.<br>Mild-to-moderate infusion reactions may be managed antihistamines for symptom management, and/or adding corticosteroids. Drug administration must be stopped immediately if the patient experiences noticeable chest pain, cardiac issues, or anaphylaxis.<br>Appropriate supportive care should be initiated.<br>Anaphylaxis.<br>Patients should be treated at centers equipped to handle life-threatening reactions and institutional treatment protocol should be followed.<br>At the first sign of a reaction, the therapy should be stopped with prompt intervention by the medical and healthcare staff. Regular safety monitoring of laboratory and clinical parameters in all clinical trials with RNA-LPX.<br>Regular safety data review (by sponsor and/or SRC) for identification and evaluation of potential safety concerns.

Abbreviations: AE, Adverse event; ALL, acute lymphoblastic leukemia; CAR-T, Chimeric antigen receptor T cell; CARTOX; CAR-T-cell-therapy-associated TOXicity (working group); CLDN6, claudin 6; CRS, Cytokine release syndrome; d, day; DLBCL, Diffuse large B-cell lymphoma; HLH, Hemophagocytic lymphohistiocytosis; ICE, immune effector cell-associated encephalopathy; ICU, intensive care unit; IFN, interferon; IFN-α, interferon alpha; IFN-γ, interferon-gamma; IHC, immunohistochemistry; IL-6, Interleukin 6; IL-12, Interleukin 12; IP-10, Interferon-gamma-induced- protein 10; IR, infrared; i.v., Intravenous; MAS, Macrophage activation syndrome; mRNA, Messenger ribonucleic acid; NSAID, Non-steroidal anti-inflammatory drug; qPCR, Quantitative polymerase chain reaction; RNA-LPX, Liposomally formulated vaccine encoding ribonucleic acid; SRC, Safety Review Committee; TLS, Tumor lysis syndrome; TNF-α, Tumor necrosis factor alpha.

2.4.2 Benefit assessment

Based on the preclinical data, it is possible that the CLDN6 CAR-T/CLDN6 CAR-T(A) alone and in combination with CLDN6 RNA-LPX will exert an anti-tumor effect as only patients with a high expression of CLDN6 on the majority of their tumor cells will be eligible for trial participation. Notably, CLDN6 is homogenously expressed on almost all tumor cells in testicular cancer.

As of September 16th, 2021, ten patients have been treated in the BNT211-01 trial. The initial tumor responses were as follows:

- Part 1, 10^7 CLDN6 CAR-T: 1 of 3 patients has shown a tumor shrinkage of 18% according to RECIST.
- Part 2, 10^7 CLDN6 CAR-T (includes one patient with a product not reaching the target dose): 1 of 4 patients has shown a tumor shrinkage of 29% according to RECIST.

- Part 1, 10^8 CLDN6 CAR-T: 2 of 2 evaluable patients have shown a tumor shrinkage of 17% and 18% according to RECIST. One patient did not have a RECIST evaluation at the data cut-off point.

Initial responses even at low doses of CLDN6 CAR-T indicate the potential benefit of the treatment.

2.4.3 Overall benefit over risk conclusion

Since CLDN6 is exclusively expressed by tumor cells and no protein expression has been detected on any analyzed adult normal tissue, no off-tumor on-target toxicities are expected. Importantly, CLDN6 is known to be expressed on embryonic stem cells, thus pregnant women must not participate in the trial and should not become pregnant thereafter. qRT-PCR analysis suggest that CLDN6 mRNA expression was either negative in the vast majority of normal tissues or slightly above the defined cut-off in a single sample each of a few tissue types (placenta, testis and umbilical cord, cerebellum, lung). Notably, CLDN6 protein was not detected in any of these samples. Even though on-target, off-tumor toxicities are not expected, they cannot be ruled out, hence patients will be observed clinically for 14 d after CLDN6 CAR-T/CLDN6 CAR-T(A) and RNA-LPX administration.

To date, RNA-LPX based cancer vaccines have demonstrated a favorable safety and tolerability profile in different indications, in different treatment settings (metastatic, post neo-adjuvant, adjuvant), and with different types of cancer vaccine antigens (see IB for details). In the clinical trials with RNA-LPX, no DLTs were reported during dose escalation, the TEAEs considered related to trial drug were transient, mostly Grade 1 and 2, and associated with the specific format of encoding and delivering the vaccine antigens, namely single-stranded RNA formulated as a 1,2-di-O-octadecenyl-3-trimethylammonium propane chloride/dioleoly-sn-glycero-phophoethanolamine (DOTMA/DOPE) lipoplex. None of the potential risks, except for class-intrinsic risks, described above is considered likely and relevant for the patients. Based on the experience from the ongoing clinical trials with RNA-LPX, the class-intrinsic potential risks are expected to manifest as mild-to-moderate, transient and manageable flu-like AEs and transient lymphopenia (by sequestration).

The occurrence of AEs related to LD, CLDN6 CAR-T/CLDN6 CAR-T(A), and CLDN6 RNA-LPX may overlap making it difficult to assess the causality at the time of occurrence.

As of September 16th 2021, four patients have been treated with the combination of CLDN6 CAR-T and CLDN6 RNA-LPX in the BNT211-01 trial. Apart from the anticipated flu-like symptoms occurring within hours of CLDN6 RNA-LPX injection, no aggravation of adverse events attributed to CLDN6 CAR-T could be confirmed although it is premature to draw conclusions based on the limited number of CLDN6 RNA-LPX injections administered.

Risks such as myelosuppression with associated cytopenias and febrile neutropenias are known risks of the LD chemotherapy. Lymphodepletion as such cannot be avoided at this stage of clinical development as it was shown to be beneficial for T-cell engraftment and persistence. To account for the fact that patients may experience febrile neutropenias at the same time as the signs and symptoms of cytokine release syndrome (CRS) typically occurred in patients treated with CD19 CAR-T, all patients will be monitored as in-patients for 14 days after the administration of CLDN6 CAR-T/CLDN6 CAR-T(A). As of September 16th 2021, pronounced cytopenias were observed in patients with testicular cancers who were pretreated with a recent HDCT/ASCT.

After careful assessment of all emerging safety, expansion and persistence data the sponsor has opened a LD-free cohort in Part 2 of the trial, to further lower the risk for the patients. To further mitigate the risk of cytopenias, patients receiving LD after a HDCT/ASCT should have an autologous stem cell back-up. Any questions related to allocation of the patients to the LD-free cohort will be discussed with the SRC per SRC charter.

Based on inclusion criteria and published literature, eligible patients will not have available standard treatment options, leaving a very poor prognosis for this population. It is possible that the CLDN6 CAR-T/CLDN6 CAR-T(A) therapy alone or with CLDN6 RNA-LPX will exert an anti-tumor effect, as demonstrated by first clinical data, as well as preclinical data in mice, for patients with advanced CLDN6 expressing solid tumors.

Taken together, the risk-benefit ratio in the heavily pretreated patient population suffering from advanced tumor diseases is regarded as positive.

Nevertheless, all ongoing and planned clinical trials contain regular safety monitoring of laboratory and clinical parameters to ensure patient safety and well-being.

3 OBJECTIVES AND ENDPOINTS

Objectives and related endpoints are described in Table 14.

Table 14: Objectives and endpoints

Objectives	Endpoints
**Primary**	
To assess the safety and tolerability of CLDN6 CAR-T/CLDN6 CAR-T(A) +/- CLDN6 RNA-LPX and to assess the comparability of CLDN6 CAR-T and CLDN6 CAR-T(A)	• Occurrence of TEAEs within a patient including ≥ Grade 3, serious, fatal TEAEs by relationship<br>• Occurrence of dose reduction and discontinuation of IMP within a patient due to TEAEs
To identify the MTD/RP2D for each IMP (i.e. CLDN6 CAR-T/CLDN6 CAR-T(A) +/- CLDN6 RNA-LPX) based on the occurrence of DLTs using the following definitions:<br>• MTD is defined as the highest tolerated dose of CLDN6 CAR-T/CLDN6 CAR-T(A) +/- CLDN6 RNA-LPX where less than 33% of the patients experience a DLT<br>• RP2D of CLDN6 CAR-T/CLDN6 CAR-T(A) +/- CLDN6 RNA-LPX based on integrated evaluation of safety and other data for all dose levels tested	• Occurrence of DLTs within a patient during the DLT evaluation period
**Secondary**	
To describe the profile of soluble immune factors in CLDN6 CAR-T/CLDN6 CAR-T(A) +/- CLDN6 RNA-LPX	• Change from baseline in the levels and kinetics of soluble immune factors measured by cytokine multiplex assay
To evaluate anti-tumor activity of CLDN6 CAR-T/CLDN6 CAR-T(A) +/- CLDN6 RNA-LPX according to response evaluation criteria in solid tumors version 1.1 (RECIST 1.1)	• ORR defined as the proportion of patients in whom a CR or PR (per RECIST 1.1) is observed as best overall response<br>• Disease control rate (DCR) defined as the proportion of patients in whom a CR or PR or SD per RECIST 1.1 (SD assessed at least 6 weeks after the first

Objectives	Endpoints
	dose) is observed as best overall response<br>• DOR defined as the time from first objective response (CR or PR per RECIST 1.1) to first occurrence of objective PD per RECIST 1.1/recurrence or death from any cause, whichever occurs first
**Exploratory**	
To evaluate anti-tumor activity of CLDN6 CAR-T/CLDN6 CAR-T(A) +/- CLDN6 RNA-LPX according to immune response evaluation criteria in solid tumors (iRECIST)	• Objective response rate (iORR) per iRECIST defined as the proportion of patients in whom a complete response or partial response per iRECIST (iCR or iPR) is observed as best overall response<br>• Disease control rate (iDCR per iRECIST) defined as the proportion of patients in whom an iCR or iPR or stable disease (iSD) per iRECIST (iSD assessed at least 6 weeks after the first dose), is observed as best overall response<br>• iDOR defined as the time from first objective response (iCR or iPR per iRECIST) to first occurrence of objective tumor progression (iCPD per iRECIST) or death from any cause, whichever occurs first
To evaluate efficacy of CLDN6 CAR-T/CLDN6 CAR-T(A) +/- CLDN6 RNA-LPX	• PFS defined as the time from first dose of CLDN6 CAR-T/CLDN6 CAR-T(A) to first objective PD per RECIST 1.1, or death from any cause, whichever occurs first<br>• PFS defined as the time from first dose of CLDN6 CAR-T/CLDN6 CAR-T(A) to first objective iCPD, or death from any cause, whichever occurs first

Objectives	Endpoints
	• OS defined as the time from first dose of CLDN6 CAR-T/CLDN6 CAR-T(A) to death from any cause<br>• TTF in patients treated with CLDN6 RNA-LPX, defined as time from first injection to discontinuation of treatment for any reason including disease progression, treatment toxicity, add-on of new anti-cancer therapy, and death
Preliminary assessment of biomarkers that might act as pharmacodynamic, anti-tumor, and safety indicators of activity of CLDN6 CAR-T/CLDN6 CAR-T(A) +/- CLDN6 RNA-LPX	• Detection, phenotypic characterization and functionality of CLDN6 CAR-T/CLDN6 CAR-T(A) detected by flow cytometry in blood (and ascites, pleural effusion, if available)<br>• Assessment of blood tumor biomarkers<br>• Change of CLDN6 expression in tumor biopsy<br>• Immune signature of the tumor<br>• Anti-CAR antibodies, if clinically indicated
To characterize the *in vivo* cellular PK profile (levels, persistence, trafficking) of CLDN6 CAR-T/CLDN6 CAR-T(A) in target tissues (blood and other tissues if available)	• Presence of CLDN6 CAR-T/CLDN6 CAR-T(A) in blood and other tissues, if available<br>• Maximum concentration (C_{max}), time to maximum concentration (T_{max}), AUC and other relevant PK parameters of CLDN6 CAR-T/CLDN6 CAR-T(A) in blood<br>• Additionally for patients crossing over from Part 1 to additional cohort in Part 1 (CLDN6 CAR-T/CLDN6 CAR-T(A) + CLDN6 RNA-LPX): re-expansion of CLDN6 CAR-T in blood and other tissues

AUC, Area under the time-concentration curve; CAR, Chimeric antigen receptor; CAR-T, Chimeric antigen receptor T cell; CLDN6, claudin 6; C_{max}, Maximum concentration; CR, Complete response; DCR, Disease control rate; DLTs, Dose-limiting toxicities; DOR, Duration of response; iCPD, Immune confirmed progressive disease; IMP, Investigational Medicinal Product; iRECIST, Immune RECIST; MTD, Maximal tolerated dose; ORR, Objective response rate; OS, Overall survival; PD, Disease progression; PFS, Progression-free survival; PK, Pharmacokinetics; PR, Partial response; qPCR, Quantitative polymerase chain reaction; RECIST, Response Evaluation Criteria in Solid

Tumors; RNA-LPX, Liposomally- formulated vaccine encoding ribonucleic acid; RP2D, Recommended Phase 2 dose; SD, Stable disease; TEAE, Treatment-emergent adverse event; T_{max}, Time to reach maximal concentration; TTF, Time to treatment failure.
"i" indicates immune responses assigned using iRECIST.

4 TRIAL DESIGN

4.1 OVERALL DESIGN

This is a FIH, open-label, multicenter Phase I/IIa dose escalation trial of CLDN6 CAR-T/CLDN6 CAR-T(A) +/- CLDN6 RNA-LPX with expansion cohorts in patients with CLDN6 positive relapsed or refractory advanced solid tumors.

Patients with any of the following tumor types will be pre-screened to determine appropriate CLDN6 status: Testicular, ovarian, gastric, endometrial, NSCLC, advanced or metastatic cancer, including rare tumors, that fulfills other inclusion criteria and for whom there is no available standard therapy likely to confer clinical benefit, or patient is not a candidate for such available therapy. Only patients whose tumor exerts intermediate to strong CLDN6 antigen expression defined as ≥ 50% of tumor cells with ≥ 2+ CLDN6 staining per IHC analysis in a central laboratory are eligible for this trial.

The trial is outlined in Figure 1 and consists of three parts:

For CLDN6 CAR-T manufactured using a manual process (IMP-1), the following applies:

Part 1 will be a CLDN6 CAR-T dose escalation in lymphodepleted patients until the MTD and/or RP2D of CLDN6 CAR-T as monotherapy are defined (Figure 2).

In case the MTD for CLDN6 CAR-T is identified at dose level 1, the sponsor together with the SRC may explore lower dose levels for CLDN6 CAR-T dose escalation and for bifurcation from CAR-T to CAR-T + RNA-LPX dose escalation, based on safety and other available data.

Part 2 will be a vaccine-modulated dose escalation until the MTD and/or RP2D of CLDN6 CAR-T + CLDN6 RNA-LPX are defined. Patients will be pretreated with an LD regimen (standard, Figure 3), or they may be pretreated with a reduced-dose LD regimen (optional, Figure 4) or without LD (optional Figure 5), as decided by the SRC per SRC charter.

Part 2 is planned to start before the MTD/RP2D is reached in Part 1 using a bifurcated trial design. Bifurcation is planned to start when Part 1 CLDN6 CAR-T dose level 1 (1×10^7 CLDN6 CAR-T) evaluation is deemed safe. At this point, Part 2 will start with the cleared CLDN6 CAR-T dose level. CLDN6 RNA-LPX will be administered at fixed step-up doses as described in Section 4.1.1 and dose modifications defined in Section 6.6.

The dose level of CLDN6 CAR-T in Part 2 at any given time will not exceed that in Part 1. This approach will allow for rapid and safe triggering of the vaccine-modulated dose escalation. This will lead to the timely optimization of the schedule that will be taken to the expansion phase. At the same time, patient safety is safeguarded by generating enough data on CAR-T before bifurcation, and clear rules for parallel dose escalations are outlined. The enrollment of patients into either CAR-T or vaccine-modulated dose escalation cohorts will be alternated. For any exception, discussion between the investigator and the SRC is required.

In Part 2, patients will be enrolled in cohorts including the LD chemotherapy as shown in Figure 3. In order to assess if LD-related toxicities can be avoided, cohorts testing vaccine-modulated CLDN6 CAR-T without LD (Figure 5) may also be activated in patients with CLDN6-positive solid tumors, if satisfactory expansion and persistence is seen at a certain dose level. All decisions concerning opening the LD-free cohorts will be made by the SRC.

Selected dose cohorts may be backfilled in both Parts 1 and 2 to explore safety and anti-tumor activity further after discussion with the SRC (refer to Section 4.1.1).

Patients treated in Part 1 may be additionally treated with CLDN6 RNA-LPX according to the SoA (Section 1.3.2) under the following conditions:

- The dose level has been deemed safe by the SRC.
- The patient consents to the additional treatment in a separate consent form.
- The SRC approves the use of CLDN6 RNA-LPX based on the available safety and CLDN6 CAR-T expansion data of the respective patient.
- Safety criteria include:
    - No significant, unacceptable or irreversible toxicities related to trial treatment.
    - The patients must meet the inclusion criteria for hematologic, renal, hepatic and coagulation functions.

For all patients, the following applies:

All patients will undergo leukapheresis for collection of blood product to manufacture the CLDN6 CAR-T/CLDN6 CAR-T(A). Repeat leukapheresis is allowed after discussion between the Investigator and the Sponsor’s Medical Monitor.

In Parts 1 and 2, patients will receive one infusion of CLDN6 CAR-T/CLDN6 CAR-T(A) as described in Section 2.2.2. In case CAR-T number is below the intended dose level, the patient will be treated and followed up per trial procedure, but replaced in the respective dose level cohort for DLT evaluation. Re-dosing with CAR-T is allowed if requested by the investigator, the IMP is available, and endorsed by the SRC. CLDN6 CAR-T/CLDN6 CAR-T(A) re-dosing may only occur after the patient has completed the DLT period without any DLTs being observed. Further details on re-dosing can be found in Section 6.1.3.2.1. During vaccine-modulated dose escalation with CLDN6 RNA-LPX, treatment will continue until

protocol-defined treatment discontinuation criteria are met. Efficacy will be assessed by on-treatment imaging at Week 6 (±7 d), every 6 weeks (±7 d) for 50 weeks, and every 12 weeks (±7 d) thereafter until disease progression is assessed by the investigator, the start of new anti-cancer therapy, withdrawal of consent, or death, whichever occurs first. The response evaluation criteria in solid tumors (RECIST) 1.1 criteria will be used for secondary endpoint response evaluation (Eisenhauer et al. 2009); immunotherapeutics response evaluation criteria in solid tumors (iRECIST) will be used for exploratory endpoint response evaluation (Seymour et al. 2017). CLDN6 RNA-LPX treatment will continue until PD has been verified. All images obtained must be submitted to the central imaging vendor.

Part 3 will start only under the condition that safety data from Parts 1 and 2 indicate a continued positive benefit to risk ratio; data supporting this is submitted to Competent Authorities and Ethics Committees via a substantial amendment; and Part 3 is approved by the respective Competent Authorities and IECs based on that data.

In Part 3, when the RP2D of CLDN6 CAR-T/CLDN6 CAR-T(A) +/- CLDN6 RNA-LPX is established and endorsed by the SRC, pre-defined expansion cohorts will be activated. Additional cohorts can be implemented by a protocol amendment. Clinical assessments will follow the SoA (Section 1.3) according to the treatment chosen (CLDN6 CAR-T/CLDN6 CAR-T(A) with or without CLDN6 RNA-LPX).

4.1.1 Dose escalation

4.1.1.1 Dose escalation in Part 1

The CLDN6 CAR-T/CLDN6 CAR-T(A) dose escalation follows the classical 3+3 design described in Table 15.

Table 15: Dose escalation

Number of evaluable patients with DLT at a given dose level after the first treatment cycle	Escalation decision rules
0 out of 3<br>OR<br>1 out of 6	Enter more patients at the next higher dose level
1 out of 3	Enter more patients at this dose level to a total of at least six evaluable patients or two patients with DLT
2 out of 3<br>OR<br>At least 2 out of 6	Dose escalation will be stopped, the MTD will be considered to be reached at the one before the last dose level explored. The sponsor will decide, based on a discussion between the investigators and the sponsor at an SRC meeting, if additional patients need to be enrolled and at which dose to finalize the trial
Any condition which would require further clarification of safety	A cohort of six patients can eventually be extended and more patients can be enrolled in a cohort if decided by the sponsor based on a discussion between the investigators and the sponsor at an SRC meeting

Abbreviations: DLT, dose-limiting toxicity; MTD, maximum tolerated dose.

The next dose level cohort can only be opened when it is endorsed by the SRC. For additional details regarding the SRC, see Section 9.6 of the protocol and the SRC Charter. The DLT period is defined as 28 d starting from the day of CLDN6 CAR-T/CLDN6 CAR-T(A) therapy administration (ACT, d1).

During dose escalation, all patients will be hospitalized for 14 d for clinical observation after the administration of the CLDN6 CAR-T/CLDN6 CAR-T(A). Prolongation of hospitalization is allowed at the discretion of the investigator. Hospitalization time can be shortened to d7 at the discretion of the SRC in case the patient shows no signs of CRS/ICANS. The timeline for Part 1 is shown in Figure 2.

The dose escalation with CLDN6 CAR-T manufactured with the manual process will evaluate CLDN6 CAR-T at three dose levels: $1x10^7$ CAR-T (total), $1x10^8$ CAR-T (total), and $1x10^9$ CAR-T (total), following the staggering rules outlined in Figure 11.

Staggering:

During the 3+3 phase with IMP-1, at every dose level to be tested, staggered enrollment will be employed as follows: in each dose level cohort, the three patients will be dosed successively with a safety monitoring interval of at least 28 d between the infusion of CAR-T in the first and the second patient (LD may be initiated within this period, 5 d prior to administration of CLDN6 CAR-T), and at least 1 d between the second and the third patient. Two patients cannot be dosed on the same day.

Figure 11: Guidance for staggered enrollment

The dose escalation with CLDN6 CAR-T(A) manufactured with the automated process (IMP-1(A)) will evaluate CLDN6 CAR-T(A) at four dose levels:1x10^6, 1x10^7 CAR-T (total), 1x10^8 CAR-T (total), and 2x10^8 to 5x10^8 CAR-T (total), following the staggering rules outlined below:

The dose escalation will start with two cohorts (testicular cancer patients with recent HDCT and other indications). One patient with a testicular cancer and one patient with another indication will be treated with 1x10^6 CLDN6 CAR-T(A) (DL0). In case of a DLT additional two patients (testicular cancer patients and other indication patient will be considered separately) will be enrolled. In case there are no DLTs within 28 days, the trial continues with the 3+3 design at DL1. During the 3+3 phase, at every dose level to be tested, staggered enrollment will be employed as follows: in each dose level cohort, the three patients will be dosed successively with a safety monitoring interval of at least 14 d between the infusion of CAR-T in the first and the second patient (LD may be initiated within this period, 5 d prior to administration of CLDN6 CAR-T(A)), and at least 1 d between the second and the third patient. Two patients cannot be dosed on the same day.

To be eligible for DLT assessment, patients should receive the first full infusion of the CLDN6 CAR-T/CLDN6 CAR-T(A) at the intended dose without interruption and modification. Patients who do not fulfill the criteria for the DLT assessment will be replaced. Replaced patients will be followed in the trial per trial procedure but not assessed for DLT.

After completion of the DLT period for each cohort, the SRC will review the data from the DLT period – including but not limited to all relevant safety and other data. Thereafter, the SRC will propose the dose level for the next cohort of patients or decide that RP2D has been established and no more patients should be enrolled.

4.1.1.1.1 Maximum tolerated dose and recommended Phase 2 dose definitions

The MTD is defined as the highest tolerated dose.

The RP2D will be determined based on integrated evaluation of safety and other data for all dose levels tested. The RP2D will be determined according to the following guidelines:

1. The RP2D will not exceed the MTD.
2. Toxicities other than DLTs will be considered even in the replaced patients, and the following are taken into account: AEs assessed as related to CLDN6 CAR-T/CLDN6 CAR-T(A) treatment but not considered dose-limiting, the nature and frequency of toxicities, and the emergence of any specific category of toxicities.
3. Evidence of clinical activity, as available.
4. Available biomarker data.

If the RP2D cannot be distinguished using the criteria above, cohort expansion for optimized RP2D determination may take place to obtain data for up to six additional patients per dose level. Selection of RP2D will then be based on this larger dataset. If serious related toxicities are observed after DLT observation period, a reduction of the MTD and/or adjustment of RP2D may be considered. This determination will be made by dose-expansion cohort and endorsed by the SRC.

4.1.1.2 Dose escalation in Part 2

The rules and details on bifurcated design are outlined in Section 4.1.

The dose escalation in Part 2 follows the classical 3+3 design and the same principles including staggering as described in Section 4.1.1.1 and Section 4.1.1.1.1. However, in a case when any dose level in Part 2 is deemed unsafe while the Part 1 is ongoing, dosing in Part 2 should stop and the MTD is declared for the vaccine-modulated dose escalation. The CAR-T dose escalation in Part 1 can proceed independently of Part 2 until MTD and/or RP2D of CLDN6 CAR-T/CLDN6 CAR-T(A) are established. Refer to Section 6.6.5 for safety stopping criteria.

In Part 2, d1 is considered the day when CLDN6 CAR-T/CLDN6 CAR-T(A) is administered. CLDN6 RNA-LPX is administered routinely on d4 in the standard and LD-free cohort and on d24 in the optional cohort with LD. This will be decided based on the safety data by the SRC. The goal is to avoid toxicities related to high CAR-T expansion. The DLT observation period in Part 2 is 28 d starting from d1 until d28. Hospitalization in Part 2 takes place from d1 until d14. Thereafter, the patient will be hospitalized as needed after each administration of CLDN6 RNA-LPX, but at least until improvement of any AE to Grade 2 or lower. Prolonged hospitalization is allowed at the discretion of the investigator and hospitalization time may be reduced if there is no safety contraindication at the discretion of the SRC. The timelines for Part 2 (standard and optional) are shown in Figure 3, Figure 4, and Figure 5.

4.1.2 Dose-limiting toxicity

In general, a DLT for a drug or other treatment is defined as a related AE that prevents an increase of the dose level of that treatment.

For the purpose of dose escalation, the DLT monitoring period will be 28 d from d1 in both Parts 1 and 2. The treatment-emergent occurrence of any of the toxicities outlined in this section will be considered a DLT if assessed by the investigator to be at least possibly related to CLDN6 CAR-T/CLDN6 CAR-T(A) or CLDN6 RNA-LPX. Toxicities clearly not related to the two IMPs (e.g., disease progression and intercurrent illness) will not be considered a DLT. The NCI CTCAE v.5.0 will be used to grade the intensity of AEs.

The definitions of DLT will be categorized as hematologic AEs and non-hematologic AEs.

Hematologic DLT

- Prolonged anemia, neutropenia or thrombocytopenia CTCAE Grade ≥ 4 not resolved to ≤ Grade 2 within DLT period, which is newly developed and not attributable to lymphodepleting chemotherapy.

Non-hematologic DLT

- Any Grade 5 related AEs.
- Any non-hematologic Grade ≥ 3 clinically relevant AE related to any of the IMPs will be considered as DLT with the following exceptions:

1. Fever > 40°C (i.e., Grade ≥ 3) that occurs within 48 h of infusion and resolves within 48 h to 39°C to 40°C (Grade ≤ 2) and fully resolves within 1 week.
2. Grade ≤ 3 nausea or vomiting that resolves to Grade ≤ 1 with or without supportive therapy within 1 week.
3. Laboratory abnormalities that are judged not to be clinically relevant or events that can be promptly controlled by appropriate medical management.
4. Manageable tumor lysis syndrome (TLS) Grade ≤ 4. Manageable means: Patients with TLS must be responsive to treatment as recommended in the protocol (see Section 6.6.4.5).
5. Manageable CRS Grade ≤ 4, as CRS is a clinical sign of expansion and function of CAR-T. Manageable means: Patients with CRS must be responsive to treatment with tocilizumab or corticosteroids per toxicity management plan, otherwise the occurrence of CRS will be considered a DLT (Notes: every case of Grade ≥ 4 CRS and every case of Grade ≥ 3 neurologic AE will be discussed with the SRC).
6. Grade ≤ 3 hypophosphatemia and transient Grade ≤ 3 hyperbilirubinemia resolved within 1 week.
7. Grade ≥ 3 fatigue that resolves to Grade ≤ 2 within 1 week.
8. Infusion-related reactions (IRRs) are not considered to be DLTs because on the basis of experience, IRRs are not dose-related events.
9. Grade ≤ 3 arthralgia that can be adequately managed with supportive care or that resolves to Grade ≤ 2 within 1 week.
10. Grade ≤ 3 diarrhea, colitis, or enteritis that resolves to Grade < 1 within 7 d with no fever or dehydration.

11. Grade ≤ 3 tumor pain that starts within 24 h of infusion/injection and resolves to Grade ≤ 2 within 1 week.
12. Grade ≤ 3 hypoxia that starts within 24 h of infusion/injection and resolves to Grade ≤ 2 within 4 d.
13. In patients with lung lesions, Grade ≤ 3 transient dyspnea secondary to localized lung edema that starts within 24 h of infusion/injection and recovers to Grade ≤ 1 or baseline within 1 week, and transient bronchospasm that resolves within 24 h.
14. In patients with liver lesions, Grade ≤ 3 transient increase of bilirubin, transaminases and/ or gamma-glutamyl transferase that starts within 24 h of infusion/injection and recovers to Grade ≤ 1 or baseline within 1 week.
15. Alopecia of any grade.

Other clinically significant toxicities, including a single event or multiple occurrences of the same event, may be considered as DLTs.

AEs occurring after DLT observation period may be considered DLTs upon discussion between the Investigator and the Sponsor's Medical Monitor.

Any other toxicity assessed as related to IMP, and which in the opinion of the trial Investigator and the Sponsor's Medical Monitor constitutes a DLT.

Patients experiencing a DLT (an AE fulfilling the DLT criteria within the DLT period of 28 d) should discontinue IMP immediately.

AEs fulfilling the DLT criteria must be documented on an SAE report form and reported to the sponsor **within 24 h** as described in Section 12.3.4.

4.1.3 Expansion (Part 3)

Expansion phase comprises cohorts in pre-defined patient populations and may be amended based on data generated in both Parts 1 and 2. The expansion phase will assess safety and efficacy in the following cohorts:

- Cohort 1: Testicular cancer
- Cohort 2: Ovarian cancer
- Cohort 3: Gastric cancer
- Cohort 4: Endometrial cancer
- Cohort 5: NSCLC
- Cohort 6: Tumors not otherwise specified (NOS), including rare tumors and cancers of unknown primary

4.2 PLANNED NUMBER OF PATIENTS

In Part 1 and Part 2 (manual manufacturing process) the number of patients will be up to 18 DLT-evaluable patients in each part depending on the number of DLTs which may occur.

In Part 1 and Part 2 (automated manufacturing process) the number of patients will be up to 96 DLT-evaluable patients depending on the number of DLTs which may occur.

For Part 3, the final sample size calculations will be introduced through protocol amendment.

4.2.1 Replacement of patients

Patients who are not evaluable for DLTs will be replaced.

DLT-evaluable patients are defined as all treated patients who received the first full infusion of the CLDN6 CAR-T/CLDN6 CAR-T(A) at the intended dose without interruption and modification in Part 1; and one full infusion of the CLDN6 CAR-T/CLDN6 CAR-T(A) at the intended dose without interruption and modification and at least one full dose of vaccination in Part 2, or have experienced a DLT during the DLT evaluation period.

Patients who do not experience a DLT during DLT evaluation period are considered evaluable if they have been observed for a minimum of 28 d following the first dose and are considered to have sufficient safety data to conclude that a DLT did not occur.

4.3 TRIAL DESIGN RATIONALE

In order to address the trial objectives in this FIH trial, a dose escalation using the classical 3+3 design was selected. The classical 3+3 design is appropriate for FIH dose escalation trials in oncology; however, this trial will consider all data generated to decide on the RP2D. The

safety data, cellular PK, biomarkers and clinical efficacy combined provide both more flexibility and better accuracy of the estimated RP2D while safeguarding the patient's safety.

The first part of this trial is a FIH, open-label, dose escalation trial testing CLDN6 CAR-T monotherapy in lymphodepleted patients with CLDN6-positive relapsed and refractory advanced solid tumors in order to determine the safety profile of CLDN6 CAR-T.

The second part aims to determine further the safety profile of CLDN6 CAR-T/CLDN6 CAR-T(A) in conjunction with CLDN6 RNA-LPX, which acts as the modulator of CLDN6 CAR-T/CLDN6 CAR-T(A). First, patients will undergo the LD regimen as shown in Figure 3. In order to assess if LD-related toxicities can be avoided, a cohort testing vaccine-modulated CLDN6 CAR-T/CLDN6 CAR-T(A) without LD may also be activated upon approval of the SRC. The prerequisite is satisfactory expansion and persistence at the selected dose level.

The third part is the expansion phase to explore further CLDN6 CAR-T/CLDN6 CAR-T(A) as a monotherapy or modulated by CLDN6 RNA-LPX in select tumor indications. Different treatment schedules may also be explored in Part 3.

4.3.1 Rationale for bifurcated design

The clinical development of combination therapies in oncology follows the tradition, where individual molecules are assessed in the clinic as monotherapy first, followed by combination trials performed after clear monotherapy proof-of-concept has been established. In many instances, failure to demonstrate substantial monotherapy activity led to cessation of further clinical development.

This approach may not be well suited for immunotherapy agents, where development efforts can be terminated prematurely based on unrealistic expectations for monotherapy activity using conventional criteria. More recently, driven by increasing recognition of the potential of immunotherapeutic combinations and the fact that some immuno-oncology agents may yield only modest clinical activity as monotherapy, yet be highly active in conjunction with a second compound augmenting their mode-of-action, innovative trial designs have been deployed (Bates et al. 2015; Emens et al. 2016).

One of these approaches integrates the combination assessment to the initial clinical development using a bifurcated design, which is pursued in our trial. This approach allows for more rapid triggering of combination testing. It is anticipated that the IMP may have limited potential for monotherapy clinical activity, yet there is clear rationale that this agent may synergize and potentiate when administered with another drug (Ott et al. 2017).

Adoptive cell therapy based on genetically engineered T cells armed with CARs shows tremendous clinical success in patients with B-cell malignancies (Yong et al. 2017). However, only limited anti-tumoral activity was observed in multiple clinical trials upon CAR-T therapy in patients suffering from solid tumors.

Two key hurdles of CAR-T therapy targeting non-hematological neoplasia are i) the lack of highly cancer selective targets providing challenges for safe treatment protocols and, ii) the insufficient expansion and long-term persistence of CAR-T, probably due to inefficient co-stimulation upon antigen-exposure *in vivo*. In particular, the latter point displays a fundamental difference comparing CAR-T treatment of B-cell-derived hematological malignancies and solid tumors. With the help of repetitive CLDN6 RNA-LPX treatment, CLDN6 CAR-T/CLDN6 CAR-T(A) can be expanded *in vivo* in a dose-dependent manner resulting in an improved persistence.

Bifurcated design to establish the safe dose of CLDN6 CAR-T/CLDN6 CAR-T(A) +/- cancer vaccine is justified to accelerate the clinical development in a safe manner.

4.4 DOSE AND SCHEDULE RATIONALE

4.4.1 CAR-T dose rationale

The dosage of infused CAR-T that can generate the best curative effect is unknown. A dose of 10×10^9 CAR-T is beyond what is currently considered a safe dose of CD19 CAR-T, and when used to target a patient's breast cancer resulted in the patient's demise due to CRS (Morgan et al. 2010). Turtle et al considered a CAR-T dose of 2×10^7/kg to be excessively toxic in non-Hodgkin lymphoma patients, and a lower dose was well tolerated (2×10^5/kg, and 2×10^6/kg) and associated with a higher response rate (Turtle et al. 2016b). In a meta-analysis of patients with lymphoma who received CAR-T infusions, a subgroup analysis, found that a CAR-T dose lower than $10^8/m^2$ is associated with increased response rates and reduced incidence rates of CRS (Grade ≥ 3) (Cao et al. 2019). These findings support the 10^7 dose, together with the fact that lower infusion doses suggest lower acute toxicity risks and with no deleterious effects on early memory (Hou et al. 2019).

Selection of the starting dose and dose range of CAR-T is based on common practice for FIH CAR-T approaches determined by a survey of current CAR-T trials in solid tumors (Table 16). Most trials use CAR-T dose ranges from 10^7 to 10^{10} per patient, with the exception of a HER2-CAR trial starting at 10^4 cells per patient. Clinical responses have been observed at doses levels ranging from $1 \times 10^7/m^2$ to $1 \times 10^9/m^2$ CAR-T using second generation CAR constructs.

The selected starting dose for the product generated with the manual manufacturing process is 10^7 CLDN6 CAR-T (total number of CLDN6 CAR-T in the product), followed by two additional dose levels of 10^8 and 10^9 CLDN6 CAR-T. Lower or higher doses may be explored based on the ongoing data generated in this trial.

The selected starting dose for the product generated with the automated manufacturing process, based on the preclinical data is 10^6 CLDN6 CAR-T(A) (total number of CLDN6 CAR-T(A) in the product), followed by three additional dose levels of 10^7, 10^8, and $2x10^8$ to $5x10^8$ CLDN6 CAR-T(A).

Unlike standard drugs that are metabolized, CAR-T are able to proliferate extensively in patients, and thus the actual *in vivo* amount of CAR-T after engraftment and expansion will vary from patient to patient. Thus, the administered dose may underestimate the *in vivo* amount of CAR-T.

Table 16: Review of CAR-T trials on solid tumors

Reference	CAR antigen	Gene-transfer technology	CAR endo-domains	CAR-T dose	Number of patients	Clinical response	CAR-T persistence
Kershaw et al. 2006	α-folate receptor	Gamma-retrovirus	FcRγ	3×10^{9} to 1.69×10^{11} (1 to 3 infusions)	14 patients with ovarian cancer	14 PD	14 to 21 d
Park et al. 2007	CD171	Electroporation	CD3ζ	1×10^{8}/m^{2} to 1.1×10^{9}/m^{2}	6 children with neuroblastoma	1 PR, 5 PD	Short (1 to 7 d) in patients with bulky disease, but significantly longer (42 d) in a patient with a limited disease burden
Lamers et al. 2013	CAIX	Gamma-retrovirus	FcRγ	0.2×10^{9} to 2.1×10^{9} (5 infusions)	12 patients with metastatic renal cell carcinoma	12 NR	Up to 3 to 5 weeks
Louis et al. 2011	Gd2	Gamma-retrovirus	CD3ζ	2×10^{7} cells/m^{2}, 5×10^{7} cells/m^{2}, 1×10^{8} cells/m^{2}	19 patients with neuroblastoma	3 CR, 1 PR, 8 NED, 4 PD, 1 SD, 2 tumor necrosis, 1 relapse	96 to 192 weeks
Morgan et al. 2010	HER2	Gamma-retrovirus	4-1BB-CD28-CD3ζ	10^{10} T cells	1 patient with colorectal cancer	Died of cytokine release syndrome	Died 5 d after treatment
Brown et al. 2015	IL13Rα2	Electroporation	CD3ζ	9.6×10^{8} to 15.35×10^{8} (11 to 17 infusions)	3 patients with glioblastoma	2 PR, 1 relapse	14 weeks
Katz et al. 2015	CEA	Gamma-retrovirus	CD28-CD3ζ	Cohort 1: 10.1×10^{9}; Cohort 2: 30×10^{9} (3 infusions)	6 patients with adenocarcinoma liver metastases	5 PD, 1 SD	Approximately 2 weeks
Ahmed et al. 2015	HER2	Gamma-retrovirus	CD28-CD3ζ	1×10^{4}/m^{2} to 1×10^{8}/m^{2} (1 to 9 infusions)	19 patients with sarcoma	1PR, 12 PD, 4 SD, 2 NE	Up to 18 months
O'Rourke et al. 2017	EGFR	Lentivirus	4-1BB-CD3ζ	5×10^{8}	10 patients with glioblastoma	2 PD, 1 SD, 7 died	< 30 d
Feng et al. 2016	EGFR	Lentivirus	4-1BB-CD3ζ	9.7×10^{6} cells/kg	11 patients with NSCLC	2 PR, 4 PD, 5 SD	> 4 weeks
Gargett et al. 2016	Gd2	Gamma-retrovirus	CS28-OX40	1×10^{7} cells/m^{2}, 2×10^{7} cells/m^{2},	4 patients with metastatic melanoma	4 PR	42 d
Beatty et al. 2014	Mesothelin	Electroporation	4-1BB-CD3ζ	4.4×10^{9} cells, 3×10^{8} cells/m^{2}	2 patients with malignant pleural mesothelioma, pancreatic cancer	2 SD	1 to 2 weeks

Reference	CAR antigen	Gene-transfer technology	CAR endo-domains	CAR-T dose	Number of patients	Clinical response	CAR-T persistence
You et al. 2016	MUC1	Lentivirus	CD28-4-1BB-CD3ζ	5×10^5 cells	1 patient with seminal vesicle cancer	1PR	No information
Zhang et al. 2017	CEA	Lentivirus	CD-28-CD3ζ	2.5×10^7 to 1.5×10^{10} cells	10 patients with metastatic colorectal cancer	2 PD, 7 SD, 1 NE	6 weeks
Junghans et al. 2016	PSMA	Gamma-retrovirus	CD28-CD3ζ	1×10^9 cells, 1×10^{10} cells	5 patients with prostate cancer	2 PR, 1 minor response, 2 NR	4 weeks
Tanyi et al. 2017	Mesothelin	Lentivirus	4-1BB-CD3ζ	3×10^7 cells/m^2	1 patient with ovarian cancer	1 CR	26 d
Tchou et al. 2017	c-Met	Electroporation	4-1BB-CD3ζ	3×10^7 cells, 3×10^8 cells	6 patients with breast cancer	2 PD, 1 SD, 3 died	7 d
Hege et al. 2017	TAG-72	Gamma-retrovirus	CD3ζ	3×10^9 to 4×10^9 cells	6 patients with colorectal cancer	3 PD, 3 not reported	≤ 14 weeks
Maus et al. 2013	Mesothelin	Electroporation	4-1BB-CD3ζ	1×10^8 to 1×10^9 cells	4 patients with pancreatic adenocarcinoma	1 CR, 1 PD, 2 SD, 1 died	No information

Abbreviations: CAIX, Carbonic anhydrase iX; CEA, Carcinoembryonic antigen; c-Met, Hepatic growth factor; CR, Complete response; d, day; EGFR, Epidermal growth factor receptor; FcRγ, Fc gamma receptor; Gd2, Disialoganglioside Gd2; HER2, Human epidermal growth factor receptor 2; MUC, Mucin 1 cell surface associated; NE, Not evaluable; NED, Indicates no evidence of disease; NR, No response; NSCLC, Non-small cell lung cancer; PD, Progressive disease; PR, Partial response; PSMA, Prostate specific membrane antigen; SD, Stable disease; TAG-72, Tumor-associated glycoprotein 72.

4.4.2 CLDN6 RNA-LPX dose rationale

Based on data from ongoing and completed trials, in which several hundreds of patients have been treated with multiple doses, RNA-LPX vaccines have a favorable safety profile characterized by mild-to-moderate, transient and manageable flu-like drug-related AEs. These AEs are expected and correlate with the laboratory changes observed in the clinical trials with RNA-LPX, namely the distinct range of cytokines released by RNA-LPX and transient lymphopenia mediated by Type 1 IFN (by sequestration). The clinically manifested AEs are manageable by analgesics or antipyretics at recommended commonly used dose.

The proposed starting dose of CLDN6 RNA-LPX cancer vaccine is 25 µg on d4 in the standard Part 2 and in the optional Part 2 LD-free cohorts and on d24 in the optional Part 2 cohort with LD. The dose is stepped up to 50 µg in the following administration on d30 in the standard Part 2 and the optional Part 2 LD-free cohorts and on d36 in the optional Part 2 cohort with LD. The same dose is administered in subsequent administrations if it is tolerated. If the starting dose of 25 µg is not tolerated, a dose of 12.5 µg will be explored. If 12.5 µg is tolerated, this TD will be administered on the remaining dosing days: d30 or d36, d51, d72, d93, and then every 6 weeks. Further guidance on dose modifications is described in Section 6.6 of the protocol.

The proposed intra-patient- titration dosing schedule of RNA-LPX as a single agent is based primarily on the safety, immunogenicity, and preliminary efficacy data emerging from trials conducted by the sponsor (refer to the IB).

Step-up dosing may offer advantages over flat dosing, including the potential mitigation of a hypothetical first-dose phenomenon (i.e., allowing the patient to acclimate to gradually increasing doses of RNA-LPX) with the lower starting dose acting as a "lead-in" dose, potentially mitigating immune system activation-related AEs.

4.5 END OF TRIAL DEFINITION

A patient is considered to have completed the trial if he/she has completed all phases of the trial including the last scheduled procedure of the End of Trial (EoT) Visit at Month 25 shown in the SoA tables (Part 1: Table 2, Part 1 cross-over: Table 3, Standard Part 2: Table 5, Optional Part 2: Table 7, and Optional Part 2 without LD: Table 9).

The end of the trial is defined as the date of the last scheduled procedure of the EoT Visit at Month 25 shown in the SoA for the last patient in the trial globally, or the sponsor discontinues the trial.

5 TRIAL POPULATION

Prospective approval of protocol deviations to recruitment and enrollment criteria, also known as protocol waivers or exemptions, is not permitted.

5.1 INCLUSION CRITERIA

Each potential patient must fulfill all of the following criteria to be enrolled in the trial.

- **Disease-specific inclusion criteria**

For all Parts 1, 2 and 3:

1. Each patient enrolled in the trial must have CLDN6-positive tumor regardless of tumor histology defined as ≥ 50% of tumors expressing ≥ 2+ CLDN6 protein using a semi-quantitative IHC assay in a central laboratory for specific detection of CLDN6 protein expression in formalin-fixed, paraffin-embedded (FFPE) neoplastic tissues.
2. Availability of a FFPE tumor tissue sample. FFPE can be from an archival tumor tissue sample, and it should be from the most recent tumor tissue obtained. If this is not available, patient must be biopsied for CLDN6 staining.
3. Must have histological documentation of the original primary tumor via a pathology report.
4. Must have measurable disease per RECIST 1.1 (except for germ cell tumors, where patients can be evaluated according to CA-125, AFP or hCG [as applicable] if they have a pre-treatment sample that is at least twice the upper limit of normal).

For Parts 1 and 2:

5. Must have a histologically confirmed solid tumor that is metastatic or unresectable and for which there is no available standard therapy likely to confer clinical benefit, or patient who is not a candidate for such available therapy.

For Part 2 only:

Cohort 7: Vaccine-modulated CLDN6 CAR-T/CLDN6 CAR-T(A) without lymphodepletion

6. Histologically or cytologically confirmed solid tumor fulfilling inclusion criteria 1-4 that is metastatic or unresectable, and for whom there is no available standard therapy likely to confer clinical benefit, or patient who is not a candidate for such available therapy.

For Part 3 only:

Cohort 1: Testicular cancer

7. Histologically or cytologically confirmed tumor of the testis of any histological subtype that has relapsed and/or is refractory to standard treatment. There is no limit on the number of prior treatment regimens.

Cohort 2: Ovarian cancer

8. Histologically or cytologically confirmed ovarian cancer of any histology type including primary peritoneal or fallopian tube tumor that is resistant to a platinum-based chemotherapy regimen, or for whom there is no available standard therapy likely to confer clinical benefit, or patient is not a candidate for such available therapy. There is no limit on the number of prior treatment regimens.
9. Patients without initial measurable disease per RECIST 1.1 and evaluable by CA-125 are eligible for the trial and the tumor response will be assessed by Gynecologic Cancer Intergroup (GCIG) criteria for evaluation of best overall response in patients without initial measurable disease and evaluable by CA-125.

Note: Patients can be evaluated according to CA-125 only if they have a pre-treatment sample that is at least twice the upper limit of normal.

Cohort 3: Gastric cancer

10. Histologically or cytologically confirmed inoperable or metastatic gastric cancer that has failed or demonstrated intolerance to standard therapy - which includes platinum or fluoropyrimidine or taxane-based chemotherapy. HER2-positive gastric cancer patient must have failed or demonstrated intolerance to HER2-targeting treatment. There is no limit on the number of prior treatment regimens.

Cohort 4: Endometrial cancer

11. Histologically or cytologically confirmed endometrial cancer of any histology type that is resistant to a platinum-based chemotherapy regimen, or for whom there is no available standard therapy likely to confer clinical benefit, or patient is not a candidate for such available therapy. There is no limit on the number of prior treatment regimens.

Cohort 5: NSCLC

12. Histological or cytological diagnosis of metastatic non-squamous NSCLC and must have progressed on the standard therapy, including platinum-based chemotherapy and/or checkpoint inhibitor therapy. Patient with EGFR, ALK, receptor tyrosine kinase encoded by gene ROS-1 (ROS-1) or BRAF mutations must have progressed on standard treatment options including EGFR, ALK, ROS-1, and BRAF directed therapies. There is no limit on the number of prior treatment regimens.

Cohort 6: Tumors not otherwise specified, including rare tumors and cancers of unknown primary

13. Advanced or metastatic cancer that fulfills other inclusion criteria and for whom there is no available standard therapy likely to confer clinical benefit, or patient is not a candidate for such available therapy. There is no limit on the number of prior treatment regimens.

- **Other inclusion criteria**

For all Parts 1, 2 and 3:

14. Must be ≥ 18 years of age at the time the pre-screening informed consent is signed.
15. Must sign an informed consent form (ICF) indicating that he or she understands the purpose of and procedures required for the trial and are willing to participate in the trial prior to any trial-related assessments or procedures.
16. Must have an Eastern Cooperative Oncology Group performance status of 0 to 1.
17. Must have adequate coagulation function at screening as determined by:
    a. International normalized ratio (INR) or prothrombin time ≤ 1.5 × upper limit of normal (ULN; unless on therapeutic anticoagulants with values within therapeutic window),
    b. Activated partial thromboplastin time (aPTT) ≤ 1.5 × ULN (unless on therapeutic anticoagulants with values within therapeutic window).
18. Must have adequate hematologic function at screening as determined by:
    a. White blood count (WBC) ≥ 3 × 10^9/L
    b. Absolute neutrophil count (ANC) ≥ 1.5 × 10^9/L (patient may not use Granulocyte-colony-stimulating factor (G-CSF) or granulocyte-macrophage colony-stimulating factor (GM-CSF) to achieve these WBC and ANC levels)
    c. Platelet count ≥ 100 × 10^9/L
    d. Hemoglobin (Hgb) ≥ 9.0 g/dL (may not transfuse or use erythropoietin to obtain this Hgb level).
19. Must have adequate hepatic function at screening as determined by:
    a. Total bilirubin ≤ 25.65 µmol/L (or ≤ 34.2 µmol/L for patients with known Gilbert's syndrome)
    b. Aspartate aminotransferase (AST) and alanine aminotransferase (ALT) ≤ 2.5 × ULN; ≤ 5 × ULN for patients with liver metastasis.
20. Must have adequate renal function at screening as determined by:
    Glomerular filtration rate (GFR) ≥ 30 mL/min/1.73 m² – according to the abbreviated Modification of Diet in Renal Disease equation:

    $$GFR = 175 \times (\text{standardized SCr}^{-1.154}) \times (\text{age}^{-0.203})$$

    (where SCr, the serum creatinine level, is expressed in mg/dL; multiply it by 0.742 if the patient is female; multiply it by 1.212 if the patient is of black ethnicity [Levey et al. 2007]).
21. Must be able to attend trial visits as required by the protocol.
22. Women of childbearing potential (WOCBP) must have a negative serum (beta-human chorionic gonadotropin [ß-hCG]) test/value at screening (see SoA). Patients who are post-menopausal or permanently sterilized can be considered as not having reproductive potential.
23. WOCBP must agree not to donate eggs (ova, oocytes) for the purposes of assisted reproduction during the entire trial and thereafter.

24. WOCBP and men that are sexually active with a WOCBP and have not had a vasectomy must agree to use highly effective birth control method(s), such as barrier method of birth control, e.g., either condom with spermicidal foam/gel/film/cream/suppository for men; and/or for females occlusive cap (diaphragm or cervical/vault caps) with spermicidal foam/gel/film/cream/suppository; an intrauterine device; and/or hormone-based contraception, with established use of oral, injected, or implanted hormonal methods of contraception. True abstinence is an acceptable alternative to the use of contraception.
25. Men must agree not to father a child or donate sperm, and WOCBP must agree not to become pregnant during the trial and for at least 12 months after the CLDN6 CAR-T/CLDN6 CAR-T(A) infusion or CLDN6 RNA-LPX treatment.

5.2 EXCLUSION CRITERIA

A patient who meets any of the following criteria will be excluded from participating in the trial.

- Prior/concomitant therapy

1. Has received prior CAR-T therapy, except CLDN6 CAR-T/CLDN6 CAR-T(A) therapy.
2. Has received vaccination with live virus vaccines within 6 weeks prior to the start of LD.
3. Receives concurrent systemic (oral or i.v.) steroid therapy > 10 mg prednisolone daily, or its equivalent, for an underlying condition.
4. Has side effects of any prior therapy or procedures for any medical condition not recovered to NCI CTCAE v.5 Grade ≤ 1.

Notes: Peripheral neuropathy Grade ≤ 2 is allowed; alopecia of any grade is allowed.

- Medical conditions

5. Current evidence of new or growing brain or spinal metastases during screening. Patients with known brain or spinal metastases may be eligible if they:
    a. Have had radiotherapy or another appropriate therapy for the brain or spinal metastases,
    b. Have no neurological symptoms,
    c. Have stable brain or spinal disease on the computer tomography (CT) or magnetic resonance imaging (MRI) scan within 4 weeks before signing of the ICF,
    d. Are not undergoing acute corticosteroid therapy or steroid taper. Chronic steroid therapy is acceptable provided that the dose is stable for the last 14 d prior to screening (≤ 10 mg prednisolone daily or equivalent),
    e. Do not require steroid therapy within 7 d before the first dose of CLDN6 CAR-T/CLDN6 CAR-T(A),
    f. Have anticipated imminent fracture or cord compression due to spinal bone metastases.

Notes: Patients with central nervous system symptoms suggestive for CNS metastasis should undergo a CT scan or MRI of the brain to exclude new or progressive brain metastases.

6. Has history of epilepsy. Isolated seizures in the past or febrile seizures in childhood are permitted; has a history of a cerebrovascular accident or transient ischemic attack less than 6 months ago.
7. Pericardial effusion requiring any drainage is excluded.
8. Has an active autoimmune disease including but not limited to inflammatory bowel disease, systemic lupus erythematosus, ankylosing spondylitis, scleroderma, or multiple sclerosis. Has any active immunologic disorder requiring immunosuppression with steroids or other immunosuppressive agents (e.g., azathioprine, cyclosporine A) with the exception of patients with isolated vitiligo, resolved childhood asthma or atopic dermatitis, controlled hypoadrenalism or hypopituitarism, and euthyroid patients with a history of Grave's disease. Patients with controlled hyperthyroidism must be negative for thyroglobulin, thyroid peroxidase antibodies, and thyroid-stimulating immunoglobulin prior to trial drug administration.
9. Seropositivity for human immunodeficiency virus (HIV).
10. Known history/positive serology for hepatitis B requiring active antiviral therapy (unless immune due to vaccination or resolved natural infection or unless passive immunization due to immunoglobulin therapy). Patients with positive serology must have hepatitis B virus viral load below the limit of quantification.
11. Active Hepatitis C virus (HCV) infection; patients who have completed curative antiviral treatment with HCV viral load below the limit of quantification are allowed.
12. Has a known hypersensitivity to a component of CLDN6 CAR-T/CLDN6 CAR-T(A) or CLDN6 RNA-LPX cancer vaccine drug product, or another similar compound.
13. Only for patients recruited for Part 2 with LD chemotherapy (CLDN6 CAR-T/CLDN6 CAR-T(A) + CLDN6 RNA-LPX with LD chemotherapy): history of severe immediate hypersensitivity reaction to LD chemotherapy consisting of cyclophosphamide or fludarabine.
14. Has a history of another primary cancer within the 2 years prior to enrollment except for the following: Non-melanoma skin cancer, cervical carcinoma in situ, superficial bladder cancer, prostate cancer with currently undetectable prostate specific antigen, or other non-metastatic carcinoma that has been in complete remission without treatment for more than 2 years.
15. Receipt of allogenic stem cell transplantation in the 5 years prior to enrollment into the trial.
16. Patients with acute or chronic graft versus host disease.

- Other comorbidities

17. Has abnormal electrocardiograms (ECGs) that are clinically significant, such as QT prolongation.
18. In the opinion of the investigator, has any concurrent conditions that could pose an undue medical hazard or interfere with the interpretation of the trial results; these conditions include, but are not limited to:
    a. Ongoing or active infection requiring antibiotic/antiviral/antifungal therapy
    b. Concurrent congestive heart failure (New York Heart Association Functional Classification Class III or IV)
    c. Concurrent unstable angina
    d. Concurrent cardiac arrhythmia requiring treatment (excluding asymptomatic atrial fibrillation)
    e. Acute coronary syndrome within the previous 6 months
    f. Significant pulmonary disease (shortness of breath at rest or on mild exertion) for example due concurrent severe obstructive pulmonary disease.
19. Has a cognitive, psychological or psychosocial impediment that would impair the ability of the patient to receive therapy according to the protocol or adversely affect the ability of the patient to comply with the informed consent process, protocol, or protocol-required visits and procedures.
20. Is pregnant or breastfeeding.

5.3 PRE-SCREENING

Inclusion criterion 1 (CLDN6 expression) will be assessed prior to any other screening assessments. Patients will sign a first ICF specifically for this assessment. It is expected that archival FFPE blocks will be available for the majority of patients and thus no fresh biopsy will be required. However, in the rare circumstances where such fresh biopsy is needed, the investigator must justify such surgical procedure for any cancer type with an expected probability of CLDN6 expression below 30%. In such cases, the biopsy can only be performed if the risk of the procedure is considered to be minimal. The medical monitor of the sponsor must be consulted prior to taking any biopsies with a probability of CLDN6 expression below 30%. Full eligibility will be assessed only when Inclusion Criterion 1 has been confirmed and the patient has signed the ICF for the trial.

5.4 SCREEN FAILURES

Screen failures are defined as patients who consent to participate in the clinical trial with regard to the protocol's inclusion and exclusion criteria. Patients who sign an ICF but do not meet eligibility criteria are defined as screen failures.

Once a patient meets all the inclusion criteria and none of the exclusion criteria during the pre-screening period, the patient will undergo apheresis, initiating the study drug manufacturing process. Once the patient successfully completes the leukapheresis process,

and continues to meet all the inclusion criteria and none of the exclusion criteria, she/he is considered enrolled.

Patients will be evaluated for their ability to tolerate the leukapheresis procedure during the pre-leukapheresis assessment. Venous access is evaluated to determine if, due to inadequate veins, placement of a central venous catheter is absolutely necessary to undergo leukapheresis. In the event that a patient's leukapheresis procedure fails, or the infusion is not possible for any other reason, the patient may be scheduled to undergo a repeat leukapheresis procedure.

A minimal set of screen failure information is required to ensure transparent reporting of screen failure patients to meet the Consolidated Standards of Reporting Trials publishing requirements and to respond to queries from regulatory authorities. Minimal information includes demographic information, informed consent, screen failure details, eligibility criteria, and any SAE. In addition, it will be attempted to receive information on the subsequent treatment and its outcome.

Patients who do not meet the criteria for participation in this trial (screen failure) may qualify for two re-screening opportunities (for a total of three screenings per patient) at the investigator's discretion. Patients must re-sign the ICF prior to re-screening.

6 TRIAL TREATMENTS

6.1 TRIAL TREATMENTS ADMINISTERED

6.1.1 Lymphodepleting chemotherapy regimen

Upon notification from the sponsor that CLDN6 CAR-T/CLDN6 CAR-T(A) will be available, LD chemotherapy should be completed at least 3 d prior to the CLDN6 CAR-T/CLDN6 CAR-T(A) infusion. The lymphodepleting chemotherapy will consist of:

- Cyclophosphamide: 500 mg/m^2/d infused i.v. according to institutional standard of care on d-5, d-4, and d-3,
  and
- Fludarabine: 30 mg/m^2/d infused i.v. according to institutional standard of care on d-5, d-4, and d-3. In case of moderate impairment of renal function, the dose should be reduced to 50% and the patient's renal function should be monitored daily.

The lymphodepleting chemotherapy may be lowered based on emerging safety data and decisions about lowering the dose will be made by the SRC.

- Cyclophosphamide: 250 mg/m^2/d infused i.v. according to institutional standard on d-5, d-4, and d-3,
  and

- Fludarabine: 25 mg/m^2/d infused i.v. according to institutional standard on d-5, d-4, and d-3. In case of moderate impairment of renal function, the dose should be reduced to 50% and the patient's renal function should be monitored daily.

The lymphodepleting chemotherapy will only be initiated if the following criteria apply (Refer to Section 2.3.3):

- No evidence of uncontrolled infection,
- No clinically significant cardiac dysfunction,
- No acute neurological toxicity > Grade 1 (with the exception of peripheral sensory neuropathy),
  and
- Adequate renal function (GFR > 30 mL/min/1.73 m²) – according to the abbreviated Modification of Diet in Renal Disease equation:

  $$GFR = 175 \times (\text{standardized SCr}^{-1.154}) \times (\text{age}^{-0.203})$$

  (where SCr, the serum creatinine level, is expressed in mg/dL; multiply it by 0.742 if the patient is female; multiply it by 1.212 if the patient is of black ethnicity [Levey et al. 2007]).

Should any of the criteria not be met, the lymphodepleting chemotherapy will be delayed until this criterion resolves to baseline of Grade 1.

The 3-day LD chemotherapy is not investigational and may be administered as an outpatient regimen by a patient's local oncologist within the specified time frame depending upon the need for i.v. fluids.

Patients must be evaluated as above mentioned for evidence of ongoing infections prior to the LD chemotherapy to be started. In case of suspicion of infection, the patient should be treated accordingly and LD chemotherapy postponed until infection resolution. In case of a prolonged delay in administration of more than 5 d based on the planned start of chemotherapy, the investigator should discuss the next course of action with the sponsor.

6.1.2 IMP-1 and IMP-1(A): CLDN6 CAR-T/CLDN6 CAR-T(A)

CLDN6 CAR-T/CLDN6 CAR-T(A) are autologous T cells that have been engineered to express a CAR composed of an extracellular scFv with specificity for CLDN6, linked via a CD8 hinge domain to intracellular 4-1BB and CD3ζ signaling domains. CLDN6 CAR-T/CLDN6 CAR-T(A) are cryopreserved in infusible cryomedia and will be administered as a single infusion. Each bag will contain an aliquot (volume dependent upon dose) of cryomedia

containing the following infusible grade reagents (% v/v): 50% CryoStor CS10, 50% sodium chloride (0.9%). The total amount of dimethyl sulfoxide is 5%.

Flat dosing is planned for this trial. The selected starting dose for CLDN6 CAR-T (IMP-1) is 10^7 CLDN6 CAR-T, followed by two additional dose levels of 10^8 and 10^9 CLDN6 CAR-T. The selected starting dose for CLDN6 CAR-T(A) (IMP-1(A)) is 10^6 CLDN6 CAR-T followed by three additional dose levels of 10^7, 10^8, and $2x10^8$ to $5x10^8$ CLDN6 CAR-T(A).

The clinical observation after the infusion is described in Section 6.6.5.

6.1.2.1 CLDN6 CAR-T/CLDN6 CAR-T(A) pre-medication

The risk of systemic reactions during or after CLDN6 CAR-T/CLDN6 CAR-T(A) infusion is unknown. Patients should be monitored closely for signs and symptoms including, but not limited to, fever, chills, rigor, headache, and myalgia, or, in more severe cases, hypotension, tachycardia, dyspnea/hypoxia or chest discomfort, wheezing, angioedema, urticaria, and rash.

Prophylactic treatment with antipyretics (e.g., acetaminophen 650 to 1000 mg, ibuprofen 400 mg, or per institutional standard) and antihistamines (e.g., clemastine 0.03 mg/kg i.v. [2 mg max] or ranitidine 50 mg i.v.) is required approximately 0.5 to 1 h prior to IMP infusion in patients who receive CLDN6 CAR-T/CLDN6 CAR-T(A). Non-pharmacologic measures (e.g., warming blankets, forced air patient warming system, etc.) should be considered and be provided after CLDN6 CAR-T/CLDN6 CAR-T(A) infusion and even prior to onset of signs and symptoms of systemic reaction to mitigate the occurrence of chills and rigors.

6.1.2.2 CLDN6 CAR-T/CLDN6 CAR-T(A) administration and duration of treatment

The infusion of CLDN6 CAR-T/CLDN6 CAR-T(A) takes place only once on d1. CLDN6 CAR-T/CLDN6 CAR-T(A) should be given on a Monday or Tuesday in order to comply with the SoA.

The CAR-T dose will be administered by i.v. infusion. The duration of the infusion will be based on the total volume to be infused and the recommended infusion rate.

Patients will be treated if they meet the following criteria:

- No new medical condition that would unduly increase the risk of infection
- No new requirement for oxygen supplementation > 2L via nasal cannula to keep saturation above 95%
- No uncontrolled cardiac arrhythmias
- No hypotension requiring pressor support
- No uncontrolled active infections

- No fever above 38°C within 24 h prior to CLDN6 CAR-T/CLDN6 CAR-T(A) administration
- For female patients with reproductive potential: repeated negative pregnancy test (within 5 d prior to CLDN6 CAR-T/CLDN6 CAR-T(A) administration)

Should any of these criteria not be met, the administration of CLDN6 CAR-T/CLDN6 CAR-T(A) will be delayed until resolved at least to Grade 1. Note that CLDN6 CAR-T/CLDN6 CAR-T(A) infusions should be administered on Mondays and Tuesdays in order to comply with the SoA.

Patients with active infection must have CLDN6 CAR-T/CLDN6 CAR-T(A) infusion postponed until the active infection has resolved (patients with suspected/active infection must have negative culture for at least 24 h on appropriate antibiotics or negative rapid viral panel). Patients with organ toxicities may not receive CLDN6 CAR-T/CLDN6 CAR-T(A) until the organ toxicities have recovered to ≤ Grade 2. In case of delayed infusion, LD chemotherapy may need to be repeated after discussion with the sponsor.

6.1.2.3 Treatment with a non-conformant CLDN6 CAR-T/CLDN6 CAR-T(A) product

In case the CLDN6 CAR-T/CLDN6 CAR-T(A) product does not meet all specifications, it can be used under certain conditions and such use must be approved by the sponsor's medical director or equally qualified person. The patient must sign a separate consent form prior to use of the non-conformant product. The cases where such use can be discussed include one of the below, but not a combination of any:

- Viability of CLDN6 CAR-T/CLDN6 CAR-T(A) prior to freezing between 50% and 69.9%.
- Cell dose for a given dose level not reached, but at least $1x10^6$ CLDN6 CAR-T/CLDN6 CAR-T(A) are available.
- Transduction efficacy between 3.00% and 9.99%

Since the CLDN6 CAR-T/CLDN6 CAR-T(A) have the ability to proliferate in the human body even at low numbers, the patient may benefit from the treatment, if one of the above mentioned criteria is met. The risks are considered comparable to a product meeting all specifications. In any case it must be ensured that the lymphodepleting chemotherapy is not started before the use of the non-conformant product has been approved and the patient has consented to it. The possibility of a repeated leukapheresis and production of the CLDN6 CAR-T/CLDN6 CAR-T(A) must also be taken into consideration.

Any other cases not mentioned above must be brought forward to the SRC, but will be approved only under exceptional circumstances where the benefit for the patient can be duly justified.

Patients treated with non-conformant CLDN6 CAR-T products will be replaced and will not be considered for efficacy analysis. The safety data will be discussed with the SCR to obtain a full picture of the safety profile of the IMP, however will not be considered for dose decision meetings. Data of such patients will be clearly marked as "out of specification" for the discussions in the safety calls. In the study report, such data will be also clearly flagged in the safety set and separated from the per protocol data set. Patients will undergo all assessment as described in this clinical trial protocol and will also participate in the long-term follow up.

6.1.3 IMP-2: CLDN6 RNA-LPX

The IMP-2 is CLDN6 RNA-LPX vaccine, which is liposomally formulated mRNA to systemically deliver CLDN6 encoding mRNA to DCs in secondary lymphoid tissues.

The CLDN6 RNA-LPX will be injected as a bolus into a peripheral vein using a peripheral venous catheter. Prior to and after the injection of the RNA-LPX product, 5 mL of isotonic sodium chloride solution (0.9%) should be administered (flushing). The RNA-LPX injection to patients should be performed as slowly as possible or over a time period of 2 min.

The proposed starting dose of CLDN6 RNA-LPX cancer vaccine is 25 µg on d4 in the standard Part 2 and the optional Part 2 LD-free cohorts and on d24 in the optional Part 2 cohort with LD. A step-up dose of 50 µg is administered on d30 in the standard Part 2 and the optional Part 2 LD-free cohort and on d36 in the optional Part 2 cohort with LD. The same dose is administered in subsequent administrations if it is tolerated. If the starting dose of 25 µg is not tolerated, a dose of 12.5 µg will be explored. If 12.5 µg is tolerated, this TD will be administered on the remaining dosing days: d30 or d36 d51, d72, d93, and then every 6 weeks until m22.

The clinical observation after the injection is described in Section 6.6.5.

6.1.3.1 CLDN6 RNA-LPX pre-medication

Patients may receive hydration with isotonic i.v. fluid (500 to 1000 mL, per institutional standard) to ensure patients remain euvolemic on days that CLDN6 RNA-LPX is administered.

To address flu-like symptomatology which might occur frequently on the day of CLDN6 RNA-LPX administration, patients should be pre-medicated with standard therapeutic doses of acetaminophen, or non-steroidal anti-inflammatory drugs (NSAIDs) at least 60 min before CLDN6 RNA-LPX injection. The drugs should be administered again following CLDN6 RNA-LPX administration within recommended interval up to daily maximum total dose – refer to Prescribing Information.

Corticosteroids should be avoided as either prophylaxis or treatment as they counteract the effects of CLDN6 RNA-LPX. Non-pharmacologic measures (e.g., warming blankets, forced

air patient warming system, etc.) should be considered and be provided after CLDN6 RNA-LPX injection and even prior to onset of signs and symptoms of systemic reaction to mitigate the occurrence of chills and rigors.

6.1.3.2 CLDN6 RNA-LPX administration and duration of treatment

The administration of CLDN6 RNA-LPX takes place 3 d after CAR-T therapy infusion on d4 in the standard Part 2 cohort and the optional Part 2 LD-free cohort and on d24 in the optional Part 2 cohort with LD, and is continued on d30 or d36, d51, d72, d93, and then every 6 weeks until m22.

Patients from Part 1 who will be allowed to receive the CLDN6 RNA-LPX vaccine after the dose level has been deemed safe by the SRC, will follow the same treatment schedule and undergo the same procedures as patients in Part 2. Since the exact starting time after the initial CAR-T-infusion is unknown, the day of first CLDN6 RNA-LPX administration will be called dx+d4, where dx is the study day of the cross-over. The CLDN6 RNA-LPX treatment should start on a Thursday or Friday to allow compliance with the SoA.

The administration of CLDN6 RNA-LPX will be delayed in cases where the patient shows signs and symptoms of CLDN6 CAR-T/CLDN6 CAR-T(A) toxicity, such as CRS, immune effector cell-associated neurotoxicity syndrome (ICANS), macrophage activation syndrome (MAS), fever, or autoimmune reactions, adverse reactions to excipients, infusion reactions such as fever, rigors, rash, urticaria, dyspnea, hypotension, and/or nausea. It should be delayed until the symptoms have resolved. The delayed administration of CLDN6 RNA-LPX should be performed on a Tuesday or Wednesday in order to comply with the SoA.

Dose modifications and guidance on step-up dosing are detailed in Section 6.6.

6.1.3.2.1 Re-dosing and treatment beyond progression

In all parts of the trial, treatment should be discontinued in all patients who exhibit evidence of PD by RECIST 1.1. However, to account for pseudo-progression seen in patients treated with cancer immunotherapies and to better accommodate standard clinical practice which is guided by the fact that these patients have, in general, limited treatment options and such options have limited efficacy and significant toxicity, patients may be considered for treatment beyond progression at the discretion of the investigator, either re-dosing with CLDN6 CAR-T/CLDN6 CAR-T(A) or continuous treatment with the CLDN6 RNA-LPX vaccine. This is only feasible after appropriate discussion with the patient and obtaining informed consent, only if all of the following criteria are met:

- Absence of clinical symptoms or signs indicating clinically significant disease progression.
- No decline in performance status.
- Absence of rapid disease progression or threat to vital organs or critical anatomical sites (e.g., central nervous system metastasis, respiratory failure due to tumor

compression, spinal cord compression) requiring urgent alternative medical intervention.

- No significant, unacceptable or irreversible toxicities related to trial treatment.
- Patients must provide written consent to acknowledge deferring alternative treatment options including other clinical trials in favor of re-dosing with CLDN6 CAR-T/CLDN6 CAR-T(A) or continuing trial treatment at the time of initial progression.
- Patients must meet the inclusion criteria for hematologic, renal, hepatic and coagulation functions.

Patients who undergo re-dosing or continue treatment beyond radiographic disease progression per RECIST 1.1 should be closely monitored clinically and with a follow-up scan in 6 weeks or sooner if symptomatic deterioration occurs. Treatment should be discontinued if clinical deterioration due to disease progression occurs at any time, or if persistent disease growth is confirmed in a follow-up scan. In addition, patients should be discontinued for unacceptable toxicity or for any other signs or symptoms of deterioration attributed to disease progression as determined by the investigator after an integrated assessment of radiographic data and clinical status.

Patients who discontinue treatment for reasons other than radiographic disease progression (e.g., toxicity) will continue scheduled tumor assessments at the same frequency as would have been followed if the patient had remained on trial treatment.

6.2 PREPARATION/HANDLING/STORAGE/ACCOUNTABILITY

Full details are provided in the Pharmacy Manual.

1) The investigator or designee must confirm appropriate temperature conditions have been maintained during transit for all trial treatment received and any discrepancies are reported and resolved before use of the trial treatment.
2) Only patients enrolled in the trial may receive trial treatment and only authorized site staff may supply or administer trial treatment. All trial treatment must be stored in a secure, environmentally controlled, and monitored (manual or automated) area in accordance with the labeled storage conditions with access limited to the investigator and authorized site staff.
3) The investigator, institution, or the head of the medical institution (where applicable) is responsible for trial treatment accountability, reconciliation, and record maintenance (i.e., receipt, reconciliation, and final disposition records).

6.3 MEASURES TO MINIMIZE BIAS: RANDOMIZATION AND BLINDING

Open-label, no blinding at site level

This is an open-label trial; potential bias will be reduced by assigning patients to treatment groups consecutively if one group is open and in alternating groups if more than one group is open.

6.4 TRIAL TREATMENT COMPLIANCE

Patients will be dosed at the site, and will receive trial treatment directly from the investigator or designee, under medical supervision. The date and time of each dose administered in the clinic will be recorded in the source documents and recorded in the electronic case report form (eCRF). The dose of trial treatment and trial patient identification will be confirmed at the time of dosing by a member of the trial site staff other than the person administering the trial treatment.

6.5 CONCOMITANT THERAPY

Any medication, vaccine (including over-the-counter or prescription medicines, vitamins, and/or herbal supplements or other specific categories of interest), or non-drug therapies that the patient is receiving at the time of enrollment (leukapheresis) or receives during the trial must be recorded along with:

- Reason for use
- Dates of administration including start and end dates
- Dosage information including dose and frequency

The medical monitor should be contacted if there are any questions regarding concomitant or prior therapy.

Documentation of concomitant therapies will end 90 d after last IMP treatment. Thereafter only medications administered for treatment of IMP-related AEs will be documented.

6.5.1 Prohibited medication

The following treatments are not permitted during the trial and within the indicated time period:

- Allogenic stem cell transplantation in the 5 years prior to enrollment into the trial.
- Live vaccines: within 4 weeks prior to enrollment and 6 months after the CLDN6 CAR-T/CLDN6 CAR-T(A) infusion.
- GM-CSF should be avoided due to its potential to worsen CRS.

6.5.2 Permitted medication

The following treatments are permitted during the trial, but have to be discontinued prior to leukapheresis within the indicated time period:

- Antiproliferative therapies (e.g. chemotherapy) other than the lymphodepleting chemotherapy: 14 d prior to leukapheresis.
  - As an exception, short-acting drugs such as tyrosine kinase inhibitors: 72 h prior to leukapheresis.
- Checkpoint inhibitors: 14 d prior to leukapheresis.
- Immunosuppressants other than steroids: 14 d prior to leukapheresis.
- Corticosteroids (e.g. prednisone ≥ 10 mg/d or equivalent doses of other corticosteroids): 72 h prior to leukapheresis.
  - As an exception, topical and inhaled corticosteroids in standard doses and physiologic replacement for patients with adrenal insufficiency are allowed.

The following treatments are permitted after leukapheresis, but have to be discontinued prior to CLDN6 CAR-T/CLDN6 CAR-T(A) infusion:

- Antiproliferative therapies (e.g. chemotherapy) other than the lymphodepleting chemotherapy: 14 d prior to CLDN6 CAR-T/CLDN6 CAR-T(A) infusion.
  - As an exception, short-acting drugs such as tyrosine kinase inhibitors: 72 h prior to CLDN6 CAR-T/CLDN6 CAR-T(A) infusion.
- Antibodies other than checkpoint inhibitors: 4 weeks prior to CLDN6 CAR-T/CLDN6 CAR-T(A) infusion.
- Checkpoint inhibitors: 14 d prior to CLDN6 CAR-T/CLDN6 CAR-T(A) infusion.
- Immunosuppressants other than steroids: 14 d prior to CLDN6 CAR-T/CLDN6 CAR-T(A) infusion.
- Corticosteroids (e.g. prednisone ≥ 10 mg/d or equivalent doses of other corticosteroids): 72 h prior to CLDN6 CAR-T/CLDN6 CAR-T(A) infusion.
  - As an exception, topical and inhaled corticosteroids in standard doses and physiologic replacement for patients with adrenal insufficiency are allowed.

6.5.3 Rescue medication and further supportive measures

At least 4 doses of tocilizumab have to be available on site for the treatment of CRS.

Patients should receive antibiotics, anti-viral and anti-fungal prophylaxis, red blood and platelet cell transfusions, as well as G-CSF according to local supportive care guidelines and agreement with the sponsor. Patients with a history of HDCT/ASCT should have a back-up stem cell boost available.

6.6 DOSE MODIFICATIONS

6.6.1 Dose modification for CLDN6 CAR-T/CLDN6 CAR-T(A)

No dose modification of CLDN6 CAR-T/CLDN6 CAR-T(A) is allowed. Patients should receive the full infusion to be DLT-evaluable. During dose escalation, lower number of produced CLDN6 CAR-T/CLDN6 CAR-T(A) than the intended dose may be administered, the patient will be replaced for DLT evaluation and will be followed up per trial procedure. During Part 3, lower numbers of produced CLDN6 CAR-T/CLDN6 CAR-T(A) than the intended dose may be administered, and the patient will be followed up per trial procedure.

6.6.2 Dose modification for CLDN6 RNA-LPX

Dose modifications may become necessary in case of occurrence of CLDN6 RNA-LPX-related AEs. No dose reduction of CLDN6 RNA-LPX below 12.5 µg is allowed.

AEs that fulfill the DLT criteria both within the DLT period and outside the DLT period in the dose escalation part of the trial or during the expansion should be handled as shown in Figure 12.

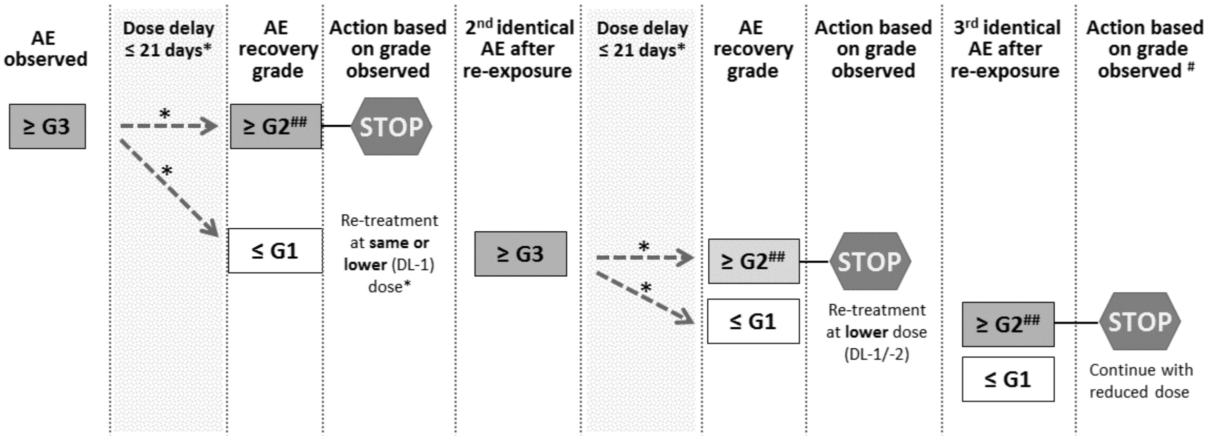

Figure 12: Handling of adverse events fulfilling the DLT criteria

Abbreviations: AE, adverse event; DL, Dose level, G, grade.
*Dose delay: Next dose of CLDN6 RNA-LPX can maximally be delayed 21 d unless approved otherwise by the Sponsor's Medical Monitor.
No delay for recovery from ≥ Grade 2 allowed
Unless grade at baseline was ≥ Grade 2 and AE has resolved to baseline grade

Note: Grade 4 AEs fulfilling DLT criteria will always lead to CLDN6 RNA-LPX discontinuation.

First occurrence of AE fulfilling DLT criteria Grade 3:

- As a first measure, administration of CLDN6 RNA-LPX needs to be held.

- Investigator must contact the sponsor for thorough discussion in order to decide whether the patient should be withdrawn from CLDN6 RNA-LPX treatment or next dosing should be delayed.
- Administration of CLDN6 RNA-LPX can be delayed for up to 21 d unless otherwise approved by the Sponsor's Medical Monitor. If the intensity resolves to ≤ Grade 1 or baseline within this period, re-treatment may be considered under the following:
    - Sponsor and investigator will discuss any safety concerns in order to decide whether the next dose of CLDN6 RNA-LPX should be administered at same dose level or one dose level lower (DL-1); not applicable if the TD is 12.5 µg. The sponsor may also consult the SRC.

<u>Second occurrence of an identical AE Grade 3 after re-exposure to CLDN6 RNA-LPX:</u>

- As a first measure, administration of CLDN6 RNA-LPX needs to be held.
- If re-treatment leads to an identical AE with same intensity, the next administration of CLDN6 RNA-LPX can be delayed for up to 21 d unless otherwise approved by the Sponsor's Medical Monitor. If the intensity of the AE resolves to ≤ Grade 1 or baseline within this period, re-treatment may be considered under the following condition:
    - The next dose of CLDN6 RNA-LPX should be administered at one dose level lower (DL-1 or DL-2) than the dose level causing the recurrence of the AE; not applicable if the TD is 12.5 µg.

<u>Third occurrence of an identical AE ≥ Grade 2 after re-exposure to CLDN6 RNA-LPX:</u>

- As a first measure, administration of CLDN6 RNA-LPX needs to be held.
- If re-treatment at a lower dose leads to a third identical AE with intensity ≥ Grade 2, the patient must permanently discontinue IMP. No dose delay is allowed. However, if the AE is ≤ Grade 1 or baseline, re-treatment may be considered under the following condition:
    - Next dose of CLDN6 RNA-LPX should be administered at same reduced dose level (DL-1 or DL-2); not applicable if the TD is 12.5 µg.

<u>Please note:</u>

- Re-escalation of CLDN6 RNA-LPX dose is not allowed for patients that have previously been dose reduced.
- CLDN6 RNA-LPX must be permanently discontinued if the patient experiences an AE fulfilling the DLT criteria (after the DLT period has ended for the dose escalation or during the expansion) that fails to resolve to ≤ Grade 1 within 21 d after the planned dosing date unless otherwise approved by the Sponsor's Medical Monitor.
- CLDN6 RNA-LPX must be permanently discontinued if more than two dose reductions are required.

- CLDN6 RNA-LPX must be permanently discontinued in case of a dose delay of more than 21 d due to toxicity possibly related to CLDN6 RNA-LPX unless otherwise approved by the Sponsor's Medical Monitor.
- The investigators are encouraged to contact the sponsor in case of any safety concern that need thorough discussion and evaluation.

6.6.3 Mitigation plans for specific adverse events

The potential toxicities related to CLDN6 CAR-T/CLDN6 CAR-T(A) therapy or the addition of CLDN6 RNA-LPX should be approached with sound clinical judgment. As general mitigation measures, the following are ensured in this trial:

1. Only investigators and sites with cell therapy experience are selected to participate in the trial.
2. Mitigation plans for specific AEs are outlined in this trial protocol.
3. Site personnel are properly trained at the trial start-up and on an ongoing basis throughout the entire trial.
4. At least four doses of tocilizumab are available on site prior to CLDN6 CAR-T/CLDN6 CAR-T(A) administration.
5. Any dose/dosage adjustment should be overseen by medically qualified trial site personnel (preferably the Principal or Sub-investigator) unless an immediate safety risk appears to be present.
6. Access to an intensive care unit (ICU) is ensured at every site participating in the trial.
7. SRC reviews and endorses all safety-related decisions.
8. The risk to patients in this trial may be minimized by compliance with the eligibility criteria, pre-infusion criteria, CRS treatment algorithm, trial procedures, and close clinical monitoring.

The potential specific AEs listed here may not cover all potential toxicities related to CLDN6 CAR-T/CLDN6 CAR-T(A) with or without CLDN6 RNA-LPX. The investigators are encouraged to be vigilant of the potential toxicities and to work closely with the sponsor to identify all concerning safety issues. Reference to the corresponding IB and literature where appropriate is highly recommended.

6.6.4 Clinical observation of patients receiving IMP-1, IMP-1(A), or IMP-2

Patients will be monitored closely during their in-patient stay for early signs of toxicities. Since administration of CLDN6 RNA-LPX can lead to enhanced expansion and activation of CLDN6 CAR-T/CLDN6 CAR-T(A), toxicities related to CLDN6 CAR-T/CLDN6 CAR-T(A) can occur after any administration of CLDN6 RNA-LPX.

Physical examinations, including the immune effector cell-associated encephalopathy (ICE) score assessment, heart rate, blood pressure and temperature will be carried out daily during the hospitalization periods. Menstrual status should be checked at d1, d28 and at all subsequent visits. Any suspicion of CRS or ICANS will trigger the actions recommended below. In addition, blood tests as detailed in Section 12.2 will be performed daily until resolution of symptoms.

Patients will be alerted to report any signs and symptoms of toxicities immediately to their investigator, also after their discharge from the hospital. They should measure their temperature while at home daily for the first two weeks post IMP-1, IMP-1(A), or IMP-2 administration.

6.6.4.1 Cytokine release syndrome

The most common acute toxicity of CAR-T is CRS. The cytokines implicated in CRS may be directly produced by the infused CAR-T, or other immune cells such as macrophages that might produce cytokines in response to cytokines produced by the infused CAR-T. A wide variety of cytokines including interleukin (IL)-6, interferon-γ (IFN-γ), tumor necrosis factor (TNF), IL-2, IL-2–receptor-α, IL-8, and IL-10 are elevated in the serum of patients experiencing fever, tachycardia, hypotension, and other toxicities after CAR-T infusions.

For assessment of CRS, the grading criteria published by the American Society for Transplantation and Cellular Therapy (ASTCT) will be applied (Lee et al. 2019). This should ensure consistent grading throughout the trial and among all participating sites.

Table 17: American Society for Transplantation and Cellular Therapy cytokine release syndrome consensus grading

CRS parameter	Grade 1	Grade 2	Grade 3	Grade 4
**Fever**[1]	Temperature ≥ 38°C	Temperature ≥ 38°C	Temperature ≥ 38°C	Temperature ≥ 38°C
with				
**Hypotension**	None	Not requiring vasopressors	Requiring vasopressor with or without vasopressin	Requiring multiple vasopressors (excluding vasopressin)
and/or[2]				
**Hypoxia**	None	Requiring low-flow nasal cannula[3] or blow-by	Requiring high-flow nasal cannula, facemask, nonrebreather mask, or Venturi mask	Requiring positive pressure (e.g., CPAP, BiPAP, intubation and mechanical ventilation)

Abbreviations: BiPAP, Bilevel positive airway pressure; CPAP, Continuous positive airway pressure; CRS, Cytokine release syndrome; CTCAE, Common terminology criteria for adverse events.

1) Fever is defined as temperature ≥ 38 C not attributable to any other cause. In patients who have CRS then receive antipyretic or anti-cytokine therapy such as tocilizumab or steroids, fever is no longer required to grade subsequent CRS severity. In this case, CRS grading is driven by hypotension and/or hypoxia.
2) CRS grade is determined by the more severe event: Hypotension or hypoxia not attributable to any other cause. For example, a patient with temperature of 39.5 C, hypotension requiring one vasopressor, and hypoxia requiring low-flow nasal cannula is classified as Grade 3 CRS.
3) Low-flow nasal cannula is defined as oxygen delivered at ≤ 6 L/minute. Low-flow also includes blow-by oxygen delivery, sometimes used in pediatrics. High-flow nasal cannula is defined as oxygen delivered at > 6 L/minute.

Organ toxicities associated with CRS may be graded according to CTCAE v5.0 but they do not influence CRS grading.

If CRS is suspected, the following parameters should be assessed daily by the central laboratory: IL-6, C-reactive protein, TNF-α, ferritin, fibrinogen, procalcitonin, clinical chemistry, and hematology (Refer to Section 12.2).

For the management of CRS, the following guidelines should apply:

Table 18: Guidelines for management of cytokine release syndrome

ASBMT CRS Grade	Symptom or sign	Management
Grade 1	Fever with temperature ≥ 38ºC but no hypotension or hypoxia	• Antipyretics and i.v. hydration<br>• Diagnostic work-up to rule out infection<br>• Consider growth factors and antibiotics if neutropenic
Grade 2	Fever with hypotension not requiring vasopressors and/or hypoxia requiring low-flow nasal cannula	• Supportive care as in Grade 1<br>• i.v. fluid boluses and/or supplemental oxygen<br>• Tocilizumab +/- dexamethasone or its equivalent of methylprednisolone
Grade 3	Fever with hypotension requiring one vasopressor with or without vasopressin and/or hypoxia requiring high-flow nasal cannula, facemask, non-rebreather mask, or venture mask	• Supportive care as in Grade 1<br>• Consider monitoring in ICU<br>• Vasopressor support and/or supplemental oxygen<br>• Tocilizumab + dexamethasone 10-20 mg i.v. q6h or its equivalent of methylprednisolone
Grade 4	Fever with hypotension requiring multiple vasopressors (excluding vasopressin) and/or hypoxia requiring positive pressure (e.g., CPAP, BiPAP, intubation and mechanical ventilation)	• Supportive care as in Grade 1<br>• Monitoring in ICU<br>• Vasopressor support and/or supplemental oxygen via positive pressure ventilation<br>• Tocilizumab + methylprednisolone 1000 mg/d

Abbreviations: ASBMT, American Society for Bone Marrow Transplant; BiPAP, Bilevel positive airway pressure; CPAP, Continuous positive airway pressure; CRS, Cytokine release syndrome; ICU, Intensive care unit; i.v., Intravenous.
Source: Neelapu 2019.

6.6.4.2 Transaminitis and elevated pancreatic enzymes

As of September 16th, asymptomatic increases in transaminases (but not total bilirubin) and lipase were observed in patients with CRS or with pre-existing hepatic metastases.

Asymptomatic Grade 3 increases in ALT, AST, and lipase should be observed closely without further interventions.

In case of symptomatic (jaundice, abdominal pain) Grade 3 and Grade 4 increases, treatment with steroids (see Grade 3 CRS, Table 18) should be considered.

6.6.4.3 Immune effector cell-associated neurotoxicity syndrome

Neurologic toxicities have been reported with CAR-T therapy in which serum cytokine levels are increased. CAR-T in the cerebrospinal fluid of patients, and elevated IL-6 levels in the cerebrospinal fluid have been observed in patients experiencing neurotoxicity. Neurologic events may occur at different times than CRS or in the absence of CRS toxicities, which suggests that at least in some cases, the neurologic toxicity might have a different mechanism than many of the other toxicities such as hypotension and fever. Reported neurologic toxicities include headaches, confusion, alterations in wakefulness, hallucinations, dysphasia, ataxia, apraxia, facial nerve palsy, tremor, dysmetria, and seizures. Neurologic toxicities may also necessitate intubation and mechanical ventilation for airway protection in the absence of respiratory failure.

For assessment of ICANS, the grading criteria published by the ASTCT will be applied. This should ensure consistent grading throughout the trial and among all participating sites.

Table 19: Grading and management of immune effector cell-associated neurotoxicity syndrome

ASBMT ICANS Grade	Defining Features of Grade	Management
**Grade 1**	• ICE score 7-9 or depressed level of consciousness but awakens spontaneously<br>• No seizures, motor weakness, or raised ICP/cerebral edema	• Aspiration precautions and i.v. hydration<br>• Seizure prophylaxis with levetiracetam<br>• EEG<br>• Imaging of brain<br>• Consider tocilizumab if there is concurrent CRS
**Grade 2**	• ICE score 3-6 and/or depressed level of consciousness but awakens to voice<br>• No seizures, motor weakness, or raised ICP/cerebral edema	• Supportive care as in Grade 1<br>• Consider dexamethasone or its equivalent of methylprednisolone
**Grade 3**	• ICE score 0-2 and/or depressed level of consciousness but awakens to tactile stimulus<br>• Any clinical seizure focal or generalized that resolves rapidly, or non-convulsive seizures on EEG that resolve with intervention<br>• No motor weakness<br>• Focal/local edema on neuroimaging	• Supportive care as in Grade 1<br>• Dexamethasone 10-20 mg i.v. q6h or its equivalent of methylprednisolone<br>• Control seizures with benzodiazepines (for short-term control) and levetiracetam +/- phenobarbital and/or lacosamide<br>• High-dose methylprednisolone 1000 mg/d for focal/local edema
**Grade 4**	• ICE score 0 and patient is unarousable or requires vigorous or repetitive tactile stimuli to arouse or stupor or coma<br>• Life-threatening prolonged seizure (> 5 min); or repetitive clinical or electrical seizures without return to baseline in between<br>• Deep focal motor weakness such as hemiparesis or paraparesis	• Supportive care as in Grade 1<br>• High-dose methylprednisolone 1000 mg/d<br>• Control seizures with benzodiazepines (for short-term control) and levetiracetam +/- phenobarbital and/or lacosamide<br>• Imaging of spine for focal weakness<br>• Lower ICP by hyperventilation, hyperosmolar therapy with mannitol/hypertonic saline, and/or

ASBMT ICANS Grade	Defining Features of Grade	Management
	• Diffuse cerebral edema on neuroimaging; decerebrate or decorticate posturing; or cranial nerve VI palsy; or papilledema; or Cushing's triad	neurosurgery consultation for ventriculopperitoneal shunt in patients with cerebral edema

Abbreviations: ASBMT, American Society for Bone Marrow Transplant; CRS, cytokine release syndrome; EEG, electroencephalogram; ICANS, immune effector cell-associated neurotoxicity syndrome; ICE, immune effector cell-associated encephalopathy score; ICP, intracranial pressure; i.v.-, intravenous.
Source: Neelapu 2019.

The ASTCT published the Consensus Grading System for CRS and neurological toxicity which they termed as ICANS (Lee et al. 2019). The ASBMT consensus grading system for ICANS was developed by further refining the neurological toxicity grading proposed by the CAR-T-cell-therapy-associated TOXicity (CARTOX) working group. The grading of ICANS requires assessment of five neurological domains and evaluates for encephalopathy by a 10-point scoring system using the ICE assessment tool:

- Orientation: orientation to year, month, city, hospital: 4 points
- Naming: ability to name three objects (e.g., point to clock, pen, button): 3 points
- Following commands: ability to follow simple commands (e.g., "Show me two fingers" or "Close your eyes and stick out your tongue"): 1 point
- Writing: ability to write a standard sentence (e.g., "Our national bird is the bald eagle"): 1 point
- Attention: ability to count backwards from 100 by 10: 1 point

Other neurological domains assessed for ICANS grading include level of consciousness, seizures, motor weakness, and raised intracranial pressure/cerebral edema. Management of ICANS based on the ASTCT was published later (Neelapu 2019).

6.6.4.4 CAR-T-related fulminant hemophagocytic lymphohistiocytosis (macrophage activation syndrome)

A patient might have hemophagocytic lymphohistiocytosis/MAS if he/she had a peak serum ferritin level of > 10000 ng/mL during the CRS phase of CAR-T therapy (typically the first 5 d after cell infusion) and subsequently developed any two of the following:

- Grade ≥ 3 increase in serum bilirubin, AST, or ALT levels
- Grade ≥ 3 oliguria or increase in serum creatinine levels
- Grade ≥ 3 pulmonary edema
- Presence of hemophagocytosis in bone marrow or organs based on histopathological assessment of cell morphology and/or CD68 IHC

Guidelines for the management of hemophagocytic lymphohistiocytosis/MAS are given in Figure 13.

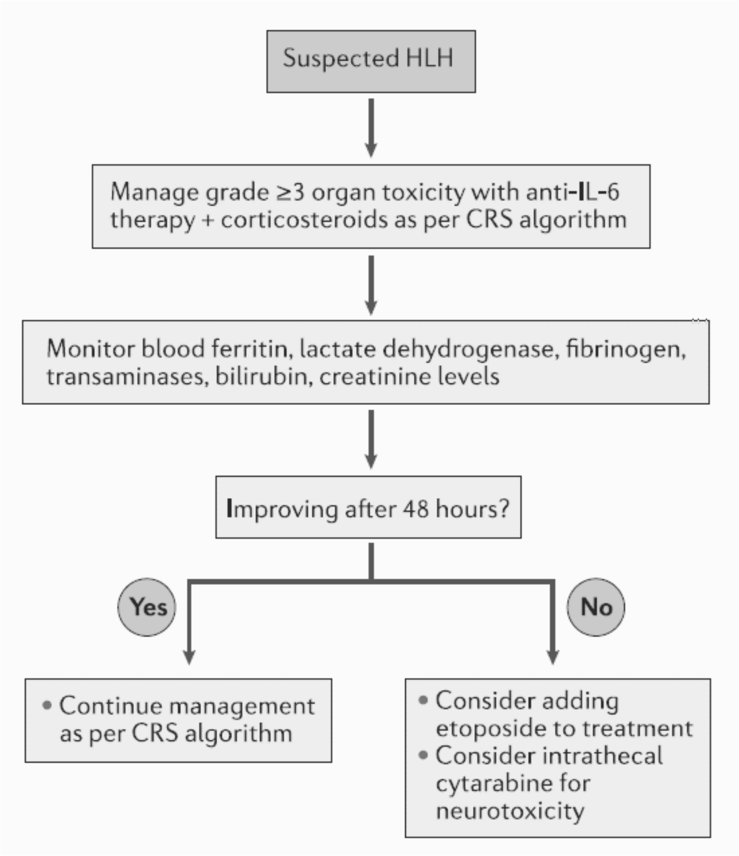

Figure 13: Management of CAR-T-related fulminant hemophagocytic lymphohistiocytosis (macrophage activation syndrome)

Abbreviations: Anti-IL-6, interleukin-6 antibody; CRS, cytokine release syndrome; HLH, hemophagocytic lymphocytosis.
Source: Neelapu et al. 2018.

6.6.4.5 Tumor lysis syndrome

Close monitoring for TLS before and after chemotherapy and CLDN6 CAR-T/CLDN6 CAR-T(A) infusions, including blood tests (potassium, uric acid, etc.) will be performed as follows:

Screening Phase A + B:

- Prophylactic allopurinol, or a non-allopurinol alternative (e.g., febuxostat), and increased oral/i.v. hydration prior to lymphodepleting chemotherapy and CLDN6 CAR-T/CLDN6 CAR-T(A) infusion should be given in patients with elevated uric acid or high tumor burden.
- Early and prompt implementation of supportive care in case of symptoms of acute TLS (i.v. hydration and rasburicase as clinically indicated, when uric acid continues to rise despite allopurinol/febuxostat and fluids).

Post infusion monitoring phase:

- Frequent monitoring of the following laboratory tests (two to three times per week for 3 weeks from start of LD chemotherapy, then weekly): potassium, phosphorus, calcium, creatinine, and uric acid
- Encourage oral hydration

Laboratory and clinical TLS is defined as follows:

Laboratory TLS is defined as two or more of the following values 3 d prior to or following CLDN6 CAR-T/CLDN6 CAR-T(A) infusion:

- Uric acid ≥ 8 mg/dL or 25% increase from baseline
- Potassium ≥ 6 mEq/L or 25% increase from baseline
- Phosphorus ≥ 4.5 mg/dL (adults) or 25% increase from baseline
- Calcium ≤ 7 mg/dL or 25% decrease from baseline

If none or one of the laboratory values above are abnormal, continue to manage with allopurinol or a non-allopurinol alternative (e.g., febuxostat) and oral hydration. Consider i.v. fluids and rasburicase if uric acid levels remain elevated, and consider in-hospital monitoring.

If laboratory TLS is identified, manage with i.v. fluids, perform laboratory blood tests every 6 to 8 h and initiate in-patient care if patient is not currently hospitalized. Cardiac monitoring should be considered, and rasburicase should be considered if uric acid levels remain elevated.

Clinical TLS is defined as the presence of laboratory TLS plus ≥ 1 of these criteria in the absence of other causes:

- Serum creatinine ≥ 1.5 × the upper limit of the age-adjusted normal range
- Symptomatic hypocalcemia
- Cardiac arrhythmia

If clinical TLS is identified, manage with i.v. fluids, laboratory blood tests every 6 to 8 h, cardiac monitoring, rasburicase/allopurinol/febuxostat and in-patient care if patient is not currently hospitalized (consider ICU).

Criteria modified from (Cairo and Bishop 2004).

6.6.4.6 Flu-like symptomology potentially related to CLDN6 RNA-LPX

It is anticipated that patients will experience AEs of flu-like symptomatology following the administration of CLDN6 RNA-LPX due to its mechanism of action. This may include but is not limited to fever, tachycardia, arthralgia, myalgia, headache, nausea, chills and rigors.

Treatment of these events is dependent on the discretion of the Principal Investigator and Sub-investigators, however management suggestions are provided below.

- Treat fever with acetaminophen or NSAIDs with a dose per institution's recommendation as needed.
- Patients should be pre-medicated with standard therapeutic doses of acetaminophen, or NSAIDs, at least 60 min before CLDN6 RNA-LPX administration (see also Section 6.1.3.1).
- Corticosteroids should be avoided as either prophylaxis or treatment as they counteract the effects of CLDN6 RNA-LPX (see also Section 6.1.3.1).

6.6.5 Safety stopping criteria

Across all patients in Part 1 and Part 2, the following rules apply:

Dosing will stop **temporarily** when one of the following criteria is met:

1. A life-threatening AE is experienced.
2. A DLT is experienced.
3. A pre-planned dose cohort completed recruitment with three or six DLT-evaluable patients. This will be a temporary hold for the trial until the safety and other data are reviewed and a decision is made to move to the next dose level.

In individual patients, treatment with CLDN6 CAR-T, CLDN6 CAR-T(A), or CLDN6 RNA-LPX should be discontinued due to safety concerns under the following conditions:

1. If the patient experiences an AE fulfilling the DLT criteria after the DLT period has ended in the dose escalation or during the expansion that fails to resolve to ≤ Grade 1 within 21 d after the planned dosing date or any other dose delay of more than 21 d due to toxicity possibly related to either or both IMPs, unless otherwise approved by the Sponsor's Medical Monitor.
2. In case of a drug-related or life-threatening Grade 4 AE that does not fulfill the DLT criteria (excluding asymptomatic Grade 4 elevations in non-hematological laboratory values that resolve to ≤ Grade 2 within 14 d [+/- medical intervention]) unless otherwise approved by the Sponsor's Medical Monitor.
3. First occurrence of life-threatening anaphylactic reactions clearly related to either CLDN6 CAR-T, CLDN6 CAR-T(A), or CLDN6 RNA-LPX requiring treatment with epinephrine or high-dose corticosteroid.
4. More than two dose reductions are required (the lowest applicable dose is 12.5 µg total RNA).
5. Re-treatment at a lower dose leads to a third identical TEAE of Grade > 3.

Treatment *may* be discontinued if any of the following applies, unless the Sponsor's Medical Monitor approves continuation of treatment:

1. TEAE of Grade > 3 which fails to resolve to Grade < 1 within 21 d after the planned dosing date.
2. Drug-related TEAE of Grade 4.
   Note: Asymptomatic Grade 4 elevations of non-hematological laboratory values that resolve to CTCAE Grade < 2 within 14 d (with or without medical intervention) do not require treatment discontinuation.
3. Dose delay of more than 21 d due to a CLDN6 RNA-LPX-related TEAE.

Please note: Patients should, whenever possible, irrespective of the reason for discontinuation, be examined as soon as possible.

Dosing will stop **permanently** when one of the following criteria is met:

1. All dose cohorts have been recruited as planned.
2. MTD (dose escalation will be stopped if ≥ 2 of six patients in any dose cohort experience a DLT) and/or RP2D of CLDN6 CAR-T or CLDN6 CAR-T(A) as monotherapy are defined.
3. MTD in any dose level in Part 1 is defined. As dose level in Part 2 at any given time will always be lower than activated dose level in Part 1, further enrollment and dose escalation in the whole trial will stop.

6.7 TREATMENT AFTER THE END OF TRIAL

Currently no treatment with the IMP is planned after the EoT.

7 DISCONTINUATION OF TRIAL TREATMENT AND PATIENT DISCONTINUATION/WITHDRAWAL

Any patient who discontinues treatment should be encouraged to return to the trial site for a Safety/EoT Follow-up Visit within 90 d of the last dose of IMP and no more than 97 d after the last study treatment administration. This EoT Visit would replace the scheduled regular visit if it fell within the same visit window. The primary reason for discontinuation must be recorded on the appropriate eCRF.

After patients complete or prematurely discontinue participation in the trial, they will be asked to participate in a separate 15-year Long-term Follow-up (LTFU) trial (Section 12.8).

7.1 DISCONTINUATION OF TRIAL TREATMENT

Patients will be discontinued from trial treatment if any of the following applies:

- Patient withdraws his/her consent to trial participation.
- Unacceptable toxicity.
- Development of any intercurrent illness or situation which may, in the judgment of the investigator, affect assessments of clinical status and trial endpoints to a relevant degree.
- Development of a second malignancy that requires a different treatment.
- Substantial non-compliance with trial procedures.
- Use of illicit drugs, prohibited concomitant therapies, or other substances that may, in the opinion of the investigator, have a reasonable chance of contributing to toxicity or otherwise confound the results.
- Patient is lost to follow-up.
- Treatment should be discontinued in all patients who exhibit evidence of PD by RECIST 1.1, unless they fulfill the criteria described in Section 6.1.3.2.1.

A patient, who for any reason (e.g., failure to satisfy the selection criteria) terminates the trial during screening is regarded a "screening failure". Re-screening of a patient is allowed. Re-enrollment is not allowed.

The investigator has the right to discontinue a patient from the trial for any medical condition that the investigator determines may jeopardize the patient's safety. For example, if there is a change in-patient eligibility, for reasons of non-compliance (e.g., missed doses, visits) or pregnancy, or if the investigator determines it is in the best interest of the patient to discontinue.

See the SoA (Part 1: Table 2 or cross-over: Table 3, Standard Part 2: Table 5, Optional Part 2: Table 7, and Optional Part 2 without LD: Table 9) for data to be collected at the time of treatment discontinuation and follow-up and for any further evaluations that need to be completed.

7.2 PATIENT EARLY DISCONTINUATION/WITHDRAWAL FROM THE TRIAL

A patient may withdraw from the trial at any time at his/her own request, or may be withdrawn at any time at the discretion of the investigator for safety, behavioral, compliance, or administrative reasons. This is expected to be uncommon. At the time of discontinuing from the trial, if possible, the patient should undergo all safety assessments planned for the EoT Visit, as shown in the SoA. Refer to the SoA (Section 1.3) for data to be collected at the time of trial discontinuation and follow-up and for any further evaluations that need to be completed. The patient will be permanently discontinued from the trial treatment, but remain in the trial for safety and efficacy follow-up (as applicable).

If the patient withdraws consent for disclosure of future information, the sponsor may retain and continue to use any data collected before such a withdrawal of consent. If a patient

withdraws from the trial, he/she may request destruction of any samples taken and not tested, and the investigator must document this in the site trial records.

7.3 LOST TO FOLLOW-UP

A patient will be considered lost to follow-up if he or she repeatedly fails to return for scheduled visits and is unable to be contacted by the trial site.

The following actions must be taken if a patient fails to return to the clinic for a required trial visit:

- The site must attempt to contact the patient and reschedule the missed visit as soon as possible and counsel the patient on the importance of maintaining the assigned visit schedule and ascertain whether or not the patient wishes to and/or should continue in the trial.
- Before a patient is deemed lost to follow-up, the investigator or designee must make every effort to regain contact with the patient (where possible, three telephone calls and, if necessary, a certified letter to the patient's last known mailing address or local equivalent methods). These contact attempts should be documented in the patient's medical record.
- Should the patient continue to be unreachable, he/she will be considered to have withdrawn from the trial.

Discontinuation of specific sites or of the trial as a whole are handled as part of Appendix 1 (Section 12.1).

8 TRIAL ASSESSMENTS AND PROCEDURES

Trial procedures and their timing are summarized in the SoA. Protocol waivers or exemptions are not allowed.

Immediate safety concerns should be discussed with the sponsor immediately upon occurrence or awareness to determine if the patient should continue or discontinue trial treatment. Adherence to the trial design requirements, including those specified in the SoA, is essential and required for trial conduct. All screening evaluations must be completed and reviewed to confirm that potential patients meet all eligibility criteria. The investigator will maintain a screening log to record details of all patients screened and to confirm eligibility or record reasons for screening failure, as applicable.

Procedures conducted as part of the patient's routine clinical management (e.g., blood count) and obtained before signing of the ICF may be used for screening or baseline purposes provided the procedures met the protocol-specified criteria and were performed within the time frame defined in the SoA.

8.1 EFFICACY ASSESSMENTS

8.1.1 Tumor response

Tumor response will be evaluated by the investigator according to RECIST v1.1 and iRECIST using unidimensional measurement such as CT scan (with i.v. contrast, chest, abdomen, pelvis) or MRI in case of i.v. contrast contraindication. Assessment of CT/MRI scans as tumor assessments will be performed at the sites. Independent central reading of CT or MRI scans may be performed, both prospectively and retrospectively, at the decision of the sponsor.

Tumor assessment will be performed during screening (see SoA), at 6 weeks (±7 d) post infusion, then every 6 weeks (±7 d) for 50 weeks and every 12 weeks (±7 d) thereafter. Tumor assessments performed within 4 weeks prior to screening do not have to be repeated.

Tumor assessments will be discontinued in case of confirmed disease progression and discontinuation of CLDN6 RNA-LPX treatment, whichever occurs last.

8.1.2 Performance status

The Eastern Cooperative Oncology Group (ECOG) Performance Status of patients will be assessed by the physician at the time points indicated in the SoA.

8.2 SAFETY ASSESSMENTS

Planned time points for all safety assessments are provided in the SoA.

The safety outcome measures for this trial consist of monitoring the incidence and severity of AEs, DLTs, and IRRs using NCI CTCAE v5.0, the incidence of laboratory abnormalities (hematology testing, coagulation, serum chemistries, and urinalysis), detection of cytokine release, and collection of physical examination findings, 12-lead ECGs, and vital signs.

8.2.1 Physical examinations

Physical examinations will be performed daily during hospitalization periods for assessment of toxicities. This includes, but is not limited to, the ICE score.

A complete physical examination will be performed at screening and the EoT Visit. The complete physical examination includes evaluation of the head, eyes, ears, nose, and throat, and the cardiovascular, dermatological, musculoskeletal, respiratory, gastrointestinal, genitourinary, and neurological systems. Depending on timing (before or after signing the ICF for trial participation), clinically significant abnormalities observed at screening will be recorded on the general medical history page or on the AE page of the eCRF. At subsequent visits, examination will be performed and new or worsened clinically significant abnormalities will be recorded on the AE page of the eCRF.

8.2.2 Vital signs

Vital signs (systolic and diastolic blood pressure, heart rate, body temperature, and oxygen saturation) will be measured in a seated or recumbent position after at least 5 min rest, according to the SoA. Depending on timing (before or after signing the ICF for trial participation), clinically significant abnormalities observed at screening will be recorded on the general medical history section or on the AE page of the eCRF. At subsequent visits, new or worsened clinically significant abnormalities will be recorded on the AE section of the eCRF.

Table 20: Vital signs

CLDN6 CAR-T/CLDN6 RNA-LPX re-administration (up to 30 min before administration)
240 min after injection or start of infusion (± 15 min)
360 min after injection or start of infusion (± 15 min)

8.2.3 Electrocardiograms

12-lead ECG recordings will be obtained during screening, and as clinically indicated at other time points. Patients should be resting in a supine position for at least 10 min prior to ECG recording.

8.2.4 Clinical safety laboratory assessments

- See Section 12.2 for the list of clinical laboratory tests to be performed and to the SoA for the timing and frequency.
- The investigator must review the laboratory report, document this review, and record any clinically relevant changes occurring during the trial in the AE section of the eCRF. The laboratory reports must be filed with the source documents. Clinically significant abnormal laboratory findings are those which are not associated with the underlying disease, unless judged by the investigator to be more severe than expected for the patient's condition.
- All laboratory tests with values considered clinically significantly abnormal during participation in the trial or within 30 d after the last dose of trial treatment should be repeated until the values return to normal or baseline or are no longer considered clinically significant by the investigator or medical monitor.
    - If such values do not return to normal/baseline within a period of time judged reasonable by the investigator, the etiology should be identified and the sponsor notified.
    - All protocol-required laboratory assessments, as defined in Appendix 2 (Section 12.2), must be conducted in accordance with the Laboratory Manual and the SoA.

- o If laboratory values from non-protocol-specified laboratory assessments performed at the institution’s local laboratory require a change in patient management or are considered clinically significant by the investigator (e.g., SAE or AE or dose modification), then the results must be recorded in the eCRF.

Routine clinical laboratory safety tests (e.g., blood chemistry) will be performed in a local clinical laboratory.

All other tests and blood biomarker panel analysis will be performed by central laboratories.

A Laboratory Manual will be provided to the sites, which specifies the procedures for collection, processing, storage, and shipment of samples, as well as central laboratory contact information, specific to this clinical research trial. Refer to the SoA for the timing and frequency of clinical laboratory tests.

8.3 ADVERSE EVENTS AND SERIOUS ADVERSE EVENTS

AEs may be reported by the patient or, when appropriate, by a caregiver, surrogate, or the patient's legally authorized representative, spontaneously, or at the scheduled study visit.

The investigator and any qualified designees are responsible for detecting, documenting, and recording events that meet the definition of an AE (Section 12.3.1) or SAE (Section 12.3.2) and remain responsible for following up AEs and SAEs in accordance with the procedures set out in Section 12.3.3 and Section 12.3.4.

8.3.1 Time period and frequency for collecting AE and SAE information

All AEs and SAEs will be collected from the signing of the main ICF until 90 d after the last IMP administration (either IMP-1, IMP-1(A), or IMP-2).

AEs and SAEs occurring later than 90 d after last IMP administration only have to be reported to the sponsor if the investigator suspects a causal relationship to the IMP(s) or the trial procedure.

SAEs occurring prior to signing of main ICF only have to be reported to the sponsor if related to a trial procedure (e.g., biopsy).

All SAEs will be recorded and reported to the sponsor or designee immediately and under no circumstance should this exceed 24 h from the time of awareness of the event at the clinical trial site, as indicated in Appendix 3 (Section 12.3). The investigator will submit any updated SAE data to the sponsor within 24 h of it being available.

Investigators are not obligated to actively seek AE or SAE after conclusion of the trial participation. However, if the investigator learns of any SAE, including a death, at any time after a patient has been discharged from the trial, and he/she considers the event to be reasonably related to the trial treatment or trial participation, the investigator must promptly notify the sponsor.

8.3.2 Method of detecting AEs and SAEs

The method of recording, evaluating, and assessing causality of AE and SAE and the procedures for completing and transmitting SAE reports are provided in Section 12.3.

Care will be taken not to introduce bias when detecting AEs and/or SAEs. Open-ended and non-leading verbal questioning of the patient is the preferred method to inquire about AE occurrences.

8.3.3 Follow-up of AEs and SAEs

After the initial AE/SAE report, the investigator is required to proactively follow each patient at subsequent visits/contacts. All AEs/SAEs will be followed until resolution, stabilization, the event is otherwise explained, or the patient is lost to follow-up. If no final status is reached at EoT, the investigator must confirm the unavailability of a final status. Further information on follow-up procedures is provided in Section 12.3.

8.3.4 Regulatory reporting requirements for SAEs

- Prompt notification by the investigator to the sponsor of a SAE is essential so that legal obligations and ethical responsibilities towards the safety of patients and the safety of a trial treatment under clinical investigation are met.
- The sponsor has a legal responsibility to notify both the local regulatory authority and other regulatory agencies about the safety of a trial treatment under clinical investigation. The sponsor will comply with country-specific regulatory requirements relating to safety reporting to the regulatory authority, IRB/IEC, and investigators.
- For all studies except those utilizing medical devices investigator safety reports must be prepared for suspected unexpected serious adverse reactions (SUSAR) according to local regulatory requirements and sponsor policy and forwarded to investigators as necessary.
- An investigator who receives an investigator safety report describing an SAE or other specific safety information (e.g., summary or listing of SAEs) from the sponsor will review and then file it along with the IB and will notify the IRB/IEC, if appropriate according to local requirements.

8.3.5 Pregnancy

- Details of all pregnancies in female patients and female partners of male patients will be collected after the start of trial treatment and until the end of LTFU.
- If a pregnancy is reported, the investigator should inform the sponsor within 24 h of learning of the pregnancy and should follow the procedures outlined in Section 12.4.
- Abnormal pregnancy outcomes (e.g., spontaneous abortion, fetal death, stillbirth, congenital anomalies, ectopic pregnancy) are considered SAEs.

8.3.6 Disease-related events and/or disease-related outcomes not qualifying as AEs or SAEs

The progression of underlying disease during trial participation is not considered an AE (Section 12.3.3.6). The following disease-related events (DREs) are common in patients with CLDN6-positive relapsed or refractory advanced solid tumors and can be medically relevant and/or serious/life-threatening:

- The progression of underlying disease (e.g., new metastases)

Because these events are typically associated with the disease under trial, they will not be reported according to the standard process for expedited reporting of SAEs even though the event may meet the definition of a SAE. These events will be recorded on the corresponding eCRF page in the patient's eCRF.

NOTE: However, if any of the following conditions applies, then the event must be recorded and reported as an AE/SAE (instead of a DRE):

- Specific symptoms at time of progression that may be considered as being caused by reasons other than the progression of underlying disease itself, and fatal cases where a cause other than the disease progression itself may not be discounted, will have to be documented as AEs and reported as SAEs if applicable.
  OR
- The investigator considers that there is a reasonable possibility that the event was related to trial treatment.

8.4 TREATMENT OF OVERDOSE

Overdose of CLDN6 CAR-T, CLDN6 CAR-T(A), and CLDN6 RNA-LPX is not expected as only the required dose will be supplied. For CLDN6 CAR-T and CLDN6 CAR-T(A), the correct dose is released by the Qualified Person and therefore an overdose is not expected. For the CLDN6 RNA-LPX, the requested dose is prepared and provided by the Pharmacy and therefore an overdose is also not expected.

8.5 PHARMACOKINETICS

Pharmacokinetic parameters (C_{max}, T_{max}, AUC, and other relevant parameters) will be determined to characterize the cellular PK profile (levels, persistence, and trafficking) of CLDN6 CAR-T/CLDN6 CAR-T(A) in blood and other tissues if available.

Samples for pharmacokinetic analyses will be collected as specified in the SoA (Section 1.3). Details on the collection, processing, storage, and shipment of samples will be provided in separate documents (e.g., Laboratory Manual). The actual date and time (24-hour clock time) of each sample will be recorded.

8.6 PHARMACODYNAMICS

Refer to Section 8.8.2.

8.7 GENETICS

Genetic as well as non-genetic analyses will be part of the biomarker investigations in this trial, if approved by local ECs / IRBs and competent authorities, and are described in Section 8.8.

8.8 BIOMARKERS

Biomarker investigations in this trial will include pharmacodynamic biomarkers to confirm biological activity of CLDN6 CAR-T/CLDN6 CAR-T(A) as monotherapy and in combination with CLDN6 RNA-LPX cancer vaccine, and predictive biomarkers that may identify patients that can benefit from therapy.

Biomarker assessments will focus on:

- Detection and characterization of CLDN6 CAR-T/CLDN6 CAR-T(A) in PBMCs and tumor tissue (is feasible).
- Evaluating tumor CLDN6 expression (RNA and/or protein) as a potential predictive biomarker that can identify patients that may respond to CLDN6 CAR-T/CLDN6 CAR-T(A) +/- CLDN6 RNA-LPX.
- Evaluating cytokines as a potential safety marker and/or pharmacodynamic biomarker and early biomarker of response.
- Evaluating the tumor and peripheral immunophenotype as a potential pharmacodynamic biomarker and early biomarker of response.
- Additional biomarker assessments may be performed on tumor tissues and/or whole blood samples (e.g., additional markers in flow cytometry, tumor mutational burden [TMB], immune gene expression signatures, comprehensive molecular profiling) to predict patient response to CLDN6 CAR-T/CLDN6 CAR-T(A) monotherapy and in combination with CLDN6 RNA-LPX.

8.8.1 CLDN6 expression at pre-screening

Since the CAR-T administered are directed against the CLDN6 protein, only patients with CLDN6-positive tumor tissue can participate in this trial. Therefore, patients must provide an archival FFPE tumor tissue sample during pre-screening to assess CLDN6 protein expression using an analytically validated IHC assay at a central laboratory. If it is not feasible to provide archival tumor samples, patients can undergo a fresh tumor resection or core needle biopsy if the investigator does not consider this to pose a significant risk to the patient.

8.8.2 Pharmacodynamic biomarkers

Serum or plasma samples will be collected to assess pharmacodynamic biomarkers such as cytokines and inflammation markers at multiple time points (see SoA tables in Section 1.3). Systemic effects in patients will be assessed using the cytokine multiplex assay (e.g., TNF, IFN-γ, IL-2, IL-10, soluble IL-2Rα, IP-10, IL-12, IFN-α, IL-6, soluble IL-6R).

8.8.3 Exploratory assessments of blood biomarkers

Planned time points for biomarker sampling are provided in the SoA tables (Section 1.3).

Exploratory biomarker assessments will include (but are not limited to) blood sampling for exploratory functional assays (e.g., intracellular cytokine staining). The list of planned assessments may change, i.e., listed assessments may be deleted or added, depending on the results obtained.

CAR-T expansion, persistence and functionality will be assessed using flow cytometry as well as qPCR methodology and in selected patients by single cell RNAseq of CAR-T. CAR-T mediated anti-tumor efficacy will be assessed using the following biomarkers: e.g., CA125 (endometrial and ovarian tumors), e.g., hCG-, AFP (testicular tumors), e.g., CEA (lung tumors), e.g., AFP, CEA, CA72-4, CA19-9 (gastric tumors).

Anti-CAR humoral response (CAR immunogenicity assay) will be measured as clinically indicated.

8.8.4 Exploratory assessment of tumor tissue

On trial, tumor tissue should be provided at baseline, pre-treatment, for immune genomic profiling and characterization. Tumor tissue may be analyzed using whole exome or targeted NGS to determine TMB and neoantigen load, microsatellite instability status, T-cell receptor (TCR) and B-cell receptor diversity and changes in clonality, HLA loss-of-heterozygosity, and mutations/insertions/deletions in genes associated with resistance. Whole transcriptome RNA-sequencing may be performed to determine immune signatures and changes in RNA expression of CLDN6. IHC methods will be used to assess changes in CLDN6 expression on

treatment and in addition other immune and tumor markers in order to characterize infiltrating T cells and the tumor microenvironment.

If deemed feasible by the investigator, a fresh tumor biopsy on treatment should be provided, ideally 2-3 weeks after CAR-T administration (e.g., d17) and at progression. The patient may be called in for an unscheduled visit, and tumor biopsies may be performed if patients have unscheduled biopsies or tumor tissue resection during the course of the trial. The tumor sample should be a core needle biopsy, or from resected tumor tissue.

In case it is not feasible to meet the required tumor tissue criteria, the sponsor medical monitor's approval for enrollment is needed.

8.8.4.1 Assessment of biomarkers in ascites and pleural effusion

If samples are available, flow cytometry will be used to assess CAR-T and tumor infiltration.

8.8.5 Biomarker sample collection

8.8.5.1 Timing

Samples for biomarker analyses will be collected as specified in the SoA (Section 1.3). Biomarker analyses are dependent upon the availability of appropriate biomarker assays and clinical response rates. Biomarker analysis may be deferred or not performed if, during or at the end of the trial, it becomes clear that the analysis will not have sufficient scientific value for biomarker evaluation, or if there are not enough samples or responders to allow for adequate biomarker evaluation. Additionally, during the course of the trial, any tumor tissue collected per the institutional guidance or the SOC will be used for biomarker analyses. A maximum of three samples may be collected at additional time points during the study if warranted and agreed upon between the investigator and the sponsor. Sampling time points might also be moved or removed.

8.8.5.2 Sample handling and storage

Details on the collection, processing, shipment and storage of samples will be provided in separate documents (e.g., Laboratory Manual). Samples may be stored for a maximum of 15 years (or according to local regulations) following the end of the trial at a facility selected by the sponsor to enable further analyses.

8.8.5.3 Reporting

Some results of biomarker investigations may be reported separately (e.g., in a biomarker evaluation report).

8.8.6 Other biomarkers

In addition to the biomarkers described above, further biomarkers related to, e.g., the mode-of-action or the safety of the study intervention and similar drugs may be investigated. The same applies to further biomarkers deemed relevant to cancer and associated health problems. These investigations may include e.g., diagnostic, safety, pharmacodynamic, monitoring, or potentially predictive biomarkers.

8.9 IMMUNOGENICITY ASSESSMENTS

Humoral immunogenicity is only analyzed if clinically indicated by assessing the presence of anti-CAR antibodies pre- and post-ACT.

8.10 HEALTH ECONOMICS OR MEDICAL RESOURCE UTILIZATION AND HEALTH ECONOMICS

Health Economics/Medical Resource Utilization and Health Economics parameters are not evaluated in this trial.

9 STATISTICAL CONSIDERATIONS

9.1 STATISTICAL HYPOTHESES

The primary objectives of Parts 1 and 2 are to assess the safety profile and to identify the MTD and/or RP2D. Hence, no statistical hypothesis is under test for Parts 1 and 2. For Part 3, hypothesis testing for each expansion cohort may be introduced through protocol amendment.

9.2 SAMPLE SIZE DETERMINATION

The sample size for Part 1 and Part 2 is driven by the 3+3 trial design. In Part 1 and Part 2 the sample size will be up to 18 DLT-evaluable patients depending on the number of DLTs that may occur. After completion of Part 1 and Part 2 with CLDN6 CAR-T manufactured with a manual process, the dose escalation will be repeated with CLDN6 CAR-T(A) manufactured with an automated process. According to the 3+3 design, up to 96 patients can be enrolled into the automated cohorts in Part 1 and Part 2.

The objective for Part 3 is to further investigate the safety profile and to assess the efficacy of the IMP in different indications in combination with other approved anti-cancer agents. The final sample size calculations will be introduced using a protocol amendment.

9.3 ANALYSIS SETS

The following analysis sets are defined:

Table 21: Analysis sets

Analysis set	Description
**Screened**	The Screened Set is defined as all patients who signed an informed consent.
**Enrolled**	The Enrolled Set is defined as all patients who have provided as leukapheresis.
**Modified intent-to-treat**	The modified Intent-to-treat (mITT) Set is defined as all patients who are assigned to IMP-1 or IMP-1(A) and have a baseline and at least one on-treatment/post treatment tumor assessment.<br>This analysis set may only be used in Part 3 of the trial.<br>Patients who have received an out of specification product as described in Section 6.1.2.3 will not be included in the mITT.
**Safety**	The Safety Set is defined as all patients who receive IMP-1 or IMP-1(A).
**Per Protocol**	The Per Protocol Set (PPS) is defined as all patients who receive IMP-1 or IMP-1(A) and fulfill the following criteria:<br>• The absence of any important protocol deviations<br>• The completion of a minimal exposure to the treatment<br>• Availability of baseline and at least one on-treatment/ post treatment tumor assessment<br>Important deviations will lead to an exclusion of patients from the PPS and will be agreed at the data review meeting prior to database snapshot for the primary analysis. Protocol deviations that may be considered important will be specified in the SAP.<br>This analysis set may only be used in Part 3 of the trial.<br>Patients who have received an out of specification product as described in Section 6.1.2.3 will not be included in the PPS.
**DLT Evaluation**	The DLT evaluation set includes all patients from the Safety Set who have<br>• experienced a DLT during the DLT evaluation period (28 d following first dose), or<br>• have completed the DLT evaluation period (28 d following first dose) and meet the minimum exposure criterion.<br>Patients who do not experience any DLT during the DLT observation period are considered to be evaluable if they have been observed for a minimum of 28 d following the first dose and are considered to have sufficient safety data to conclude that a DLT did not occur. A patient is considered to have met the minimum exposure criterion if the patient receives the first full infusion of the CLDN6 CAR-T at the intended dose without interruption and modification in Part 1; and one full infusion of the CLDN6 CAR-T at the intended dose without interruption and modification and at least one full dose of vaccination in Part 2.<br>Patients who have received an out of specification product as described in Section 6.1.2.3 will not be included in the PPS.

Abbreviations: CAR-T, Chimeric antigen receptor T cell; CLDN6, claudin 6; DLT, Dose-limiting toxicity; IMP-1, Investigational medicinal product 1 (manual process); IMP-1(A), Investigational medicinal product 1 (automated process); mITT, Modified intent-to-treat; PPS, Per Protocol Set; SAP, Statistical analysis plan.

The DLT evaluation set will be used for the evaluation of DLTs in order to assess the MTD and the RP2D. The Safety Set will be used for all other safety analyses, and the modified Intent-to-treat Set will be used for efficacy analyses.

9.4 STATISTICAL ANALYSES

Statistical analyses will be performed by the sponsor or a designated contract research organization (CRO). All statistical analyses will be carried out using SAS®, Version 9.4 or higher, and/or other statistical software as required.

The statistical analysis plan (SAP) will be finalized prior to snapshot for the main analysis and it will include a more technical and detailed description of the statistical analyses described in this section. Any deviations from the planned analyses described in the final SAP will be described and justified in the clinical study report. This section is a summary of the planned statistical analyses of the most important endpoints including primary and key secondary endpoints.

9.4.1 General considerations

In general, the statistical analysis will be performed by cohort. Moreover, cohorts may be combined as appropriate. In particular, the same dose levels of IMP-1 and IMP-1(A) may be combined, if both IMPs are deemed to be comparable.

Continuous variables will be summarized by cohort using the following descriptive statistics: number of patients (n), mean, standard deviation, median, minimum and maximum.

Categorical variables will be summarized by cohort, presenting absolute and relative frequencies (n and %) of patients in each category.

Time-to-event-endpoints (DOR, PFS and OS) will be analyzed using Kaplan-Meier methodology and censored in accordance with the FDA Guidance: “Clinical Trial Endpoints for the Approval of Cancer Drugs and Biologics” and the EMA guidance “Guideline on the evaluation of anti-cancer medicinal products in man”. Censoring rules will be defined in the SAP.

The median survival time (including 95% confidence limits according to [Brookmeyer and Crowley 1982]) and the first and third quartile will be presented for each cohort. Survival rates (including two-sided 95% confidence interval) based on Greenwood’s formula (Greenwood. 1926) as well as the number and percentage of patients with events, censored and under risk will be displayed for selected time points (e.g., at 3, 6, 12 months).

The time-to-event analysis will be illustrated using Kaplan-Meier plots.

Baseline is defined as last available value prior to first dose of CLDN6 CAR-T/CLDN6 CAR-T(A).

Data up to and including the clinical cut-off date for the statistical analysis will be taken into account for the statistical analysis.

9.4.2 Primary endpoints

Primary endpoints include the occurrence of DLTs within a patient during the DLT evaluation period to identify the MTD and RP2D, and the occurrence of TEAEs as well as the occurrence of dose reduction and discontinuation of IMP due to TEAE to assess the safety and tolerability of the IMP. The analysis of these endpoints are described in Section 9.4.5.

9.4.3 Secondary endpoints

Objective response rate

ORR is defined as the proportion of patients in whom a CR or PR is observed as best overall response. Patients not meeting the criteria for CR or PR, including those without any post-baseline tumor assessments, will be considered as non-responders.

ORR will be summarized with absolute and relative frequencies along with two-sided 95% Clopper-Pearson confidence intervals by cohort.

Disease control rate

DCR is defined as the proportion of patients in whom a CR or PR or SD (SD assessed at least 6 weeks after first dose) is observed as best overall response. Patients not meeting the criteria for CR or PR or SD, including those without any post-baseline tumor assessments, will be considered as non-responders.

DCR will be summarized with absolute and relative frequencies along with two-sided Clopper-Pearson 95% confidence intervals by cohort.

Duration of response

DOR is defined as the time from first objective response (CR or PR) to first occurrence of objective PD, or death from any cause, whichever occurs first. Only patients in whom a CR or PR is observed will be analyzed for DOR.

DOR will be analyzed using Kaplan-Meier methodology by cohort. Patients alive and without disease progression at data cut-off date or patients lost to follow-up will be censored at the day of their last tumor assessment. Additional censoring rules will be defined in the SAP.

9.4.4 Exploratory endpoints

The exploratory endpoints iORR, iDCR and iDOR will be analyzed in the same way as ORR, DCR and DOR as described in Section 9.4.3.

Progression-free survival

PFS is defined as the time from first dose of CLDN6 CAR-T/CLDN6 CAR-T(A) to first confirmed objective PD, or death from any cause, whichever occurs first.

PFS will be analyzed using Kaplan-Meier methodology by cohort. Patients alive and without disease progression or patients lost to follow-up at data cut-off date will be censored at the day of their last tumor assessment. Additional censoring rules will be defined in the SAP.

Overall survival

OS is defined as the time from first dose of CLDN6 CAR-T/CLDN6 CAR-T(A) to death from any cause.

OS will be analyzed using Kaplan-Meier methodology by cohort. Patients alive or patients lost to follow-up at date of analysis cut-off will be censored at the day of their last date known to be alive. Additional censoring rules will be defined in the SAP.

9.4.5 Safety endpoints

All safety analyses will be made on the Safety Set.

Adverse events

AEs will be coded using the most recent version of MedDRA® coding system to get an SOC and PT for each AE and graded for severity using NCI CTCAE v5.0.

A TEAE is defined as any AE with an onset date on or after the first administration of IMP-1 or IMP-1(A) (if the AE was absent before the first administration of IMP-1 or IMP-1(A)) or worsened after the first administration of IMP (if the AE was present before the first administration of IMP). AEs with an onset date more than 90 d after the last administration of any IMP will be considered as treatment-emergent only if assessed as related to any IMP by the Investigator. TEAEs will be summarized overall and by cohort.

The number and percentage of patients reporting at least one AE will be summarized by PT nested within SOC for each of the following AE types:

- Any AE
- Related AE
- Grade ≥ 3 AE
- Related Grade ≥ 3 AE
- Any SAE
- Related SAE
- SAE leading to death
- AE leading to dose reduction
- AE leading to permanent discontinuation of treatment
- DLT

Moreover, the number and percentage of patients with any AE will be summarized by worst NCI CTCAE grade by PT nested within SOC.

DLTs will be presented in terms of listings presenting the reported term and MedDRA PT and SOC, its time of onset, duration, and outcome, relationship, NCI CTCAE grade, and seriousness including dose exposure data.

9.4.6 Other analyses

Other analyses will be described in the SAP.

9.5 INTERIM ANALYSES

No formal interim analysis is planned. However, data will be reviewed after each cohort.

9.6 SAFETY REVIEW COMMITTEE

Refer to Section 12.1.5.

10 INVESTIGATORS AND STUDY ADMINISTRATIVE STRUCTURE

10.1 INVESTIGATORS AND TRIAL SITE PERSONNEL

10.1.1 Investigators

There must be an investigator at each trial site.

If the trial is conducted by a team of individuals at the trial site, the investigator leading and responsible for the team is called the Principal Investigator.

All persons assigned responsibility as Principal Investigator must sign a declaration of their responsibilities and their agreement to this protocol before any trial-related procedure is performed.

Curriculum vitae and/or other relevant documents confirming the current qualification of the investigators must be provided to the sponsor. This should include any previous training in the principles of GCP, experience obtained from work with clinical trials, and experience with patient care.

Documentation of all involved investigators must be maintained according to GCP and applicable regulatory requirements.

Documentation of all responsibilities assigned to country-specific Coordinating Investigator(s)/international Coordinating Investigator(s) must be maintained according to GCP and applicable regulatory requirements.

10.1.2 Trial site personnel assigned trial-related duties

The Principal Investigator may define appropriately qualified personnel at a trial site to perform significant trial-related procedures and/or to make trial-related decisions under his/her supervision. In this case, the Principal Investigator must maintain a signed list of the persons to whom they delegate significant trial-related duties/responsibilities; the delegated trial-related duties/responsibilities must be specified in the list.

When personnel or responsibility changes are made, the Principal Investigator must ensure that the relevant documentation is updated before any trial-related activities are performed.

Documentation of all involved trial site personnel performing significant trial-related procedures and/or making trial-related decisions must be maintained according to GCP and applicable regulatory requirements.

10.2 CONTRACT RESEARCH ORGANIZATIONS

Documentation of all involved CROs must be maintained according to GCP and applicable regulatory requirements. This includes documentation of any delegation of responsibilities to CROs.

10.3 THE SPONSOR AND SPONSOR'S PERSONNEL

The trial sponsor listed on the title page accepts the responsibilities of the sponsor according to GCP and applicable regulatory requirements.

The sponsor must designate appropriately qualified personnel to advise on trial-related topics. The trial site will be provided with contact details for these personnel before any trial-related procedure is performed.

A list of key sponsor personnel involved in the preparation of this protocol and the conduct of the trial, including their full names, titles, roles, and responsibilities, must be maintained.

10.4 OTHER BODIES (STEERING COMMITTEES)

An SRC will be established to review the safety data. The SRC will act according to its own written Standard Operating Procedures described in a charter, and will prepare written minutes of its meetings.

11 REFERENCES

Abuazza G, Becker A, Williams SS, Chakravarty S, Truong HT, Lin F, et al. Claudins 6, 9, and 13 are developmentally expressed renal tight junction proteins. Am J Physiol Renal Physiol. 2006;291(6):F1132-1141.

Ahmed N, Brawley VS, Hegde M, Robertson C, Ghazi A, Gerken C, et al. Human Epidermal Growth Factor Receptor 2 (HER2) - Specific Chimeric Antigen Receptor – Modified T cells for the Immunotherapy of HER2 Positive Sarcoma. J Clin Oncol. 2015;33(15):1688-1696.

Albers P, Albrecht W, Algaba F, Bokemeyer C, Cohn-Cedermark G, Fizazi K, et al. Guidelines on testicular cancer: 2015 update. Eur Urol. 2015;68(6):1054–1068.

American Cancer Society 2019a. Ovarian Cancer. Accessed at https://www.cancer.org/cancer/ovarian-cancer.html on 5 August 2019.

American Cancer Society 2019b. Uterine Cancer. Accessed at https://www.cancer.org/content/dam/CRC/PDF/Public/8609.00.pdf on 5 August 2019.

American Cancer Society 2019d. About lung cancer. Accessed at https://www.cancer.org/content/dam/CRC/PDF/Public/8703.00.pdf on 5 August 2019.

Anderson WJ, Zhou Q, Alcalde V, Kaneko OF, Blank LJ, Sherwood RI, et al. Genetic targeting of the endoderm with claudin-6CreER. Dev Dyn. 2008;237(2):504-512.

Avastin® USPI. Accessed at https://www.gene.com/download/pdf/avastin_prescribing.pdf on 5 August 2019.

Bang YJ, Van Cutsem E, Feyereislova A, Chung HC, Shen L, Sawaki A, et al. Trastuzumab in combination with chemotherapy versus chemotherapy alone for treatment of HER2 positive advanced gastric or gastrooesophageal junction cancer (ToGA): a phase 3, -openlabel, randomised- controlled trial. Lancet. 2010;376(9742):687–697.

Bates SE, Berry DA, Balasubramaniam S, Bailey S, LoRusso PM, Rubin EH. Advancing Clinical Trials to Streamline Drug Development. Clin Cancer Res. 2015;21(20):4527-4535.

Beatty GL, Haas AR, Maus MV, Torigian DA, Soulen MC, Plesa G, et al. Mesothelin-specific chimeric antigen receptor mRNA-engineered T cells induce anti-tumor activity in solid malignancies. Cancer Immunol Res. 2014;2(2):112–120.

Beck SD, Foster RS, Bihrle R, Einhorn LH, Donohue JP. Pathologic findings and therapeutic outcome of desperation post-chemotherapy retroperitoneal lymph node dissection in advanced germ cell cancer. Urol Oncol. 2005;23(6):423–430.

Birks DK, Kleinschmidt-DeMasters BK, Donson AM, Barton VN, McNatt SA, Foreman NK, et al. Claudin 6 is a positive marker for atypical teratoid/rhaboid tumors. Brain Pathol. 2010;20(1):140-150.

Bokhman JV. Two pathogenetic types of endometrial cancer. Gynecol Oncol. 1983;15(1):10-17.

Brentjens R, Yeh R, Bernal Y, Riviere I, Sadelain M. Treatment of chronic lymphocytic leukemia with genetically targeted autologous T cells: case report of an unforeseen adverse event in a phase I clinical trial. Mol Ther. 2010;18(4):666-668.

Brookmeyer R, Crowley J. A confidence interval for the median survival time. Biometrics. 1982;38:29-41.

Brown CE, Badie B, Barish ME, Weng L, Ostberg JR, Chang WC, et al. Bioactivity and Safety of IL13Rα2 Redirected Chimeric Antigen Receptor Cd8+ T cells in Patients with Recurrent Glioblastoma. Clin Cancer Res. 2015;21(18):4062-4072.

Bryk S, Färkkilä A, Bützow R, Leminen A, Tapper J, Heikinheimo M, et al. Characteristics and outcome of recurrence in molecularly defined adult-type ovarian granulosa cell tumors. Gynecol Oncol. 2016;143(3):571-577.

Cairo MS, Bishop M. Tumour lysis syndrome: new therapeutic strategies and classification. Br J Haematol. 2004;127(1):3-11.

Cairo MS, Coiffier B, Reiter A, Younes A (TLS Expert Panel). Recommendations for the evaluation of risk and prophylaxis of tumor lysis syndrome (TLS) in adults and children with malignant diseases: and expert TLS panel consensus. Br J Haematol. 2010;149(4):5768-586.

Cao G, Lei L, Xhu X. Efficiency and safety of autologous chimeric antigen receptor Tcells- therapy used for patients with lymphoma: a systematic review and meta-analysis. Medicine (Baltimore). 2019;98(42):1-8.

Chan JK, Cheung MK, Husain A, Teng NN, West D, Whittemore AS, et al. Patterns and progress in ovarian cancer over 14 years. Obstet Gynecol. 2006;108(3 Pt 1):521-528.

Charalampakis N, Economopoulou P, Kotsantis I, Tolia M, Schizas D, Liakakos D, et al. Medical management of gastric cancer: a 2017 update. Cancer Med. 2018;7(1):123–133.

Cheadle EJ, Hawkins RE, Batha H, Rothwell DG, Ashton G, Gilham DE. Eradication of established Bcell- lymphoma by Cd19specific murine T cells is dependent on host lymphopenic- environment and can be mediated by Cd4+ and Cd8+ T cells. J Immunother. 2009;32(3):207-218.

Cheson BD, Vena DA, Foss FM, Sorensen JM. Neurotoxicity of of purine analogs: a review. J Clin Oncol. 1994;12(10):2216-2228.

Chovanec M, Hanna N, Cary KC, Einhorn L, Albany C. Management of Stage I testicular germ cell tumours. Nat Rev Urol. 2016;13(11):663–673.

Colegio OR, Van Itallie CM, McCrea HJ, Rahner C, Anderson JM. Claudins create chargeselective- channels in the paracellular pathway between epithelial cells. Am J Cell Physiol. 2002;283(1):C142-147.

Cunningham D, Starling N, Rao S, Iveson T, Nicolson M, Coxon F, et al. Capecitabine and oxaliplatin for advanced esophagogastric cancer. N Engl J Med. 2008;358(1):36–46.

Daneshmand S, Albers P, Fosså SD, Heidenreich A, Kollmannsberger C, Krege S, et al. Contemporary management of postchemotherapy testis cancer. Eur Urol. 2012;62(5):867–876.

Dowdy SC. Improving oncologic outcomes for women with endometrial cancer: realigning our sights. Gynecol Oncol. 2014;133(2):370-374.

Dridi M, Chraiet N, Batti R, Ayadi M, Mokrani A, Meddeb K, et al. Granulosa cell tumor of the ovary: a retrospective study of 31 cases and a review of the literature. Int J Surg Oncol. 2018:4547892. doi: 10.1155/2018/4547892.

Dudley ME, Wunderlich JR, Yang JC, Hwu P, Schwartzentruber DJ, Topalian SL, et al. A phase I study of nonmyeloablative chemotherapy and adoptive transfer of autologous tumor antigenspecific T lymphocytes in patients with metastatic melanoma. J Immunother-. 2002;25(3):243-251.

Dudley ME, Yang JC, Sherry R, Hughes MS, Royal R, Kammula U, et al. Adoptive cell therapy for patients with metastatic melanoma: evaluation of intensive myeloablative chemoradiation preparative regimens. J Clin Oncol. 2008;26(32):5233-5239.

Durmuş Y, Kılıç Ç, Çakır C, Yüksel D, Boran N, Karalök A, et al. Sertoli-Leydig cell tumor of the ovary: analysis of a single institution database and review of the literature. J Obstet Gynaecol Res. 2019;45(7):1311-1318.

Eisenhauer EA, Therasse P, Bogaerts J, Schwartz LH, Sargent D, Ford R, et al. New response evaluation criteria in solid tumours: revised RECIST guideline (version 1.1). Eur J Cancer. 2009;45(2):228-247.

EMA/CHMP/205/95 Rev.5 (2017). Guideline on the evaluation of anticancer medicinal products in man.

EMEA/CHMP/GTWP/60436/2007 (2009). CHMP Guideline on Follow-Up of Patients administered with Gene Therapy.

Emens LA, Butterfield LH, Hodi FS, Jr, Marincola FM, Kaufman HL. Cancer immunotherapy trials: leading a paradigm shift in drug development. J Immunother Cancer. 2016;4:42. doi: 10.1186/s40425-016-0146-9.

FDA (2006). Guidance for Industry, Gene Therapy Clinical Trials-Observing Subjects for Delayed Adverse Events.

FDA (2018). Draft Guidance for Industry. Long Term Follow-Up After Administration of Human Gene Therapy Products.

FDA (2018). Guidance for Industry. Clinical Trial Endpoints for the Approval of Cancer Drugs and Biologics.

Feldman DR, Bosl GJ, Sheinfeld J, Motzer RJ. Medical treatment of advanced testicular cancer. JAMA. 2008;299(6):672–684.

Feng K, Guo Y, Dai H, Wang Y, Li X, Jia H, et al. Chimeric antigen receptor-modified T cells for the immunotherapy of patients with EGFR-expressing advanced relapsed/refractory nonsmall- cell lung cancer. Sci China Life Sci. 2016;59(5):468–479.

Ford HE, Marshall A, Bridgewater JA, Janowitz T, Coxon FY, Wadsley J, et al. Docetaxel versus active symptom control for refractory oesophagogastric adenocarcinoma (COUGAR02): an -openlabel, phase 3 randomised- controlled trial. Lancet Oncol. 2014;15(1):78–86.

Fuchs CS, Tomasek J, Yong CJ, Dumitru F, Passalacqua R, Goswami C, et al. Ramucirumab monotherapy for previously treated advanced gastric or gastro oesophageal junction adenocarcinoma (REGARD): an international, randomised, multicentre, placebocontrolled-, phase 3 trial. Lancet. 2014;383(9911):31–39.

Galon J, Bruni D. Approaches to treat immune hot, altered and cold tumours with combination immunotherapies. Nat Rev Drug Discov. 2019;18(3):197-218.

Gargett T, Yu W, Dotti G, Yvon ES, Christo SN, Hayball JD, et al. Gd2-specific CAR T cells undergo potent activation and deletion following antigen encounter but can be protected from activation-induced cell death by PD-1 blockade. Mol Ther. 2016;24(6):1135–1149.

Gatta G, Capocaccia R, Botta L, Mallone S, De Angelis R, Ardanaz E, et al. Burden and centralised treatment in Europe of rare tumours: results of RARECAREnet—a population-based study. Lancet Oncol. 2017;18(8):1022 1039.

Gershenson DM. Management of ovarian germ cell tumors. J Clin Oncol. 2007; 25(20):2938-2943.

Globocan. Cancer Fact Sheet: Corpus Uteri. Accessed at http://gco.iarc.fr/today/data/factsheets/cancers/24-Corpus-uteri-fact-sheet.pdf on 5 August 2019a.

Globocan. Cancer Fact Sheet: Stomach. Accessed at http://gco.iarc.fr/today/data/factsheets/cancers/7-Stomach-fact-sheet.pdf on 5 August 2019.

Greenwood M. The natural duration of cancer. Reports on Public Health and Medical Subjects. London: Her Majesty's Stationery Office. 1926;33:1-26.

Griffith LM, Pavletic SZ, Tyndall A, Bredesen CN, Bowen JD, Childs RW, et al. Feasibility of allogeneic hematopoietic stem cell transplantation for autoimmune disease: position statement from a National Institute of Allergy and Infectious Diseases and National Cancer Institute Sponsored- International Workshop, Bethesda, MD, March 12 and 13, 2005. Biol Blood Marrow Transplant. 2005;11(11):862-870.

Groll RJ, Warde P, Jewett MA. A comprehensive systematic review of testicular germ cell tumor surveillance. Crit Rev Oncol Hematol. 2007;64(3):182-197.

Hege KM, Bergsland EK, Fisher GA, Nemunaitis JJ, Warren RS, McArthur JG, et al. Safety, tumor trafficking and immunogenicity of chimeric antigen receptor (CAR)-T cells specific for TAG-72 in colorectal cancer. J Immunother Cancer. 2017;5:22. doi:10.1186/s40425-017-0222-9.

Herbst RS, Baas P, Kim DW, Felipe E, Pérez-Gracia JL, Han JY, et al. Pembrolizumab versus docetaxel for previously treated, Pd-L1-positive, advanced non-small-cell lung cancer (KEYNOTE-010): a randomised controlled trial. Lancet. 2016;387(10027):1540-1550.

Hewitt KJ, Agarwal R, Morin PJ. The claudin family: expression in normal and neoplastic tissues. BMC Cancer. 2006;6:186.

Hou B, Tang Y, Li W, Zeng Q, Chang D. Efficiency of CART therapy for treatment of solid tumor in clinical trials: a meta-analsysis. Hindawi- Disease Markers. 2019. Article ID 3425291. Accessed at: https://doi.org//10.1155/2019/3425291 on 20 November 2019.

Imfinzi® USPI. Accessed at http://www.azpicentral.com/pi.html?product=imfinzi&country=us&popup=no on 5 August 2019.

Jelovac D, Armstrong DK. Recent progress in the diagnosis and treatment of ovarian cancer. CA Cancer J Clin. 2011;61(3):183-203.

Jones WG, Fossa SD, Mead GM, et al. Randomized trial of 30 versus 20 Gy in the adjuvant treatment of Stage I testicular seminoma: a report on Medical Research Council trial TE18, European Organisation for the Research and Treatment of Cancer Trial 30942 (ISRCTN18525328). J Clin Oncol. 2005;23(6):1200–1208.

Junghans RP, Ma Q, Rathore R, Gomes EM, Bais AJ, Lo AS, et al. Phase I trial of anti-PSMA designer CAR-T cells in prostate cancer: possible role for interacting Interleukin 2-T cell pharmacodynamics as a determinant of clinical response. Prostate. 2016;76(6): 1257–1270.

Kalos M, Levine BL, Porter DL, Katz S, Grupp SA, Bagg A, et al., T cells with chimeric antigen receptors have potent antitumor effects and can establish memory in patients with advanced leukemia. Sci Transl Med. 2011;3(95):95ra73.

Kang YK, Boku N, Satoh T, Ryu MH, Chao Y, Kato K, et al. Nivolumab in patients with advanced gastric or gastro-oesophageal junction cancer refractory to, or intolerant of, at least two previous chemotherapy regimens (ONO-4538-12, ATTRACTION-2): a randomised, double-blind, placebo-controlled, phase 3 trial. Lancet. 2017;390(10111):2461-2471. doi: 10.1016/S0140-6736(17)31827-5.

Katz SC, Burga RA, McCormack E, Wang LJ, Mooring W, Point GR, et al. Phase I hepatic immunotherapy for metastases study of intra-arterial chimeric antigen receptor-modified T-cell therapy for CEA+ liver metastases. Clin Cancer Res. 2015;21(14):3149-3159.

Kershaw MH, Jackson JT, Haynes NM, Teng MW, Moeller M, Hayakawa Y, et al. Gene engineered T cells as a superior adjuvant therapy for metastatic cancer. J Immunol. 2004;173(3):2143-2150.

Kershaw MH, Westwood JA, Parker LL, Wang G, Eshhar S, Mavroukakis SA, et al. A phase I study on adoptive immunotherapy using gene-modified T cells for ovarian cancer. Clin Cancer Res. 2006;12(20 Pt 1):6106-6115.

Keytruda® USPI. Accessed at http://www.merck.com/product/usa/pi_circulars/k/keytruda/keytruda_pi.pdf on 5 August 2019.

Klebanoff CA, Khong HT, Antony PA, Palmer DC, Restifo NP. Sinks, suppressors and antigen presenters: how lymphodepletion enhances T cellmediated- tumor immunotherapy. Trends Immunol. 2005;26(2):111-117.

Kochenderfer JN, Wilson WH, Janik JE, Dudley ME, StetlerStevenson- M, Feldman SA. Eradication of Blineage- cells and regression of lymphoma in a patient treated with autologous T cells genetically engineered to recognize Cd19. Blood. 2010;116(20):4099-4102.

Koizumi W, Narahara H, Hara T, Takagane A, Akiya T, Tagaki M, et al. S-1 plus cisplatin versus S-1 alone for first line treatment of advanced gastric cancer (SPIRITS trial): a phase III trial. Lancet Oncol. 2008;9(3):215–221.

Kollmannsberger C, Tandstad T, Bedard PL, Cohn-Cedermark G, Chung PW, Jewett MA, et al. Patterns of relapse in patients with clinical Stage I testicular cancer managed with active surveillance. J Clin Oncol. 2015;33(1):51–57.

Krege S, Beyer J, Souchon R, et al. European consensus conference on diagnosis and treatment of germ cell cancer: a report of the second meeting of the European Germ Cell Cancer Consensus group (EGCCCG): part I. Eur Urol. 2008;53(3):478-496.

Lamers CH, Sleijfer S, van Steenbergen S, van Elzakker P, van Krimpen B, Groot C, et al. Treatment of metastatic renal cell carcinoma with CAIX CAR-engineered T cells: clinical evaluation and management of on-target toxicity. Mol Ther. 2013;21(4):904-912.

Ledermann JA, Raja FA, Fotopoulou C, Gonzalez-Martin A, Colombo N, Sess C, et al. Newly diagnosed and relapsed epithelial ovarian carcinoma: ESMO Clinical Practice Guidelines for diagnosis, treatment and follow-up. Ann Oncol. 2013;24 Suppl 6:vi24-32.

Lee DW, Santomasso BD, Locke FL, Ghobadi A, Turtle CJ, Brudno JN, et al. ASTCT consensus for grading cytokine release syndrome and neurologic toxicity associated with immune effector cells. Biol Blood Marrow Transplant. 2019;25(4):625-638.

Levey AS, Coresh J, Greene T, Marsh J, Stevens LA, Kusek JW, Van Lente F; Chronic Kidney Disease Epidemiology Collaboration. Expressing the Modification of Diet in Renal Disease Study equation for estimating glomerular filtration rate with standardized serum creatinine values. Clin Chem. 2007 Apr;53(4):766-72.

Li J, Qin S, Xu J, Xiong J, Wu C, Bai Y, et al. Randomized, doubleblind, -placebocontrolled phase III trial of apatinib in patients with -chemotherapyrefractory- advanced or metastatic adenocarcinoma of the stomach or gastroesophageal junction. J Clin Oncol. 2016;34(13):1448–1454.

Locke FL, Ghobadi A, Jacobson CA, Miklos DB, Lekakis LJ, Oluwole OO, et al. Long-term safety and activity of axicabtagene ciloleucel in refractory large B-cell lymphoma (ZUMA-1): a single-arm, multicentre, phase 1-2 trial. Lancet Oncol. 2019 Jan;20(1):31-42.

Louis CU, Savoldo B, Dotti G, Pule M, Yvon E, Myers GD, et al. Antitumor activity and longterm fate of chimeric antigen -receptorpositive- T cells in patients with neuroblastoma. Blood. 2011;118(23):6050-6056.

Luchini C, Bibeau F, Ligtenberg MJL, Singh N, Nottegar A, Bosse T, et al. ESMO recommendations on microsatellite instability testing for immunotherapy in cancer, and its

relationship with Pd-1/Pd-L1 expression and tumour mutational burden: a systematic review-based approach. Ann Oncol. 2019;pii: mdz116. doi: 10.1093/annonc/mdz116.

Lynparza® USPI, accessed at https://www.azpicentral.om/pi.html?product=lynparza_tb&country=us&popup=no on 5 August 2019.

Mackall C, Tap WD, Glod J, Druta M, Chow WA, Araujo DM, et al. Open label, non-randomized, multi-cohort pilot study of genetically engineered NY-ESO-1c259T in HL-A2+ patients with synovial sarcoma (NCT01343043). J Clin Oncol. 2017;35(15 suppl):3000.

Majzner RG, Mackall CL. Clinical lessons learned from the first leg of the CAR T cell journey. Nat Medicine. 2019;25(9):1341-1355.

Mangili G, Sigismondi C, Gadducci A, Cormio G, Scollo P, Tateo S, et al. Outcome and risk factors for recurrence in malignant ovarian germ cell tumors: a MITO-9 retrospective study. Int J Gynecol Cancer. 2011;21(8):1414-1421.

Matsubara A, Miyashita T, Mori T, Akiyama K, Inamoto R, Mori N. The mRNA of claudins is expressed in the endolymphatic sac epithelia. Auris Nasus Larynx. 2012;39(4):361-364.

Maude SL, Laetsch TW, Buechner J, Rives S, Boyer M, Bittencourt H. Tisagenlecleucel in children and young adults with B-cell lymphoblastic leukemia. N Eng J Med. 2018;378(5);439-448.

Maus MV, Haas AR, Beatty GL, Albelda SM, Levine BL, Liu X, et al. T cells expressing chimeric antigen receptors can cause anaphylaxis in humans. Cancer Immunol Res. 2013;1(1): 26–31.

Mendivil A, Schuler KM, Gehrig PA. Non-endometrioid adenocarcinoma of the uterine corpus: a review of selected histological subtypes. Cancer Control. 2009;16(1):46-52.

Micke P, Mattsson JS, Edlund K, Lohr M, Jirström K, Berglund A, et al. Aberrantly activated claudin 6 and 18.2 as potential therapy targets in non-small-cell lung cancer. Int J Cancer. 2014;135(9):2206-2214.

Mittica G, Genta S, Aglietta M and Valbrega G. Immune checkpoint inhibitors: a new opportunity in the treatment of ovarian cancer? Int J Mol Sci. 2016;17(7). pii: E1169. doi:10.3390/ijms17071169.

Miura Y, Sunaga N. Role of immunotherapy for oncogene-driven non-small cell lung cancer. Cancers (Basel). 2018;10(8). pii: E245. doi: 10.3390/cancers10080245.

Morgan RA, Yang JC, Kitano M, Dudley ME, Laurencot CM, Rosenberg SA. Case report of a serious adverse event following the administration of T cells transduced with a chimeric antigen receptor recognising ERBB2. Mol Ther. 2010;18(4):843-851.

Morita K, Furuse M, Fujimoto K, Tsukita S. Claudin multigene family encoding four transmembrane domain protein components of tight junction strands. Proc Natl Acad Sci USA. 1999;96:511-516.

Motzer RJ, Nichols CJ, Margolin KA, Bacik J, Richardson PG, Vogelzang LJ, et al. Phase III randomized trial of conventional-dose chemotherapy with or without high-dose chemotherapy and autologous hematopoietic stem-cell rescue as first-line treatment for patients with poorprognosis- metastatic germ cell tumors. J Clin Oncol. 2007;25(3):247-256.

NCCN 2019a. NCCN Clinical Practice Guidelines in Oncology (NCCN Guidelines®). Testicular Cancer. Accessed at https://www.nccn.org/professionals/physician_gls/pdf/testicular.pdf on 5 August 2019.

NCCN 2019b. NCCN Clinical Practice Guidelines in Oncology (NCCN Guidelines®). Ovarian Cancer. Accessed at https://www.nccn.org/professionals/physician_gls/pdf/ovarian.pdf on 5 August 2019.

NCCN 2019c. NCCN Clinical Practice Guidelines in Oncology (NCCN Guidelines®). Uterine Neoplasms. Accessed at https://www.nccn.org/professionals/physician_gls/pdf/uterine.pdf on 5 August 2019.

NCI PDQ 2019a. PDQ® Adult Treatment Editorial Board. PDQ Testicular Cancer Treatment. Bethesda, MD: National Cancer Institute. Updated 5 August 2019. Available at: https://www.cancer.gov/types/testicular/hp/testicular-treatment-pdq. Accessed 5 August 2019. [PMID: 26389220]

NCI PDQ 2019b. PDQ® Adult Treatment Editorial Board. PDQ Ovarian Epithelial, Fallopian Tube, and Primary Peritoneal Cancer Treatment. Bethesda, MD: National Cancer Institute. Updated 5 August 2019. Available at: https://www.cancer.gov/types/ovarian/hp/ovarian-epithelial-treatment-pdq. Accessed 5 August 2019. [PMID: 26389443]

NCI 2019. About Rare Cancers. Accessed at https://www.cancer.gov/pediatric-adult-rare-tumor/rare-tumors/about-rare-cancers on 5 August 2019.

Neelapu SS, Locke FL, Bartlett NL, Lekakis LJ, Miklos DB, Jacobson CA, et al. Axicabtagene Ciloleucel CAR T cell therapy in refractory large B-cell lymphoma. N Eng- J Med. 2017;377(26):2531-2544.

Neelapu SS, Tummala S, Kebriaei P, Wierda W, Gutierrez C, Locke FL, et al. Chimeric antigen receptor T-cell therapy – assessment and management of toxicities. Nat Rev Clin Oncol. 2018;15(1):47-62.

Neelapu SS. Managing the toxicities of CAR T cell therapy. Hematol. Oncol. 2019;37 Suppl 1:48-52.

Neill M, Warde P, Fleshner N. Management of low-stage testicular seminoma. Urol Clin North Am. 2007;34(2):127-136.

Oldenburg J, Fosså SD, Nuver J, Heidenreich A, Schmoll HJ, Bokemeyer C, et al. Testicular seminoma and non-seminoma: ESMO clinical practice guidelines for diagnosis, treatment and follow-up. Ann Oncol. 2013;24 (suppl 6):vi125-32. doi: 10.1093/annonc/mdt304

Oliver RT, Mead GM, Rustin GJ, Joffe JK, Aass N, Coleman R, et al. Randomized trial of carboplatin versus radiotherapy for Stage I seminoma: mature results on relapse and contralateral testis cancer rates in MRC TE19/EORTC 30982 study (ISRCTN27163214). J Clin Oncol. 2011;29(8):957–962.

Opdivo® USPI. Accessed at https://packageinserts.bms.com/pi/pi_opdivo.pdf on 5 August 2019.

O'Rourke DM, Nasrallah MP, Desai A, Melenhorst JJ, Mansfield K, Morrissette JJD, et al. A single dose of peripherally infused EGFRvIII-directed CAR T cells mediates antigen loss and induces adaptive resistance in patients with recurrent glioblastoma. Sci Transl Med. 2017;9(399). pii: eaaa0984. doi:10.1126/scitranslmed.aaa0984.

Ott PA, Bang YJ, Berton-Rigaud D, Elez E, Pishvaian MJ, Rugo HS, et al. Safety and antitumor activity of pembrolizumab in advanced programmed death ligand 1-positive endometrial cancer: results from the KEYNOTE-028 study. J Clin Oncol. 2017;35(22):2535-41.

Pal SK, Miller MJ, Agarwal N, Chang SM, Chavez-MacGregor M, Cohen E, et al. Clinical cancer advances 2019: annual report on progress against cancer from the American Society of Clinical Oncology. J Clin Oncol. 2019;37(10):834-849.

Park JR, DiGiusto DL, Slovak M, Wright C, Naranjo A, Wagner J, et al. Adoptive transfer of chimeric antigen receptor redirected cytolytic T lymphocyte clones in patients with neuroblastoma. Mol Ther-. 2007;15(4):825-833.

Porter DL, Hwang WT, Frey NV, Lacey SF, Shaw PA, Loren AW, et al. Chimeric antigen receptor T cells persist and induce sustained remissions in relapsed refractory chronic lymphocytic leukemia. Sci Transl Med. 2015;7(303):303ra139. doi:10.1126/scitranslmed.aac5415.

Prat J. New insights into ovarian cancer pathology. Ann Oncol. 2012;23 Suppl 10:x111-117.

Rajpert-De Meyts E, McGlynn KA, Okamoto K, Jewett MA, Bokemeyer C. Testicular germ cell tumours. Lancet. 2016;387(10029):1762–1774.

Rendón-Huerta E, Teresa F, Teresa GM, Xochitl GS, Georgina AF, Veronica ZZ, et al. Distribution and expression pattern of claudins 6, 7, and 9 in diffuse- and intestinal-type gastric adenocarcinomas. J Gastrointest Cancer. 2010;41(1):52-59.

Ries LAG, Melbert D, Krapcho M, et al. SEER Cancer Statistics Review, 1975-2005. Bethesda, MD: National Cancer Institute, 2007. Also available online at https://seer.cancer.gov/archive/csr/1975_2005/, based on November 2007 SEER data submission, posted to SEER web site, 2008. Last accessed November 11, 2019.

Robbins PF, Kassim SH, Tran TL, Crystal JS, Morgan RA, Feldman SA. A pilot trial using lymphocytes genetically engineered with an NY-ESO-1-reactive T-cell receptor: long-term follow-up and correlates with response. Clin Cancer Res. 2015;21(5):1019-1027.

Rubraca® USPI accessed at https://www.rubracahcp.com/?utm_source=google&utm_medium=cpc&utm_term=rucaparib&utm_campaign=Branded_Alone_GS_EX on 5 August 2019.

Schuster SJ, Bishop MR, Tam CS, Waller EK, Borchmann P, McGuirk JP, et al; JULIET Investigators. Tisagenlecleucel in Adult Relapsed or Refractory Diffuse Large B-Cell Lymphoma. N Engl J Med. 2019 Jan 3;380(1):45-56.

Scott BL, Sandmaier BM, Storer B, Maris MB, Sorror mL, Maloney DG, et al. Myeloablative vs nonmyeloablative allogeneic transplantation for patients with myelodysplastic syndrome or acute myelogenous leukemia with multilineage dysplasia: a retrospective analysis. Leukemia. 2006;20(1):128-135.

SEER. Cancer Stat Facts: Stomach Cancer. Accessed at https://seer.cancer.gov/statfacts/html/stomach.html on 5 August 2019.

Seymour L, Bogaerts J, Perrone A, Ford R, Schwartz LH, Mandrekar S, et al. iRECIST: guidelines for response criteria for use in trials testing immunotherapeutics. Lancet Oncol. 2017;18(3):e143-e152.

Sheinfeld J, Motzer RJ, Rabbani F, McKiernan J, Bajorin D, Bosl GJ, et al. Incidence and clinical outcome of patients with teratoma in the retroperitoneum following primary retroperitoneal lymph node dissection for clinical stages I and IIA nonseminomatous germ cell tumors. J Urol. 2003;170(4 Pt 1):1159-1162.

Shin DW, Cho J, Yang HK, Kim SY, Lee SH, Suh B, et al. Oncologist perspectives on rare cancer care: a nationwide survey. Cancer Res Treat. 2015;47(4):591–599.

Smith ZL, Werntz RP and Eggener SE. Testicular cancer: epidemiology, diagnosis and management. Med Clin North Am. 2018;102(2):251-264.

Sorror ML, Maris MB, Storer B, Sandmaier BM, Diaconescu R, Flowers C, et al. Comparing morbidity and mortality of HLA-matched unrelated donor hematopoietic cell transplantation after nonmyeloablative and myeloablative conditioning: influence of pretransplantation comorbidities. Blood. 2004;104(4):961-968.

Sullivan LM, Yankovich T, Le P, Martinez D, Santi M, Biegel JA, et al. Claudin6 is a nonspecific marker for malignant rhabdoid and other pediatric tumors. Am J Surg Pathol-. 2012;36(1):73-80.

Tandstad T, Dahl O, Cohn-Cedermark G, Cavallin-Stahl E, Steirner U, Solberg A, et al. Riskadapted treatment in clinical Stage I non-seminomatous germ cell testicular cancer: the SWENOTECA management program. J Clin Oncol. 2009;27(13):2122-2128.

Tanyi JL, Stashwick C, Plesa G, Morgan MA, Porter D, Maus MV, et al. Possible compartmental cytokine release syndrome in a patient with recurrent ovarian cancer after treatment with mesothelin-targeted CAR-T cells. J Immunother. 2017;40(3):104–107.

Tao X, Sood AK, Deavers MT, Schmeler KM, Nick AM, Coleman RL, et al. Anti-angiogenesis therapy with bevacizumab for patients with ovarian granulosa cell tumors. Gynecol Oncol. 2009;114(3):431-436.

Tchou J, Zhao Y, Levine BL, Zhang PJ, Davis MM, Melenhorst JJ, et al. Safety and efficacy of intratumoral injections of chimeric antigen receptor (CAR) T cells in metastatic breast cancer. Cancer Immunol Res. 2017;5(12):1152–1161.

Tecentriq® USPI. Accessed at https://www.gene.com/download/pdf/tecentriq_prescribing.pdf on 5 August 2019.

Teoh D, Freedman R, Soliman PT. Nearly 30 years of treatment for recurrent granulosa cell tumor of the ovary: a case report and review of the literature. Case Rep Oncol. 2010;3(1):14-18.

Thuss-Patience PC, Kretzschmar A, Bichev D, Deist T, Hinke A, Breithaupt K, et al. Survival advantage for irinotecan versus best supportive care as second-line chemotherapy in gastric cancer–a randomized phase III study of the Arbeitsgemeinschaft Internistische Onkologie (AIO). Eur J Cancer. 2011;47(15):2306-2314.

Turksen K, Troy TC. Barriers built on claudins. J Cell Sci. 2004;117(Pt12):2435-2447.

Turksen K, Troy TC. Claudin-6: a novel tight junction molecule is developmentally regulated in mouse embryonic epithelium. Dev Dyn. 2001;222(2):292-300.

Turtle CJ, Hanafi LA, Berger C, Gooley TA, Cherian S, Hudecek M, et al. Cd19 CAR-T cells of define Cd4+:Cd8+ composition in adult B cell ALL patients. J Clin Invest. 2016b;126(6):2123-2138.

Turtle CJ, Hanafi LA, Berger C, Hudecek M, Pender B, Robinson E, et al. Immunotherapy of non-Hodgkin's lymphoma with a defined ratio of Cd8+ and Cd4+ C19specific chimeric antigen receptor-modified cells. Sci Transl- Med. 2016a;8(355):355ra116.

Ushiku T, Shinozaki-Ushiku A, Maeda D, Fukayama M. Distinct expression pattern of claudin6, a primitive phenotypic tight junction molecule in germ cell tumours- and visceral carcinomas. 2012;61(6):1043-1056.

Van Cutsem E, Moiseyenko VM, Tjulandin S, Majlis A, Constenla M, Boni C, et al. Phase III study of docetaxel and cisplatin plus fluorouracil compared with cisplatin and fluorouracil as first-line therapy for advanced gastric cancer: a report of the V325 study group. J Clin Oncol. 2006;24(31):4991-4997.

van Dijk MR, Steyerberg EW, Habbema JD. Survival of non-seminomatous germ cell cancer patients according to the IGCC classification: an update based on meta-analysis. Eur J Cancer. 2006;42(7):820–826.

Vitrakvi® USPI. Accessed at http://labeling.bayerhealthcare.com/html/products/pi/vitrakvi_PI.pdf on 5 August 2019.

Wilke H, Muro K, Van Cutsem E, Oh SC, Bodoky G, Shimada Y, et al. Ramucirumab plus paclitaxel versus placebo plus paclitaxel in patients with previously treated advanced gastric or gastrooesophageal junction adenocarcinoma (RAINBOW): a -doubleblind-, randomized phase 3 trial. Lancet Oncol. 2014;15(11):1224–1235.

Yong CSM, Dardalhon V, Devaud C, Taylor N, Darcy PK, Kershaw MH. CAR-T-cell therapy of solid tumors. Immunol Cell Biol. 2017;95(4):356-363.

You F, Jiang L, Zhang B, Lu Q, Zhou Q, Liao X, et al. phase I clinical trial demonstrated that MUC1 positive metastatic seminal vesicle cancer can be effectively eradicated by modified Anti-MUC1 chimeric antigen receptor transduced T cells. Sci China Life Sci. 2016;59(4):386–397.

Zhang C, Wang Z, Yang Z, Wang M, Li S, Li Y, et al. Phase I escalating-dose trial of CAR-T therapy targeting CEA^+ metastatic colorectal cancers. Mol Ther. 2017;25(5):1248–1258.

Zhang T, Cao L, Xie J, Shi N, Zhang Z, Luo Z, et al. Efficiency of Cd19 chimeric antigen receptor-modified T cells for treatment of B-cell malignancies in phase I clinical trials: a metaanalysis. Oncotarget. 2015;6(32):33961-33971.

12 SUPPORTING DOCUMENTATION AND OPERATIONAL CONSIDERATIONS (APPENDICES)

12.1 APPENDIX 1: REGULATORY, ETHICAL, AND TRIAL OVERSIGHT CONSIDERATIONS

12.1.1 Regulatory and ethical considerations

- This trial will be conducted in accordance with the protocol and with the following:
  - Consensus ethical principles derived from international guidelines including the Declaration of Helsinki and Council for International Organizations of Medical Sciences International Ethical Guidelines
  - Applicable ICH GCP Guidelines
  - Applicable laws and regulations
- The protocol, protocol amendments, ICFs, IB, and other relevant documents (e.g., advertisements) must be submitted to an IRB/IEC by the investigator and reviewed and approved by the IRB/IEC before the trial is initiated.
- Any amendments to the protocol will require IRB/IEC approval before implementation of changes made to the trial design, except for changes necessary to eliminate an immediate hazard to trial patients.
- The investigator will be responsible for the following:
  - Providing written summaries of the status of the trial to the IRB/IEC annually or more frequently in accordance with the requirements, policies, and procedures established by the IRB/IEC
  - Notifying the IRB/IEC of SAEs or other significant safety findings as required by IRB/IEC procedures
  - Providing oversight of the conduct of the trial at the site and adherence to requirements of 21 CFR, ICH guidelines, the IRB/IEC, European regulation 536/2014 for clinical studies (if applicable), and all other applicable local regulations

12.1.2 Financial disclosure

Investigators and sub-investigators will provide the sponsor with sufficient, accurate financial information as requested to allow the sponsor to submit complete and accurate financial certification or disclosure statements to the appropriate regulatory authorities. Investigators are responsible for providing information on financial interests during the course of the trial and for 1 year after completion of the trial.

12.1.3 Informed consent process

The investigator or his/her representative will explain the nature of the trial to the patient or his/her legally authorized representative and answer all questions regarding the trial. Patients

must be informed that their participation is voluntary. Patients or their legally authorized representative will be required to sign a statement of informed consent that meets the requirements of 21 CFR 50, local regulations, ICH guidelines, Health Insurance Portability and Accountability Act requirements, where applicable, and the IRB/IEC or trial center.

The medical record must include a statement that written informed consent was obtained before the patient was enrolled in the trial and the date the written consent was obtained. The authorized person obtaining the informed consent must also sign the ICF. Patients must be re-consented to the most current version of the ICF(s) during their participation in the trial, in accordance with the requirements, policies, and procedures established by the IRB/IEC. A copy of the ICF(s) must be provided to the patient or the patient's legally authorized representative.

Patients who are re-screened are required to sign a new ICF.

12.1.4 Data protection

Patients will be assigned a unique identifier by the sponsor. Any patient records or datasets that are transferred to the sponsor will contain the identifier only; patient names or any information which would make the patient identifiable will not be transferred.

The patient must be informed that his/her personal trial-related data will be used by the sponsor in accordance with local data protection law. The level of disclosure must also be explained to the patient who will be required to give consent for their data to be used as described in the informed consent.

The patient must be informed that his/her medical records may be examined by Clinical Quality Assurance auditors or other authorized personnel appointed by the sponsor, by appropriate IRB/IEC members, and by inspectors from regulatory authorities.

In case that bodily materials and/or personal data will be transferred to third countries outside the EU, which do not have the same data protection standard as in Europe and in the absence of an EU adequacy decision for such third country, the sponsor of the clinical trial will establish appropriate safeguards that ensure that the recipient will process the personal data in compliance with Art. 46 of the European General Data Protection Regulation (GDPR). By default, the sponsor will ensure GDPR compliance and an equivalent level of protection of personal data in such cases by employing Standard Contractual Clauses adopted by the European Commission as an appropriate safeguard.

12.1.5 Committees structure

An SRC will be established to review the interim efficacy and safety results. The SRC will act according to its own written standard operating procedure described in a charter, and will prepare written minutes of its meetings.

12.1.6 Data quality assurance

- All patient data relating to the trial will be recorded on eCRF unless transmitted to the sponsor or designee electronically (e.g., laboratory data). The investigator is responsible for verifying that data entries are accurate and correct by electronically signing the eCRF and comply with country-specific regulations.
- The investigator must maintain accurate documentation (source data) that supports the information entered in the eCRF.
- The investigator must permit trial-related monitoring, audits, IRB/IEC review, and regulatory agency inspections and provide direct access to source data documents.
- Monitoring details describing strategy (e.g., risk-based initiatives in operations and quality such as Risk Management and Mitigation Strategies and Analytical Risk-Based Monitoring), methods, responsibilities and requirements, including handling of non-compliance issues and monitoring techniques (central, remote, or on-site monitoring) are provided in the Monitoring Plan.
- The sponsor or designee is responsible for the data management of this trial including quality checking of the data.
- The sponsor assumes accountability for actions delegated to other parties (e.g., CROs).
- Trial monitors will perform ongoing source data verification to confirm that data entered into the eCRF by authorized site personnel are accurate, complete, and verifiable from source documents; that the safety and rights of patients are being protected; and that the trial is being conducted in accordance with the currently approved protocol and any other trial agreements, ICH GCP, and all applicable regulatory requirements.
- Records and documents, including signed ICFs, pertaining to the conduct of this trial must be retained by the investigator for 2 years after trial completion unless local regulations or institutional policies require a longer retention period. No records may be destroyed during the retention period without the written approval of the sponsor. No records may be transferred to another location or party without written notification to the sponsor.

12.1.7 Source documents

Source documents provide evidence for the existence of the patient and substantiate the integrity of the data collected. Source documents are filed at the investigator's site.

Data reported on the CRF or entered in the eCRF that are transcribed from source documents must be consistent with the source documents or the discrepancies must be explained. The investigator may need to request previous medical records or transfer records, depending on the trial. In addition, current medical records must be available.

Definition of what constitutes source data can be found in the Investigator File.

12.1.8 Trial and site start and closure

The trial start date is the date on which the clinical trial will be open for recruitment of patients.

The first act of recruitment is the first site open and will be the trial start date.

The sponsor/designee reserves the right to close the trial site or terminate the trial at any time for any reason at the sole discretion of the sponsor. Trial sites will be closed upon trial completion. A trial site is considered closed when all required documents and trial supplies have been collected and a trial site closure visit has been performed.

The investigator may initiate trial site closure at any time, provided there is reasonable cause and sufficient notice is given in advance of the intended termination.

Reasons for the early closure of a trial site by the Sponsor or Investigator may include but are not limited to:

- Failure of the investigator to comply with the protocol, the requirements of the IRB/IEC or local health authorities, the sponsor's procedures, or GCP guidelines
- Inadequate recruitment of patients by the investigator
- Discontinuation of further trial treatment development

If the trial is prematurely terminated or suspended, the sponsor shall promptly inform the investigators, the IECs/IRBs, the regulatory authorities, and any CRO(s) used in the trial of the reason for termination or suspension, as specified by the applicable regulatory requirements. The investigator shall promptly inform the patient and should assure appropriate patient therapy and/or follow-up.

12.1.9 Publication policy

The results of this trial will be publically disclosed in accordance with the sponsor's disclosure policy and applicable regulations (e.g., on ClinicalTrials.gov).

The results of this trial will be submitted for publication, e.g., as a full publication or at a congress (e.g., as a poster or presentation). The sponsor reserves the right to review any proposed full publication or poster or presentation of the results of this trial by the Coordinating Investigator before they are submitted for publication or public disclosure.

In accordance with standard editorial and ethical practice, the sponsor will generally support publication of multicenter studies only in their entirety and not as individual site data. In this case, a Coordinating Investigator will be designated by mutual agreement.

Authorship will be determined by mutual agreement and in line with the sponsor's publication policy, which follows the International Committee of Medical Journal Editors authorship requirements.

Neither the sponsor nor the Coordinating Investigator has the right to prohibit publication or public disclosure unless it can be shown to affect possible patent rights.

12.1.10 Trial results reporting

A final report integrating clinical, PK, pharmacodynamic and statistical results will be prepared by the sponsor. The Coordinating Investigator will approve the final report on behalf of the participating investigators.

12.2 APPENDIX 2: CLINICAL LABORATORY TESTS

The tests detailed in Table 22 will be performed by the central laboratory with the exception of urinalysis, which will be performed by the local laboratory. Protocol-specific requirements for inclusion or exclusion of patients are detailed in Section 5 of the protocol. Additional tests may be performed at any time during the trial as determined necessary by the investigator or required by local regulations. Pregnancy testing will be performed locally.

Table 22: Protocol-required safety laboratory assessments

Laboratory assessments	Parameters			
**Hematology**	Platelet Count<br>RBC Count<br>Hemoglobin<br>Hematocrit	RBC Indices:<br>MCV<br>MCH<br>%Reticulocytes	WBC count with Differential:<br>Neutrophils<br>Lymphocytes<br>Monocytes<br>Eosinophils<br>Basophils	
**Clinical chemistry**	BUN	Potassium	AST/SGOT	Total and direct bilirubin
	Creatinine	Sodium	ALT/SGPT	Phosphate
	LDH	Calcium	Alkaline phosphatase	Uric acid
	Lipase	Amylase	GGT	GLDH
**Coagulation**	INR			
**Endocrinology**	TSH	T4	T3	
**Tests of special interest for CRS and ICANS**	IL-6,	CRP	Ferritin	Fibrinogen
	Procalcitonin			
	Hematology (as above)		Clinical chemistry (as above)	
**Routine urinalysis**	• Specific gravity<br>• pH, glucose, protein, blood, ketones, bilirubin, urobilinogen, nitrite, leukocyte esterase by dipstick<br>• Microscopic examination (if blood or protein is abnormal)			
**Other screening tests**	• Follicle-stimulating hormone and estradiol (as needed in women of non-childbearing potential only)<br>• Serum pregnancy test (as needed for women of childbearing potential)<br>• Serology/virology (HIV antibody, hepatitis B surface antigen [HBsAg], and hepatitis C virus [HCV] antibody)<br>All trial-required laboratory assessments will be performed by a central laboratory, with the exception of urinalysis. The results of each test performed by the local laboratory will be entered into the eCRF.			

Abbreviations: ALT, alanine aminotransferase; AST, aspartate aminotransferase; BUN, blood urea nitrogen; CRP, C-reactive protein; eCRF, electronic case report form; GGT, gamma-glutamyl transferase; GLDH, Glutamate dehydrogenase; HIV, human immunodeficiency virus; ICANS, immune effector cell-associated neurotoxicity syndrome; IL-6, interleukin 6; LDH, lactate dehydrogenase; MCH, mean corpuscular hemoglobin; MCV, mean corpuscular volume; RBC, red blood cell; SGOT, serum glutamic-oxaloacetic transaminase; SGPT, serum glutamic pyruvic transaminase; sIL-2R, soluble interleukin 2 receptor; T3, triiodothyronine; T4, thyroxine; TSH, thyroid-stimulating hormone; WBC, white blood cell.

Investigators must document their review of each laboratory safety report.

The biomarker assays detailed in Table 23 will be performed by the central laboratory.

Table 23: Biomarker assays to monitor CAR-T mediated effects

Laboratory assessment	Parameter
**Testicular tumor marker assessment**	Alpha-fetoprotein (AFP)
	Lactate dehydrogenase (LDH)
	Human chorionic gonadotropin (hCG)
**Endometrial and ovarian tumors**	Cancer antigen (CA)-125
**Gastric tumors**	AFP, carcinoembryonic antigen (CEA), CA72-4, CA19-9, CA-125
**NSCLC-specific**	CEA, CA-125
**Anti-CAR humoral response**	CAR immunogenicity assay
**Cytokine analysis**	Cytokine multiplex assay (e.g., TNF, IFN-α, IFN-γ, IL-2, IL-10, soluble IL-2Rα, IP-10, IL-12, IL-6, soluble IL-6R)
**CAR-T expansion**	Flow cytometry
	qPCR

Abbreviations not defined in table: IFN-α, interferon; IL, interleukin; IP-10, interferon-gamma induced protein 10 kD, (CXCL10); TNF, tumor necrosis factor.

12.3 APPENDIX 3: ADVERSE EVENTS: DEFINITIONS AND PROCEDURES FOR RECORDING, EVALUATING, FOLLOW-UP, AND REPORTING

12.3.1 Definition of AE

12.3.1.1 AE definition

- An AE is any untoward medical occurrence in a patient or clinical trial patient, administered a pharmaceutical product and which does not necessarily have to have a causal relationship with this treatment.
- NOTE: An AE can therefore be any unfavorable and unintended sign (including an abnormal laboratory finding), symptom, or disease (new or exacerbated) temporally associated with the use of a medicinal product, whether or not considered related to the medicinal product.

12.3.1.2 Events MEETING the AE definition

- Any abnormal laboratory test results (hematology, clinical chemistry, or urinalysis) or other safety assessments (e.g., ECG, radiological scans, vital signs measurements), including those that worsen from baseline, considered clinically significant in the medical and scientific judgment of the investigator.
- Exacerbation of a chronic or intermittent pre-existing condition including either an increase in frequency and/or intensity of the condition.
- New conditions or worsening of pre-existing conditions detected or diagnosed after signing the main ICF.
- Signs, symptoms, or the clinical sequelae of a suspected drug-drug interaction.
- Signs, symptoms, or the clinical sequelae of a suspected overdose of either trial treatment or a concomitant therapy. Overdose per se will not be reported as an AE/SAE.

12.3.1.3 Events NOT MEETING the AE definition

- Any clinically significant abnormal laboratory findings or other abnormal safety assessments that are associated with the underlying disease, unless judged by the investigator to be more severe than expected for the patient's condition.
- The disease/disorder being studied or expected progression, signs, or symptoms of the disease/disorder being studied, unless more severe than expected for the patient's condition.
- Medical or surgical procedure (e.g., endoscopy, appendectomy): The condition that leads to the procedure is the AE.
- Situations in which an untoward medical occurrence did not occur (social and/or convenience admission to a hospital).

- Anticipated day-to-day fluctuations of pre-existing disease(s) or condition(s) present or detected at the start of the trial that do not worsen.
- Disease-related events and/or disease-related outcomes not qualifying as AEs or SAEs are further specified in Section 8.3.6. The progression of underlying disease (e.g., new metastases) during trial participation is not considered an AE. However, specific symptoms at time of progression that may be considered as being caused by reasons other than the progression of underlying disease itself, and fatal cases where a cause other than the disease progression itself may not be discounted, will have to be documented as AEs and reported as SAEs if applicable.

12.3.2 Definition of SAE and suspected unexpected serious adverse reaction (SUSAR)

If an event is not an AE per definition above, then it cannot be an SAE even if serious conditions are met.

An SAE is defined as any untoward medical occurrence that, at any dose:

a. Results in death

b. Is life-threatening

The term 'life-threatening' in the definition of 'serious' refers to an event in which the patient was at risk of death at the time of the event. It does not refer to an event, which hypothetically might have caused death, if it were more severe.

c. Requires in-patient hospitalization or prolongation of existing hospitalization

- In general, hospitalization signifies that the patient has been detained (usually involving at least an overnight stay) at the hospital or emergency ward for observation and/or treatment that would not have been appropriate in the physician's office or outpatient setting. Complications that occur during hospitalization are AEs. If a complication prolongs hospitalization or fulfills any other serious criteria, the event is serious. When in doubt as to whether "hospitalization" occurred or was necessary, the AE should be considered serious.
- Hospitalization for elective treatment of a pre-existing condition that did not worsen from baseline is not considered an AE.

d. Results in persistent disability/incapacity

- The term disability means a substantial disruption of a person's ability to conduct normal life functions.
- This definition is not intended to include experiences of relatively minor medical significance such as uncomplicated headache, nausea, vomiting, diarrhea, influenza,

and accidental trauma (e.g., sprained ankle) which may interfere with or prevent everyday life functions but do not constitute a substantial disruption.

e. Is a congenital anomaly/birth defect

f. Is another medically important condition

- Medical or scientific judgment should be exercised in deciding whether SAE reporting is appropriate in other situations such as important medical events that may not be immediately life-threatening or result in death or hospitalization but may jeopardize the patient or may require medical or surgical treatment to prevent one of the other outcomes listed in the above definition. These events should usually be considered serious.
- Examples of such events include invasive or malignant cancers, intensive treatment in an emergency room or at home for allergic bronchospasm, blood dyscrasias or convulsions that do not result in hospitalization, or development of drug dependency or drug abuse.

The terms "severe" and "serious" are not synonymous. Severity refers to the intensity of an AE (e.g., rated as mild, moderate, or severe, or according to NCI CTCAE; see Section 12.3; the event itself may be of relatively minor medical significance such as severe headache without any further findings).

Severity and seriousness need to be assessed independently for each AE recorded on the eCRF.

<u>Suspected unexpected serious adverse reactions</u>

Suspected unexpected serious adverse reactions are defined as any SAE considered related to the IMP (by the investigator or the sponsor) AND assessed as unexpected as per reference safety information (RSI) (by the sponsor).

The expectedness of an SAE related to the IMP is determined by the sponsor according to the RSI. This should be done from the perspective of events previously observed, not based on what might be anticipated from the pharmacological properties of a medicinal product.

An SAE assessed as related to the IMP is classified as unexpected when the nature or severity is not consistent with the applicable product information (= RSI, i.e., IB for an unauthorized IMP or summary of product characteristics (SmPC) for an authorized product). The term severity is used here to describe the intensity of a specific event. This has to be distinguished from the term serious.

The RSI is kept up to date by the Sponsor and in a definitive way presented in the Investigator’s Brochure. For the up-to-date version of the RSI, the latest approved edition of the IB is applicable.

12.3.3 Recording and follow-up of any AE

12.3.3.1 AE recording

The investigator must assess and document any AE regardless of association with the use of the trial treatment during the period of observation (refer to Section 8.3.1 for the timing).

- Data pertaining to AEs will be collected during each trial visit, either based on the patient's spontaneous description or investigator's inquiry, or discovered in the course of examinations done during the visit. Clinical significance of any sign or symptom must be evaluated by the investigator.
- Clinically significant findings must be documented as AEs in the eCRF. Findings that are evaluated as not clinically significant (e.g., an abnormal laboratory value without any clinical manifestation), should not be documented as AEs.
- The investigator will perform an assessment on:
    - Intensity according to CTCAE v5.0
    - Seriousness
    - Outcome
    - Causal relationship of the AE to the IMPs
    - Any action taken with the IMPs and/or any other action taken
    - If applicable: causal relationship to optional chemotherapy
- All assessments as well as AE term (diagnosis/description), start date and time of onset, end date and time must be documented in the eCRF.
- It is not acceptable for the investigator to send photocopies of the patient’s medical records in lieu of completion of the AE eCRF page.
- There may be instances when copies of medical records for certain cases are requested by the sponsor. In this case, all patient identifiers, with the exception of the patient number, will be redacted on the copies of the medical records before submission to the sponsor.
- The investigator will attempt to establish a diagnosis of the event based on signs, symptoms, and/or other clinical information. Whenever possible, the diagnosis (not the individual signs/symptoms) will be documented as the AE. If a definitive diagnosis is not possible, the individual signs and symptoms should be recorded.

12.3.3.2 Assessment of intensity

The intensity of an AE (i.e., severity of organ toxicity) will be graded according to the NCI CTCAE v5.0. AEs that are not listed in CTCAE v5.0 should be classified according to the investigator’s discretion as close as possible to CTCAE v5.0, based on the comparison with the most severe case encountered in past training and clinical experience.

- The investigator will make an assessment of intensity for each AE and SAE reported during the trial and will assign it to one of the following categories:

- Grade 1 - Mild
- Grade 2 - Moderate
- Grade 3 - Severe
- Grade 4 - Life-threatening consequences
- Grade 5 - Death related to AE

- An event is defined as ‘serious’ when it meets at least one of the pre-defined serious criteria as described in the definition of an SAE, NOT when it is rated as severe.

The following must be documented in the eCRF:

- Initial intensity of the AE
- For each change of intensity:
  - New grade of intensity
  - Date of change (= start of new grade of intensity)
  - Time of change (only if relevant)

A change of intensity only needs to be documented if there is a clearly definable change in grading of the AE (e.g., a laboratory result changes from severe to moderate according to CTCAE criteria).

12.3.3.3 Assessment of causality

- The investigator is obligated to assess the relationship (Table 24) between trial treatment/trial procedure and each occurrence of each AE/SAE.
- A “reasonable possibility” of a relationship conveys that there are facts, evidence, and/or arguments to suggest a causal relationship, rather than a relationship cannot be ruled out.
- The investigator will use clinical judgment to determine the relationship.
- Alternative causes, such as LD, underlying disease(s), concomitant therapy, and other risk factors, as well as the temporal relationship of the event to trial treatment administration will be considered and investigated.
- The investigator will also consult the IB and/or Product Information, for marketed products, in his/her assessment.
- There may be situations in which an SAE has occurred and the investigator has minimal information to include in the initial report to the sponsor. However, it is very important that the investigator always make an assessment of causality for every event before the initial transmission of the SAE data to the sponsor.
- The investigator may change his/her opinion of causality in light of follow-up information and send a SAE follow-up report with the updated causality assessment.
- The causality assessment is one of the criteria used when determining regulatory reporting requirements.

Table 24 can be taken as guidance:

Table 24: Assessment of causality

Causality term	Assessment criteria	Assessed as
Certain	• Event or laboratory test abnormality, with plausible time relationship to drug intake<br>• Cannot be explained by disease or other drugs<br>• Response to withdrawal plausible (pharmacologically, pathologically)<br>• Event definitive pharmacologically or phenomenologically (i.e., an objective and specific medical disorder or a recognized pharmacological phenomenon)<br>• Re-challenge satisfactory, if necessary	related
Probable/Likely	• Event or laboratory test abnormality, with reasonable time relationship to drug intake<br>• Unlikely to be attributed to disease or other drugs<br>• Response to withdrawal clinically reasonable<br>• Re-challenge not required	related
Possible	• Event or laboratory test abnormality, with reasonable time relationship to drug intake<br>• Could also be explained by disease or other drugs<br>• Information on drug withdrawal may be lacking or unclear	related
Unlikely	• Event or laboratory test abnormality, with a time to drug intake that makes a relationship improbable (but not impossible)<br>• Disease or other drugs provide plausible explanations	not related

It is sufficient to document the causality in the eCRF as:

- **Related, or**
- **Not related**

For an event considered "related to trial treatment" there is a **reasonable possibility** of a causal relationship.

For an event considered "not related to trial treatment" there is **no reasonable possibility** of a causal relationship.

Relationship to IMP-1, IMP-1(A), and IMP-2

The relationship or association of an AE or SAE to IMP(s) will be made by the investigator after having evaluated all accessible data and, if necessary, he/she will re-evaluate the case as new information becomes available.

Events caused by the procedure of IMP administration should be differentiated from events caused by the IMP(s) themselves. Only events suspected to be caused by the IMPs themselves should be documented as related.

Relationship to LD

The relationship or association of an AE or SAE to LD chemotherapy as an alternative cause will be made by the investigator after having evaluated all accessible data and, if necessary, he/she will re-evaluate the case as new information becomes available.

Events caused by the procedure of LD administration should be differentiated from events caused by the LD chemotherapy itself. Only events suspected to be caused by the LD itself should be documented as related.

Relationship to trial procedures

In this trial, it cannot be excluded that during the course of the trial some procedures give rise to AEs which are related to the trial procedure and not to the trial treatment. Procedure-related AEs can occur at the site of injection of the trial drug e.g., redness, swelling, hematoma or itching or during or after trial-specific procedure, e.g., discomfort after blood drawing, leukapheresis. These events have to be reported in the eCRF on the AE page as "related to trial procedure" with the causal procedure specified.

Relationship to optional chemotherapy

The relationship or association of an AE or SAE to optional chemotherapy as an alternative cause will be made by the investigator after having evaluated all accessible data and, if necessary, he/she will re-evaluate the case as new information becomes available.

Events caused by the procedure of optional chemotherapy administration should be differentiated from events caused by the optional chemotherapy itself. Only events suspected to be caused by the optional chemotherapy itself should be documented as related.

12.3.3.4 Action taken by the Investigator

Actions taken by the investigator as a result of an AE must be documented.

Action(s) taken with the IMPs:

- Dose increased
- Dose not changed
- Dose reduced*
- Drug interrupted
- Drug withdrawn
- Not applicable

* If an increase of trial drug dosage is intended according to the trial protocol and the dosage is kept the same as the last administration of trial drug, it needs to be documented as "Dose reduced".

Other action(s) that may be taken by the investigator include:

- None
- Initiation of a concomitant therapy for the treatment of the AE
- Termination of a concomitant medication
- Change of the dose of a concomitant medication
- Initiation/termination of a non-drug therapy
- Other (please specify)

12.3.3.5 Outcome

The investigator has to assess the outcome of an AE (and not the patient's outcome) at the time of documentation based on the following criteria:

- Recovered/resolved* (= complete resolution of the AE)
- Recovering/resolving (= AEs which are improving but not yet resolved completely, e.g., decrease in an intensity grade)
- Not recovered/not resolved (= AEs which are ongoing without improving or still present when the patient deceases due to another cause)
- Recovered/resolved with sequelae* (= patient recuperated but retained pathological conditions resulting from the AE; the sequelae should be indicated)
- Fatal** (= death due to the AE)
- Unknown (e.g., in case the patient is lost to follow-up)

* Generally, an AE is defined as recovered/resolved if all symptoms have ceased, no medication for treatment of the event is taken anymore and no other measures (e.g., hospitalization) are ongoing.

If the patient has developed permanent or chronic symptoms or if the event requires long-term medication(s), the AE is defined as recovered/resolved with sequelae as soon as no changes of symptoms and/or medication(s) are expected anymore.

An AE that is documented as a worsening of a medical condition already known at baseline, is defined as recovered as soon as the medical condition has returned to baseline status.

** In case of a fatal event, the event term should not be "death" but the underlying event which led to death (death = outcome). If there is more than one AE in a fatal case, only the AE leading to death will be attributed with the outcome "fatal". All other AEs ongoing at the time of death will be attributed with the outcome "not recovered/not resolved". A copy of an autopsy report should be submitted if available.

All ongoing AEs will be followed until resolution, considered by the investigator to be stable or chronic (resolved with sequelae), the patient is lost to follow-up or the patient withdraws consent. If no final status is reached at EoT, the investigator must confirm the unavailability of a final status.

12.3.3.6 SAE exemptions

In general, SAEs are defined according to ICH Topic E2A (CPMP/ICH/377/95), EU Directive 2001/20/EC and ENTR/CT-3. In the present trial, some events are excluded from the SAE definition. The following events do not need to be reported as SAEs:

- AEs and SAEs occurring later than 90 d after last administration of any IMP must only be reported by the investigator to the sponsor if a relationship to trial drug or trial procedure is suspected.
- Hospitalizations for respite care will not be considered as reportable SAE.
- Hospitalizations solely for coordination of care, including hospice arrangements, will not be considered as reportable SAE.
- Hospitalizations that were necessary solely because of patient requirement for outpatient care outside of normal outpatient clinic operating hours will not be considered as reportable SAE.
- Planned hospitalizations required by the protocol (e.g., for trial drug administration, hospitalization/observation for initial infusion of CLDN6 CAR-T, CLDN6 CAR-T(A), CLDN6 RNA-LPX vaccination or insertion of access device for trial drug administration) will not be considered as reportable SAE.
- Hospitalizations for procedures or interventions of a pre-existing condition of the patient (elective surgery = planned, non-emergency surgical procedure) will not be considered as a reportable SAE:
    - If it was planned and documented in the patient record before the trial-specific patient ICF was signed, or
    - If it was scheduled during the trial when elective surgery became necessary and the patient has not experienced an AE.

  Nevertheless, this kind of hospitalization should be avoided during trial treatment.

- Routine treatment or monitoring of the underlying disease not associated with any deterioration in the patient’s condition.
- The progression of underlying disease (e.g., new metastases) during trial participation is not considered as AE. However, specific symptoms at time of progression that may be considered as being caused by reasons other than the progression of underlying disease itself, and fatal cases where a cause other than the disease progression itself may not be discounted, will have to be documented as AEs and reported as SAEs if applicable.

12.3.3.7 Documentation of particular situations

<u>Adverse events that are secondary to other events:</u>

In general, AEs that are secondary to other events (e.g., cascade events or clinical sequelae) should be identified by their primary cause, with the exception of severe or serious secondary

events. A medically significant secondary AE that is separated in time from the initiating event should be documented as an independent AE in the eCRF. For example:

- If vomiting results in mild dehydration with no additional treatment in a healthy adult, only vomiting should be documented as AE.
- If vomiting results in severe dehydration, both events should be documented as AEs separately.

Abnormal laboratory results and vital signs values:

Not every laboratory or vital signs abnormality needs to be documented as AE. For clinically significant laboratory/vital signs abnormalities the following definitions and documentation rules apply:

- If a laboratory/vital sign abnormality is a sign of a disease or syndrome, the laboratory/vital signs abnormality is clinically significant and only the diagnosis of the causing disease or syndrome needs to be documented as AE.
- If a laboratory/vital sign abnormality results in specific symptoms, but no diagnosis of a disease or syndrome can be made, the laboratory/vital signs abnormality is clinically significant and only the symptoms need to be documented as AEs.
- If a laboratory/vital signs abnormality is not a sign of a disease or syndrome and does not result in specific symptoms but leads to a change in trial treatment or in a medical intervention, the laboratory/vital signs abnormality is clinically significant and must be documented as AE.

Death:

- Any death that occurs within the observation period will be reported as a SAE. Exemptions to the SAE definition as defined above do also apply for fatal cases. Date and cause of death will be recorded.
- In case of a fatal event, the event term should not be "death" but the underlying event which led to death (death = outcome). If there is more than one AE in a fatal case, only for the AE leading to death the outcome "fatal" should be selected. If the cause of death is unknown and cannot be ascertained at the time of reporting, "unexplained death" should be documented as event term.
- In addition to reporting as SAE, the death page of the eCRF needs to be completed.
- Autopsy results should be provided to the sponsor, autopsies are recommended.

Adverse events associated with an overdose or error in drug administration:

AEs associated with an overdose or error in drug administration are not expected.

In any case, all AEs associated with an overdose or incorrect administration should be documented as AEs in the eCRF and reported as SAEs if applicable.

12.3.3.8 Follow-up of AEs and SAEs

- The investigator is obligated to perform or arrange for the conduct of supplemental measurements and/or evaluations as medically indicated or as requested by the sponsor to elucidate the nature and/or causality of the AE or SAE as fully as possible. This may include additional laboratory tests or investigations, histopathological examinations, or consultation with other health care professionals.
- If a patient dies during participation in the trial or during a recognized follow-up period, the investigator will provide the sponsor with a copy of any post mortem findings including histopathology.
- New or updated information will be recorded in the originally completed eCRF.
- The investigator will submit any updated SAE data to the sponsor within 24 hours of receipt of the information.

12.3.4 Reporting of SAEs

12.3.4.1 SAE reporting to Sponsor via paper SAE form

All SAEs and AEs fulfilling the DLT criteria which occur during the observation period (refer to Section 8.3.1 for the timing), whether considered to be associated with trial medication or not, must be reported by the investigator **to the sponsor immediately but at the latest within 24 h following knowledge of the event**.

All SAEs occurring after the final visit or 90 days after last IMP treatment, whatever is the latest, only have to be reported to the sponsor if the investigator suspects a relationship to trial medication or the trial procedure.

The investigator needs to complete the paper **Serious Adverse Event Form** which has to be sent to the sponsor and report it via one of the following reporting lines:

Safety Report Fax No.: [redacted]

Safety Report Email Address: [redacted]

Information for final description and evaluation of a case report may not be available within the required time frames for reporting. Nevertheless, for regulatory purposes, initial reports should be submitted if the following minimal information is available:

- An identifiable patient (patient number)
- A suspected medicinal product
- An identifiable reporting source (investigator/trial site identification)
- An event or outcome that can be identified as serious

SAE follow-up information should be sent to the sponsor (indicating that this is a "follow-up" report) without delay as described above and accompanied by appropriate anonymous supporting documentation (e.g., discharge letters, medical reports or death certificates), until a final outcome and date are available. All confidential information (name, address, full day of birth) must be blackened before sending. In addition to a medical record, the investigator should complete an **Additional Information and Follow-up Form**, which contains the SAE term and patient number.

A copy of the submitted SAE report must be retained on file by the investigator. If explicitly required according to national legislation, the investigator must submit copies of the SAEs to the IRB/IEC or authority and retain documentation of these submissions in the Site Trial File.

In case an investigator or any other trial team member has questions on safety reporting to the sponsor, these can be addressed to:

Email: [redacted]

Please note that for medical questions the medical monitor for this trial should be contacted.

<u>24/7 Coverage for Urgent Protocol-related Medical Questions</u>

In a trial-related health emergency, a physician can be reached 24 hours per day, 7 days per week via:

TELEPHONE: [redacted]

12.4 APPENDIX 4: CONTRACEPTIVE GUIDANCE AND COLLECTION OF PREGNANCY INFORMATION

Definitions:

Woman of childbearing potential (WOCBP)

A woman is considered fertile following menarche and until becoming post-menopausal unless permanently sterile (see below).

If fertility is unclear (e.g., amenorrhea in adolescents or athletes) and a menstrual cycle cannot be confirmed before first dose of trial treatment, additional evaluation should be considered.

Women in the following categories are not considered WOCBP:

1) Premenarchal
2) Premenopausal female with one of the following:
   a) Documented hysterectomy
   b) Documented bilateral salpingectomy
   c) Documented bilateral oophorectomy

For patients with permanent infertility due to an alternate medical cause other than the above, (e.g., Müllerian agenesis, androgen insensitivity), investigator discretion should be applied to determining trial entry.

Note: Documentation can come from the site personnel's: review of the patient's medical records, medical examination, or medical history interview.

3) Post-menopausal female
   a) A post-menopausal state is defined as no menses for 12 months without an alternative medical cause.
      i) A high follicle-stimulating hormone (FSH) level in the post-menopausal range may be used to confirm a post-menopausal state in women not using hormonal contraception or hormonal replacement therapy (HRT). However, in the absence of 12 months of amenorrhea, confirmation with more than one FSH measurement is required.
4) Females on HRT and whose menopausal status is in doubt will be required to use one of the non-estrogen hormonal highly effective contraception methods if they wish to continue their HRT during the trial. Otherwise, they must discontinue HRT to allow confirmation of post-menopausal status before trial enrollment.

Contraception guidance

The contraception guidance derived from the cyclophosphamide SmPC must be observed in all patients receiving cyclophosphamide for lymphodepletion: Women should not become

pregnant during the treatment and for a period of 12 months following discontinuation of the therapy. Men should not father a child during the treatment and for a period of 12 months following discontinuation of the therapy.

Furthermore, as no CLDN6 CAR-T persistence data are available, no recommendations can be made with regards to the cessation of the use of contraception beyond this point of time. This guidance will be updated with emerging persistence data.

The investigator or delegate should advise the patient how to achieve adequate contraception. The following birth control methods may be considered as highly effective (with a failure rate of < 1% per year):

- Combined (estrogen and progestogen containing) hormonal contraception associated with inhibition of ovulation:
    - Oral.
    - Intravaginal.
    - Transdermal.
- Progestogen-only hormonal contraception associated with inhibition of ovulation:
    - Oral.
    - Injectable.
    - Implantable.
- Intrauterine device (IUD).
- Intrauterine hormone-releasing system (IUS).
- Bilateral tubal occlusion.
- Vasectomy (for a male patient or male partner of a female patient, provided that partner is the sole sexual partner of the WOCBP trial patient and that the vasectomized partner has received medical assessment of the surgical success).
- Female sterilization.

True abstinence (refraining from heterosexual intercourse during the entire period of risk associated with the trial treatments) is an acceptable alternative.

Collection of pregnancy information

Male patients with partners who become pregnant

- The investigator will attempt to collect pregnancy information on any male patient's female partner who becomes pregnant after the start of trial intervention until the end of LTFU.
- After obtaining the necessary signed ICF from the pregnant female partner directly, the investigator will record pregnancy information on the paper-based Pregnancy Reporting Form and submit it to the sponsor within 24 hours of learning of the partner's pregnancy, as described in Section 12.3.4.1. The female partner will also be followed to determine the outcome of the pregnancy. Information on the status of the

mother and child will be forwarded to the sponsor. Generally, the follow-up will be no longer than 6 to 8 weeks following the estimated delivery date. Any termination of the pregnancy will be reported regardless of fetal status (presence or absence of anomalies) or indication for the procedure.

Female Patients who become pregnant

- The investigator will collect pregnancy information on any female patient who becomes pregnant after the start of trial intervention until the end of LTFU. The initial information will be recorded on the paper-based Pregnancy Reporting Form and submitted to the sponsor within 24 hours of learning of a patient's pregnancy, as described in Section 12.3.4.1.
- The patient will be followed to determine the outcome of the pregnancy. The investigator will collect follow-up information on the patient and the neonate and the information will be forwarded to the sponsor. Generally, follow-up will not be required for longer than 6 to 8 weeks beyond the estimated delivery date. Any termination of pregnancy will be reported, regardless of fetal status (presence or absence of anomalies) or indication for the procedure.
- While pregnancy itself is not considered to be an AE or SAE, any pregnancy complication or elective termination of a pregnancy for medical reasons will be reported as an AE or SAE.
- A spontaneous abortion (occurring at < 22 weeks gestational age) or still birth (occurring at > 22 weeks gestational age) is always considered to be an SAE and will be reported as such.
- Any post-trial pregnancy related SAE considered reasonably related to the trial treatment by the investigator will be reported to the sponsor as described in Section 12.3.4.1. While the investigator is not obligated to actively seek this information in former trial patients, he or she may learn of an SAE through spontaneous reporting.
- Any female patient who becomes pregnant while participating in the trial will discontinue trial treatment.

12.5 APPENDIX 5 RECIST 1.1 DEFINITIONS AND RESPONSE CRITERIA

For the evaluation of measurable disease, target, non-target lesions and response, following definitions and criteria apply:

Measurable is defined by the presence of at least one measurable lesion:

- Tumor lesions: Must be accurately measured in at least one dimension (longest diameter in the plane of measurement is to be recorded) with a minimum size of 10mm by CT scan (CT scan slice thickness no greater than 5 mm).
- Malignant lymph nodes: To be considered pathologically enlarged and measurable, a lymph node must be ≥15 mm in short axis when assessed by CT scan (CT scan slice thickness recommended to be no greater than 5 mm). At baseline and in follow-up, only the short axis will be measured and followed.

Target lesions:

- Target lesions should be selected on the basis of their size (lesions with the longest diameter), be representative of all involved organs, but in addition should be those that lend themselves to reproducible repeated measurements. A sum of the diameters (longest for non-nodal lesions, short axis for nodal lesions) for all target lesions will be calculated and reported as the baseline sum diameters.

Non-target lesions:

- All other lesions (or sites of disease) including pathological lymph nodes

Response criteria

Evaluation of target lesions:

- Complete Response (CR): Disappearance of all target lesions. Any pathological lymph nodes (whether target or non-target) must have reduction in short axis to < 10 mm.
- PR: At least a 30% decrease in the sum of diameters of target lesions, taking as reference the baseline sum diameters.
- Progressive Disease (PD): At least a 20% increase in the sum of diameters of target lesions, taking as reference the smallest sum on study (this includes the baseline sum if that is the smallest on study). In addition to the relative increase of 20%, the sum must also demonstrate an absolute increase of at least 5 mm. (Note: the appearance of one or more new lesions is also considered progression).
- SD: Neither sufficient shrinkage to qualify for PR nor sufficient increase to qualify for PD, taking as reference the smallest sum diameters while on study.

Evaluation of non-target lesions:

- CR: Disappearance of all non-target lesions and normalization of tumor marker level. All lymph nodes must be non-pathological in size (< 10mm short axis).
- Non-CR/Non-PD: Persistence of one or more non-target lesion(s) and/or maintenance of tumor marker level above the normal limits.
- PD: Unequivocal progression of existing non-target lesions. (Note: the appearance of one or more new lesions is also considered progression).

For a complete description, refer to Eisenhauer 2009.

12.6 APPENDIX 6 IRECIST DEFINITIONS AND RESPONSE CRITERIA

iRECIST is based on RECIST 1.1. Responses assigned using iRECIST have a prefix of "i" (i.e., immune)—e.g., "immune" complete response (iCR) or partial response (iPR), and unconfirmed progressive disease (iUPD) or confirmed progressive disease (iCPD) to differentiate them from responses assigned using RECIST 1.1. Similar nomenclature is used for stable disease (iSD). New lesions are assessed and subcategorized into those that qualify as target lesions (new lesion, target) or non-target lesions (new lesion, non-target).

The principles used to establish objective tumor response are largely unchanged from RECIST 1.1, but the major change for iRECIST is the concept of resetting the bar if RECIST 1.1 progression is followed at the next assessment by tumor shrinkage. Target lesions, non-target lesions, and new lesions defined according to RECIST 1.1 principles; if no pseudo-progression occurs, RECIST 1.1 and iRECIST categories for complete response, partial response, and stable disease would be the same.

Table 25: Comparison of RECIST 1.1 and iRECIST

	**RECIST 1.1**	**iRECIST**
Definitions of measurable and non-measurable disease; numbers and site of target disease	Measurable lesions are ≥ 10 mm in diameter (≥ 15 mm for nodal lesions); maximum of five lesions (two per organ); all other disease is considered non-target (must be ≥ 10 mm in short axis for nodal disease)	No change from RECIST 1.1; however, new lesions are assessed as per RECIST 1.1 but are recorded separately on the case report form (but not included in the sum of lesions for target lesions identified at baseline)
Complete response, partial response, or stable disease	Cannot have met criteria for progression before complete response, partial response, or stable disease	Can have had iUPD (one or more instances), but not iCPD, before iCR, iPR, or iSD
Confirmation of complete response or partial response	Only required for non-randomized trials	As per RECIST 1.1
Confirmation of stable disease	Not required	As per RECIST 1.1
New lesions	Result in progression; recorded but not measured	Results in iUPD but iCPD is only assigned on the basis of this category if at next assessment additional new lesions appear or an increase in size of new lesions is seen (≥ 5 mm for sum of new lesion target or any increase in new lesion non-target); the appearance of new lesions when none have previously been recorded, can also confirm iCPD
Independent blinded review and central collection of scans	Recommended in some circumstances —e.g., in some trials with progression-based endpoints planned for marketing approval	Collection of scans (but not independent review) recommended for all trials
Confirmation of progression	Not required (unless equivocal)	Required
Consideration of clinical status	Not included in assessment	Clinical stability is considered when deciding whether treatment is continued after iUPD

Abbreviations: RECIST, Response Evaluation Criteria in Solid Tumors; iUPD, unconfirmed progression; iCPD, confirmed progression; iCR, complete response; iPR, partial response; iSD, stable disease.
"i" indicates immune responses assigned using iRECIST.

Table 26: Assignment of timepoint response using iRECIST

	**Timepoint response with no previous iUPD in any category**	**Timepoint response with previous iUPD in any category***
Target lesions: iCR; non-target lesions: iCR; new lesions: no	iCR	iCR
Target lesions: iCR; non-target lesions: non-iCR/non-iUPD; new lesions: no	iPR	iPR
Target lesions: iPR; non-target lesions: non-iCR/non-iUPD; new lesions: no	iPR	iPR
Target lesions: iSD; non-target lesions: non-iCR/non-iUPD; new lesions: no	iSD	iSD
Target lesions: iUPD with no change, or with a decrease from last timepoint; non-target lesions: iUPD with no change, or decrease from last timepoint; new lesions: yes	Not applicable	New lesions confirm iCPD if new lesions were previously identified and they have increased in size (≥ 5 mm in sum of measures for new lesion target or any increase for new lesion non-target) or number; if no change is seen in new lesions (size or number) from last timepoint, assignment remains iUPD
Target lesions: iSD, iPR, iCR; non-target lesions: iUPD-; new lesions: no	iUPD	Remains iUPD unless iCPD is confirmed on the basis of a further increase in the size of non-target disease (does not need to meet RECIST 1.1 criteria for unequivocal progression)
Target lesions: iUPD; non-target lesions: non-iCR/non-iUPD, or iCR; new lesions: no	iUPD	Remains iUPD unless iCPD is confirmed on the basis of a further increase in sum of measures ≥ 5 mm; otherwise, assignment remains iUPD
Target lesions: iUPD; non-target lesions: iUPD; new lesions: yes	iUPD	Remains iUPD unless iCPD is confirmed based on a further increase in previously identified target lesion iUPD in sum of measures > 5 mm or non-target lesion iUPD (previous assessment need not have shown unequivocal progression)
Target lesions: iUPD; non-target lesions: iUPD; new lesions: yes	iUPD	Remains iUPD unless iCPD is confirmed on the basis of a further increase in previously identified target lesion iUPD sum of measures ≥5 mm, previously identified non-target lesion iUPD (does not need to be unequivocal), or an increase in the size or number of new lesions previously identified

	Timepoint response with no previous iUPD in any category	Timepoint response with previous iUPD in any category*
Target lesions: non-iUPD or progression; non-target lesions: non-iUPD or progression; new lesions: yes	iUPD	Remains iUPD unless iCPD is confirmed on the basis of an increase in the size or number of new lesions previously identified

iCR, complete response; iPR, partial response; iSD, stable disease; iUPD, unconfirmed progression, non-iCR/non-iUPD, criteria for neither CR nor PD have been met; iCPD, confirmed progression; RECIST, Response Evaluation Criteria in Solid Tumors.
*Previously identified in assessment immediately before this timepoint. “i” indicates immune responses assigned using iRECIST.

For a complete description, refer to Seymour et al. 2017.

12.7 APPENDIX 7 COUNTRY-SPECIFIC REQUIREMENTS

Not applicable.

12.8 APPENDIX 8 LONG-TERM FOLLOW-UP

12.8.1 Introduction and rationale

Patients exposed to genetically engineered therapies, including CAR modified T cells and other genetically engineered T cell products, may be at risk of delayed AEs. The risk for delayed adverse reactions and decreasing efficacy for genetically modified cells is correlated to the actual risk profile of the vector used for the genetic modification of the cell, the nature of the gene product, the life-span (persistence) of the modified cells, and the biodistribution. The LTFU evaluation in this trial is designed to adhere to the FDA and EMA guidance for LTFU of patients in gene therapy clinical trials (EMEA 2009; FDA 2006; FDA 2018), and involves monitoring patients for 15 years after they have been treated with genetically engineered medicinal therapies that use integrating vectors (i.e., retroviral vectors).

Production of genetically engineered therapies such as CAR or T cell receptor (TCR) genetically modified T cells, requires T cells to be genetically modified by *ex vivo* transduction using a recombinant viral vector containing the CAR ribonucleic acid (RNA) sequence or TCR genes. Viral vectors are usually derived from retroviruses, and lentiviruses and gamma-retroviruses are retroviruses subgroups. The term "replication-competent retroviruses" (RCR) will be used to indicate both replication-competent lentiviruses and gamma-retroviruses. Even if the IMP was engineered through the use of replication-incompetent lentiviruses or gamma-retroviruses there is a theoretical risk for the emergence of RCRs. As a consequence, potential toxicities and unexpected AEs may arise from the presence and activity of these RCRs. These toxicities and delayed AEs might be related to the administered genetically engineered product *per se*, or to integration of these RCRs in the host genome.

Therefore, after patients complete or prematurely discontinue participation in the trial, they will be asked to participate in a separate 15-year Long-term Follow-up trial.

12.8.2 Objectives and endpoints

Objectives and related endpoints for the 15-year LTFU are described in Table 27.

Table 27: Objectives and endpoints

Objectives	Endpoints
**Primary**	
• To assess the long-term safety of CLDN6 CAR-T/CLDN6 CAR-T(A) +/- CLDN6 RNA-LPX<br>• To assess the risk of delayed related AEs following the administration of	• Occurrence of AEs during the LTFU period suspected to be related to CLDN6 CAR-T/CLDN6 CAR-T(A) +/- CLDN6 RNA-LPX, such as:<br>o New malignancy (hematologic or solid)

CLDN6 CAR-T/CLDN6 CAR-T(A) +/- RNA-LPX	○ New neurologic disorder, or exacerbation of a pre-existing disorder<br>○ New rheumatologic or autoimmune disorder, or exacerbation of a prior rheumatologic or other autoimmune disorder<br>○ New hematologic disorder<br>○ Other new clinical condition considered by the Investigator to be related to the prior genetically engineered T cell therapy
**Secondary**	
• To assess the long-term efficacy following treatment with CLDN6 CAR-T/CLDN6 CAR-T(A) +/- CLDN6 RNA-LPX	• Progression-free survival<br>• Overall survival. Survival status will be collected until 15 years from last genetically engineered T cell infusion or until death, whichever occurs first
**Exploratory**	
• To assess long-term efficacy following treatment with CLDN6 CAR-T/CLDN6 CAR-T(A) +/- CLDN6 RNA-LPX	• Presence of CLDN6 CAR-T/CLDN6 CAR-T(A) in blood and other tissues if available

Abbreviations: AE, adverse event; CAR-T, chimeric antigen receptor T cell; CLDN6, claudin 6; LTFU, long-term follow-up; RNA-LPX, liposomally formulated ribonucleic acid encoding the vaccine.

12.8.3 Trial design

This is an open-label extension of the main trial that will evaluate the LTFU safety and efficacy of patients previously treated with CLDN6 CAR-T/CLDN6 CAR-T(A). Participating patients will be followed from time of roll over from the main trial until 15 years after the last CAR-T infusion, or until withdrawal of consent, lost to follow-up, or death, whichever occurs first.

Patients who consent to be enrolled on this LTFU protocol will roll over from the main trial, on either completion or premature discontinuation from the main trial. Patients who complete the main trial, which includes a 2-year follow-up will enter the LTFU at Year 3 and will continue to be followed for 13 years. Patients who receive at least one CAR-T infusion but who discontinue early from the main trial will enter the LTFU at the visit that corresponds to completion of a total of 15 years follow-up from the last infusion of CAR-T.

Safety assessments will be performed at the time points defined in the SoA, Table 28. The baseline for these assessments will be the last assessment recorded from the main trial. The end of the LTFU trial is defined as the date of the last assessment at the last scheduled visit for the last patient.

12.8.4 Trial population

Any patient who is enrolled in the main trial and who received at least one CAR-T infusion will be asked to roll over to this LTFU on either premature discontinuation from, or completion of the prior main trial.

12.8.4.1 Inclusion criteria

1. Patients must understand and sign a written ICF prior to any assessment being performed.
2. Patients who received at least one CAR-T infusion as part of their disease treatment and who have completed the main treatment trial or have prematurely discontinued from the main treatment trial.
3. Patient is willing and able to adhere to the scheduled LTFU visits and related requirements.

12.8.4.2 Exclusion criteria

There are no additional exclusion criteria to those defined in the main trial protocol. Participation on other clinical trials or treatment with any anti-cancer therapy is not contraindicated and does not preclude the patient participating in this LTFU trial.

12.8.5 Trial treatments

No IMP will be administered during the LTFU trial.

12.8.6 Further therapies

Any potentially mutagenic agents (e.g., chemo[radio]therapy), other IMPs, or anti-cancer therapies taken since administration of the CAR-T infusion will have been recorded during the main trial, and will continue to be recorded during the LTFU (refer to Table 28), along with reason for use, dates of administration including start and end dates, dosage information including dose and frequency.

12.8.7 Patient discontinuation and withdrawal

Patients will be encouraged to participate in this LTFU until completion. However, patients may withdraw consent at any time.

12.8.8 Trial assessments and procedures

The SoA for the LTFU is provided in Table 28.

12.8.8.1 Efficacy assessments

The disease status of the primary malignancy will be assessed as indicated and as per local institutional guidelines.

Since no IMP will be administered during this LTFU trial, participation on other clinical trials or treatment with any anti-cancer therapy is not contraindicated and does not preclude the patient participating in this LTFU trial.

12.8.8.1.1 CLDN6 CAR-T persistence

Presence of CAR-T will be assessed in blood and other tissues, if available.

12.8.8.1.2 Tumor assessment

For patients entering the LTFU in CR: Imaging studies will be performed according to RECIST v1.1 and iRECIST if there is a clinical suspicion of relapse.

For all other patients: Imaging studies will be performed according to RECIST v1.1 and iRECIST as clinically indicated.

12.8.8.1.3 Survival status

Survival status (PFS where relevant and OS) will be collected until 15 years from last genetically engineered T cell infusion or until death, whichever occurs first.

12.8.8.2 Safety assessments

Due to the potential risk related to the administration of CLDN6 CAR-T/CLDN6 CAR-T(A), related AEs will be documented.

All patients will undergo safety assessment through a brief clinical history, physical examination, and screening for development of potentially related delayed AEs. These new potentially related AEs include new malignancy (hematological or solid), neurologic disorder or exacerbation of pre-existing disorder, new rheumatic or autoimmune disorder or exacerbation of prior rheumatic disorder or other autoimmune disorder, new hematologic disorder, or other new clinical condition considered by the investigator to be related to the prior genetically engineered T-cell therapy.

Delayed related AEs or SAEs occurring after the administration of at least one infusion of CLDN6 CAR-T/CLDN6 CAR-T(A) will be collected during this LTFU trial. The documentation of any related AEs, SAEs in the eCRF must be supported by an entry in the patient study files during the LTFU trial. Whenever possible the severity of each AE should

be graded as per the CTCAE version 5.0. If the AE is not defined in the CTCAE it should then be evaluated for frequency and severity to a similar scale to the one used in the CTCAE. Refer to Appendix 4, Section 12.3.4.1 for details of the safety reporting email address and fax number.

- Grade 1 = mild
- Grade 2 = moderate
- Grade 3 = severe
- Grade 4 = life-threatening
- Grade 5 = death

12.8.8.2.1 Physical examination

A complete physical examination at each LTFU Visit will be performed and recorded. If clinically indicated, this physical examination will include the evaluation of the head, eyes, ears, nose, and throat, and the cardiovascular, dermatological, musculoskeletal, respiratory, gastrointestinal, genitourinary, and neurological systems. New or worsened clinically significant abnormalities considered by the investigator to be related to the prior genetically engineered T cell therapy will be recorded as AEs (refer to Section 12.8.8.2.3).

Patients should be encouraged to monitor themselves for any new or worsening AEs and report them to their investigator and/or study staff during their visit at the clinical site. All AEs and SAEs will be recorded by the investigator throughout the duration of the patient LTFU. Hospitalizations should also be reported to the investigator and/or study staff.

12.8.8.2.2 Clinical safety laboratory parameters

The clinical laboratory tests detailed in Table 28 will be performed at each LTFU Visit.

Local laboratory safety evaluations will be collected as indicated and as per local institutional guidelines. Only clinically relevant changes, assessed as related to the IMP, will be recorded in the eCRF (on the AE pages only).

12.8.8.2.3 Adverse events and serious adverse events

AE and SAE collection will be limited to new malignancy (hematological or solid), neurologic disorder or exacerbation of pre-existing disorder, new rheumatic or autoimmune disorder or exacerbation of prior rheumatic disorder or other autoimmune disorder, new hematologic disorder, or other new clinical condition considered by the investigator to be related to the prior genetically engineered T cell therapy.

12.8.8.2.4 Pregnancy

Any pregnancy or suspected pregnancy should be immediately reported to the sponsor as described in Section 12.4. The outcome of the pregnancy will be assessed and recorded.

If a female partner of a male patient becomes pregnant, the male patient should notify the investigator and/or study staff, and the pregnant female partner should be advised to call her healthcare provider immediately.

Table 28: Schedule of activities for long-term follow-up

	Year 1				Year 2		Year 3		Year 4		Year 5		Years 6-15
**Months**	**2**	**3**	**6**	**12**	**18**	**24**	**30**	**36**	**42**	**48**	**54**	**60**	**Annually**
**Visit window**	**± 1 month**												**± 3 months**
Physical examination	X	X	X	X	X	X	X	X	X	X	X	X	X
Any potentially mutagenic agents (e.g., chemo[radio]therapy), other IMPs or anti-cancer therapies	X	X	X	X	X	X	X	X	X	X	X	X	X
AEs considered as possibly related to prior exposure to CLDN6 CAR-T	X	X	X	X	X	X	X	X	X	X	X	X	X
CLDN6 CAR-T presence in blood [1]	X	X	X	X	X	X	X	X	X	X	X	X	X
Local laboratory assessments for safety (per institutional guidelines)	X	X	X	X	X	X	X	X	X	X	X	X	X
Tumor assessment	**Information from tumor assessments (as part of regular care) will be collected until patient dies, withdraws consent, or is lost to follow-up.**												
Survival status	**Survival status will be collected until patient dies, withdraws consent, or is lost to follow-up. Patients will be followed beyond disease progression.**												
Pregnancy	**Information relating to a pregnancy or suspected pregnancy should be immediately reported to the sponsor**												
Hospitalizations	X	X	X	X	X	X	X	X	X	X	X	X	X

Abbreviations: AE, adverse event; CAR-T, chimeric antigen receptor T cell; CLDN6, claudin 6.

1. Samples will only be collected if CLDN6 CAR-T are detectable by qPCR.

STATISTICAL ANALYSIS PLAN (SAP)
BNT211-01

Version: Final v2.0 **Date:** 20Jul2022
Sponsor: BioNTech Cell & Gene Therapies GmbH

**Protocol number:**	BNT211-01
**Protocol title:**	Phase I/IIa, first-in-human, open-label, dose escalation trial with expansion cohorts to evaluate safety and preliminary efficacy of CLDN6 CAR-T with or without CLDN6 RNA-LPX in patients with CLDN6-positive relapsed or refractory advanced solid tumors
**Protocol version:**	5.0
**Protocol date:**	23 Nov 2021
**Investigational medicinal products:**	CLDN6 CAR-T, CLDN6 CAR-T(A), CLDN6 RNA-LPX
**CRO name:**	ICON plc, South County Business Park, Leopardstown, Dublin 18, D18 X5R3, Ireland
**SAP author:**	[redacted], Senior Statistician

Confidentiality Statement: The information contained in this document is the property and copyright of BioNTech Cell & Gene Therapies GmbH. Therefore, this document is provided in confidence to the recipient. No information contained herein shall be published, disclosed or reproduced without prior written approval of the proprietor(s).

Prepared at ICON Clinical Research by: [redacted]

REASON: I approve this document as author.
d11a85e3-4468-4c08-9bb9-ac58f3c8cdaa

[redacted], **Senior statistician** **Date**

Reviewed at ICON Clinical Research by: [redacted]

REASON: I approve this document
d27e4348-6021-4e17-83b8-9e14a0b4de64

[redacted], **Principal Biostatistician** **Date**

Approved at BioNTech by: [redacted]

REASON: I approve this document
5b168162-1a94-4153-a9eb-129c41d5e446

[redacted], **Director Biostatistics** **Date**

[redacted]

REASON: I approve this document
4dbe26de-e138-4c3c-9186-09b7f137bebe

[redacted], **VP Clinical research** **Date**

DocUUID : 6a803e66-f054-4d62-ad9b-00b24cc3317b

TABLE OF CONTENTS

1	SAP APPROVAL	5
2	VERSION HISTORY	5
3	INTRODUCTION	7
3.1	Objectives and endpoints	7
3.2	Trial design	11
3.3	Schema (graphical representation of the trial)	11
3.4	Schedule of activities	17
4	STATISTICAL HYPOTHESES	17
5	INTERIM ANALYSES AND ANALYSIS SEQUENCE	17
6	SAMPLE SIZE DETERMINATION	17
7	ANALYSIS SETS AND SUBGROUPS	17
7.1	Analysis sets	17
7.2	Protocol deviations	19
7.3	Subgroups	19
8	STATISTICAL ANALYSES	19
8.1	General considerations	19
8.2	Patient disposition	24
8.3	Baseline characteristics	25
8.3.1	Demographics	25
8.3.2	Disease characteristics	25
8.3.3	Prior anti-cancer treatments	26
8.3.4	Prior and Concomitant medication/Procedures/Non-drug Therapy	27
8.3.5	Medical history	28
8.4	Efficacy analyses	28
8.4.1	Primary analysis	28
8.4.2	Supplementary analyses	28
8.4.3	Secondary analyses	28
8.4.4	Exploratory analyses	29
8.5	Safety analyses	31
8.5.1	Extent of exposure	31
8.5.2	Adverse events	33
8.5.3	Laboratory assessments	37
8.5.4	Vital signs	38
8.5.5	ECG 39	
8.5.6	Physical Examination	39
8.5.7	ECOG Performance status	39

DocUUID : 6a803e66-f054-4d62-ad9b-00b24cc3317b

8.5.8	Leukapheresis	39
8.5.9	Lymphodepleting Chemotherapy/ Bridging Chemotherapy	39
8.5.10	Cytokine Release Syndrome (CRS)	39
8.5.11	Immune Effector Cell-Associated Neurotoxicity Syndrome (ICANS)	40
8.6	Other analyses	40
8.6.1	Pharmacokinetics	40
8.6.2	Pharmacodynamics and Biomarkers	40
8.6.3	New Antineoplastic medications	40
8.6.4	Hospitalizations	40
9	REFERENCES	41
10	SUPPORTING DOCUMENTATION	42
10.1	Appendix 1: Changes to protocol-planned analyses	42
10.2	Appendix 2: List of abbreviations	43
10.3	Appendix 3: Reporting conventions	46
10.4	Appendix 4: Schedule of activities	49
10.4.1	Dose Escalation (Parts 1 and 2)	49
10.5	Appendix 5: Censoring Rules	95
10.6	Appendix 6: Dose escalation table	100

DocUUID : 6a803e66-f054-4d62-ad9b-00b24cc3317b

1 SAP APPROVAL

This SAP has been prepared, reviewed, and approved in accordance with the BioNTech standard operating procedure (SOP). Documentation of this process is filed in the trial master file (TMF).

2 VERSION HISTORY

Table 1: SAP Version History Summary

SAP version	Version date	Change	Rationale
Final V1.0	02Nov2021	First version	
Final v2.0	29June2022	Updated for protocol amendment (Protocol v5.0)	Protocol amendment
		Added to changes from protocol section the removal of the '' *'Patients who have received an out of scope specification"* text from the mITT analysis set definition.	Not appropriated for mITT analysis set.
		Section 8.1	
		• Added clarification about the denominator that should be used for percentage calculations.	Clarification
		• Clarified that for this study confirmation of CR and PR is not required.	Clarification
		• Added text to clarify that A separate programming guideline will be used for programming best overall response using RECIST 1.1 and iRECIST criteria	Clarification
		• Section 8.1: The text 'If an AE has a missing stop date, then the AE will be reported as treatment–emergent.'' Was updated to ''If an AE has a missing start date, then the AE will be reported as treatment–emergent	Clarification
		• Added detail that for the analysis of ORR, DCR, DOR, PFS, OS endpoints All tumour assessment data and OS data from the main trial and LTFU (up to and including the clinical cut-off date) will be used for the analysis.	Clarification
		Section 8.4.4:	
		• Updated iDOR calculation to clarify that the date of iUPD associated with confirmed iCPD should be used.	Clarification
		• Updated PFS calculation to clarify that the date of iUPD associated with confirmed iCPD should be used.	To ensure meaning was understood.

DocUUID : 6a803e66-f054-4d62-ad9b-00b24cc3317b

		• Added 'censor date' to overall survival calculation.<br><br>Appendix: Generic SAS code removed.<br><br>Section 10.5 Appendix 5: Censoring Rules:<br>• Amended 'before' to 'not after'<br>• Added clarity that for iRECIST the date of iUPD is used if progression has been confirmed.<br>• For the censoring rules '' Progression documented on or between scheduled tumor assessments *prior to treatment discontinuation*" and ''Death without objective progression *prior to treatment discontinuation*'' '' ''Death or progression not after/after 2 or more consecutive missing scheduled assessments *during the treatment period or before 1 or more missed scheduled assessments during the Follow-up period*'' the text in italics was removed as not relevant for this study as Part 1 is single dose and as censoring rules for Part 1 and Part 2 are the same.	Clarification<br><br>SAP will not contain generic SAS code<br><br>Clarification<br><br>Censoring rule not relevant for single dose.

DocUUID : 6a803e66-f054-4d62-ad9b-00b24cc3317b

3 INTRODUCTION

This is a first-in-human (FIH), open-label, multicenter Phase I/IIa dose escalation trial of CLDN6 CAR-T with or without CLDN6 RNA-LPX with expansion cohorts in patients with CLDN6 positive relapsed or refractory advanced solid tumors. This statistical analysis plan (SAP) describes the detailed procedures for the planned statistical analyses for Part 1 and Part 2 of the trial for protocol v3.0 dated 19 Feb 2021 and protocol v5.0 (amendment v4.0) dated 23Nov2021, to support the completion of the Clinical Trial Report (CTR). Part 3 of the clinical trial will be initiated by a protocol amendment and the SAP will be amended subsequently extending the scope of analyses. This SAP will include presentation/analysis of data from the Main trial and also some data (tumour assessments and Overall survival data) from the LTFU (Long Term Follow-up) trial. Section 8.1 contains details about this.

The statistical analyses will be conducted by BioNTech or ICON using SAS® software Version 9.4 or higher.

3.1 Objectives and endpoints

Objectives	Endpoints
Primary	
To assess the safety and tolerability of CLDN6 CAR-T/CLDN6 CAR-T(A) +/- CLDN6 RNA-LPX and to assess the comparability of CLDN6 CAR-T and CLDN6 CAR-T(A)	• Occurrence of treatment-emergent adverse events (TEAEs) within a patient including ≥ Grade 3, serious, fatal TEAEs by relationship<br>• Occurrence of dose reduction and discontinuation of investigational medicinal product (IMP) within a patient due to TEAEs
To identify the maximum tolerated dose (MTD)/ recommended Phase 2 dose (RP2D) for each IMP (i.e. CLDN6 CAR-T/CLDN6 CAR-T(A) +/- CLDN6 RNA-LPX) based on the occurrence of DLTs using the following definitions:<br>• MTD is defined as the highest tolerated dose of CLDN6 CAR-T/CLDN6 CAR-T(A) +/- CLDN6 RNA-LPX where less than 33% of the patients experience a DLT<br>• RP2D of CLDN6 CAR-T/CLDN6 CAR-T(A) +/- CLDN6 RNA-LPX based on integrated evaluation of safety and other data for all dose levels tested	• Occurrence of DLTs within a patient during the DLT evaluation period
Secondary	

DocUUID : 6a803e66-f054-4d62-ad9b-00b24cc3317b

Objectives	Endpoints
To describe the profile of soluble immune factors in CLDN6 CAR-T/CLDN6 CAR-T(A) +/- CLDN6 RNALPX	• Change from baseline in the levels and kinetics of soluble immune factors measured by cytokine multiplex assay
To evaluate anti-tumor activity of CLDN6 CAR-T/CLDN6 CAR-T(A) +/- CLDN6 RNA-LPX according to response evaluation criteria in solid tumors version 1.1 (RECIST 1.1)	• Objective response rate (ORR) defined as the proportion of patients in whom a CR or PR (per RECIST 1.1) is observed as best overall response<br>• Disease control rate (DCR) defined as the proportion of patients in whom a CR or PR or stable disease (SD) per RECIST 1.1 (SD assessed at least 6 weeks after the first dose) is observed as best overall response<br>• Duration of response (DOR) defined as the time from first objective response (CR or PR per RECIST 1.1) to firstoccurrence of objective PD perRECIST 1.1/recurrence or death from any cause, whichever occurs first
Exploratory	
To evaluate anti-tumor activity of CLDN6 CAR-T/CLDN6 CAR-T(A) +/- CLDN6 RNA-LPX according to immune response evaluation criteria in solid tumors (iRECIST)	• Objective response rate per iRECIST (iORR) defined as the proportion of patients in whom a complete response or partial response per iRECIST (iCR or iPR) is observed as best overall response<br>• Disease control rate (iDCR per iRECIST) defined as the proportion ofpatients in whom an iCR or iPR or stable disease (iSD) per iRECIST (iSD assessed at least 6 weeks after the first dose), is observed as best overall response<br>• iDOR defined as the time from first objective response (iCR or iPR per iRECIST) to first occurrence of objective tumor progression (iCPD periRECIST) or death from any cause,whichever occurs first

Objectives	Endpoints
To evaluate efficacy of CLDN6 CAR-T/CLDN6 CAR-T(A) +/- CLDN6 RNA-LPX	• Progression-free survival (PFS) defined as the time from first dose of CLDN6 CAR-T/CLDN6 CAR-T(A) to first objective PD per RECIST 1.1, or death from any cause, whichever occurs first<br>• PFS defined as the time from first doseof CLDN6 CAR-T/CLDN6 CAR-T(A) to first objectiveiCPD, or death from any cause,whichever occurs first<br>• Overall Survival (OS) defined as the time from first dose of CLDN6 CAR-T/CLDN6 CAR-T(A) to death from any cause<br>• Time to treatment failure (TTF) in patients treated with CLDN6 RNALPX, defined as time from first injection to discontinuation of treatment for any reason including disease progression, treatment toxicity, add-on of new anti-cancer therapy, and death
Preliminary assessment of biomarkers that might act as pharmacodynamic, anti-tumor, and safety indicators of activity of CLDN6 CAR-T/CLDN6 CAR-T(A) +/- CLDN6 RNA-LPX	• Detection, phenotypic characterization and functionality of CLDN6 CAR-T/CLDN6 CAR-T(A) detected by flow cytometry in blood (and ascites, pleural effusion, if available)<br>• Assessment of blood tumor biomarkers<br>• Change of CLDN6 expression in tumor biopsy<br>• Immune signature of the tumor<br>• Anti-CAR antibodies, if clinically indicated
To characterize the in vivo cellular PK profile (levels, persistence, trafficking) of CLDN6 CAR-T/CLDN6 CAR-T(A)in target tissues (blood and other tissues if available)	• Presence of CLDN6 CAR-T/CLDN6 CAR-T(A) in blood and other tissues, if available<br>• Maximum concentration (Cmax), time to maximum concentration (Tmax), AUC and other relevant PK parameters of CLDN6 CAR-T/CLDN6 CAR-T(A) in blood<br>• Additionally for patients crossing-over from Part 1 to additional cohort in Part 1 (CLDN6 CAR-T/CLDN6 CAR-T(A) + CLDN6 RNA-LPX): re-expansion of CLDN6 CAR-T/CLDN6 CAR-T(A) in blood and other tissues

AUC, Area under the time-concentration curve; CAR, Chimeric antigen receptor; CAR-T, Chimeric antigen receptor T cell; CLDN6, Claudin 6; Cmax, Maximum concentration; CR, Complete response; DCR, Disease control rate; DLTs, Dose-limiting toxicities; DOR, Duration of response; iCPD, Immune confirmed progressive disease; IMP, Investigational Medicinal Product; iRECIST, Immune RECIST;

DocUUID : 6a803e66-f054-4d62-ad9b-00b24cc3317b

MTD, Maximal tolerated dose; ORR, Objective response rate; OS, Overall survival; PD, Progressive Disease; PFS, Progression-free survival; PK, Pharmacokinetics; PR, Partial response; qPCR, Quantitative polymerase chain reaction; RECIST, Response Evaluation Criteria in Solid Tumors; RNA-LPX, Liposomally formulated vaccine encoding ribonucleic acid; RP2D, Recommended Phase 2 dose; SD, Stable disease; TEAE, Treatment-emergent adverse event; Tmax, Time to reach maximal concentration; TTF, Time to treatment failure.
"i" indicates immune responses assigned using iRECIST.

DocUUID : 6a803e66-f054-4d62-ad9b-00b24cc3317b

3.2 Trial design

This is a FIH, open-label, multicenter Phase I/IIa dose escalation trial of Claudin 6 Chimeric antigen receptor T cell (CLDN6 CAR-T/CLDN6 CAR-T(A)) +/- Claudin 6 Liposomally formulated vaccine encoding ribonucleic acid (CLDN6 RNA-LPX) with expansion cohorts in patients with CLDN6 positive relapsed or refractory advanced solid tumors.

Patients with any of the following tumor types will be pre-screened to determine appropriate CLDN6 status: Testicular, ovarian, gastric, endometrial, NSCLC, advanced or metastatic cancer, including rare tumors, that fulfills other inclusion criteria and for whom there is no available standard therapy likely to confer clinical benefit, or patient is not a candidate for such available therapy. Only patients whose tumor exerts intermediate to strong CLDN6 antigen expression defined as ≥ 50% of tumor cells with ≥ 2+ CLDN6 staining per Immunohistochemistry (IHC) analysis in a central laboratory are eligible for this trial.

3.3 Schema (graphical representation of the trial)

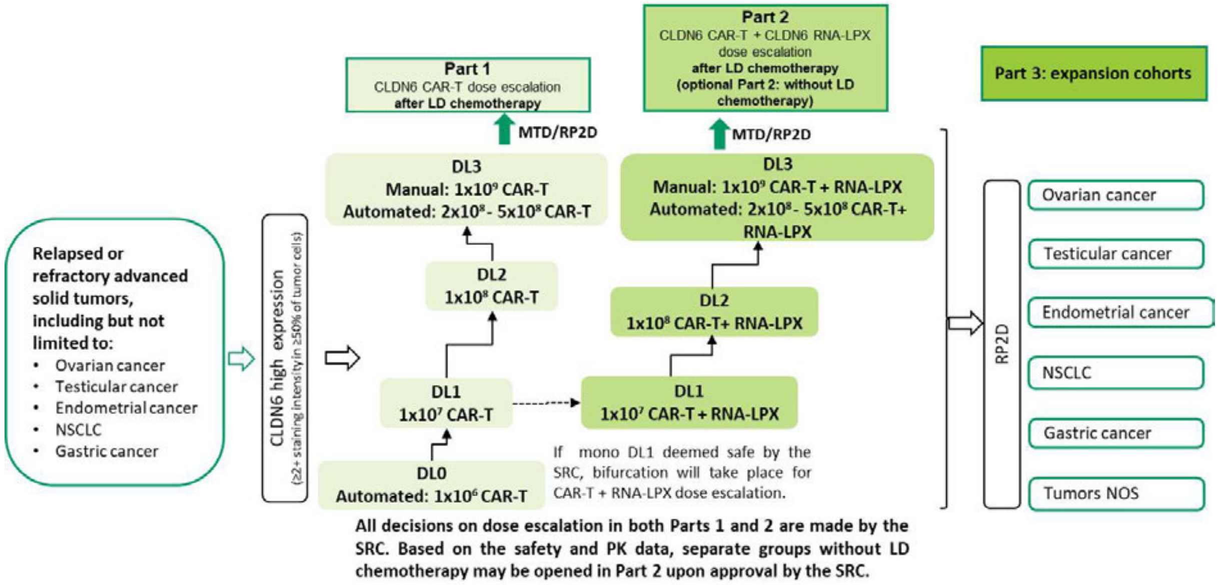

Figure 1 Trial schema

Abbreviations: CAR-T, chimeric antigen receptor T cell; CLDN6, Claudin 6; DL, dose level; LD, lymphodepletion; MTD, maximum tolerated dose; NOS, not otherwise specified; NSCLC, non-small cell lung cancer; RNA-LPX, liposomally formulated ribonucleic acid encoding the vaccine; RP2D, recommended Phase 2 dose; SRC, Safety Review Committee.

The trial consists of three parts as shown in below **Table 2**

DocUUID : 6a803e66-f054-4d62-ad9b-00b24cc3317b

Table 2 Trial design

	Part 1	Part 2	Part 3
**Trial design**	All patients will undergo leukapheresis for collection of blood product to manufacture the CLDN6 CAR-T /CLDN6 CAR-T(A). Repeat leukapheresis is allowed after discussion between the Investigator and the Sponsor's Medical Monitor.		
	In Parts 1 and 2, patients will receive one infusion of CLDN6 CAR-T/CLDN6 CAR-T(A) as described in Section 2.2.2 of the Protocol. In case CAR-T number is below the intended dose level, the patient will be treated and followed up per trial procedure, but replaced in the respective dose level cohort for DLT evaluation.		
	Part 1 will be a CLDN6 CAR-T/CLDN6 CAR-T(A) dose escalation in lymphodepleted patients until the MTD and/or RP2D of CLDN6 CAR-T/CLDN6 CAR-T(A) as monotherapy are defined.<br>Based on criteria in section 4.1 of the protocol, patients may cross over from Part 1 (CLDN6 CAR-T/ CLDN6 CAR-T(A)) to an additional cohort in Part 1 (CLDN6 CAR-T/ CLDN6 CAR-T(A) + CLDN6 RNA-LPX)	Part 2 will be a vaccine-modulated dose escalation in lymphodepleted patients until the MTD and/or RP2D of CLDN6 CAR-T/ CLDN6 CAR-T(A) + CLDN6 RNA-LPX are defined. Patients will be pretreated with an LD regimen (standard), or they may be pretreated with a reduced-dose LD regimen (optional**Error! Reference source not found.**) or without LD (optional), as decided by the SRC per SRC charter.<br>Part 2 is planned to start before the MTD/RP22D is reached in Part 1.	Part 3 will start only under the condition that safety data from Parts 1 and 2 indicate a continued positive benefit to risk ratio.
**Trial population**	Patients with CLDN6 positive relapsed or refractory advanced solid tumors (Only patients whose tumor exerts intermediate to strong CLDN6 antigen expression) defined as ≥ 50% of tumor cells with ≥ 2+ CLDN6 staining per IHC analysis in a central laboratory are eligible for this trial)		
**Investigational medical product(s)**	Name: CLDN6 CAR-T/CLDN6 CAR-T(A)<br>Dose: Flat dosing with starting dose for CLDN6 CAR-T (IMP-1) is 10^7 CLDN6 CAR-T , followed by two additional dose	Name: CLDN6 CAR-T/CLDN6 CAR-T(A)<br>Dose: Same as for Part 1<br>Schedule: Same as Part 1<br>Route of administration: Same as Part 1	Name: CLDN6 CAR-T/CLDN6 CAR-T(A) +/- CLDN6 RNA-LPX (RP2D)<br>Dose: This will be introduced through protocol amendment

DocUUID : 6a803e66-f054-4d62-ad9b-00b24cc3317b

<table>
<tr><td></td><td>levels of 10^8 and 10^9 CLDN6 CAR-T . The selected starting dose for CLDN6 CAR-T(A) (IMP-1(A)) is 10^6 CLDN6 CAR-T followed by three additional dose levels of 10^7, 10^8, and $2x10^8$ to $5x10^8$ CLDN6 CAR-T(A).

Schedule: Day 1
Route of administration: Intravenous
Re-dosing with CAR-T is allowed if requested by the investigator, the IMP is available, and endorsed by the SRC.

Name: CLDN6 RNA-LPX (For patients crossing over from Part 1 (CLDN6 CAR-T/ CLDN6 CAR-T(A)) to additional cohorts in Part 1 (CLDN6 CAR-T/CLDN6 CAR-T(A) + CLDN6 RNA-LPX)
Dose: Same as Part 2
Schedule: Same as Part 2
Route of administration: Same as Part 2</td><td>Re-dosing with CAR-T is allowed if requested by the investigator, the IMP is available, and endorsed by the SRC.

Name: CLDN6 RNA-LPX
Dose: The proposed starting dose of CLDN6 RNA-LPX cancer vaccine is 25 µg on day 4 in the standard Part 2 and in the optional Part 2 LD-free cohorts and on day 24 in the optional Part 2 cohort with LD. This will be decided based on the safety data by the SRC.
A step-up dose of 50 µg is administered on day 30 in the standard Part 2 and the optional Part 2 LD-free cohorts and on day 36 in the optional Part 2 cohort with LD. The same dose is administered in subsequent administrations if it is tolerated. If the starting dose of 25 µg is not tolerated, a dose of 12.5 µg will be explored. If 12.5 µg is tolerated, this will be administered on the remaining dosing days.
Schedule: Days 4, 30 or 36, 51, 72, 93 and every 6 weeks (+/-7d) until Month 22.
Route of administration: Intravenous venous catheter</td><td>Schedule: This will be introduced through protocol amendment
Route of administration: intravenous infusion.</td></tr>
<tr><td>Planned number of patients</td><td colspan="2">• The number of patients for Part 1 and Part 2 is driven by the 3+3 trial design
• Up to 18 Dose-Limiting Toxicities (DLT)-evaluable patients in each of Part 1 and Part 2 depending on the number of DLTs which may occur. After completion of Part 1 and Part 2 with CLDN6 CAR-T manufactured with a</td><td>The final sample size calculations will be introduced using a protocol amendment.</td></tr>
</table>

DocUUID : 6a803e66-f054-4d62-ad9b-00b24cc3317b

	manual process, the dose escalation will be repeated with CLDN6 CAR-T(A) manufactured with an automated process. According to the 3+3 design, up to 96 patients can be enrolled into the automated cohorts in Part 1 and Part 2.		
**Treatment and trial duration**	Patient Level:<br>The infusion of CLDN6 CAR-T/CLDN6 CAR-T(A) takes place only once on Day 1.<br>CLDN6 RNA-LPX (for patients crossing over to a cross-over cohort CLDN6 CAR-T + CLDN6 RNA-LPX in Part 1): Patients will receive treatment until one of the protocol pre-defined discontinuation of treatment criteria has been met (refer Section 6.6.5 of the protocol). For treatment beyond progression see section 6.1.3.2.1 of the protocol.<br>24 months of Primary follow-up from enrolment Optional LTFU of up to 15 years.	Patient Level:<br>The infusion of CLDN6 CAR-T/CLDN6 CAR-T(A) takes place only once on Day 1.<br>CLDN6 RNA-LPX: Patients will receive treatment until one of the protocol pre-defined discontinuation of treatment criteria has been met (refer Section 6.6.5 of the protocol). For treatment beyond progression see section 6.1.3.2.1 of the protocol.	Part 3 information will be provided through protocol amendment.
**Randomization and blinding**	This is a non-randomized, open-label trial		
**Tumor assessment schedule:**	Tumor assessment will be performed during screening, at 6 weeks (+/-7d) post infusion, then every 6 weeks (±7 d) for 50 weeks and every 12 weeks (±7 d) thereafter. Until disease progression is assessed by the investigator, the start of new anticancer therapy, withdrawal of consent, or death, whichever occurs first. Tumor response will be assessed by the investigator and will be evaluated according to RECIST v1.1 and iRECIST criteria.		This will be introduced through protocol amendment
**Other features**	After patients complete or prematurely discontinue participation in the trial, they will be asked to participate in a separate 15-year Long-term Follow-up (LTFU) trial (Refer Protocol Section 12.8 for LTFU details).		

In Part 1, in case the MTD for CLDN6 CAR-T is identified at dose level 1, the sponsor together with the Safety Review Committee (SRC) may explore lower dose levels for CLDN6 CAR-T dose escalation and for bifurcation from CAR-T to CAR-T + RNA-LPX dose escalation, based on safety and other available data.

Patients treated in Part 1 may be additionally treated with CDLN6 RNA-LPX under the following conditions:

- The dose level has been deemed safe by the SRC.
- The patient consents to the additional treatment in a separate consent form.
- The SRC approves the use of CLDN6 RNA-LPX based on the available safety and CLDN6 CAR-T expansion data of the respective patient.
- Safety criteria include:
    - No significant, unacceptable or irreversible toxicities related to trial treatment.
    - The Patients must meet the inclusion criteria for hematologic, renal, hepatic and coagulation functions.

Part 2 is planned to start before the MTD/RP2D is reached in Part 1 using a bifurcated trial design. Bifurcation is planned to start when Part 1 CLDN6 CAR-T/ CLDN6 CAR-T(A) dose level 1 (1×10^7CLDN6 CAR-T/ CLDN6 CAR-T(A)) evaluation is deemed safe by the SRC. At this point, Part 2 will start with the cleared CLDN6 CAR-T/ CLDN6 CAR-T(A) dose level. The dose level of CLDN6 CAR-T in Part 2 at any given time will not exceed that in Part 1.

In order to assess if LD-related toxicities can be avoided, cohorts testing vaccine-modulated CLDN6 CAR-T without LD may also be activated in patients with CLDN6-positive solid tumors, if satisfactory expansion and persistence is seen at a certain dose level. All decisions concerning opening the LD-free cohorts will be made by the SRC.

The Expansion Part (Part 3) is described in section 4.1.3 of the protocol. Additional expansion cohorts can be implemented by protocol amendment. The decision to expand is based on data generated in both Part 1 and Part 2. The expansion phase will assess safety and efficacy for defined cohorts. Further details can be seen in the protocol.

DocUUID : 6a803e66-f054-4d62-ad9b-00b24cc3317b

Dose Escalation in Part 1

The CLDN6 CAR-T/CLDN6 CAR-T(A) dose escalation follows the classical 3+3 design, see Appendix 6.

The next dose level cohort can only be opened when it is endorsed by the SRC (Additional details regarding the SRC can be found in the protocol). The DLT period is defined as 28 days starting from the day of CLDN6 CAR-T/CLDN6 CAR-T(A) therapy administration (day 1). During dose escalation, all patients will be hospitalized for 14 days for clinical observation after the administration of the CLDN6 CAR-T/CLDN6 CAR-T(A). Details are described in section 4.1.1.1 of the protocol.

The dose escalation with CLDN6 CAR-T manufactured with the manual process will evaluate CLDN6 CAR-T at three dose levels: 1×10^{7}CAR-T(total), 1×10^{8}CAR-T(total), and 1×10^{9}CAR-T(total), following the staggering rules outlined in Protocol Section 4.1.1.1.

The dose escalation with CLDN6 CAR-T(A) manufactured with the automated process will evaluate CLDN6 CAR-T(A) at four dose levels:$1x10^{6}$, $1x10^{7}$ CAR-T (total), $1x10^{8}$ CAR-T (total), and $2x10^{8}$ to $5x10^{8}$ CAR-T (total), following the staggering rules outlined in Protocol Section 4.1.1.1:

Dose Escalation in Part 2

The dose escalation in Part 2 follows the classical 3+3 design and the same principles including staggering. See Protocol Section 4.1.1.1 and Section 4.1.1.1.1 for staggering rules. However, in a case when any dose level in Part 2 is deemed unsafe while the Part 1 is ongoing, dosing in Part 2 should stop and the MTD is declared for the vaccine-modulated dose escalation. The CAR-T dose escalation in Part 1 can proceed independently of Part 2 until MTD and/or RP2D of CLDN6 CAR-T/CLDN6 CAR-T(A) are established. Refer to Protocol Section 6.6.5 for safety stopping criteria.

In Part 2, day 1 is considered the day when CLDN6 CAR-T/CLDN6 CAR-T(A) is administered. CLDN6 RNA-LPX is administered routinely on day 4 in the standard and LD-free cohort and on day 24 in the optional cohort with LD. This will be decided based on the safety data by the SRC. The DLT observation period in Part 2 is 28 days starting from day 1 until day 28. Hospitalization in Part 2 takes place from day 1 until day 14. Thereafter, the patient will be hospitalized as needed after each administration of CLDN6 RNA-LPX but at least until improvement of any Adverse Event (AE) to Grade 2 or lower. Details are described in section 4.1.1.2 of the protocol.

3.4 Schedule of activities

See Section 10.4 Appendix 4 for the schedule of activities.

4 STATISTICAL HYPOTHESES

The primary objectives of Parts 1 and 2 are to assess the safety profile and to identify the MTD and/or RP2D. Hence, no statistical hypothesis testing is planned for Parts 1 and 2.

5 INTERIM ANALYSES AND ANALYSIS SEQUENCE

No formal interim analysis is planned. However, data will be reviewed by the SRC after each cohort (details in the protocol).

6 SAMPLE SIZE DETERMINATION

The sample size for Part 1 and Part 2 is driven by the 3+3 trial design. In Part 1 and Part 2 the sample size will be up to 18 DLT-evaluable patients depending on the number of DLTs that may occur. After completion of Part 1 and Part 2 with CLDN6 CAR-T manufactured with a manual process, the dose escalation will be repeated with CLDN6 CAR-T(A) manufactured with an automated process. According to the 3+3 design, up to 96 patients can be enrolled into the automated cohorts in Part 1 and Part 2.

7 ANALYSIS SETS AND SUBGROUPS

7.1 Analysis sets

Screened Set

The Screened Set is defined as all patients who signed an informed consent.

Enrolled Set

The Enrolled Set is defined as all patients who have provided as leukapheresis.

Modified Intent to Treat Set

The modified Intent-to-treat (mITT) Set is defined as all patients who are assigned to IMP-1 or IMP-1(A) and have a baseline and at least one on-treatment/post treatment tumor assessment.

Safety Set

The Safety Set is defined as all patients who receive IMP-1 or IMP-1(A).

Per Protocol Set

DocUUID : 6a803e66-f054-4d62-ad9b-00b24cc3317b

The Per Protocol Set (PPS) is defined as all patients who receive IMP-1 or IMP-1(A) and fulfill the following criteria:

- The absence of any important protocol deviations
- The completion of a minimal exposure to the treatment
- Availability of baseline and at least one on-treatment/ post treatment tumor assessment

Important deviations will lead to an exclusion of patients from the PPS and will be agreed at the data review meeting prior to database snapshot for the primary analysis. Patients who have received an out of specification product as described in Section 6.1.2.3 of the Protocol will not be included in the PPS.

This analysis set may only be used in Part 3 of the trial.

DLT Evaluation Set

The DLT evaluation set includes all patients from the Safety Set who have

- experienced a DLT during the DLT evaluation period (28 days following first dose), or
- have completed the DLT evaluation period (28 days following first dose) and meet the minimum exposure criterion.

Patients who do not experience any DLT during the DLT observation period are considered to be evaluable if they have been observed for a minimum of 28 days following the first dose and are considered to have sufficient safety data to conclude that a DLT did not occur.

A patient is considered to have met the minimum exposure criterion if the patient receives the first full infusion of the CLDN6 CAR-T at the intended dose without interruption and modification in Part 1; and one full infusion of the CLDN6 CAR-T at the intended dose without interruption and modification and at least one full dose of vaccination in Part 2.

Patients who have received an out of specification product as described in Section 6.1.2.3 of the Protocol will not be included in the DLT.

Patients treated with non-conformant CLDN6 CAR-T products will be replaced and will not be considered for efficacy analysis. The safety data will be discussed with the SCR to obtain a full picture of the safety profile of the IMP, however will not be considered for dose decision meetings. In the study report, such data will be also clearly flagged in the safety set and separated from the per protocol data set. Patients will undergo all assessment as described in this clinical trial protocol and will also participate in the long-term follow up.

7.2 Protocol deviations

Protocol deviations are failures to adhere to the inclusion/exclusion criteria and protocol requirements and will be classified into important protocol deviations (IPDs) and non-important protocol deviations.

IPDs are a subset of protocol deviations that may significantly impact the completeness, accuracy, and/or reliability of the trial data or that may significantly affect a patient's rights, safety, or well-being. For example, IPDs may include enrolling patients in violation of key eligibility criteria designed to ensure a specific patient population or failing to collect data necessary to interpret primary endpoints, as this may compromise the scientific value of the trial.

- Non-Important protocol deviations (NIPDs) are those that are not considered to significantly affect the data collected and hence do not warrant patients' exclusion from an analysis set.
- IPDs will be identified by medical review prior to database snapshot for the main analysis.

The following criteria might be considered as IPDs not limited to. The list will be finalized before the data base lock:

- Violation of major inclusion or exclusion criteria
- Assignment to incorrect treatment/dose (i.e., actual treatment/dose taken differs from the scheduled)
- Non-compliance (i.e., relative dose intensity less than 80% or greater than 120%)
- Intake of prohibited concomitant medication

All protocol deviation will be presented in a listing. The number and percentage of patients excluded from the analysis sets will be summarized in total and by protocol deviation type.

7.3 Subgroups

There are no planned subgroup analyses.

8 STATISTICAL ANALYSES

8.1 General considerations

Unless otherwise specified, the details mentioned are for trial Part 1 and 2. The summaries will be presented by cohort, total and manufacturing process (manual or automated) within each trial part (Part 1 and Part 2). The presentation of data for Part 3 will be included in a SAP amendment. Moreover, cohorts may be combined as appropriate. In particular, the same dose levels of IMP-1 and IMP-1(A) may be combined, if both IMPs are deemed to be

DocUUID : 6a803e66-f054-4d62-ad9b-00b24cc3317b

comparable. This will be decided before the final analysis and will be documented in an update to the SAP.

No formal statistical comparisons between cohorts/trial parts will be performed.

Continuous variables will be summarized by cohort, total and manufacturing process (manual or automated) (as specified) using the following descriptive statistics: number of patients with non-missing data (n), mean, standard deviation (SD), median, minimum (min) and maximum (max).

Categorical variables will be summarized by cohort, total and manufacturing process (manual or automated) (as specified) presenting absolute and relative frequencies (n and %) of patients in each category and the number of patients with missing data ('missing' category will be presented if there is one or more missing value). For event-driven occurrence data (e.g. adverse event, concomitant medication, etc) the percentages will be based on the number of patients in the analysis set (N). For reported visit data (e.g. gender etc.) the percentages will be based on the number of patients with non-missing values (n). (N=xx) in the tables denotes the number of patients in the analysis set within each cohort.

The rates of binary endpoints such as efficacy variables (ORR and DCR) will be provided by cohort along with the corresponding 2-sided 95% Confidence Intervals (CI) using an exact method. Exact confidence intervals for binomial proportions will be derived using the Clopper-Pearson method[4].

Time-to-event-endpoints (DOR, PFS, TTF and OS) will be analyzed using Kaplan-Meier methodology by cohort and censored in accordance with the FDA Guidance: "Clinical Trial Endpoints for the Approval of Cancer Drugs and Biologics, 2018[5]". See Appendix 5 for censoring rules.

The number and type of events and the number and reason for censoring will be summarized (n, %) by cohort and manufacturing process (manual or automated) for each trial part. The median event/survival time in days (including two-sided 95% confidence limits according to Brookmeyer and Crowley, 1982[3]) and the first and third quartile will be presented for each cohort and manufacturing process (manual or automated). Survival rates (including two-sided 95% confidence interval based on Greenwood's formula) as well as the number and percentage of patients with events, censored and under risk will be displayed for selected time points 3, 6 and 12 months, with more time points added as appropriate.

The time-to-event analysis will be illustrated using Kaplan-Meier plots by cohort. The Kaplan-Meier plot will be presented 'going down', starting at 100% experiencing no events at the time of trial enrollment. The time to event analysis will be performed for a cohort in Part 1 and Part 2 and manufacturing process (manual or automated) if there are at least 10 patients in that cohort.

Tumor response will be assessed by investigator review of radiographic images and will be evaluated using RECIST 1.1 and iRECIST. iRECIST endpoints will be analyzed only if there

DocUUID : 6a803e66-f054-4d62-ad9b-00b24cc3317b

are relevant differences compared to the RECIST 1.1 endpoint i.e. if at least 10% of patients have different response for the iRECIST endpoint compared to RECIST 1.1 endpoint.

The best overall response (required for the analysis in Section 8.4.3 and 8.4.4) for the BICR assessment and investigator assessment (using RECIST 1.1 and iRECIST) will be programmatically determined using the RECIST 1.1 and iRECIST guidelines. A separate programming guideline will be used to program best overall response using RECIST 1.1 and iRECIST criteria. Confirmation of CR, PR, iCR and iPR is not required for this study.

See section 8.4 on how data for patients that have re-dosing and/or cross-over treatment are dealt with in the summary and analysis of efficacy data.

Data up to and including the clinical cut-off date will be taken into account for the statistical analysis.

The following will occur for the analysis of ORR, DCR, DOR, PFS, OS endpoints:

- All tumour assessment data and OS data from the main trial and LTFU (up to and including the clinical cut-off date) will be used for the analysis.

All data collected during the trial will be listed, the trial day will be presented as appropriate. The data will be sorted by trial part (Part 1, Part 2), manufacturing process (manual or automated) cohort, site number, patient number and date of assessment (if applicable).

All analyses will be conducted using SAS® version 9.4 or higher.

Baseline is defined as last available value prior to first dose of CLDN6 CAR-T/CLDN6 CAR-T(A).

Generally, if there are multiple assessments available for a particular day, the assessment that is closest to the day (and time if collected) of the first dose of a trial treatment (i.e. CLDN6 CAR-T/CLDN6 CAR-T(A)) will be used as the baseline value in the summary/analyses.

Multiple values at post-baseline visits: If there are multiple post-baseline assessments available within a visit/day, the earliest value and corresponding date/time will be considered.

Change from baseline: Unless otherwise specified, this will be calculated as follows:

Change from baseline = post-baseline assessment value – baseline assessment value.

If either the baseline or post-baseline assessment value is missing, the change from baseline is set to missing as well.

Body mass index (BMI) will be calculated as follows:

$$\text{BMI}\left(\frac{\text{kg}}{\text{m}^2}\right) = \frac{\text{Weight (kg)}}{\text{Height(m)}^2}$$

Duration (Other than "Trial Treatment Duration" defined in Section 8.5.1) will be calculated as follows:

- Duration = last observation date – first observation date + 1.

For conversion of days to months or years the following rules will be applied:

- 1 month = 30.25 days
- 1 year = 365.25 days

Trial Day and Treatment Day will be calculated for each trial part (1 and 2) as follows:

- Trial day:
  - If assessment date < treatment start date, then trial day = assessment date – treatment start date
  - If assessment date >= treatment start date, then trial day = assessment date – treatment start date + 1
- Treatment Day = treatment date – date of first dose +1

That is, trial day 1 indicates the date of treatment initiation.

Treatment Day will be calculated considering each treatment.

All trial data will be presented at the visit in the visit assigned in the Case Report Form (CRF)/visit assigned in external data. No windowing of data for the purposes of assigning to a visit will occur.

Unscheduled assessments will be included in the analysis of tumor assessment, time to event endpoints and Overall Survival. All other unscheduled assessments will be listed only and not included in summaries/analysis.

Handling missing Data:

For the purposes of assigning treatment-emergent flag for AEs, partial or missing AE dates will be handled as follows:

- If the day of the month is missing, the onset day will be set to the first day of the month unless it is the same month and year as trial treatment. In this case, in order to conservatively report the event as treatment-emergent, the onset date will be assumed to be the first date of trial treatment.
- If the onset day and month are both missing, the day and month will be assumed to be January 1, unless the event occurred in the same year as the trial treatment. In this case, the event onset will be assumed to be the day and month of treatment in order to conservatively report the event as treatment-emergent.
- A completely missing onset date will be assumed to be the first day of trial treatment.

DocUUID : 6a803e66-f054-4d62-ad9b-00b24cc3317b

- If an AE has a partial stop date that is in the same month/year as the first dose of trial treatment, then the AE will be reported as treatment–emergent. If an AE has a missing start date, then the AE will be reported as treatment–emergent.

For the purposes of assigning prior or concomitant flag for medications, partial or missing medication dates will be handled as follows:

- If end day is missing and month/year are non-missing, then day is the minimum of treatment end date and the last day of the month.
- If end day/month are missing and year is non-missing, then day is the minimum of treatment end date and the end of the year (31DECYYYY).
- If imputed end date is less than the start date, use the start date as the imputed end date.

Start dates:

- If the start date year is missing, the start date is set to one day prior to treatment start date.
- If the start date year is less than the treatment start date year, then:
    - If the month is missing, the start date is assumed to be mid-year point (01JULYYYY).
    - Else if the month is not missing, the start date is assumed to be mid-month point (15MONYYYY).
- If the start date year value is greater than the treatment start date year, then:
    - If the month is missing, the start date is assumed to be the year start point (01JANYYYY).
    - Else if the month is not missing, the start date is assumed to be the month start point (01MONYYYY).
- If the start date year value is equal to the treatment start date year value:
    - If the month is missing or the month is equal to the treatment start date month, then the start date is assumed to be one day prior to the treatment start date.
    - Else if the month is less than the treatment start date month, the start date is assumed to the mid-month point (15MONYYYY).

DocUUID : 6a803e66-f054-4d62-ad9b-00b24cc3317b

– Else if the month is greater than the treatment start date month, the start date is assumed to be the month start point (01MONYYYY).

If complete end date is available and the start date assumed from the steps above is greater than the end date, then the assumed start date should be set to the end date.

Missing data, other than that described for AEs/ Concomitant Medications (CMs)/ above will not be imputed.

8.2 Patient disposition

Unless otherwise specified, all the summaries will be presented by cohort, total and manufacturing process (manual or automated) within each trial part (Part 1 and Part 2).

For the screened patients, the number and percentage of patients having failed screening will be presented along with a summary of the primary reason for screening failure. A listing of patients that failed inclusion/exclusion criteria will also be presented.

The number and percentage of enrolled and treated patients will be presented by site including the number and percentage of IPDs (by cohort, total and manufacturing process (manual or automated) within each trial part).

The number and percentage of patients in each analysis set will be summarized by cohort, total and manufacturing process (manual or automated) within each trial part for the Enrolled Set.

For each analysis set (e.g. Safety Set, mITT Set, Per Protocol Set and DLT Evaluation Set) the number and percentage of patients excluded from the analysis set will be presented by cohort, total and manufacturing process (manual or automated) along with a summary of the reasons for exclusion based on the Enrolled Set.

The number and percentage of patients who are enrolled, without/reduced lymphodepletion, treated and non-treated with CLDN6 CAR-T/CLDN6 CAR-T(A) will be summarized along with the primary reason for missing administration of CLDN6 CAR-T/CLDN6 CAR-T(A). In addition, the number and percentage of patients who are still on treatment (CLDN6 RNA-LPX) and treated patients who are off-treatment will be summarized along with a summary of the primary reason for treatment discontinuation as reported in the eCRF. Patients who are “off-treatment” are the patients who are discontinued from treatment.

The number of patients that discontinued the trial together with the primary reason for discontinuation will be summarized. The total number of deaths will also be summarized.

In addition, for Part 1, the number of patients that cross-over to a CLDN6 CAR-T/CLDN6 CAR-T(A) + CLDN6 RNA-LPX cohort will also be summarized, together with the number of patients treated, not-treated, on and off treatment and all summaries will provided be by cohort, total and manufacturing process (manual or automated) within each trial part for Enrolled Set

DocUUID : 6a803e66-f054-4d62-ad9b-00b24cc3317b

A listing of patient disposition (including discontinuation from trial treatment and discontinuation from trial) will be presented.

A listing of Enrolled Set patients that are excluded from the Safety, DLT Evaluation, mITT or PP Set will be presented.

A listing of protocol deviations for the Enrolled Set will be provided.

8.3 Baseline characteristics

8.3.1 Demographics

Demographic data will be summarized by cohort and manufacturing process (manual or automated) for patients in the Safety Set for each part of the trial (Part 1 and Part 2).

Age (years), will be summarized as continuous data. Age category < 65, ≥ 65 years, Gender (male vs female), childbearing potential, race (White, Black or African American, Asian, American Indian or Alaska Native, Native Hawaiian or Other Pacific Islander, Not reportable, Unknown and other) and Ethnicity (Hispanic or Latino, Not Hispanic or Latino, Not reportable and Unknown) will be summarized as categorical data.

A listing of demographic data will be provided.

Baseline characteristics will be summarized by cohort and manufacturing process (manual or automated) for patients in the Safety Set for each part of the trial (Part 1 and Part 2).

Height (cm), weight (kg) at Screening and Body Mass Index (BMI (kg/m^2)) at Screening and BSA (body surface area) at Screening, will be summarized descriptively. BMI category (<18 kg/m^2 vs ≥18 kg/m^2, < 25 kg/m^2 vs. ≥ 25 kg/m^2, < 30 kg/m^2, ≥ 30 kg/m^2) will be summarized with frequency counts and percentage by cohort and manufacturing process (manual or automated).

A patient data listing will be provided.

8.3.2 Disease characteristics

The following disease characteristics at baseline will be summarized by cohort and manufacturing process (manual or automated) for patients in the Safety Set for each trial part (Part 1 and Part 2):

- Location at initial diagnosis
- Time from initial cancer diagnosis to enrollment (months),
- Tumor stage at diagnosis (American Joint Committee on Cancer Staging (AJCC))
- Tumor stage at baseline (AJCC)
- Eastern Cooperative Oncology Group (ECOG) Performance Status at baseline
- TNM stage at initial diagnosis

DocUUID : 6a803e66-f054-4d62-ad9b-00b24cc3317b

- TNM stage at baseline (rT, rN, rM, rTNM unknown)
- Histopathological type of cancer

Time from initial cancer diagnosis to enrollment (months) will be calculated as:

Time = date of enrollment – initial diagnosis date + 1.

In order to calculate the time from initial cancer diagnosis, the following rules will be applied for partial dates of first diagnosis:

- The first day of the month will be assumed;
- If the month is missing, January 1st will be assumed

8.3.3 Prior anti-cancer treatments

The summary of prior anti-cancer treatments will be based on the Safety Set and will be presented by cohort, total and manufacturing process (manual or automated) for each trial part (Part 1 and Part 2).

For prior systemic cancer therapy, the following will be summarized:

- The number of prior systemic therapies (0,1, 2, 3+)
- The number of prior systemic therapies (as continuous data)

The last prior systemic cancer therapy prior to enrollment into the trial (the prior systemic therapy with end date closest to enrollment date) will be summarized as follows:

- Setting (adjuvant, neoadjuvant, metastatic, other)
- Intent (curative, palliative and unknown)
- Best response at the end of the therapy (Complete Response (CR), Partial Response (PR), PD, Stable disease (SD), unknown)
- Reason for discontinuation of last prior systemic therapy (Toxicity/Intolerance, Disease progression, Completion of therapy. Adverse Event, Other, Unknown)

The treatment setting and intent for prior cancer radiotherapy and treatment intent for prior cancer surgery will be summarized by cohort, total and manufacturing process (manual or automated) for each trial part (Part 1 and Part 2).

Prior systemic cancer therapies will be coded using the World Health Organization Drug Dictionary (WHO DD) version B3 March 2020 resulting in Anatomical-Therapeutic-Chemical (ATC) codes indicating therapeutic classification.

The number and percentage of patients taking prior systemic cancer therapies will be summarized by ATC therapeutic class (ATC level 2), ATC pharmacological class (ATC level 3), and chemical substance (ATC level 5) for each cohort, total and manufacturing process (manual or automated) for each part of the trial (Part 1 and Part 2).

DocUUID : 6a803e66-f054-4d62-ad9b-00b24cc3317b

Prior cancer surgeries will be coded using the Medical Dictionary for Regulatory Activities (MedDRA®) coding system version 24.0 and will be listed only.

A listings of prior cancer therapies (systemic cancer therapy, cancer radiotherapy, cancer surgery) will be provided.

8.3.4 Prior and Concomitant medication/Procedures/Non-drug Therapy

Prior and concomitant medications will be defined using medication start and stop dates recorded, relative to the first and last dose of trial treatment (CLDN6 CAR-T/CLDN6 CAR-T(A)) for both the trial part (Part 1 and Part 2).

A prior medication will be defined as any medication taken 28 days prior to (but not including) the earliest start date of trial treatment (CLDN6 CAR-T/CLDN6 CAR-T(A), CLDN6 RNA-LPX).

A concomitant medication will be defined as:

- any medication either ongoing at the start of trial treatment in each trial part (1 and 2)
- or with a start date on or after the first dose of any trial treatment until 30 days after the last trial treatment administration (either CLDN6 CAR-T/ CLDN6 CAR-T(A) or CLDN6 RNA-LPX)
- or taken up to 90 days safety period for treatment of related AEs.

A separate summary and listing of antineoplastic medications started after CLDN6 CAR-T/ CLDN6 CAR-T(A) administration will be presented. See Section 8.1 for handling missing dates for concomitant medications.

Medications will be coded using the World Health Organization Drug Dictionary (WHODrug) drug codes of version B3 March 2022 or later. Resulting in Anatomical-Therapeutic-Chemical (ATC) codes indicating therapeutic classification.

The number and percentage of patients who had prior medications and concomitant medications will be summarized by ATC therapeutic class (ATC level 2), ATC pharmacological class (ATC level 3), and chemical substance (ATC level 5) for each cohort, total and manufacturing process (manual or automated) within each trial part (Part 1 and 2) based on the Safety Set. The summary will be presented alphabetically.

A listing of prior and concomitant medications will be provided.

Procedures and Non-drug therapies will be coded using the Medical Dictionary for Regulatory Activities (MedDRA®) coding system version 25.0 or later. Procedure and non-drug therapies will be listed only.

DocUUID : 6a803e66-f054-4d62-ad9b-00b24cc3317b

8.3.5 Medical history

Medical history data will be coded using Medical Dictionary for Regulatory Activities (MedDRA®) version 24.0 or later. The number and percentage of patients with each medical history will be summarized by System Organ Class (SOC) and Preferred Term (PT) for each cohort, total and manufacturing process (manual or automated) within each trial part based on the Safety Set.

A listing of medical history data will be provided.

8.4 Efficacy analyses

Unless otherwise stated, all summaries and analyses for efficacy data will use the mITT Set.

As noted in Section 4, there are no plans to test statistical hypotheses for this trial. All analyses will be exploratory in nature.

Patient data listings will be provided for efficacy data.

The censoring rules for analysis of time-to-event endpoints (DOR, PFS, OS and TTF) are listed in Appendix 5.

If a patient is re-dosed with CLDN6 CAR-T/ CLDN6 CAR-T(A) then only the data up until the day before re-dosing will be included in the summary and analysis of efficacy data. All data will be listed, the data collected on and after re-dosing will be flagged in the listings.

If a patient enrolls into cross-over treatment (see section 3.2) then all data will be included in the summary and analysis of efficacy data. All data will be listed, the data collected on and after first dose of cross-over treatment will be flagged in the listings.

8.4.1 Primary analysis

Not applicable for this trial as there is no primary efficacy endpoint defined for this trial. The primary endpoint is a safety endpoint and this endpoint is described in section 8.5.2.

8.4.2 Supplementary analyses

Not applicable.

8.4.3 Secondary analyses

Details for presentation and analysis of soluble immune factors in CLDN6 CAR-T/CLDN6 CAR-T(A) +/- CLDN6 RNA will be presented in a separate SAP.

The secondary efficacy endpoints are based on Investigator assessment using RECIST1.1 criteria and are defined as:

- ORR defined as the proportion of patients in whom a CR or PR (per RECIST 1.1) is observed as best overall response.

DocUUID : 6a803e66-f054-4d62-ad9b-00b24cc3317b

- DCR defined as the proportion of patients in whom a CR or PR or SD per RECIST 1.1 (SD assessed at least 6 weeks after first dose) is observed as best overall response.
- DOR defined as the time in months from first objective response (CR or PR per RECIST 1.1) to first occurrence of objective progression (PD) per RECIST 1.1 /recurrence or death from any cause, whichever occurs first.

The best overall response for a patient is defined as the best response across all time points.

Best overall response will be summarized using absolute and relative frequencies (n, %) by cohort and manufacturing process (manual or automated) for each trial part.

The ORR and DCR will be summarized using absolute and relative frequencies (n, %) along with two-sided 95% Clopper-Pearson confidence intervals by cohort and manufacturing process (manual or automated) for each trial part. Patients not meeting the criteria for CR or PR for ORR and CR or PR or SD for DCR, including those without any post-baseline tumor assessments, will be considered as non-responders.

A listing of tumor response assessment (ORR and DCR) will be provided.

DOR will be analyzed as a time to event endpoint using Kaplan-Meier methodology as described in Section 8.1 (Refer to Appendix 5 for censoring rules). Only patients in whom a CR or PR is observed will be analyzed for DOR. If too few (less than 10 patients in a cohort, with less than 5 of those patients with a CR or PR) are observed the analysis of DOR will be omitted.

A listing for DOR including time to event, event type and censoring information will be provided.

8.4.4 Exploratory analyses

The exploratory endpoints are based on Investigator assessment using iRECIST criteria and are defined as:

- iORR per iRECIST 1.1 defined as the proportion of patients in whom a iCR or iPR (per iRECIST) is observed as best overall response.
- iDCR (per iRECIST) defined as the proportion of patients in whom an iCR or iPR or iSD per iRECIST (iSD assessed at least 6 weeks after first dose) is observed as best overall response.
- iDOR defined as the time from first objective response (iCR or iPR per iRECIST) to first occurrence of objective progression (iCPD) per RECIST 1.1 /recurrence or death from any cause, whichever occurs first.

iDOR (response based on iRECIST) (days) = [date of first iUPD associated with confirmed iCPD /recurrence or death date – date of first objective response + 1]). iCPD is based on iRECIST criteria.

DocUUID : 6a803e66-f054-4d62-ad9b-00b24cc3317b

iORR, iCDR and iDOR will be analyzed only if there are relevant differences compared RECIST 1.1 endpoints (secondary endpoints in section 8.4.3) i.e. if at least 10% of patients have different response for iRECIST endpoints compared to RECIST 1.1 endpoints.

Best overall response will be summarized using absolute and relative frequencies (n, %) by cohort and manufacturing process (manual or automated) for each trial part.

The iORR and iDCR will be summarized using absolute and relative frequencies (n, %) along with two-sided 95% Clopper-Pearson confidence intervals by cohort and manufacturing process (manual or automated) for each trial part. Patients not meeting the criteria for iCR or iPR for iORR and iCR or iPR or iSD for iDCR, including those without any post-baseline tumor assessments, will be considered as non-responders.

A listing of tumor response assessment (ORR and DCR) will be provided

iDOR will be analyzed as a time to event endpoint using Kaplan-Meier methodology as described in Section 8.1 (Refer Appendix 5 for censoring rules). Only patients in whom a iCR or iPR is observed will be analyzed for iDOR. If too few (less than 10 patients in a cohort, with less than 5 of those patients with a iCR or iPR) are observed the analysis of iDOR will be omitted.

A listing for iDOR including time to event, event type and censoring information will be provided.

A preliminary assessment of efficacy will also be included based on evaluation of:

- Progression-free survival (PFS) defined as the time from first dose of CLDN6 CAR-T/CLDN6 CAR-T(A) to first objective PD per RECIST 1.1, or death from any cause, whichever occurs first.

PFS (days) = [date of iUPD associated with confirmed iCPD/death date – date of first dose of CLDN6 CAR-T/CLDN6 CAR-T(A) + 1]).

- PFS (response based on iRECIST) defined as the time from first dose of CLDN6 CAR-T to first objective iCPD, or death from any cause, whichever occurs first.

PFS (response based on iRECIST) (days) = [date of first iUPD associated with confirmed iCPD /death date – date of first dose of CLDN6 CAR-T cells + 1]). iCPD is based on iRECIST criteria.

- OS is defined as the time from first dose of CLDN6 CAR-T to death from any cause.

OS (days) = [Death date/Censor date – date of first dose of CLDN6 CAR-T/CLDN6 CAR-T(A) + 1].

- Time to treatment failure (TTF) in patients treated with CLDN6 RNA-LPX, defined as time from first injection to discontinuation of treatment for any reason including disease progression, treatment toxicity, add-on of new anti-

cancer therapy, and death. For patients that completed the treatment (as reported on the end of treatment page) TTF will be censored.

TTF (days) = [Treatment discontinuation date – date of first injection of CLDN6 RNA-LPX + 1]. Patients that received CLDN6 RNA-LPX in a Part 1 cross-over cohort will not be included in the analysis of this endpoint.

PFS (per RECIST 1.1 and iRECIST), OS and TTF will be analyzed as time to event endpoints using Kaplan-Meier methodology as described in Section 8.1. See Appendix 5 for censoring rules.

A listing will be provided that includes, time to event, event type and censoring information will be provided for each endpoint.

8.5 Safety analyses

Safety data that will be summarized includes treatment exposure, adverse events, clinical safety laboratory assessments (including Hematology, Clinical chemistry, Urinalysis, Coagulation Endocrine Tests), vital signs, electrocardiogram (ECGs), Eastern Cooperative Oncology Group Performance Status (ECOG PS), Physical examinations. All safety analyses will be based on the Safety Set and will be summarized by cohort, manufacturing process (manual or automated) and trial part and if appropriate, by cohort and treatment, for example for summary of treatment related AEs. For all the summaries patients will be assigned to the treatments according to the actual treatment they have received ("as treated") even it applies for patients with crossover treatment.

All safety data will be listed.

8.5.1 Extent of exposure

The following dose exposure variables will be derived and summarized for CLDN6 RNA-LPX (separately for Part 1 and Part 2) only, apart from actual cumulative dose, which will be calculated for CLDN6 CAR- T/CLDN6 CAR-T(A) (separately for Part 1 and Part 2). The summaries will be based on patients in the Safety Set and will be repeated for the DLT Evaluation Set. The summary for the DLT Evaluation Set will be restricted to the exposure data from the first dose till 28 days for the calculations of 'actual' values. For the calculation of planned treatment duration for the DLT Evaluation Set for the standard Part 2 this calculation will use data from the first dose (Day 4, first dose of LPX vaccination as per visit schedule) until the end of the DLT evaluation period (Day 28). Thus it is assumed that planned treatment duration is a maximum 25 days (number of weeks = 25/7).. If a patient discontinued before 25 days then the calculation is from first dose until date of EOT. For the calculation of planned treatment duration for the DLT Evaluation Set for the optional Part 2 (with LD) this calculation will use data from the first dose (Day 24, first dose of LPX vaccination as per visit schedule) until the end of the DLT evaluation period (Day 28). Thus it is assumed that planned treatment duration is a maximum 5 days (number of weeks = 5/7).. If a patient discontinued before 5 days then the calculation is from first dose until date

DocUUID : 6a803e66-f054-4d62-ad9b-00b24cc3317b

of EOT. Thus planned treatment duration is a maximum of 5 days. For planned cumulative dose for the DLT Evaluation Set it is assumed the dose is 25ug unless the patients discontinued (from study or treatment) prior to expected dosing day (Day 4 for standard Part 2 and Day 24 for Optional Part 2 (with LD)), or the data cut-off is prior to expected dosing day (Day 4 for standard Part 2 and Day 24 for Optional Part 2 (with LD)), in which case 0 is assigned.

- (Actual) Treatment Duration (weeks) defined as follows: (Date of last administration - Date of first administration + Planned Duration)/7, where the Planned Duration (weeks) is defined as the planned time between two consecutive administrations. Planned duration for CLDN6 RNA-LPX (for standard Part 2) will be calculated from the treatment schedule 'Day 4, Day 30, Day 51, day 72, Day 93 then every 6 weeks until month 22' (26 days for the second dose and then 21 days for all doses to day 93 then every 42 days until month 22. Planned duration for CLDN6 RNA-LPX (for optional Part 2 with LD) will be calculated from the treatment schedule Day 24, Day 36, Day 51, Day 73, Day 93 then every 6 weeks until month 22.
- Planned treatment duration (weeks) defined as (scheduled date of last administration - scheduled date of first administration + Planned Duration) / 7. The scheduled date of last administration is the date at which the last administration should be given according to the protocol schedule.
- (Actual) Cumulative Dose is defined as sum of all administered doses for each treatment. CLDN6 CAR-T/CLDN6 CAR-T(A) (mL) for Part 1 and CLDN6 CAR-T/CLDN6 CAR-T(A) (mL) and CLDN6 RNA-LPX (µg) for part 2. Cumulative Dose will be calculated for CLDN6 RNA-LPX (µg) in Part 1 if at least one patient receives cross-over CLDN6 RNA-LPX treatment.
- (Planned) Cumulative Dose for CLDN6 RNA-LPX (for standard Part 2), it is defined as the of sum of all planned doses at Day 4, Day 30, Day 51, day 72, Day 93 until month 22. See protocol section 6.1.3 for planned doses. For CLDN6 RNA-LPX (for optional Part 2 with LD), it is defined as the sum of all planned doses at Day 24, Day 36, Day 51, Day 73, Day 93 then every 6 weeks until month 22.
- Dose Intensity (DI) is defined as Cumulative Dose CLDN6 RNA-LPX (µg) / Treatment Duration (weeks).
- Relative Dose Intensity (RDI) is defined as follows:

$$\text{RDI }(\%) = \frac{\text{Actual Dose Intensity } \left(\frac{\text{mL}}{\text{weeks}}\right)}{\text{Planned Dose Intensity } \left(\frac{\text{mL}}{\text{weeks}}\right)} \text{ x } 100 = \text{DI x TI x } 100,$$

DocUUID : 6a803e66-f054-4d62-ad9b-00b24cc3317b

whereas

$$\text{(Actual) Dose Intensity}\left(\frac{\text{mL}}{weeks}\right) = \frac{\text{(Actual) Cumulative Dose (mL)}}{\text{(Actual) Treatment Duration (weeks)}}$$

$$\text{Planned Dose Intensity}\left(\frac{\text{mL}}{\text{weeks}}\right) = \frac{\text{Planned Cumulative Dose (mL)}}{\text{Planned Treatment Duration (weeks)}}$$

$$\text{Time Index (TI)} = \frac{\text{Planned Treatment Duration (weeks)}}{\text{Actual Treatment Duration (weeks)}}$$

The units in the derivations above are mL for CLDN6 CAR-T/ CLDN6 CAR-T(A) and µg for CLDN6 RNA-LPX µg.

The following variables will be presented with summary statistics for CLDN6 RNA-LPX (separately for Part 1 and Part 2) only, apart from cumulative dose, which will be presented for CLDN6 CAR- T/CLDN6 CAR-T(A). All will be presented by cohort and manufacturing process (manual or automated) for each trial part:

- Cumulative dose
- Dose intensity
- Relative dose intensity
- Treatment duration

Additionally, the relative dose intensity will be presented categorically (i.e., number and percentage of patients with relative dose intensity of <60%, 60-<80%, ≥80%). Moreover, the number and percentage of patients with any dose delay, dose modification or dose interruption (interruption is for CLDN6 CAR-T/CLDN6 CAR-T(A) only) will be presented separately for each trial treatment and manufacturing process (manual or automated) for each trial part.

All the above mentioned dose exposure variables will be presented in a by-patient listing.

A by-patient listing of treatment administration will be presented.

8.5.2 Adverse events

Adverse events (AEs) will be coded using the most recent version of Medical Dictionary for Regulatory Activities (MedDRA®) coding system version 24.0 or later to get a SOC and PT for each AE and graded for severity using National Cancer Institute - Common Terminology Criteria for Adverse Events (NCI CTCAE) v5.0 or later. In case a patient has an AE with missing relationship status, the event will be assumed to be related if the patient has received at least part of a dose of treatment and associated with the treatment received in the summaries (will be listed as collected in the listings). No imputation for missing NCI-CTC grades will be performed.

A Treatment Emergent Adverse Event (TEAE) is defined as any AE with an onset date on or after the first administration of trial treatment (CLDN6 CAR-T/CLDN6 CAR-T(A)) (if the AE was absent before the first administration of CLDN6 CAR-T/CLDN6 CAR-T(A)) or worsened after the first administration of trial treatment (if the AE was present before the first administration of CLDN6 CAR-T/CLDN6 CAR-T(A)). AEs with an onset date more than 90 days after the last administration of trial treatment will be considered as treatment-emergent only if assessed as related to trial treatment (CLDN6 CAR-T/CLDN6 CAR-T(A) or CLDN6 RNA-LPX) by the Investigator.

Treatment-emergent AEs and all AEs (All AEs are only considered when summarizing AEs related to Lymphodepletion(LD), Optional Chemotherapy, Trial Procedure (including Leukapheresis) will be summarized by cohort and manufacturing process (manual or automated) and trial part and total within trial part. In addition, treatment-related TEAEs will be summarized by cohort and manufacturing process (manual or automated) trial treatment (CLDN6 CAR-T/CLDN6 CAR-T(A) and CLDN6 RNA-LPX) within trial part.

Dose Limiting Toxicities

In general, a DLT for a drug or other treatment is defined as an AE that prevents an increase of the dose level of that treatment (for details on dose-escalation decision rules see Appendix 6).

Serious AEs (SAEs), non-serious (NS) Grade ≥3 AEs and clinically significant abnormal laboratory values Grade ≥3 will be collected and assessed for DLTs. Common Terminology Criteria for Adverse Events (NCI CTCAE) v.5.0 will be used to grade the intensity of AEs.

More details regarding DLTs can be found in the section 4.1.2 of the protocol.

For the additional rules and considerations of MTD and RP2D, see Section 4.1.1.1.1 of the protocol.

The number and percentage of patients reporting DLTs will be summarized by PT nested within SOC for each cohort manufacturing process (manual or automated) by trial part using the DLT Evaluable Set.

A separate listing of DLTs will be presented, including reported term, SOC, PT, time of onset, duration, outcome, relationship to trial treatment, NCI CTCAE grade, and seriousness including dose exposure data for day 1 to 28.

Overall summary of adverse events (AE)

The number and percentage of patients reporting at least one TEAE (unless otherwise specified) will be summarized for each of the following AE types by cohort manufacturing process (manual or automated) and trial part (Part 1 and Part 2).

For the Enrolled Set (all AEs)

- any AE

DocUUID : 6a803e66-f054-4d62-ad9b-00b24cc3317b

- AE related to Lymphodepletion Chemotherapy
- AE related to Optional Chemotherapy (bridging therapy)
- AE related to Trial Procedure (including Leukapheresis)
- SAE
- SAE related to Lymphodepletion Chemotherapy
- SAE related to Optional Chemotherapy (bridging therapy)
- SAE related to Trial Procedure (including Leukapheresis)

For the Safety Set

- TEAE
- TEAE related to CLDN6 CAR-T/CLDN6 CAR-T(A)
- TEAE related to CLDN6 RNA-LPX
- TEAE related to CLDN6 CAR-T/CLDN6 CAR-T(A) and CLDN6 RNA-LPX
- TEAE with NCI CTCAE grade ≥3
- TEAE related to CLDN6 CAR-T/CLDN6 CAR-T(A) NCI CTCAE grade ≥3
- TEAE related to CLDN6 RNA-LPX NCI CTCAE grade ≥3
- TEAE related to CLDN6 CAR-T/CLDN6 CAR-T(A) and CLDN6 RNA-LPX NCI CTCAE grade ≥3
- TEAE associated with CRS
- TEAE associated with CRS grade ≥3
- any TESAE
- TESAE related to CLDN6 CAR-T/CLDN6 CAR-T(A)
- TESAE related to CLDN6 RNA-LPX
- TESAE related to CLDN6 CAR-T/CLDN6 CAR-T(A) and CLDN6 RNA-LPX
- TESAE leading to death
- TESAE leading to death and related to CLDN6 CAR-T/CLDN6 CAR-T(A)
- TESAE leading to death Related to CLDN6 RNA-LPX
- TESAE leading to death Related to CLDN6 CAR-T/CLDN6 CAR-T(A) and CLDN6 RNA-LPX
- TEAEs leading to dose modification or interruption (interruption applicable for CLDN6 CAR-T/CLDN6 CAR-T(A) only)

- TEAE leading to permanent discontinuation of treatment (applicable only for CLDN6 RNA-LPX).
- TEAEs by worst NCI-CTC grade
- Related TEAEs by worst NCI-CTC grade
- TEAE related to CAR-T by worst NCI-CTC Grade
- TEAE related to RNA-LPX by worst NCI-CTC Grade
- DLTs

Analyses of adverse events

The number and percentage of patients reporting at least one TEAE will be summarized by PT nested within SOC for each of the above AE categories. If a SOC / PT is reported more than once for a patient, the patient will only be counted once for this SOC / PT. Strongest relationship or worst grade will be counted if a TEAE is reported more than once by the same patient for a SOC / PT.

All AE summary tables will be sorted alphabetically by SOC and PT within SOC. Unless specified otherwise.

SAEs and AEs related to LD chemotherapy/Bridging therapy/Trial Procedure (including Leukapheresis)

The number and percentage of patients with TEAEs related to LD chemotherapy/Bridging therapy/Trial Procedure (including Leukapheresis) will be summarized by PT nested within SOC. If an AE is reported more than once by the same patient for a System Organ Class (SOC) / Preferred Term (PT) the related AE will be counted once for this SOC / PT. The relationship will be considered as related if relationship status is reported as missing for an AE.

Treatment related TEAEs

The number and percentage of patients with TEAEs related to CLDN6 CAR-T/CLDN6 CAR-T(A), CLDN6 RNA-LPX and related to both CLDN6 CAR-T/CLDN6 CAR-T(A) and CLDN6 RNA-LPX will be summarized by PT nested within SOC. If an AE is reported more than once by the same patient for a System Organ Class (SOC) / Preferred Term (PT) the related AE will be counted once for this SOC / PT. The relationship to trial treatment will be considered as related if relationship status is reported as missing for an AE.

TEAE by NCI-CTC grade

The number and percentage of patients with TEAEs will be summarized by worst NCI-CTC grade by PT nested within SOC. Worst grade will be counted if an AE is reported more than once by the same patient for a SOC / PT.

The number and percentage of patients with NCI-CTC grade ≥ 3 AEs and NCI-CTC grade ≥ 3 related (CLDN6 CAR-T/CLDN6 CAR-T(A), CLDN6 RNA-LPX and both CLDN6 CAR-T/CLDN6 CAR-T(A) and CLDN6 RNA-LPX) AEs will be summarized by PT nested within SOC. In addition, AEs with a missing grade will be presented in the summary table with a grade category of “Missing.”

Serious Adverse Events and Other Significant Adverse Events

The number and percentage of patients

- with any Treatment Emergent Serious Adverse Event (TESAE),
- with any related TESAE (CLDN6 CAR-T/CLDN6 CAR-T(A), CLDN6 RNA-LPX and both CLDN6 CAR-T/CLDN6 CAR-T(A) and CLDN6 RNA-LPX)
- with any TESAE leading to death,
- with any TESAE Leading to death and related to treatment (CLDN6 CAR-T/CLDN6 CAR-T(A), CLDN6 RNA-LPX and both CLDN6 CAR-T/CLDN6 CAR-T(A) and CLDN6 RNA-LPX)
- with any TEAE leading to permanent discontinuation of treatment (CLDN6 RNA-LPX)
- with any TEAE leading to dose modification or interruption (interruption applicable for CLDN6 CAR-T/CLDN6 CAR-T(A) only)

will be summarized by PT nested within SOC by cohort and manufacturing process (manual or automated) within each trial part.

Deaths

All deaths and reasons for death (includes all deaths during the study) will be summarized by cohort and manufacturing process (manual or automated) for each trial part. All deaths will be listed, days since last dose will be displayed.

AE listings

All deaths, AEs, SAEs and TEAEs leading to permanent discontinuation of treatment, and discontinuation from trial will be listed. Also a listing of TEAEs leading to death will be provided. DLTs will be flagged in all AE listings.

The listings will be sorted by trial part, cohort and patient within cohort.

8.5.3 Laboratory assessments

Hematology, clinical chemistry, coagulation factors, endocrinology tests, tests of special interest (CRS, ICANS) and other screening tests will be performed by a central laboratory. Routine urinalysis will be performed by a local laboratory. Summaries of all laboratory data

DocUUID : 6a803e66-f054-4d62-ad9b-00b24cc3317b

will be based on Safety Set for each trial part (Part 1 and Part 2), total and manufacturing process (manual or automated) within each trial part.

For the purposes of summarizing and presentation in tables and listings, all laboratory values will be summarized by cohort, manufacturing process (manual or automated) and trial part and presented in System International (SI) units. If a laboratory value is reported using a nonnumeric qualifier e.g., less than (<) a certain value, or greater than (>) a certain value, the given numeric value will be used in the summary statistics, ignoring the nonnumeric qualifier. The data as collected will be presented in the listings.

Clinical laboratory data to be summarized includes hematology, clinical chemistry, urinalysis, coagulation factors, endocrine tests along with CRS and ICANS.

Clinical laboratory variables at each protocol scheduled visit and its change from baseline to each post-baseline protocol scheduled visit (for CLDN6 CAR-T/ CLDN6 CAR-T(A) infusion days and CLDN6 RNA-LPX injection days the value collected pre-infusion will be used for this summary) will be summarized using descriptive summary statistics for each parameter listed in the Protocol by cohort. Categorical variables will be summarized using n and %.

Clinical laboratory variables collected on CLDN6 CAR-T/CLDN6 CAR-T(A) infusion days/ CLDN6 RNA-LPX injection days and its change from baseline (pre-infusion/injection value at that visit) to each post-baseline time-point will be summarized for each time-point at each visit using descriptive summary statistics by cohort, manufacturing process (manual or automated) and trial part.

Abnormal and clinically significant, abnormal and not clinically significant and normal laboratory values will be summarized for each parameter by visit and treatment group.

All laboratory data will be presented in the data listings. Abnormal clinical laboratory values will be flagged in the listing.

Local laboratory data and Serology, FSH test and serum pregnancy results will be listed only.

8.5.4 Vital signs

Vital sign parameters to be summarized by cohort, manufacturing process (manual or automated) and trial part (Part 1 and Part 2) include weight, BSA, systolic and diastolic blood pressure, heart rate, body temperature, and oxygen saturation.

Vital sign parameters at each visit, and change from baseline to each post-baseline visit (for CLDN6 CAR-T/ CLDN6 CAR-T(A) infusion days and CLDN6 RNA-LPX injection days, the value collected pre-infusion/injection will be used for this summary) will be summarized using descriptive summary statistics for each parameter by cohort, manufacturing process (manual or automated) and for each trial part. Note that Vital signs will be collected both pre- and post-IMP administration.

DocUUID : 6a803e66-f054-4d62-ad9b-00b24cc3317b

Vital sign parameters collected on CLDN6 CAR-T/CLDN6 CAR-T(A) infusion days/ CLDN6 RNA-LPX injection days and its change from baseline (pre-infusion/injection value at that visit) to each post-baseline time-point will be summarized for each time-point at each visit using descriptive summary statistics by cohort, manufacturing process (manual or automated) and trial part.

All vital sign data will also be presented in a data listing.

8.5.5 ECG

A listing of ECG data will be provided.

8.5.6 Physical Examination

A complete physical examination will be performed at screening and the EoT Visit. At other visits, an abbreviated examination will be performed

The number and percentage of patients will be provided for Immune effector cell-associated encephalopathy (ICE) individual score along with total score (0-10).

A listing of physical examination will be presented.

8.5.7 ECOG Performance status

The ECOG performance status is a rating scale used to assess how a patient's disease is progressing as well as how the disease affects the daily living abilities of the patient. The score values are 0 – 5, where 0 indicates "fully active, able to carry on all pre-disease performance without restriction" and 5 indicates the patient has died.

ECOG will be summarised as categorical data by treatment group and visit. Additionally, a shift table from baseline to each visit by treatment will be provided.

A listing of ECOG performance status will also be provided.

8.5.8 Leukapheresis

A listing of leukapheresis details will be provided.

8.5.9 Lymphodepleting Chemotherapy/ Bridging Chemotherapy

Lymphodepleting and Bridging Chemotherapy data will be listed only.

8.5.10 Cytokine Release Syndrome (CRS)

The number and percentage of patients will be provided for CRS data (hypertension, hypoxia, fever). The highest severity for hypertension, hypoxia will be presented. For fever if a patients has at least one record of fever then they will be summarised in the fever category, else summarised as none. The summary will be repeated for CSR of grade ≥ 3.

The order of severity for hypertension is (least sever to most severe):

1. Not requiring vasopressors
2. Requiring vasopressors (excluding vasopressin)
3. Requiring vasopressors with vasopressin

The order of severity or Hypoxia is (least sever to most severe) is:

1. Requiring low-flow nasal cannula or blow-by
2. Requiring high-flow nasal cannula, facemask, nonrebreather mask or Venturi mask
3. Requiring positive pressure (e.g.,CPAP, BiPAP, intubation and mechanical ventilation)

A summary of grade for CSR will be presented, worst grade for each patient will be included in the summary.

For summary of TEAEs related to CSR see section 8.5.2.

A listing of Cytokine Release Syndrome (CRS) will also be provided.

8.5.11 Immune Effector Cell-Associated Neurotoxicity Syndrome (ICANS)

A listing will be provided for Immune Effector Cell-Associated Neurotoxicity Syndrome (ICANS).

8.6 Other analyses

8.6.1 Pharmacokinetics

Details for presentation and analysis of PK data will be presented in a separate SAP.

8.6.2 Pharmacodynamics and Biomarkers

Details for presentation and analysis of pharmacodynamics and biomarker data will be presented in a separate SAP

8.6.3 New Antineoplastic medications

New Antineoplastic medications after CLDN6 CAR-T/ CLDN6 CAR-T(A) infusion will be listed.

8.6.4 Hospitalizations

The number and percentage of patients with hospitalizations will be summarized. A listing will also be provided for patient hospitalization.

DocUUID : 6a803e66-f054-4d62-ad9b-00b24cc3317b

9 REFERENCES

1. International Conference on Harmonisation of Technical Requirements for Registration of Pharmaceuticals for Human Use (ICH) Guideline E3: Note for Guidance on Structure and Content of Clinical Study Reports (CPMP/ICH/137/95), July 1996. Retrieved on 30 January 2018 from http://www.ich.org/fileadmin/Public_Web_Site/ICH_Products/Guidelines/Efficacy/E3/E3_Guideline.pdf
2. International Conference on Harmonisation of Technical Requirements for Registration of Pharmaceuticals for Human Use (ICH) Guideline E9: Statistical Principles for Clinical Trials (CPMP/ICH/363/96), March 1998. Retrieved on 21 April 2019 from http://www.ich.org/fileadmin/Public_Web_Site/ICH_Products/Guidelines/Efficacy/E9/Step4/E9_Guideline.pdf
3. Brookmeyer R. and Crowley J. A confidence interval for the median survival time. Biometrics 38:29-41, 1982
4. Clopper, CJ and Pearson, ES. The use of confidence or fiducial limits illustrated in the case of the binomial. Biometrika 26: 404-413, 1934
5. Guidance for Industry: Clinical Trial Endpoints for the Approval of Cancer Drugs and Biologics. FDA December 2018.
6. Guidance for Industry Clinical Trial Endpoints for the Approval of Non-Small Cell Lung Cancer Drugs and Biologics, FDA April 2015
7. Eisenhauer, E.A., Therasse, P., Bogaerts, J., Schwartz, L.H., Sargent, D., Ford, R., Dancey, J.,Arbuck, S., Gwyther, S., Mooney, M., et al. (2009). New response evaluation criteria in solid tumours: revised RECIST guideline (version 1.1). Eur J Cancer 45, 228-247.

DocUUID : 6a803e66-f054-4d62-ad9b-00b24cc3317b

10 SUPPORTING DOCUMENTATION

10.1 Appendix 1: Changes to protocol-planned analyses

- Added clarity to the definition of the efficacy endpoint defined as ,'*PFS defined as the time from first dose of CLDN6 CAR-T to first objective iCPD, or death from any cause, whichever occurs first"* in the protocol. This is to distinguish it from the first efficacy endpoint progression free survival (PFS, defined as '*'the time from first dose of CLDN6 CAR-T to first objective PD per RECIST 1.1, or death from any cause, whichever occurs first*.'')
- Section 7.1: For definition of the DLT Evaluation Set, "DLT" is presented in place of "PPS" that is present in the protocol. "DLT" was intended and "PPS" was a typo in the protocol.
- Section 8.4.4: Added clarity for the definition of PFS, which is defined in the protocol as "time from first dose of CLDN6 CAR-T/CLDN6 CAR-T(A) to first objective iCPD". The endpoint will be calculated as "time from first dose of CLDN6 CAR-T/ CLDN6 CAR-T(A) to date of iUPD associated with confirmed iCPD".
- Section 7.1: The text has been removed from the definition of the mITT analysis set '*'Patients who have received an out of scope specification product as described in Section 6.1.2.3 of the Protocol will not be included in the mITT* '' the reason is that this text is not appropriate for mITT set

DocUUID : 6a803e66-f054-4d62-ad9b-00b24cc3317b

10.2 Appendix 2: List of abbreviations

AE	Adverse Event
AJCC	American Joint Committee on Cancer Staging
ATC	Anatomical Therapeutic Chemical
BMI	Body Mass Index
CAR	Chimeric antigen receptor
CAR-T	Chimeric antigen receptor T cell
CI	Confidence Interval
CLDN6	Claudin 6
CR	Complete Response
CRO	Contract Research Organization
CRS	Cytokin Release Syndrome
CV	Coefficient of Variation
DCR	Disease control rate
DL	Dose Level
DLT	Dose-Limiting Toxicities
DOR	Duration of response
ECG	Electrocardiogram
ECOG	Eastern Cooperative Oncology Group
eCRF	Electronic Case Report Form
EoT	End of Trial
FDA	Food and Drug Administration
GFR	Glomerular Filtration Rate
hrs	hours
ICANS	Immune Effector Cell-Associated Neurotoxicity Syndrome
iCPD	Immune confirmed progressive disease
IHC	Immunohistochemistry
IMP	Investigational medicinal product

DocUUID : 6a803e66-f054-4d62-ad9b-00b24cc3317b

IMP-1,	Investigational medicinal product 1 (manual process)
IMP-1(A),	Investigational medicinal product 1 (automated process)
IPD	Important Protocol Deviations
iRECIST	immune Response Evaluation Criteria in Solid Tumors
ITT	Intent-To-Treat
kg	kilogram
LD	Lymphodepletion
m^2	Meter square
max	Maximum
MedDRA®	Medical Dictionary for Regulatory Activities
mL	millilitre
min	Minimum
min	Minute
mITT	Modified Intent-To-Treat
MTD	Maximal tolerated dose
N	Number of Patients
n	Number of Observations
NCI-CTCAE	National Cancer Institute - Common Terminology Criteria for Adverse Events
NIPD	Non-Important Protocol Deviations
NOS	Not otherwise specified
NSCLC	Non-small cell lung cancer
NS	Non-serious
ORR	Objective Response Rate
OS	Overall Survival
PD	Progressive Disease
PFS	Progression-Free Survival
PK	Pharmacokinetics
PPS	Per Protocol Set
PR	Partial Response

PT	Preferred Term
qPCR	Quantitative polymerase chain reaction
RDI	Relative dose intensity
RECIST	Response Evaluation Criteria in Solid Tumors
RNA-LPX	Liposomally formulated vaccine encoding ribonucleic acid
RP2D	Recommended Phase 2 dose
SAE	Serious Adverse Event
SAP	Statistical Analysis Plan
SAS	Statistical Analysis Software
SD	Standard Deviation
SD	Stable Disease
SI	International System of Units
SOC	System Organ Class
SOP	Standard Operating Procedures
SoTp	Schedule of Trial Procedures
SRC	Safety Review Committee
TEAE	Treatment Emergent Adverse Event
TESAE	Treatment Emergent Serious Adverse Event
TLS	Tumor Lysis Syndrome
TMF	Trial Master File
TTF	Time to treatment failure
WHO DD	World Health Organisation Drug Dictionary

DocUUID : 6a803e66-f054-4d62-ad9b-00b24cc3317b

10.3 Appendix 3: Reporting conventions

SAS version 9.4, or higher, will be used to produce all tables, listings, and figures.

For summary statistics, the mean and median will be displayed to one decimal place greater than the original value and the measure of variability (e.g., SD) will be displayed to two decimal places greater than the original value. Minimum and maximum will be reported to the same decimal places as the original value. Percentages (%) will be displayed with one decimal place and 95% CIs & KM estimates will be displayed with one decimal place greater than the original value.

Cohort in the Table 3.1 will be used for the Tables/ Figures/ Listings display as appropriate:

DocUUID : 6a803e66-f054-4d62-ad9b-00b24cc3317b

Table 3.1 Trial Part and Cohort Descriptors

If the actual dose levels/cohort differ then the dose levels will still be labelled in ascending order and displayed as manual /automated as appropriate.

Trial Part	Cohort	Cohort Label	Cohort Label for Tables and Figures	Treatment Code
Part 1	Cohort 1	Automated: 1×10^{6} CLDN6 CAR-T	DL0	1
	Cohort 2	Manual: 1×10^{7} CLDN6 CAR-T<br>Automated: 1×10^{7} CLDN6 CAR-T	DL1	2
	Cohort 3	Manual: 1×10^{8} CLDN6 CAR-T<br>Automated: 1×10^{8} CLDN6 CAR-T	DL2	3
	Cohort 4	Manual: 1×10^{9} CLDN6 CAR-T<br>Automated: 2×10^{8} -5×10^{8} CLDN6 CAR-T	DL3	4
Part 2	Cohort 1	Manual: 1×10^{7} CLDN6 CAR-T + CLDN6 RNA-LPX<br>Automated: 1×10^{7} CLDN6 CAR-T + CLDN6 RNA-LPX	DL1	101
	Cohort 2	Manual: 1×10^{8} CLDN6 CAR-T +CLDN6 RNA-LPX<br>Automated: 1×10^{8} CLDN6 CAR-T +CLDN6 RNA-LPX	DL2	102

DocUUID : 6a803e66-f054-4d62-ad9b-00b24cc3317b

Trial Part	Cohort	Cohort Label	Cohort Label for Tables and Figures	Treatment Code
	Cohort 3	Manual: 1×10^9 CLDN6 CAR-T + CLDN6 RNA-LPX<br>Automated: 2×10^8 -5×10^8 CLDN6 CAR-T + CLDN6 RNA-LPX	DL3	103
	Cohort 7	Manual: CLDN6 CAR-T + CLDN6 RNA-LPX without Lymphodepletion	DL7	7

Note: The treatment code will be used in the statistical procedures.

10.4 Appendix 4: Schedule of activities

10.4.1 Dose Escalation (Parts 1 and 2)

Table 4.1, Table 4.2, Table 4.3, Table 4.4, Table 4.5, Table 4.6, Table 4.7 and Table 4.8 respectively, list all of the assessments to be performed in Parts 1 and 2 (dose escalation parts) of the trial.

DocUUID : 6a803e66-f054-4d62-ad9b-00b24cc3317b

Schedule of Activities – Part 1

Table 4.1: Part 1 (CLDN6 CAR-T/ CLDN6 CAR-T(A))

Part 1 SoA (CLDN6 CAR-T/CLDN6 CAR-T(A)) pre-screening, screening, apheresis, pre-treatment:

**Phase**	**Pre-screening for CLDN6 expression**	**Screening [1]**	**Apheresis**		**Pre-treatment**		
**Visit name**			**Leukapheresis**	**Post-leukapheresis FU**	**LD chemotherapy**		
**Trial period**	**Before screening**	**d-56 to d-18 [1]**	**d-18 (or earlier)**	**Within 3 d of leukapheresis**	**d-5**	**d-4**	**d-3**
Informed consent [2]	X	X					
**Patient history**							
Inclusion/exclusion criteria		X					
Demography [3]	X	X					
Medical history		X					
Diagnosis and extent of cancer	X	X					
Prior antineoplastic therapies		X					
Prior/concomitant therapies [4]		X	X	X	X	X	X
Height		X					
Body weight		X					
Physical examination [5]		X	X	X	X	X	X
Vital signs [6]		X	X		X	X	X
ECG		X			(X)	(X)	(X)
ECOG performance status		X	X				
Eligibility		X					
**Intervention**							
Leukapheresis screening (according to local standards)		X					

DocUUID : 6a803e66-f054-4d62-ad9b-00b24cc3317b

Phase	Pre-screening for CLDN6 expression	Screening [1]	Apheresis		Pre-treatment		
Visit name			Leukapheresis	Post-leukapheresis FU	LD chemotherapy		
Trial period	Before screening	d-56 to d-18 [1]	d-18 (or earlier)	Within 3 d of leukapheresis	d-5	d-4	d-3
Leukapheresis			X				
LD chemotherapy [7]					X	X	X
Optional chemotherapy [8]		As clinically indicated					
**Disease assessments**							
Archival tumor tissue [9]	X						
Fresh biopsy [10]	(X)	As clinically indicated					
Ascites/pleural effusion sample [11]		As clinically indicated					
Tumor assessment [12]		X			X [13]		
**Safety**							
Adverse events [14]		X	X	X	X	X	X
**Laboratory assessments**							
Serology/virology (HIV test, Hep B+C) (central lab)		X					
Hematology (central lab) [15]		X			X (pre)		
Clinical chemistry (central lab) [15]		X			X (pre)		
Coagulation (central lab)		X					
Endocrine tests (TSH, T3, and T4) (central lab)		X					
Lab test of special interest for CRS/ICANS (IL-6, CRP, ferritin, fibrinogen, procalcitonin) (central lab) [15,16]					X (pre)		
Urinalysis (local lab)		X					

DocUUID : 6a803e66-f054-4d62-ad9b-00b24cc3317b

Phase	Pre-screening for CLDN6 expression	Screening[1]	Apheresis		Pre-treatment		
Visit name			Leukapheresis	Post-leukapheresis FU	LD chemotherapy		
Trial period	Before screening	d-56 to d-18[1]	d-18 (or earlier)	Within 3 d of leukapheresis	d-5	d-4	d-3
Serum pregnancy test (local lab)[17]		X			X		
FSH and estradiol tests (central lab)[18]		X					
**Biomarker**							
Flow-phenotype[19]					X		
Tumor biomarker[19]					X		
Tumor tissue (molecular profiling)[20]		X					
HLA typing		X					

Abbreviations: CAR, chimeric antigen receptor; CAR-T, chimeric antigen receptor T cell; CLDN6, claudin 6; CRP, C-reactive protein; CRS, cytokine release syndrome; d, day; ECG, electrocardiogram; ECOG, Eastern Cooperative Oncology Group; EoT, End of Trial; FSH, follicle-stimulating hormone; FU, follow-up; h, hour; Hep B, hepatitis B virus; Hep C, hepatitis C virus; HIV, human immunodeficiency virus; ICANS, immune effector cell-associated neurotoxicity syndrome; ICF, informed consent form; IL 6, interleukin 6; LD, lymphodepleting; LDH, lactate dehydrogenase; m, month; T3, triiodothyronine; T4, thyroxine; TSH, thyroid-stimulating hormone; (X), as clinically indicated.

1) All screening assessments must be completed prior to leukapheresis: completion of screening assessments within a 4-week window is preferred.
2) The patient will sign one pre-screening ICF for CLDN6 expression evaluation at the Pre-screening Visit and the main ICF at the Screening Visit.
3) Year of birth/age, sex, ethnicity, race.
4) All therapies (including medications, non-drug therapies) being taken by the patient from screening until 90 d after last IMP treatment will be regarded as prior or concomitant therapies (newly started or ongoing during this time period). Afterwards only therapies corresponding to related AEs will be recorded.
5) A complete physical examination will be performed at screening and the EoT Visit. At other visits, an abbreviated examination will be performed and new or worsened clinically significant abnormalities will be recorded. Patients should be examined daily when hospitalized. All physical examinations should include the immune effector cell-associated encephalopathy (ICE) score assessment.
6) Vital signs (systolic and diastolic blood pressure, heart rate, body temperature, and oxygen saturation) will be measured in a seated or recumbent position after at least 5 min rest (please refer to Section 8.2.2 of the Protocol). Vital signs should be checked daily when the patient is hospitalized.
7) LD chemotherapy should be completed at least three days prior to the CLDN6 CAR-T/CLDN6 CAR-T(A) infusion. Prior to LD chemotherapy initiation, infections, cardiac functions, glomerular filtration rate, and neurological assessments should be measured (please refer to Section 6.1.1 of the Protocol). Only if the criteria apply, can the LD chemotherapy be initiated.
8) Refer to Section 6.5.2 of the Protocol for further information about documenting optional chemotherapy.
9) The formalin-fixed, paraffin-embedded (FFPE) sample should be from the most recent tumor tissue obtained. If no archival tumor tissue is available, the patient must be biopsied for CLDN6 staining at pre-screening. In addition, a baseline pre-treatment tumor sample (archival or fresh) is required.
10) At the Pre-screening Visit, patients must be biopsied if the most recent FFPE sample obtained is not available anymore. During the course of the trial fresh tumor biopsies should be taken at the discretion of the investigator, if this does not pose a significant risk for the patient.
11) Ascites and pleural effusion samples should be taken during the course of the trial at the discretion of the investigator, if this does not pose a significant risk for the patient.

DocUUID : 6a803e66-f054-4d62-ad9b-00b24cc3317b

12) Tumor assessment will be performed during screening, at 6 weeks (±7d) post infusion, then every 6 weeks (±7 d) for 50 weeks and every 12 weeks (±7 d) thereafter. Tumor response will be evaluated according to RECIST v1.1 and iRECIST.
13) Tumor assessment will be performed at d-5, if screening was more than 4 weeks ago and in case of bridging chemotherapy.
14) All AEs will be reported from signing of main ICF until 90 d after last IMP treatment. Afterwards only related AEs will be reported. Prior to signing of main ICF only SAEs related to a trial procedure will be reported.
15) At visit d-5, blood samples will be collected prior to the start of LD chemotherapy.
16) In case the lab test of special interest blood draw is not performed on the same visit as blood draw for hematology and clinical chemistry, blood sample for hematology and clinical chemistry need to be collected in addition.
17) Pregnancy tests will be performed for women of childbearing potential only. CAR-T dosing will only occur with a repeated negative pregnancy test (performed 5 d prior to CLDN6 CAR-T/CLDN6 CAR-T(A) administration).
18) FSH and estradiol tests will be performed at screening if indicated in post-menopausal females.
19) At visit d-5, blood samples will be collected prior to LD chemotherapy administration.
20) Collection of tumor tissue before treatment start, either the most recent archival FFPE material or a fresh biopsy is mandatory for molecular profiling (e.g., NGS, RNAseq, multiplex immune-profiling).

DocUUID : 6a803e66-f054-4d62-ad9b-00b24cc3317b

Part 1 SoA (CLDN6 CAR-T/CLDN6 CAR-T(A)) treatment, primary follow-up

**Phase**	**Treatment and primary follow-up**												**EoT & Primary FU** [1]
**Visit name**	**Infusion of CAR-T**	**Post infusion**											
**Trial period**	**d1**	**d2**	**d4 ±1d**	**d8 ±1d**	**d11 ±1d**	**d14 ±1d**	**d17 ±1d**	**d24 ±1d**	**d28 ±1d**	**d59, d87, d108 (=week16/m4) ±3d**	**d150, d192, d234, d276, d318, d360 ±7d**	**m15, m18, m21, m24 ±14d**	**m25 ±14d** [2]
**Patient history**													
Prior/concomitant therapies [3]	X	X	X	X	X	X	X	X	X	X	X	X	(X)
Body weight	X								X	Only d87 (m3)			X
Physical examination [4]	X	X	X	X	X	X	X	X	X	X	X	X	X
Vital signs [5]	X	X	X	X	X	X	X	X	X	X	X	X	X
ECG	(X)	(X)	(X)	(X)	(X)	(X)	(X)	(X)	(X)	(X)	(X)	(X)	(X)
ECOG Performance Status	X	X		X		X		X	X	X	X	X	X
Hospitalization status[6]	Hospitalization from d1-d14												
**Intervention**													
CLDN6 CAR-T/CLDN6 CAR-T(A) cell infusion [7]	X												
Documentation of antineoplastic therapy after CLDN6 infusion or trial discontinuation	X	X	X	X	X	X	X	X	X	X	X	X	X
**Disease assessment**													
Fresh biopsy [8]	As clinically indicated												
Ascites/pleural effusion sample [9]	As clinically indicated												
Tumor assessment [10]										Every 6 weeks (±7d) for 50 weeks, and every 12 weeks (±7d) thereafter			
**Safety**													
Adverse events [11]	X	X	X	X	X	X	X	X	X	X	X	X	(X)

DocUUID : 6a803e66-f054-4d62-ad9b-00b24cc3317b

Phase	Treatment and primary follow-up												EoT & Primary FU [1]
Visit name	Infusion of CAR-T	Post infusion											
Trial period	d1	d2	d4 ±1d	d8 ±1d	d11 ±1d	d14 ±1d	d17 ±1d	d24 ±1d	d28 ±1d	d59, d87, d108 (=week16/m4) ±3d	d150, d192, d234, d276, d318, d360 ±7d	m15, m18, m21, m24 ±14d	m25 ±14d [2]
**Laboratory assessments**													
Hematology (central lab) [12]	X (pre)	X	X	X	X	X	X	X	X	X	X	X	X
Clinical chemistry (central lab) [12]	X (pre)	X	X	X	X	X	X	X	X	X	X	X	X
Coagulation (central lab)	X (pre)												X
Endocrine tests (TSH, T3, and T4) (central lab)									X	X	X	X	X
Lab test of special interest for CRS/ICANS (IL-6, CRP, ferritin, fibrinogen, procalcitonin) (central lab) [13]	X (pre+6h post)	X	X	X	X	X	X	X	X				
Serum pregnancy test (local lab) [14]										X	X	X	X
**Biomarker [15]**													
Cytokines [15, 16]	X (pre)	X	d3&d4	X	X		X	X		d38, d45, d52, d59, d66, d73, d80, d94, d108 ±3d [16]	X		
qPCR incl. hematology [15,17]		X	d3&d4	X	X		X	X		d38, d45, d52, d59, d66, d73, d80, d94, d108 ±3d [17]	X	X	
Flow-phenotype [15]					X		X	X			X	X	
CAR characterization [15,18]							X			d45 (±3d), d87 (±3d)			
Tumor biomarker [15]					X		X	X		d38, d52, d66, d80, d94, d108	X	X	

DocUUID : 6a803e66-f054-4d62-ad9b-00b24cc3317b

**Phase**	**Treatment and primary follow-up**												**EoT & Primary FU [1]**
**Visit name**	**Infusion of CAR-T**	**Post infusion**											
**Trial period**	**d1**	**d2**	**d4 ±1d**	**d8 ±1d**	**d11 ±1d**	**d14 ±1d**	**d17 ±1d**	**d24 ±1d**	**d28 ±1d**	**d59, d87, d108 (=week16/m4) ±3d**	**d150, d192, d234, d276, d318, d360 ±7d**	**m15, m18, m21, m24 ±14d**	**m25 ±14d [2]**
CAR immunogenicity assay [15,19]	X (pre)							X		d94			
Tumor tissue (fresh) [20]		As clinically indicated											
**Survival status**	**For all patients who receive a CLDN6 CAR-T/CLDN6 CAR-T(A) infusion, patients may be enrolled into the long-term follow-up period for survival status as per schedule in Appendix 8 after the EoT Visit. If a patient misses a scheduled visit where survival status is required, survival status can be obtained via phone contact.**												

Abbreviations: CAR, chimeric antigen receptor; CAR-T, chimeric antigen receptor T cell; CLDN6, claudin 6; CRP, C-reactive protein; CRS, cytokine release syndrome; d, day; ECG, electrocardiogram; ECOG, Eastern Cooperative Oncology Group; EoT, End of Trial; FSH, follicle-stimulating hormone; FU: follow-up; h, hour; Hep B, hepatitis B virus; Hep C, hepatitis C virus; HIV, human immunodeficiency virus; ICANS, immune effector cell-associated neurotoxicity syndrome; ICF, informed consent form; IL 6, interleukin 6; INR, International normalized ratio; LDH, lactate dehydrogenase; (LT)FU, (long-term) follow-up; m, month; qPCR, quantitative polymerase chain reaction; T3, triiodothyronine; T4, thyroxine; TSH, thyroid-stimulating hormone; (X), as clinically indicated.

21) Refer to LTFU appendix for follow-up after trial completion.
22) All patients (if alive) who discontinue trial treatment will be evaluated at a Safety/EoT Follow-up Visit, at least 90 d and no more than 97 d after the last trial treatment administration. This visit will replace a regular visit if it is in the same visit window.
23) All therapies (including medications, non-drug therapies) being taken by the patient from screening until 90 d after last IMP treatment will be regarded as prior or concomitant therapies (newly started or ongoing during this time period). Afterwards only therapies corresponding to related Aes will be recorded.
24) A complete physical examination will be performed at screening and the EoT Visit. At other visits, an abbreviated examination will be performed and new or worsened clinically significant abnormalities will be recorded. Patients should be examined daily when hospitalized. All physical examinations should include the immune effector cell-associated encephalopathy (ICE) score assessment; the ICE score must be assessed daily within the hospitalization period.
25) Vital signs (systolic and diastolic blood pressure, heart rate, body temperature, and oxygen saturation) will be measured in a seated or recumbent position after at least 5 min rest (please refer to Section 8.2.2 of the Protocol). Vital signs should be checked daily when the patient is hospitalized.
26) Hospitalization can be shortened to d7 at the discretion of the SRC in case the patient shows no signs of CRS/ICANS. Prolonged hospitalization is allowed at the discretion of the investigator.
27) The CAR-T dose will be administered by i.v. infusion. At visit d1, assessments according to Section 6.1.2.2 of the Protocol should be made before CLDN6 CAR-T/CLDN6 CAR-T(A) administration. Only if the criteria apply, can the infusion of CLDN6 CAR-T/CLDN6 CAR-T(A) take place.
28) During the course of the trial fresh tumor biopsies should be taken at the discretion of the investigator, if this does not pose a significant risk for the patient.
29) Ascites and pleural effusion samples should be taken during the course of the trial at the discretion of the investigator, if this does not pose a significant risk for the patient.
30) Tumor assessment will be performed during screening, at 6 weeks (±7d) post infusion, then every 6 weeks (±7 d) for 50 weeks and every 12 weeks (±7 d) thereafter. Tumor response will be evaluated according to RECIST v1.1 and iRECIST.

31) All AEs will be reported from signing of main ICF until 90 d after last IMP treatment. Afterwards only related AEs will be reported. Prior to signing of main ICF only SAEs related to a trial procedure will be reported.
32) At visit d1, blood samples will be collected prior to infusion of CAR-T. At visit d4, blood samples will not be collected in case the visit falls on a weekend.
33) At visit d1, blood samples will be collected prior to infusion of CAR-T, and 6 h post infusion (± 1 h). If CRS/ICANS is suspected, parameters should be assessed daily by the central laboratory for the whole duration of the CRS/ICANS. As soon as the CRS/ICANS is over, parameters will be analyzed at the visits indicated in the SoA again (refer to Section 6.6.4.1 of the Protocol). At visit d4, blood samples will not be collected in case the visit falls on a weekend (blood collection on a weekend only in case of a CRS/ICANS).
34) Pregnancy tests will be performed for women of childbearing potential only. CAR-T dosing will only occur with a repeated negative pregnancy test (performed 5 d prior to CLDN6 CAR-T/CLDN6 CAR-T(A) administration).
35) After one year: The indicated biomarker assessments will only be performed if clinically indicated (after discussion with sponsor) – if CLDN6 CAR-T/CLDN6 CAR-T(A) are not detectable, biomarker assessments would cease with the exception of the qPCR assessment, and tumor biomarkers where relevant.
36) At visit d1, blood samples will be collected prior to infusion of CAR-T. Samples will be collected every 6 weeks after d108 until d356. At visit d4, blood samples will not be collected in case the visit falls on a weekend.
37) At visit d1, blood samples will be collected prior to infusion of CAR-T. A blood tube for hematology assessments must be taken with all samples for qPCR, if not already specified for this time point in the SoA. Samples will be collected every 6 weeks after d108 until d356, then every 3 m until d712. At visit d4, blood samples will not be collected in case the visit falls on a weekend.
38) For CAR characterization, the second and third time points should be aligned with the tumor assessments at 6 and 12 weeks. Up to three unscheduled additional blood sample collections are allowed if indicated by qPCR.
39) At visit d1, blood samples will be collected prior to infusion of CAR-T.
40) An on treatment fresh tumor biopsy should be taken at the discretion of the investigator, if this does not pose a significant risk for the patient, ideally around d17 and at progression for molecular profiling and to determine CLDN6 expression and CAR-T infiltration into the tumor tissue.

DocUUID : 6a803e66-f054-4d62-ad9b-00b24cc3317b

Table 4.2 Part 1 cross-over SoA (CLDN6 CAR-T/CLDN6 CAR-T(A) + CLDN6 RNA-LPX)

Part 1 cross-over SoA – treatment, primary follow-up for patients crossing over from Part 1 (CLDN6 CAR-T/CLDN6 CAR-T(A)) to additional cohort in Part 1 (CLDN6 CAR-T/CLDN6 CAR-T(A) + CLDN6 RNA-LPX)

Phase	Treatment and primary follow-up																EoT & Primary FU [1]
Visit name	CLDN6 RNA-LPX treatment phase																
Trial period	dx+ d4	dx+ d5 d6 d7	dx+ d8	dx+ d10	dx+ d17	dx+ d24 ±1d	dx+ d30-d35	dx+ d36, d43 ±1d	dx+ d51-d56	dx+ d57 ±1d	dx+ d72-d77	dx+ d78, d85 ±1d	dx+ d93-d98	dx+ d99 ±1d	dx+ d135, d177, d219, d261, d303, d345 [2], d387, d429, d471, d513, d555, d597, d639, d681 ±7d	Safety Visit [3]	dx+ m25 ±14d
**Patient history**																	
Prior/concomitant therapies [4]	X	X	X	X	X	X	X	X	X	X	X	X	X	X	(X)	X	(X)
Body weight							dx+d30									X	X
Physical examination [5]	X	X	X	X	X	X	X	X	X	X	X	X	X	X	X	X	X
Vital signs [6]	X	X	X	X	X	X	X	X	X	X	X	X	X	X	X	X	X
ECG	(X)	(X)	(X)	(X)	(X)	(X)	(X)	(X)	(X)	(X)	(X)	(X)	(X)	(X)	(X)	(X)	(X)
ECOG Performance Status	X				X		dx+d30	dx+d36	dx+d51	X	dx+d72	dx+d85	dx+d93	X	X	X	X
Hospitalization status [7]	dx+d4-d5 (24 h)																
**Intervention**																	
Vaccination of CLDN6 RNA-LPX [8]	X						dx+d30		dx+d51		dx+d72		dx+d93		Vaccinations every 6 weeks (±7d) until m22		
Documentation of antineoplastic therapy after CLDN6 infusion or trial discontinuation	X	X	X	X	X	X	X	X	X	X	X	X	X	X	X	X	X

DocUUID : 6a803e66-f054-4d62-ad9b-00b24cc3317b

**Phase**	**Treatment and primary follow-up**																**EoT & Primary FU** [1]
**Visit name**	**CLDN6 RNA-LPX treatment phase**																
**Trial period**	**dx+ d4**	**dx+ d5 d6 d7**	**dx+ d8**	**dx+ d10**	**dx+ d17**	**dx+ d24 ±1d**	**dx+ d30-d35**	**dx+ d36, d43 ±1d**	**dx+ d51-d56**	**dx+ d57 ±1d**	**dx+ d72-d77**	**dx+ d78, d85 ±1d**	**dx+ d93-d98**	**dx+ d99 ±1d**	**dx+ d135, d177, d219, d261, d303, d345** [2]**, d387, d429, d471, d513, d555, d597, d639, d681 ±7d**	**Safety Visit** [3]	**dx+ m25 ±14d**
**Disease assessment**																	
Fresh biopsy [9]		As clinically indicated															
Ascites/pleural effusion sample [10]		As clinically indicated															
Tumor assessment [11]								dx+d43 (±7d)	Week 6 (±7d) post infusion, every 6 weeks (±7d) for 50 weeks, and every 12 weeks (±7d) thereafter								
**Safety**																	
Adverse events [12]	X	X	X	X	X	X	X	X	X	X	X	X	X	X	(X)	(X)	(X)
**Laboratory assessments**																	
Hematology (central lab) [13]	X (pre)		X	X	X	X	dx+d30 (pre)	X	dx+d51 (pre)	X	dx+d72 (pre)	X	dx+d93 (pre)	X	X	X	X
Clinical chemistry (central lab) [13]	X (pre)		X	X	X	X	dx+d30 (pre)	X	dx+d51 (pre)	X	dx+d72 (pre)	X	dx+d93 (pre)	X	X	X	X
Coagulation (central lab)																	X
Endocrine tests (TSH, T3, and T4) (central lab)							dx+d30 (pre)						dx+d93 (pre)		X	X	X
Lab test of special interest for CRS/ICANS (IL 6, CRP, ferritin, fibrinogen, procalcitonin) (central lab) [14]	X (pre, 6h)		X	X	X	X	dx+d30 (pre)	X	dx+d51 (pre)	X	dx+d72 (pre)	X	dx+d93 (pre)	X			
Serum pregnancy test (local lab) [15]										X				X	X	X	X

DocUUID : 6a803e66-f054-4d62-ad9b-00b24cc3317b

**Phase**	**Treatment and primary follow-up**															**EoT & Primary FU [1]**
**Visit name**	**CLDN6 RNA-LPX treatment phase**															
**Trial period**	**dx+ d4**	**dx+ d5 d6 d7**	**dx+ d8**	**dx+ d10**	**dx+ d17**	**dx+ d24 ±1d**	**dx+ d30-d35**	**dx+ d36, d43 ±1d**	**dx+ d51-d56**	**dx+ d57 ±1d**	**dx+ d72-d77**	**dx+ d78, d85 ±1d**	**dx+ d93-d98**	**dx+ d99 ±1d**	**dx+ d135, d177, d219, d261, d303, d345 [2], d387, d429, d471, d513, d555, d597, d639, d681 ±7d**	**Safety Visit [3]**	**dx+ m25 ±14d**
**Biomarker [16]**																	
Cytokines [16,17]	X (pre, 6h, 24h)		X	X	X	X	dx+d30(pre, 6h, 24h)	dx+d36 (±1d)	dx+d51 (pre, 6h, 24h)	X	dx+d72 (pre, 6h, 24h)	dx+d78 (±1d)	dx+d93 (pre, 6h, 24h)	X	dx+d135 (pre, 6h, 24h), dx+d141, then every 6/7 weeks (±7d) [18]		
qPCR (incl. hematology) [17,18]	X (pre)		X	X	X	X	dx+d30(pre)	dx+d36 (±1d) (±1d)	dx+d51 (pre)	X	dx+d72 (pre)	dx+d78 (±1d)	dx+d93 (pre)	X	dx+d135 (pre), dx+d141, then every 6/7 weeks (±7d) [19]		
Flow-phenotype [17,19]				X	X	X											
CAR characterization [17,20]					X			dx+d43 (±1d)				dx+d85 (±1d)					
Tumor biomarker [17,21]				X		X		dx+d36 (±1d)	dx+d51 (pre)		dx+d72 (pre)		dx+d93 (pre)		dx+d135 (pre) (±7d)		
CAR immunogenicity assay [17,21]						X							dx+d94				
Tumor tissue (fresh) [22]	As clinically indicated																
**Survival status**	**For all patients who receive a CLDN6 CAR-T/CLDN6 CAR-T(A) infusion, patients are enrolled into the long-term follow-up period for survival status as per schedule in Appendix 8 after the EoT Visit. If a patient misses a scheduled visit where survival status is required, survival status can be obtained via phone contact.**																

Abbreviations: AE, adverse event; CAR, chimeric antigen receptor; CAR-T, chimeric antigen receptor T cell; CLDN6, claudin 6; CRP, C-reactive protein; CRS, cytokine release syndrome; d, day; ECG, electrocardiogram; ECOG, Eastern Cooperative Oncology Group; EoT, End of Trial; FSH, follicle-stimulating hormone; FU, follow-up; h, hour; Hep B, hepatitis B virus; Hep C, hepatitis C virus; HIV, human immunodeficiency virus; ICANS, immune effector cell-associated neurotoxicity syndrome; ICF, informed consent form; IL 6, interleukin 6; INR, international normalized ratio; LDH, lactate dehydrogenase; (LT)FU,

(long-term) follow-up; m, month; qPCR, quantitative polymerase chain reaction; RNA-LPX, liposomally formulated ribonucleic acid encoding the vaccine; T3, triiodothyronine; T4, thyroxine; TSH, thyroid-stimulating hormone; (X), as clinically indicated.

1) Refer to LTFU appendix for follow-up after trial completion.
2) If at m12/d345 vaccinations cease, visits switch from every 6 weeks to quarterly (m15, m18, m21, m24).
3) All patients (if alive) who discontinue trial treatment will be evaluated at a Safety/EoT Follow-up Visit at least 90 d and no more than 97 d after the last trial treatment administration. This visit will replace a regular visit if it is in the same visit window.
4) All therapies (including medications, non-drug therapies) being taken by the patient from screening until 90 d after last IMP treatment will be regarded as prior or concomitant therapies (newly started or ongoing during this time period). Afterwards only therapies corresponding to related AEs will be recorded.
5) A complete physical examination will be performed at screening and the EoT Visit. At other visits, an abbreviated examination will be performed and new or worsened clinically significant abnormalities will be recorded. Patients should be examined daily when hospitalized. On CLDN6 RNA-LPX vaccination days, patients should be examined prior to and 4-6 h following vaccination. All physical examinations should include the ICE score; the ICE score must be assessed daily within the hospitalization period.
6) Vital signs (systolic and diastolic blood pressure, heart rate, body temperature, and oxygen saturation) will be measured in a seated or recumbent position after at least 5 min rest (please refer to Section 8.2.2 of the Protocol), and should be measured daily if the patient is hospitalized. On CLDN6 RNA-LPX vaccination days, vital signs should be checked prior to and 4-6 h following vaccination.
7) Hospitalization from dx+d4 until dx+d5 after cross-over (24 h). Thereafter, after each administration of CLDN6 RNA-LPX, the patient will be hospitalized as needed, but at least until improvement of any adverse event to Grade 2 or lower. Prolonged hospitalization is allowed at the discretion of the investigator.
8) The vaccination will be administered as a slow i.v. bolus injection. Further vaccinations after dx+d93 are given every 6 weeks until m22. Refer to Section 6.1.3 of the Protocol for further information.
9) During the course of the trial fresh tumor biopsies should be taken at the discretion of the investigator, if this does not pose a significant risk for the patient.
10) Ascites and pleural effusion samples should be taken during the course of the trial at the discretion of the investigator, if this does not pose a significant risk for the patient.
11) Tumor assessment will be performed during screening, at 6 weeks (±7 d) post infusion, then every 6 weeks (±7 d) for 50 weeks and every 12 weeks (±7 d) thereafter. Tumor response will be evaluated according to RECIST v1.1 and iRECIST.
12) All AEs will be reported from signing of the main ICF until 90 d after last IMP treatment. Afterwards only related AEs will be reported. Prior to signing of main ICF only SAEs related to a trial procedure will be reported.
13) At all RNA-LPX vaccination visits, blood samples will be collected prior to vaccination. At visit d5,6,7 blood samples will not be collected in case the visit falls on a weekend (blood collection on a weekend only in case of a CRS/ICANS).
14) At visit dx+d4 blood samples will be collected prior to infusion of CAR-T, and 6 h (±1 h) post infusion. If CRS/ICANS is suspected, the parameters should be assessed daily by the central laboratory for the whole duration of the CRS/ICANS. As soon as the CRS/ICANS is over, parameters will be analyzed at the visits indicated in the SoA again (refer to Section 6.6.4.1 of the Protocol).
15) Pregnancy tests will be performed for women of childbearing potential only.
16) After one year: The indicated biomarker assessments will only be performed if clinically indicated (after discussion with sponsor) – if CLDN6 CAR-T/CLDN6 CAR-T(A) are not detectable, biomarker assessments would cease with the exception of the qPCR assessment, and tumor biomarkers where relevant.
17) On RNA-LPX administration visits, blood samples will be collected prior to administration of RNA-LPX. After dx+d141, every 6 weeks (pre- and 6 h, and 1 week after) RNA-LPX vaccination, if no RNA-LPX vaccination, then together with tumor assessment.
18) On RNA-LPX administration visits, blood samples will be collected prior to administration of RNA-LPX. A blood tube for hematology assessments must be taken with all samples for qPCR, if not already specified for this time point in the SoA. After dx+d141, every 6 weeks (pre- and 1 week after) RNA-LPX vaccination, if no RNA-LPX vaccination, then together with tumor assessment.
19) On RNA-LPX administration visits, blood samples will be collected prior to administration of RNA-LPX. After dx+d141, every 6 weeks (pre- and 1 week after) RNA-LPX vaccination, if no RNA-LPX vaccination, then together with tumor assessment.
20) For CAR characterization, the second and third time points should be aligned with the tumor assessments at 6 and 12 weeks. Up to three unscheduled additional blood sample collections are allowed if indicated by qPCR.

DocUUID : 6a803e66-f054-4d62-ad9b-00b24cc3317b

21) On RNA-LPX administration visits, blood samples will be collected prior to administration of RNA-LPX.
22) An on treatment fresh tumor biopsy should be taken at the discretion of the investigator, if this does not pose a significant risk for the patient, ideally around d17 and at progression for molecular profiling and to determine CLDN6 expression and CAR-T infiltration into the tumor tissue.

Table 4.3: Standard Part 2 SoA (CLDN6 CAR-T/CLDN6 CAR-T(A) + CLDN6 RNA-LPX)

Standard Part 2 SoA - pre-screening, screening, apheresis, pre-treatment

Phase	Pre-screening for CLDN6 expression	Screening [1]	Apheresis		Pre-treatment		
Visit name			Leukapheresis	Post-leukapheresis FU	LD chemotherapy		
Trial period	Before screening	d-56 to d-18 [1]	d-18 (or earlier)	Within 3 d of leukapheresis	d-5	d-4	d-3
Informed consent [2]	X	X					
**Patient history**							
Inclusion/exclusion criteria		X					
Demography [3]	X	X					
Medical history		X					
Diagnosis and extent of cancer	X	X					
Prior antineoplastic therapies		X					
Prior/concomitant therapies [4]		X	X	X	X	X	X
Height		X					
Body weight		X					
Physical examination [5]		X	X	X	X	X	X
Vital signs [6]		X	X		X	X	X
ECG		X			(X)	(X)	(X)

DocUUID : 6a803e66-f054-4d62-ad9b-00b24cc3317b

Phase	Pre-screening for CLDN6 expression	Screening[1]	Apheresis		Pre-treatment		
Visit name			Leukapheresis	Post-leukapheresis FU	LD chemotherapy		
Trial period	Before screening	d-56 to d-18[1]	d-18 (or earlier)	Within 3 d of leukapheresis	d-5	d-4	d-3
ECOG performance status		X	X				
Eligibility		X					
**Intervention**							
Leukapheresis screening (according to local standards)		X					
Leukapheresis			X				
LD chemotherapy[7]					X	X	X
Optional chemotherapy[8]		As clinically indicated					
**Disease assessments**							
Archival tumor tissue[9]	X						
Fresh biopsy[10]	(X)	As clinically indicated					
Ascites/pleural effusion sample[11]		As clinically indicated					
Tumor assessment[12]		X			X[13]		
**Safety**							
Adverse events[14]		X	X	X	X	X	X
**Laboratory assessments**							
Serology/virology (HIV		X					

DocUUID : 6a803e66-f054-4d62-ad9b-00b24cc3317b

Phase	Pre-screening for CLDN6 expression	Screening[1]	Apheresis		Pre-treatment		
Visit name			Leukapheresis	Post-leukapheresis FU	LD chemotherapy		
Trial period	Before screening	d-56 to d-18 [1]	d-18 (or earlier)	Within 3 d of leukapheresis	d-5	d-4	d-3
test, Hep B+C) (central lab)							
Hematology (central lab) [15]		X			X		
Clinical chemistry (central lab) [15]		X			X		
Coagulation (central lab)		X					
Endocrine tests (TSH, T3, and T4) (central lab)		X					
Lab test of special interest for CRS/ICANS (IL-6, CRP, ferritin, fibrinogen, procalcitonin) (central lab) [15,16]					X		
Urinalysis (local lab)		X					
Serum pregnancy test (central lab) [17]		X			X		
FSH and estradiol tests (central lab) [18]		X					

DocUUID : 6a803e66-f054-4d62-ad9b-00b24cc3317b

Phase	Pre-screening for CLDN6 expression	Screening[1]	Apheresis		Pre-treatment		
Visit name			Leukapheresis	Post-leukapheresis FU	LD chemotherapy		
Trial period	Before screening	d-56 to d-18[1]	d-18 (or earlier)	Within 3 d of leukapheresis	d-5	d-4	d-3
**Biomarker**							
Flow-phenotype[19]					X (pre)		
Tumor biomarker[19]					X (pre)		
Tumor tissue (molecular profiling)[20]		X					
HLA typing		X					

Abbreviations: AE, adverse event; CAR, chimeric antigen receptor; CAR-T, chimeric antigen receptor T cell; CLDN6, claudin 6; CRP, C-reactive protein; CRS, cytokine release syndrome; d, day; ECG, electrocardiogram; ECOG, Eastern Cooperative Oncology Group; EoT, End of Trial; FSH, follicle-stimulating hormone; FU, follow-up; h, hour; Hep B, hepatitis B virus; Hep C, hepatitis C virus; HIV, human immunodeficiency virus; ICANS, immune effector cell-associated neurotoxicity syndrome; ICF, informed consent form; IL 6, interleukin 6; INR, International normalized ratio; LD, lymphodepleting; LDH, lactate dehydrogenase; (LT)FU, (long-term) follow-up; m, month; qPCR, quantitative polymerase chain reaction; RNA-LPX, liposomally formulated ribonucleic acid encoding the vaccine; T3, triiodothyronine; T4, thyroxine; TSH, thyroid-stimulating hormone; (X), as clinically indicated.

1) All screening assessments must be completed prior to leukapheresis: completion of screening assessments within a 4-week window is preferred.
2) The patient will sign one pre-screening ICF for CLDN6 expression evaluation at the Pre-screening Visit and the main ICF at the Screening Visit.
3) Year of birth/age, sex, ethnicity, race.
4) All therapies (including medications, non-drug therapies) being taken by the patient from screening until 90 d after last IMP treatment will be regarded as prior or concomitant therapies (newly started or ongoing during this time period). Afterwards only therapies corresponding to related AEs will be recorded.
5) A complete physical examination will be performed at screening and the EoT Visit. At other visits, an abbreviated examination will be performed and new or worsened clinically significant abnormalities will be recorded. Patients should be examined daily when hospitalized. All physical examinations should include the ICE score assessment.
6) Vital signs (systolic and diastolic blood pressure, heart rate, body temperature, and oxygen saturation) will be measured in a seated or recumbent position after at least 5 min rest (refer to Section 8.2.2 of the Protocol), and should be checked daily when the patient is hospitalized.
7) LD chemotherapy should be completed at least three days prior to the CLDN6 CAR-T/CLDN6 CAR-T(A) infusion. Prior to LD chemotherapy initiation, infections, cardiac functions, glomerular filtration rate, and neurological assessments should be measured (refer to Section 6.1.1 of the Protocol). Only if the criteria apply, can the LD chemotherapy be initiated.
8) Refer to Section 6.5.2 of the Protocol for information about documenting optional chemotherapy.

9) The FFPE sample should be from the most recent tumor tissue obtained. If no archival tumor tissue is available, the patient must be biopsied for CLDN6 staining at pre-screening. In addition, a baseline pre-treatment tumor sample (archival or fresh) is required.
10) At the Pre-screening Visit, patients must be biopsied if the most recent FFPE sample obtained is not available anymore. During the course of the trial fresh tumor biopsies should be taken at the discretion of the investigator, if this does not pose a significant risk for the patient.
11) Ascites and pleural effusion samples should be taken during the course of the trial at the discretion of the investigator, if this does not pose a significant risk for the patient.
12) Tumor assessment will be performed during screening, at 6 weeks (±7 d) post infusion, then every 6 weeks (±7 d) for 50 weeks and every 12 weeks (±7 d) thereafter. Tumor response will be evaluated according to RECIST v1.1 and iRECIST.
13) Tumor assessment will be performed at d-5, if screening was more than 4 weeks ago and in case of bridging chemotherapy.
14) All AEs will be reported from signing of the main ICF until 90 d after last IMP treatment. Afterwards only related AEs will be reported. Prior to signing of main ICF only SAEs related to a trial procedure will be reported.
15) At visit d-5, blood samples will be collected prior to the start of LD chemotherapy.
16) In case the lab test of special interest blood draw is not performed on the same visit as blood draw for hematology and clinical chemistry, blood sample for hematology and clinical chemistry need to be collected in addition.
17) Pregnancy tests will be performed for women of childbearing potential only. CAR-T dosing will only occur with a repeated negative pregnancy test (performed 5 d prior to CLDN6 CAR-T/CLDN6 CAR-T(A) administration).
18) FSH and estradiol tests will be performed at screening if indicated in post-menopausal females.

19) At visit d-5, blood samples will be collected prior to LD chemotherapy administration.
20) Collection of tumor tissue before treatment start, either the most recent archival FFPE material or a fresh biopsy is mandatory for molecular profiling (e.g., NGS, RNAseq, multiplex immuno-profiling).

DocUUID : 6a803e66-f054-4d62-ad9b-00b24cc3317b

Table 4.4: Standard Part 2 SoA (CLDN6 CAR-T/CLDN6 CAR-T(A) + CLDN6 RNA-LPX)

Standard Part 2 SoA - treatment, primary follow-up

Phase	Treatment and primary follow-up																			LTFU[1]
Visit name	Infusion of CAR-T	CLDN6 RNA-LPX treatment phase																	Safety visit[3]	EoT & Primary FU
Trial period	d1	d2	d3	d4	d5 d6 d7	d8	d10	d17	d24 ±1d	d30-d35	d36, d43 ±1d	d51-d56	d57 ±1d	d72-d77	d78, d85, ±1d	d93-d98	d99 ±1d	d135, d177, d219, d261, d303, d345[2], d387, d429, d471, d513, d555, d597, d639, d681 ±7d		m25 ±14d
**Patient history**																				
Prior/ concomitant therapies[4]	X	X	X	X	X	X	X	X	X	X	X	X	X	X	X	X	X	(X)	X	(X)
Body weight	X									d30									X	X
Physical examination[5]	X	X	X	X	X	X	X	X	X	X	X	X	X	X	X	X	X	X	X	X
Vital signs[6]	X	X	X	X	X	X	X	X	X	X	X	X	X	X	X	X	X	X	X	X
ECG	(X)	(X)	(X)	(X)	(X)	(X)	(X)	(X)	(X)	(X)	(X)	(X)	(X)	(X)	(X)	(X)	(X)	(X)	(X)	(X)
ECOG performance status	X	X	X					X		d30	d36	d51	X	d72	d85	d93	X	X	X	X
Hospitalization status[7]	d1-d14																			
**Intervention**																				
CLDN6 CAR-T/CLDN6 CAR-T(A) infusion[8]	X																			
Vaccination of CLDN6 RNA-LPX[9]				d4						d30		d51		d72		d93		Vaccinations every 6 weeks (±7d) until m22		

DocUUID : 6a803e66-f054-4d62-ad9b-00b24cc3317b

**Phase**	**Treatment and primary follow-up**																			**LTFU [1]**
**Visit name**	**Infusion of CAR-T**	**CLDN6 RNA-LPX treatment phase**																	**Safety visit [3]**	**EoT & Primary FU**
**Trial period**	**d1**	**d2**	**d3**	**d4**	**d5 d6 d7**	**d8**	**d10**	**d17**	**d24 ±1d**	**d30-d35**	**d36, d43 ±1d**	**d51-d56**	**d57 ±1d**	**d72-d77**	**d78, d85, ±1d**	**d93-d98**	**d99 ±1d**	**d135, d177, d219, d261, d303, d345 [2], d387, d429, d471, d513, d555, d597, d639, d681 ±7d**		**m25 ±14d**
Documentation of antineoplastic therapy after CLDN6 infusion or trial discontinuation	X	X	X	X	X	X	X	X	X	X	X	X	X	X	X	X	X	X	X	X
**Disease assessments**																				
Fresh tumor biopsy [10]		As clinically indicated																		
Ascites/pleural effusion sample [11]		As clinically indicated																		
Tumor assessment [12]												Week 6 (±7d) post infusion, every 6 weeks (±7d) for 50 weeks, and every 12 weeks (±7d) thereafter								
**Safety**																				
Adverse events [13]	X	X	X	X	X	X	X	X	X	X	X	X	X	X	X	X	X	(X)	(X)	(X)
**Laboratory assessments**																				
Hematology (central lab) [14]	X (pre)			X (pre)		X	X	X	X	d30 (pre)	X	d51 (pre)	X	d72 (pre)	X	d93 (pre)	X	X	X	X
Clinical chemistry (central lab) [14]	X (pre)			X (pre)		X	X	X	X	d30 (pre)	X	d51 (pre)	X	d72 (pre)	X	d93 (pre)	X	X	X	X
Coagulation (central lab)	X (pre)																			X

DocUUID : 6a803e66-f054-4d62-ad9b-00b24cc3317b

Phase	Treatment and primary follow-up																			LTFU [1]
Visit name	Infusion of CAR-T	CLDN6 RNA-LPX treatment phase																	Safety visit [3]	EoT & Primary FU
Trial period	d1	d2	d3	d4	d5 d6 d7	d8	d10	d17	d24 ±1d	d30-d35	d36, d43 ±1d	d51-d56	d57 ±1d	d72-d77	d78, d85, ±1d	d93-d98	d99 ±1d	d135, d177, d219, d261, d303, d345 [2], d387, d429, d471, d513, d555, d597, d639, d681 ±7d		m25 ±14d
Endocrine tests (TSH, T3, and T4) (central lab)										d30 (pre)						d93 (pre)		X	X	X
Lab test of special interest for CRS/ICANS (IL-6, CRP, ferritin, fibrinogen, procalcitonin,) (central lab) [15]	X (pre+6h post)	X	X	X (pre)		X	X	X	X	d30 (pre)	X	d51 (pre)	X	d72 (pre)	X	d93 (pre)	X			
Serum pregnancy test (local lab) [16]													X				X	X	X	X
**Biomarker** [17]																				
Cytokines [17,18]	X pre	X	X	X (pre, 6h, 24h)		X	X	X	X	d30 (pre, 6h, 24h)	X	d51 (pre, 6h, 24h)	X	d72, (pre, 6h, 24h)	d78 (±1d)	d93, (pre, 6h, 24h)	X	d135 (pre, 6h, 24h), d141, then every 6/7 weeks (±7d) [19]		
qPCR (incl. hematology) [17,19]		X	X	X (pre)		X	X	X	X	d30 (pre)	X	d51 (pre)	X	d72 (pre)	d78 (±1d)	d93 (pre)	X	d135 (pre), d141, then every 6/7 weeks (±7d) [20]		
Flow-phenotype [17,20]							X	X	X											
CAR characterization [17,21]								X			d43 (±1d)				d85 (±1d)					

DocUUID : 6a803e66-f054-4d62-ad9b-00b24cc3317b

Phase	Treatment and primary follow-up																			LTFU [1]
Visit name	Infusion of CAR-T	CLDN6 RNA-LPX treatment phase																	Safety visit [3]	EoT & Primary FU
Trial period	d1	d2	d3	d4	d5 d6 d7	d8	d10	d17	d24 ±1d	d30-d35	d36, d43 ±1d	d51-d56	d57 ±1d	d72-d77	d78, d85, ±1d	d93-d98	d99 ±1d	d135, d177, d219, d261, d303, d345 [2], d387, d429, d471, d513, d555, d597, d639, d681 ±7d		m25 ±14d
Tumor biomarker [17,20]							X		X		d36 (±1d)	d51 (pre)		d72 (pre)		d93 (pre)		d135 (pre) (±7d)		
CAR immunogenicity assay [17,22]	X (pre)								X							d93 (pre)				
Tumor tissue (fresh) [23]								X												
Survival status	For all patients who receive a CLDN6 CAR-T/CLDN6 CAR-T(A) infusion, patients are enrolled into the long-term follow-up period for survival status as per schedule in Appendix 8 after the EoT Visit. If a patient misses a scheduled visit where survival status is required, survival status can be obtained via phone contact.																			

Abbreviations: AE, adverse event; CAR, chimeric antigen receptor; CAR-T, chimeric antigen receptor T cell; CLDN6, claudin 6; CRP, C-reactive protein; CRS, cytokine release syndrome; d, day; ECG, electrocardiogram; ECOG, Eastern Cooperative Oncology Group; EoT, End of Trial; FSH, follicle-stimulating hormone; FU, follow-up; h, hour; Hep B, hepatitis B virus; Hep C, hepatitis C virus; HIV, human immunodeficiency virus; ICANS, immune effector cell-associated neurotoxicity syndrome; ICF, informed consent form; IL 6, interleukin 6; INR, international normalized ratio; LDH, lactate dehydrogenase; (LT)FU, (long-term) follow-up; m, month; qPCR, quantitative polymerase chain reaction; RNA-LPX, liposomally formulated ribonucleic acid encoding the vaccine; T3, triiodothyronine; T4, thyroxine; TSH, thyroid-stimulating hormone; (X), as clinically indicated.

1) Refer to LTFU appendix for follow-up after trial completion.
2) If at m12/d345 vaccinations cease, visits switch from every 6 weeks to quarterly (m15, m18, m21, m24).
3) All patients (if alive) who discontinue trial treatment will be evaluated at a Safety/EoT Follow-up Visit at least 90 d and no more than 97 d after the last trial treatment administration. This visit will replace a regular visit if it is in the same visit window.
4) All therapies (including medications, non-drug therapies) being taken by the patient from screening until 90 d after last IMP treatment will be regarded as prior or concomitant therapies (newly started or ongoing during this time period). Afterwards only therapies corresponding to related AEs will be recorded.

5) A complete physical examination will be performed at screening and the EoT Visit. At other visits, an abbreviated examination will be performed and new or worsened clinically significant abnormalities will be recorded. Patients should be examined daily when hospitalized. On CLDN6 RNA-LPX vaccination days, patients should be examined prior to and 4-6 h following vaccination. All physical examinations should include the ICE score; the ICE score must be assessed daily within the hospitalization period.
6) Vital signs (systolic and diastolic blood pressure, heart rate, body temperature, and oxygen saturation) will be measured in a seated or recumbent position after at least 5 min rest (refer to Section 8.2.2 of the Protocol), and should be measured daily if the patient is hospitalized. On CLDN6 RNA-LPX vaccination days, vital signs should be checked prior to and 4-6 h following vaccination.
7) Hospitalization can be shortened to d7 at the discretion of the SRC in case the patient shows no signs of CRS/ICANS. Thereafter, after each administration of CLDN6 RNA-LPX, the patient will be hospitalized as needed, but at least until improvement of any adverse event to Grade 2 or lower. Prolonged hospitalization is allowed at the discretion of the investigator.
8) The CAR-T dose will be administered by i.v. infusion. At visit d1, assessments according to Section 6.1.2.2 of the Protocol should be made before CLDN6 CAR-T/CLDN6 CAR-T(A) administration. Only if the criteria apply, can the infusion of CLDN6 CAR-T/CLDN6 CAR-T(A) take place.
9) The vaccination will be administered as a slow i.v. bolus injection. Further vaccinations after d93 are given every 6 weeks until m22. Refer to Section 6.1.3 of the Protocol for further information.
10) At the Pre-screening Visit, patients must be biopsied if the most recent FFPE sample obtained is not available anymore. During the course of the trial fresh tumor biopsies should be taken at the discretion of the investigator, if this does not pose a significant risk for the patient.
11) Ascites and pleural effusion samples should be taken during the course of the trial at the discretion of the investigator, if this does not pose a significant risk for the patient.
12) Tumor assessment will be performed during screening, at 6 weeks (±7 d) post infusion, then every 6 weeks (±7 d) for 50 weeks and every 12 weeks (±7 d) thereafter. Tumor response will be evaluated according to RECIST v1.1 and iRECIST.
13) All AEs will be reported from signing of the main ICF until 90 d after last IMP treatment. Afterwards only related AEs will be reported. Prior to signing of main ICF only SAEs related to a trial procedure will be reported.
14) At visit d1, blood samples will be collected prior to infusion of CAR-T. On RNA-LPX vaccination visits, blood samples will be collected prior to vaccination (blood collection on a weekend only in case of a CRS/ICANS).
15) At visit d1 blood samples will be collected prior to infusion of CAR-T, and 6 h (±1 h) post infusion. If CRS/ICANS is suspected, the parameters should be assessed daily by the central laboratory for the whole duration of the CRS/ICANS. As soon as the CRS/ICANS is over, parameters will be analyzed at the visits indicated in the SoA again (refer to Section 6.6.4.1 of the Protocol).
16) Pregnancy tests will be performed for women of childbearing potential only. CAR-T dosing will only occur with a repeated negative pregnancy test (performed 5 d prior to CLDN6 CAR-T/CLDN6 CAR-T(A) administration).
17) After one year: The indicated biomarker assessments will only be performed if clinically indicated (after discussion with sponsor) – if CLDN6 CAR-T/CLDN6 CAR-T(A) are not detectable, biomarker assessments would cease with the exception of the qPCR assessment, and tumor biomarkers where relevant.
18) At visit d1, blood samples will be collected prior to infusion of CAR-T. On RNA-LPX administration visits, blood samples will be collected prior to and 6h and 24 h after administration of RNA-LPX. After d141, every 6 weeks (pre-, 6 h, 24 h, and 1 week after) RNA-LPX vaccination, if no RNA-LPX vaccination, then together with tumor assessment.
19) On RNA-LPX administration visits, blood samples will be collected prior to administration of RNA-LPX. A blood tube for hematology assessments must be taken with all samples for qPCR, if not already specified for this time point in the SoA. After d141, every 6 weeks (pre- and 1 week after) RNA-LPX vaccination, if no RNA-LPX vaccination, then together with tumor assessment.
20) On RNA-LPX administration visits, blood samples will be collected prior to administration of RNA-LPX.
21) For CAR characterization, the second and third time points should be aligned with the tumor assessments at 6 and 12 weeks. Up to three unscheduled additional blood sample collections are allowed if indicated by qPCR.
22) At visit d1, blood samples will be collected prior to CAR-T infusion.

DocUUID : 6a803e66-f054-4d62-ad9b-00b24cc3317b

23) An on treatment fresh tumor biopsy should be taken at the discretion of the investigator, if this does not pose a significant risk for the patient, ideally around d17 and at progression for molecular profiling and to determine CLDN6 expression and CAR-T infiltration into the tumor tissue.

DocUUID : 6a803e66-f054-4d62-ad9b-00b24cc3317b

Table 4.5: Optional Part 2 SoA (CLDN6 CAR-T/CLDN6 CAR-T(A) + CLDN6 RNA-LPX)

Optional Part 2 SoA - pre-screening, screening, apheresis, pre-treatment

Phase	Pre-screening for CLDN6 expression	Screening [1]	Apheresis		Pre-treatment		
Visit name			Leukapheresis	Post-leukapheresis FU	LD chemotherapy		
Trial period	Before screening	d-56 to d-18 [1]	d-18 (or earlier)	Within 3 d of leukapheresis	d-5	d-4	d-3
Informed consent [2]	X	X					
**Patient history**							
Inclusion/exclusion criteria		X					
Demography [3]	X	X					
Medical history		X					
Diagnosis and extent of cancer	X	X					
Prior antineoplastic therapies		X					
Prior/concomitant therapies [4]		X	X	X	X	X	X
Height		X					
Body weight		X					
Physical examination [5]		X	X	X	X	X	X
Vital signs [6]		X	X		X	X	X
ECG		X			(X)	(X)	(X)

DocUUID : 6a803e66-f054-4d62-ad9b-00b24cc3317b

Phase	Pre-screening for CLDN6 expression	Screening [1]	Apheresis		Pre-treatment		
Visit name			Leukapheresis	Post-leukapheresis FU	LD chemotherapy		
Trial period	Before screening	d-56 to d-18 [1]	d-18 (or earlier)	Within 3 d of leukapheresis	d-5	d-4	d-3
ECOG performance status		X	X				
Eligibility		X					
**Intervention**							
Leukapheresis screening (according to local standards)		X					
Leukapheresis			X				
LD chemotherapy [7]					X	X	X
Optional chemotherapy [8]		As clinically indicated					
**Disease assessments**							
Archival tumor tissue [9]	X						
Fresh biopsy [10]	(X)	As clinically indicated					
Ascites/pleural effusion sample [11]		As clinically indicated					
Tumor assessment [12]		X			X [13]		
**Safety**							
Adverse events [14]		X	X	X	X	X	X
**Laboratory assessments**							
Serology/virology (HIV		X					

DocUUID : 6a803e66-f054-4d62-ad9b-00b24cc3317b

Phase	Pre-screening for CLDN6 expression	Screening[1]	Apheresis		Pre-treatment		
Visit name			Leukapheresis	Post-leukapheresis FU	LD chemotherapy		
Trial period	Before screening	d-56 to d-18 [1]	d-18 (or earlier)	Within 3 d of leukapheresis	d-5	d-4	d-3
test, Hep B+C) (central lab)							
Hematology (central lab) [15]		X			X		
Clinical chemistry (central lab) [15]		X			X		
Coagulation (central lab)		X					
Endocrine tests (TSH, T3, and T4) (central lab)		X					
Lab test of special interest for CRS/ICANS (IL-6, CRP, ferritin, fibrinogen, procalcitonin) (central lab) [15,16]					X		
Urinalysis (local lab)		X					
Serum pregnancy test (local lab) [17]		X			X		
FSH and estradiol tests (central lab) [18]		X					

DocUUID : 6a803e66-f054-4d62-ad9b-00b24cc3317b

Phase	Pre-screening for CLDN6 expression	Screening[1]	Apheresis		Pre-treatment		
Visit name			Leukapheresis	Post-leukapheresis FU	LD chemotherapy		
Trial period	Before screening	d-56 to d-18[1]	d-18 (or earlier)	Within 3 d of leukapheresis	d-5	d-4	d-3
**Biomarker**							
Flow-phenotype[19]					X (pre)		
Tumor biomarker[19]					X (pre)		
Tumor tissue (molecular profiling)[20]		X					
HLA typing		X					

Abbreviations: AE, adverse event; CAR, chimeric antigen receptor; CAR-T, chimeric antigen receptor T cell; CLDN6, claudin 6; CRP, C-reactive protein; CRS, cytokine release syndrome; d, day; ECG, electrocardiogram; ECOG, Eastern Cooperative Oncology Group; EoT, End of Trial; FSH, follicle-stimulating hormone; FU, follow-up; h, hour; Hep B, hepatitis B virus; Hep C, hepatitis C virus; HIV, human immunodeficiency virus; ICANS, immune effector cell-associated neurotoxicity syndrome; ICF, informed consent form; IL 6, interleukin 6; INR, International normalized ratio; LD, lymphodepleting; LDH, lactate dehydrogenase; (LT)FU, (long-term) follow-up; m, month; qPCR, quantitative polymerase chain reaction; RNA-LPX, liposomally formulated ribonucleic acid encoding the vaccine; T3, triiodothyronine; T4, thyroxine; TSH, thyroid-stimulating hormone; (X), as clinically indicated.

1) All screening assessments must be completed prior to leukapheresis: completion of screening assessments within a 4-week window is preferred.
2) The patient will sign one pre-screening ICF for CLDN6 expression evaluation at the Pre-screening Visit and the main ICF at the Screening Visit.
3) Year of birth/age, sex, ethnicity, race.
4) All therapies (including medications, non-drug therapies) being taken by the patient from screening until 90 d after last IMP treatment will be regarded as prior or concomitant therapies (newly started or ongoing during this time period). Afterwards only therapies corresponding to related AEs will be recorded.
5) A complete physical examination will be performed at screening and the EoT Visit. At other visits, an abbreviated examination will be performed and new or worsened clinically significant abnormalities will be recorded. Patients should be examined daily when hospitalized. All physical examinations should include the ICE score assessment.
6) Vital signs (systolic and diastolic blood pressure, heart rate, body temperature, and oxygen saturation) will be measured in a seated or recumbent position after at least 5 min rest (refer to Section 8.2.2 of the Protocol), and should be checked daily when the patient is hospitalized.
7) LD chemotherapy should be completed at least three days prior to the CLDN6 CAR-T/CLDN6 CAR-T(A) infusion. Prior to LD chemotherapy initiation, infections, cardiac functions, glomerular filtration rate, and neurological assessments should be measured (refer to Section 6.1.1 of the Protocol). Only if the criteria apply, can the LD chemotherapy be initiated.
8) Refer to Section 6.5.2 of the Protocol for information about documenting optional chemotherapy.

9) The FFPE sample should be from the most recent tumor tissue obtained. If no archival tumor tissue is available, the patient must be biopsied for CLDN6 staining at pre-screening. In addition, a baseline pre-treatment tumor sample (archival or fresh) is required.
10) At the Pre-screening Visit, patients must be biopsied if the most recent FFPE sample obtained is not available anymore. During the course of the trial fresh tumor biopsies should be taken at the discretion of the investigator, if this does not pose a significant risk for the patient.
11) Ascites and pleural effusion samples should be taken during the course of the trial at the discretion of the investigator, if this does not pose a significant risk for the patient.
12) Tumor assessment will be performed during screening, at 6 weeks (±7 d) post infusion, then every 6 weeks (±7 d) for 50 weeks and every 12 weeks (±7 d) thereafter. Tumor response will be evaluated according to RECIST v1.1 and iRECIST.
13) Tumor assessment will be performed at d-5, if screening was more than 4 weeks ago and in case of bridging chemotherapy.
14) All AEs will be reported from signing of the main ICF until 90 d after last IMP treatment. Afterwards only related AEs will be reported. Prior to signing of main ICF only SAEs related to a trial procedure will be reported.
15) At visit d-5, blood samples will be collected prior to the start of LD chemotherapy.
16) In case the lab test of special interest blood draw is not performed on the same visit as blood draw for hematology and clinical chemistry, blood sample for hematology and clinical chemistry need to be collected in addition.
17) Pregnancy tests will be performed for women of childbearing potential only. CAR-T dosing will only occur with a repeated negative pregnancy test (performed 5 d prior to CLDN6 CAR-T/CLDN6 CAR-T(A) administration).
18) FSH and estradiol tests will be performed at screening if indicated in post-menopausal females.

19) At visit d-5, blood samples will be collected prior to LD chemotherapy administration.
20) Collection of tumor tissue before treatment start, either the most recent archival FFPE material or a fresh biopsy is mandatory for molecular profiling (e.g., NGS, RNAseq, multiplex immuno-profiling).

DocUUID : 6a803e66-f054-4d62-ad9b-00b24cc3317b

Table 4.6: Optional Part 2 SoA (CLDN6 CAR-T/CLDN6 CAR-T(A) + CLDN6 RNA-LPX)

Optional Part 2 SoA - treatment, primary follow-up

Phase	Treatment and primary follow-up																			LTFU [1]
**Visit name**	**Infusion of CAR-T**	**CLDN6 RNA-LPX treatment phase**																	**Safety visit [3]**	**EoT & Primary FU**
**Trial period**	**d1**	**d2**	**d3**	**d4**	**d5 d6 d7**	**d8**	**d10**	**d17**	**d24 ±1d**	**d30-d35**	**d36, d43 ±1d**	**d51-d56**	**d57 ±1d**	**d72-d77**	**d78, d85, ±1d**	**d93-d98**	**d99 ±1d**	**d135, d177, d219, d261, d303, d345 [2], d387, d429, d471, d513, d555, d597, d639, d681 ±7d**		**m25 ±14d**
**Patient history**																				
Prior/ concomitant therapies [4]	X	X	X	X	X	X	X	X	X	X	X	X	X	X	X	X	X	(X)	X	(X)
Body weight	X									d30									X	X
Physical examination [5]	X	X	X	X	X	X	X	X	X	X	X	X	X	X	X	X	X	X	X	X
Vital signs [6]	X	X	X	X	X	X	X	X	X	X	X	X	X	X	X	X	X	X	X	X
ECG	(X)	(X)	(X)	(X)	(X)	(X)	(X)	(X)	(X)	(X)	(X)	(X)	(X)	(X)	(X)	(X)	(X)	(X)	(X)	(X)
ECOG performance status	X	X	X					X		d30	d36	d51	X	d72	d85 (±1d)	d93	X	X	X	X
Hospitalization status [7]	d1-d14																			
**Intervention**																				
CLDN6 CAR-T/CLDN6 CAR-T(A) infusion [8]	X																			

DocUUID : 6a803e66-f054-4d62-ad9b-00b24cc3317b

**Phase**	**Treatment and primary follow-up**																			**LTFU** [1]
**Visit name**	**Infusion of CAR-T**	**CLDN6 RNA-LPX treatment phase**																	**Safety visit** [3]	**EoT & Primary FU**
**Trial period**	**d1**	**d2**	**d3**	**d4**	**d5 d6 d7**	**d8**	**d10**	**d17**	**d24 ±1d**	**d30-d35**	**d36, d43 ±1d**	**d51-d56**	**d57 ±1d**	**d72-d77**	**d78, d85, ±1d**	**d93-d98**	**d99 ±1d**	**d135, d177, d219, d261, d303, d345** [2]**, d387, d429, d471, d513, d555, d597, d639, d681 ±7d**		**m25 ±14d**
Vaccination of CLDN6 RNA-LPX [9]									X		d36	d51		d72		d93		Vaccinations every 6 weeks (±7d) until m22		
Documentation of antineoplastic therapy after CLDN6 infusion or trial discontinuation	X	X	X	X	X	X	X	X	X	X	X	X	X	X	X	X	X	X	X	X
**Disease assessments**																				
Fresh tumor biopsy [10]		As clinically indicated																		
Ascites/pleural effusion sample [11]		As clinically indicated																		
Tumor assessment [12]												Week 6 (±7d) post infusion, every 6 weeks (±7d) for 50 weeks, and every 12 weeks (±7d) thereafter								
**Safety**																				
Adverse events [13]	X	X	X	X	X	X	X	X	X	X	X	X	X	X	X	X	X	(X)	(X)	(X)
**Laboratory assessments**																				
Hematology (central lab) [14]	X (pre)			X		X	X	X	X (pre)	d30	d36 (pre), d43 (±1d)	d51 (pre)	X	d72 (pre)	X	d93 (pre)	X	X	X	X

DocUUID : 6a803e66-f054-4d62-ad9b-00b24cc3317b

**Phase**	**Treatment and primary follow-up**																			**LTFU** [1]
**Visit name**	**Infusion of CAR-T**	**CLDN6 RNA-LPX treatment phase**																	**Safety visit** [3]	**EoT & Primary FU**
**Trial period**	**d1**	**d2**	**d3**	**d4**	**d5 d6 d7**	**d8**	**d10**	**d17**	**d24 ±1d**	**d30-d35**	**d36, d43 ±1d**	**d51-d56**	**d57 ±1d**	**d72-d77**	**d78, d85, ±1d**	**d93-d98**	**d99 ±1d**	**d135, d177, d219, d261, d303, d345** [2]**, d387, d429, d471, d513, d555, d597, d639, d681 ±7d**		**m25 ±14d**
Clinical chemistry (central lab) [14]	X (pre)			X		X	X	X	X (pre)	d30	d36 (pre), d43 (±1d)	d51 (pre)	X	d72 (pre)	X	d93 (pre)	X	X	X	X
Coagulation (central lab)	X (pre)																			X
Endocrine tests (TSH, T3, and T4) (central lab)										d30						d93 (pre)		X	X	X
Lab test of special interest for CRS/ICANS (IL-6, CRP, ferritin, fibrinogen, procalcitonin,) (central lab) [15]	X (pre+6h post)	X	X	X		X	X	X	X (pre)	d30	d36 (pre), d43 (±1d)	d51 (pre)	X	d72 (pre)	X	d93 (pre)	X			
Serum pregnancy test (local lab) [16]													X				X	X	X	X
**Biomarker** [17]																				
Cytokines [17,18]	X pre	X	X	X		X	X	X	X (pre, 6h, 24h)	d30	d36 (pre), d43 (±1d)	d51 (pre, 6h, 24h)	X	d72, (pre, 6h, 24h)	d78 (±1d)	d93, (pre, 6h, 24h)	X	d135 (pre, 6h, 24h), d141, then every 6/7 weeks (±7d) [19]		

DocUUID : 6a803e66-f054-4d62-ad9b-00b24cc3317b

Phase	Treatment and primary follow-up																			LTFU [1]
**Visit name**	**Infusion of CAR-T**	**CLDN6 RNA-LPX treatment phase**																	**Safety visit [3]**	**EoT & Primary FU**
**Trial period**	**d1**	**d2**	**d3**	**d4**	**d5 d6 d7**	**d8**	**d10**	**d17**	**d24 ±1d**	**d30-d35**	**d36, d43 ±1d**	**d51-d56**	**d57 ±1d**	**d72-d77**	**d78, d85, ±1d**	**d93-d98**	**d99 ±1d**	**d135, d177, d219, d261, d303, d345 [2], d387, d429, d471, d513, d555, d597, d639, d681 ±7d**		**m25 ±14d**
qPCR (incl. hematology) [17,19]		X	X	X		X	X	X	X (pre)	d30	d36 (pre), d43 (±1d)	d51 (pre)	X	d72 (pre)	d78 (±1d)	d93 (pre)	X	d135 (pre), d141, then every 6/7 weeks (±7d) [20]		
Flow-phenotype [17,20]							X	X	X (pre)											
CAR characterization [17,21]								X			d43 (±1d)				d85 (±1d)					
Tumor biomarker [17,20]							X		X (pre)		d36 (pre)	d51 (pre)		d72 (pre)		d93 (pre)		d135 (pre) (±7d)		
CAR immunogenicity assay [17,22]	X (pre)								X (pre)							d93 (pre)				
Tumor tissue (fresh) [23]								X												
**Survival status**	**For all patients who receive a CLDN6 CAR-T/CLDN6 CAR-T(A) infusion, patients are enrolled into the long-term follow-up period for survival status as per schedule in Appendix 8 after the EoT Visit. If a patient misses a scheduled visit where survival status is required, survival status can be obtained via phone contact.**																			

Abbreviations: AE, adverse event; CAR, chimeric antigen receptor; CAR-T, chimeric antigen receptor T cell; CLDN6, claudin 6; CRP, C-reactive protein; CRS, cytokine release syndrome; d, day; ECG, electrocardiogram; ECOG, Eastern Cooperative Oncology Group; EoT, End of Trial; FSH, follicle-stimulating hormone; FU, follow-up; h, hour; Hep B, hepatitis B virus; Hep C, hepatitis C virus; HIV, human immunodeficiency virus; ICANS, immune effector cell-associated neurotoxicity syndrome; ICF, informed consent form; IL 6, interleukin 6; INR, international normalized ratio; LDH, lactate dehydrogenase; (LT)FU, (long-term) follow-up; m, month; qPCR, quantitative polymerase chain reaction; RNA-LPX, liposomally formulated ribonucleic acid encoding the vaccine; T3, triiodothyronine; T4, thyroxine; TSH, thyroid-stimulating hormone; (X), as clinically indicated.

DocUUID : 6a803e66-f054-4d62-ad9b-00b24cc3317b

1) Refer to LTFU appendix for follow-up after trial completion.
2) If at m12/d345 vaccinations cease, visits switch from every 6 weeks to quarterly (m15, m18, m21, m24).
3) All patients (if alive) who discontinue trial treatment will be evaluated at a Safety/EoT Follow-up Visit at least 90 d and no more than 97 d after the last trial treatment administration. This visit will replace a regular visit if it is in the same visit window.
4) All therapies (including medications, non-drug therapies) being taken by the patient from screening until 90 d after last IMP treatment will be regarded as prior or concomitant therapies (newly started or ongoing during this time period). Afterwards only therapies corresponding to related AEs will be recorded.
5) A complete physical examination will be performed at screening and the EoT Visit. At other visits, an abbreviated examination will be performed and new or worsened clinically significant abnormalities will be recorded. Patients should be examined daily when hospitalized. On CLDN6 RNA-LPX vaccination days, patients should be examined prior to and 4-6 h following vaccination. All physical examinations should include the ICE score; the ICE score must be assessed daily within the hospitalization period.
6) Vital signs (systolic and diastolic blood pressure, heart rate, body temperature, and oxygen saturation) will be measured in a seated or recumbent position after at least 5 min rest (refer to Section 8.2.2 of the Protocol), and should be measured daily if the patient is hospitalized. On CLDN6 RNA-LPX vaccination days, vital signs should be checked prior to and 4-6 h following vaccination.
7) Hospitalization can be shortened to d7 at the discretion of the SRC in case the patient shows no signs of CRS/ICANS. Thereafter, after each administration of CLDN6 RNA-LPX, the patient will be hospitalized as needed, but at least until improvement of any adverse event to Grade 2 or lower. Prolonged hospitalization is allowed at the discretion of the investigator.
8) The CAR-T dose will be administered by i.v. infusion. At visit d1, assessments according to Section 6.1.2.2 of the Protocol should be made before CLDN6 CAR-T/CLDN6 CAR-T(A) administration. Only if the criteria apply, can the infusion of CLDN6 CAR-T/CLDN6 CAR-T(A) take place.
9) The vaccination will be administered as a slow i.v. bolus injection. Further vaccinations after d93 are given every 6 weeks until m22. Refer to Section 6.1.3 of the Protocol for further information.
10) At the Pre-screening Visit, patients must be biopsied if the most recent FFPE sample obtained is not available anymore. During the course of the trial fresh tumor biopsies should be taken at the discretion of the investigator, if this does not pose a significant risk for the patient.
11) Ascites and pleural effusion samples should be taken during the course of the trial at the discretion of the investigator, if this does not pose a significant risk for the patient.
12) Tumor assessment will be performed during screening, at 6 weeks (±7 d) post infusion, then every 6 weeks (±7 d) for 50 weeks and every 12 weeks (±7 d) thereafter. Tumor response will be evaluated according to RECIST v1.1 and iRECIST.
13) All AEs will be reported from signing of the main ICF until 90 d after last IMP treatment. Afterwards only related AEs will be reported. Prior to signing of main ICF only SAEs related to a trial procedure will be reported.
14) At visit d1, blood samples will be collected prior to infusion of CAR-T. On RNA-LPX vaccination visits, blood samples will be collected prior to vaccination (blood collection on a weekend only in case of a CRS/ICANS).
15) At visit d1 blood samples will be collected prior to infusion of CAR-T, and 6 h (±1 h) post infusion. If CRS/ICANS is suspected, the parameters should be assessed daily by the central laboratory for the whole duration of the CRS/ICANS. As soon as the CRS/ICANS is over, parameters will be analyzed at the visits indicated in the SoA again (refer to Section 6.6.4.1 of the Protocol).
16) Pregnancy tests will be performed for women of childbearing potential only. CAR-T dosing will only occur with a repeated negative pregnancy test (performed 5 d prior to CLDN6 CAR-T/CLDN6 CAR-T(A) administration).
17) After one year: The indicated biomarker assessments will only be performed if clinically indicated (after discussion with sponsor) – if CLDN6 CAR-T/CLDN6 CAR-T(A) are not detectable, biomarker assessments would cease with the exception of the qPCR assessment, and tumor biomarkers where relevant.
18) At visit d1, blood samples will be collected prior to infusion of CAR-T. On RNA-LPX administration visits, blood samples will be collected prior to and 6h and 24 h after administration of RNA-LPX. After d141, every 6 weeks (pre-, 6 h, 24 h, and 1 week after) RNA-LPX vaccination, if no RNA-LPX vaccination, then together with tumor assessment.

DocUUID : 6a803e66-f054-4d62-ad9b-00b24cc3317b

19) On RNA-LPX administration visits, blood samples will be collected prior to administration of RNA-LPX. A blood tube for hematology assessments must be taken with all samples for qPCR, if not already specified for this time point in the SoA. After d141, every 6 weeks (pre- and 1 week after) RNA-LPX vaccination, if no RNA-LPX vaccination, then together with tumor assessment.
20) On RNA-LPX administration visits, blood samples will be collected prior to administration of RNA-LPX.
21) For CAR characterization, the second and third time points should be aligned with the tumor assessments at 6 and 12 weeks. Up to three unscheduled additional blood sample collections are allowed if indicated by qPCR.
22) At visit d1, blood samples will be collected prior to CAR-T infusion.
23) An on treatment fresh tumor biopsy should be taken at the discretion of the investigator, if this does not pose a significant risk for the patient, ideally around d17 and at progression for molecular profiling and to determine CLDN6 expression and CAR-T infiltration into the tumor tissue.

Table 4.7: Optional Part 2 SoA (CLDN6 CAR-T/CLDN6 CAR-T(A) + CLDN6 RNA-LPX without LD chemotherapy)

Optional Part 2 SoA - pre-screening, screening, apheresis, pre-treatment (without LD chemotherapy)

**Phase**	**Pre-screening for CLDN6 expression**	**Screening [1]**	**Apheresis**	
**Visit name**			**Leukapheresis**	**Post-leukapheresis FU**
**Trial period**	**Before screening**	**d-56 to d-18 [1]**	**d-18 (or earlier)**	**Within 3 d of leukapheresis**
Informed consent [2]	X	X		
**Patient history**				
Inclusion/exclusion criteria		X		
Demography [3]	X	X		
Medical history		X		
Diagnosis and extent of cancer	X	X		
Prior antineoplastic therapies		X		
Prior/concomitant therapies [4]		X	X	X
Height		X		
Body weight		X		
Physical examination [5]		X	X	X
Vital signs [6]		X	X	
ECG		X		
ECOG performance status		X	X	
Eligibility		X		
**Intervention**				
Leukapheresis screening (according to local standards)		X		

DocUUID : 6a803e66-f054-4d62-ad9b-00b24cc3317b

Phase	Pre-screening for CLDN6 expression	Screening [1]	Apheresis	
Visit name			Leukapheresis	Post-leukapheresis FU
Trial period	Before screening	d-56 to d-18 [1]	d-18 (or earlier)	Within 3 d of leukapheresis
Leukapheresis			X	
Optional chemotherapy [7]		As clinically indicated		
**Disease assessments**				
Archival tumor tissue [8]	X			
Fresh biopsy [9]	(X)	As clinically indicated		
Ascites/pleural effusion sample [10]		As clinically indicated		
Tumor assessment [11]		X		
**Safety**				
Adverse events [12]		X	X	X
**Laboratory assessments**				
Serology/virology (HIV test, Hep B+C) (central lab)		X		
Hematology (central lab)		X		
Clinical chemistry (central lab)		X		
Coagulation (central lab)		X		
Endocrine tests (TSH, T3, and T4) (central lab)		X		
Urinalysis (local lab)		X		
Serum pregnancy test (local lab) [14]		X		
FSH and estradiol tests (central lab) [15]		X		

DocUUID : 6a803e66-f054-4d62-ad9b-00b24cc3317b

Phase	Pre-screening for CLDN6 expression	Screening[1]	Apheresis	
Visit name			Leukapheresis	Post-leukapheresis FU
Trial period	Before screening	d-56 to d-18[1]	d-18 (or earlier)	Within 3 d of leukapheresis
**Biomarker**				
Tumor tissue (molecular profiling)[16]		X		
HLA typing		X		

Abbreviations: AE, adverse event; CAR, chimeric antigen receptor; CAR-T, chimeric antigen receptor T cell; CLDN6, claudin 6; CRP, C-reactive protein; CRS, cytokine release syndrome; d, day; ECG, electrocardiogram; ECOG, Eastern Cooperative Oncology Group; EoT, End of Trial; FSH, follicle-stimulating hormone; FU, follow-up; h, hour; Hep B, hepatitis B virus; Hep C, hepatitis C virus; HIV, human immunodeficiency virus; ICANS, immune effector cell-associated neurotoxicity syndrome; ICF, informed consent form; IL 6, interleukin 6; INR, international normalized ratio; LD, lymphodepleting; LDH, lactate dehydrogenase; (LT)FU, (long-term) follow-up ; m, month; qPCR, quantitative polymerase chain reaction; RNA-LPX, liposomally formulated ribonucleic acid encoding the vaccine; T3, triiodothyronine; T4, thyroxine; TSH, thyroid-stimulating hormone; (X), as clinically indicated.

1) All screening assessments must be completed prior to leukapheresis: completion of screening assessments within a 4-week window is preferred.
2) The patient will sign one pre-screening ICF for CLDN6 expression evaluation at the Pre-screening Visit and the main ICF at the Screening Visit.
3) Year of birth/age, sex, ethnicity, race.
4) All therapies (including medications, non-drug therapies) being taken by the patient from screening until 90 d after last IMP treatment will be regarded as prior or concomitant therapies (newly started or ongoing during this time period). Afterwards only therapies corresponding to related AEs will be recorded.
5) A complete physical examination will be performed at screening and the EoT Visit. At other visits, an abbreviated examination will be performed and new or worsened clinically significant abnormalities will be recorded. Patients should be examined daily when hospitalized. All physical examinations should include the ICE score.
6) Vital signs (systolic and diastolic blood pressure, heart rate, body temperature, and oxygen saturation) will be measured in a seated or recumbent position after at least 5 min rest (refer to Section 8.2.2 of the Protocol), and should be measured daily if the patient is hospitalized.
7) Refer to Section 6.5.2 of the Protocol for information about documenting optional chemotherapy.
8) The FFPE sample should be from the most recent tumor tissue obtained. If no tumor tissue is available, the patient must be biopsied for CLDN6 staining at pre-screening. In addition, a baseline pre-treatment tumor sample (archival or fresh) is required.
9) At the Pre-screening Visit, patients must be biopsied if the most recent FFPE sample obtained is not available anymore. During the course of the trial fresh tumor biopsies should be taken at the discretion of the investigator, if this does not pose a significant risk for the patient.
10) Ascites and pleural effusion samples should be taken during the course of the trial at the discretion of the investigator, if this does not pose a significant risk for the patient.
11) Tumor assessment will be performed at d-5, if screening was more than 4 weeks ago and in case of bridging chemotherapy.
12) All AEs will be reported from signing of the main ICF until 90 d after last IMP treatment. Afterwards only related AEs will be reported. Prior to signing of main ICF only SAEs related to a trial procedure will be reported.

DocUUID : 6a803e66-f054-4d62-ad9b-00b24cc3317b

13) In case of CRS/ICANS, blood sample for hematology and clinical chemistry also need to be collected and analyzed.
14) Pregnancy tests will be performed for women of childbearing potential only. CAR-T dosing will only occur with a repeated negative pregnancy test (performed 5 d prior to CLDN6 CAR-T/CLDN6 CAR-T(A) administration).
15) FSH and estradiol tests will be performed at screening if indicated in post-menopausal females.
16) Collection of tumor tissue before treatment start, either the most recent archival FFPE material or a fresh biopsy is mandatory for molecular profiling (e.g., NGS, RNAseq, multiplex immuno-profiling).

DocUUID : 6a803e66-f054-4d62-ad9b-00b24cc3317b

Table 4.8: Optional Part 2 SoA (CLDN6 CAR-T/CLDN6 CAR-T(A) + CLDN6 RNA-LPX without LD chemotherapy)

Optional Part 2 SoA - treatment, primary follow-up (without LD chemotherapy)

Phase	Treatment and primary follow-up																			LTFU [1]
**Visit name**	**Infusion of CAR-T**	**CLDN6 RNA-LPX treatment phase**																	**Safety Visit [3]**	**EoT & Primary FU**
**Trial period**	**d1**	**d2**	**d3**	**d4**	**d5 d6 d7**	**d8**	**d10**	**d17**	**d24 ±1d**	**d30-d35**	**d36, d43 ±1d**	**d51-d56**	**d57 ±1d**	**d72-d77**	**d78, d85, ±1d**	**d93-d98**	**d99 ±1d**	**d135, d177, d219, d261, d303, d345 [2], d387, d429, d471, d513, d555, d597, d639, d681 ±7d**		**m25 ±14d**
**Patient history**																				
Prior/ concomitant therapies [4]	X	X	X	X	X	X	X	X	X	X	X	X	X	X	X	X	X	(X)	X	(X)
Body weight	X									d30									X	X
Physical examination [5]	X	X	X	X	X	X	X	X	X	X	X	X	X	X	X	X	X	X	X	X
Vital signs [6]	X	X	X	X	X	X	X	X	X	X	X	X	X	X	X	X	X	X	X	X
ECG	(X)	(X)	(X)	(X)	(X)	(X)	(X)	(X)	(X)	(X)	(X)	(X)	(X)	(X)	(X)	(X)	(X)	(X)	(X)	(X)
ECOG performance status	X	X	X					X		d30	d36 (±1d)	d51	X	d72	d85 (±1d)	d93	X	X	X	X
Hospitalization status [7]	d1-d14																			
**Intervention**																				
CLDN6 CAR-T/CLDN6 CAR-T(A) infusion [8]	X																			

DocUUID : 6a803e66-f054-4d62-ad9b-00b24cc3317b

**Phase**	**Treatment and primary follow-up**																			**LTFU [1]**
**Visit name**	**Infusion of CAR-T**	**CLDN6 RNA-LPX treatment phase**																	**Safety Visit [3]**	**EoT & Primary FU**
**Trial period**	**d1**	**d2**	**d3**	**d4**	**d5 d6 d7**	**d8**	**d10**	**d17**	**d24 ±1d**	**d30-d35**	**d36, d43 ±1d**	**d51-d56**	**d57 ±1d**	**d72-d77**	**d78, d85, ±1d**	**d93-d98**	**d99 ±1d**	**d135, d177, d219, d261, d303, d345 [2], d387, d429, d471, d513, d555, d597, d639, d681 ±7d**		**m25 ±14d**
Vaccination of CLDN6 RNA-LPX [9]				d4						d30		d51		d72		d93		Vaccinations every 6 weeks (±7d) until m22		
Documentation of antineoplastic therapy after CLDN6 infusion or trial discontinuation	X	X	X	X	X	X	X	X	X	X	X	X	X	X	X	X	X	X	X	X
**Disease assessments**																				
Fresh tumor biopsy [10]		As clinically indicated																		
Ascites/pleural effusion sample [11]		As clinically indicated																		
Tumor assessment [12]	X [13]											W6 (±7d) post infusion, every 6 weeks (±7d) for 50 weeks, and every 12 weeks (±7d) thereafter								
**Safety**																				
Adverse events [14]	X	X	X	X	X	X	X	X	X	X	X	X	X	X	X	X	X	(X)	(X)	(X)
**Laboratory assessments**																				
Hematology (central lab) [15]	X (pre)	X	X	X (pre)	X	X	X	X	X	d30 (pre)	X	d51 (pre)	X	d72 (pre)	X	d93 (pre)	X	X	X	X
Clinical chemistry (central lab) [15]	X (pre)	X	X	X (pre)	X	X	X	X	X	d30 (pre)	X	d51 (pre),	X	d72, (pre)	X	d93 (pre)	X	X	X	X

DocUUID : 6a803e66-f054-4d62-ad9b-00b24cc3317b

Phase	Treatment and primary follow-up																		LTFU [1]
Visit name	Infusion of CAR-T	CLDN6 RNA-LPX treatment phase																Safety Visit [3]	EoT & Primary FU
Trial period	d1	d2	d3	d4	d5 d6 d7	d8	d10	d17	d24 ±1d	d30-d35	d36, d43 ±1d	d51-d56	d57 ±1d	d72-d77	d78, d85, ±1d	d93-d98	d99 ±1d	d135, d177, d219, d261, d303, d345 [2], d387, d429, d471, d513, d555, d597, d639, d681 ±7d		m25 ±14d
Coagulation (central lab)	X (pre)																			X
Endocrine tests (TSH, T3, and T4) (central lab)										d30 (pre)						d93 (pre)		X	X	X
Lab test of special interest for CRS/ICANS (IL-6, CRP, ferritin, fibrinogen, procalcitonin) (central lab) [16]	X (pre, 6h)	X	X	X (pre)	X	X	X	X	X	d30 (pre)	X	d51 (pre)	X	d72 (pre)	X	d93 (pre)	X			
Serum pregnancy test (local lab) [17]													X				X	X	X	X
**Biomarker [18]**																				
Cytokines [18,19]	X (pre)	X	X	X (pre, 6h, 24h)		X	X	X	X	d30 (pre, 6h, 24h)	d36 (±1d)	d51 (pre, 6h, 24h)	X	d72 (pre, 6h, 24h)	d78 (±1d)	d93 (pre, 6h, 24h)	X	d135 (pre, 6h, 24h), d141 (±7d)		
qPCR (incl. hematology) [18,20]		X	X	X (pre)		X	X	X	X	d30 (pre)	X	d51 (pre)	X	d72 (pre)	d78 (±1d)	d93 (pre)	X	d135 (pre), d141 (±7d)		
Flow-phenotype [18,21]	X (pre)						X	X	X											
CAR characterization [18,22]								X			d43 (±1d)				d85 (±1d)					

DocUUID : 6a803e66-f054-4d62-ad9b-00b24cc3317b

Phase	Treatment and primary follow-up																			LTFU [1]
**Visit name**	**Infusion of CAR-T**	**CLDN6 RNA-LPX treatment phase**																	**Safety Visit [3]**	**EoT & Primary FU**
**Trial period**	**d1**	**d2**	**d3**	**d4**	**d5 d6 d7**	**d8**	**d10**	**d17**	**d24 ±1d**	**d30-d35**	**d36, d43 ±1d**	**d51-d56**	**d57 ±1d**	**d72-d77**	**d78, d85, ±1d**	**d93-d98**	**d99 ±1d**	**d135, d177, d219, d261, d303, d345 [2], d387, d429, d471, d513, d555, d597, d639, d681 ±7d**		**m25 ±14d**
Tumor biomarker [18,21]	X (pre)						X		X	d30 (pre)		d51 (pre)		d72 (pre)		d93 (pre)		d135 (pre)		
CAR immunogenicity assay [18,23]	X (pre)								X								X			
Tumor tissue (fresh) [24]		As clinically indicated																		
**Survival status**	**For all patients who receive a CLDN6 CAR-T/CLDN6 CAR-T(A) infusion, patients are enrolled into the long-term follow-up period for survival status as per schedule in Appendix 8 after the EoT Visit. If a patient misses a scheduled visit where survival status is required, survival status can be obtained via phone contact.**																			

Abbreviations: AE, adverse event; CAR, chimeric antigen receptor; CAR-T, chimeric antigen receptor T cell; CLDN6, claudin 6; CRP, C-reactive protein; CRS, cytokine release syndrome; d, day; ECG, electrocardiogram; ECOG, Eastern Cooperative Oncology Group; EoT, End of Trial; FSH, follicle-stimulating hormone; FU, follow-up; h, hour; Hep B, hepatitis B virus; Hep C, hepatitis C virus; HIV, human immunodeficiency virus; ICANS, immune effector cell-associated neurotoxicity syndrome; ICF, informed consent form; IL 6, interleukin 6; INR, international normalized ratio; LDH, lactate dehydrogenase; (LT)FU, (long-term) follow-up; m, month; qPCR, quantitative polymerase chain reaction; RNA-LPX, liposomally formulated ribonucleic acid encoding the vaccine; T3, triiodothyronine; T4, thyroxine; TSH, thyroid-stimulating hormone; (X), as clinically indicated.

1) Refer to LTFU appendix for follow-up after trial completion.
2) If at m12/d345 vaccinations cease, visits switch from every 6 weeks to quarterly (m15, m18, m21, m24).
3) All patients (if alive) who discontinue trial treatment will be evaluated at a Safety/EoT Follow-up Visit at least 90 d and no more than 97 d after the last trial treatment administration. This visit will replace a regular visit if it is in the same visit window.
4) All therapies (including medications, non-drug therapies) being taken by the patient from screening until 90 d after last IMP treatment will be regarded as prior or concomitant therapies (newly started or ongoing during this time period). Afterwards only therapies corresponding to related AEs will be recorded.

5) A complete physical examination will be performed at screening and the EoT Visit. At other visits, an abbreviated examination will be performed and new or worsened clinically significant abnormalities will be recorded. Patients should be examined daily when hospitalized. On CLDN6 RNA-LPX vaccination days, patients should be examined prior to and 4-6 h following vaccination. All physical examinations should include the ICE score; the ICE score must be assessed daily within the hospitalization period.
6) Vital signs (systolic and diastolic blood pressure, heart rate, body temperature, and oxygen saturation) will be measured in a seated or recumbent position after at least 5 min rest (refer to Section 8.2.2 of the Protocol), and should be measured daily if the patient is hospitalized. On CLDN6 RNA-LPX vaccination days, vital signs should be checked prior to and 4-6 h, following vaccination.
7) Hospitalization can be shortened to d7 at the discretion of the SRC in case the patient shows no signs of CRS/ICANS. Thereafter, after each administration of CLDN6 RNA-LPX, the patient will be hospitalized as needed, but at least until improvement of any adverse event to Grade 2 or lower. Prolonged hospitalization is allowed at the discretion of the investigator.
8) The CAR-T dose will be administered by i.v. infusion. At visit d1, assessments according to Section 6.1.2.2 of the Protocol should be made before CLDN6 CAR-T/CLDN6 CAR-T(A) administration. Only if the criteria apply, can the infusion of CLDN6 CAR-T/CLDN6 CAR-T(A) take place.
9) The vaccination will be administered as a slow i.v. bolus injection. Further vaccinations after d93 are given every 6 weeks until m22. Refer to Section 6.1.3 of the Protocol for further information.
10) At the Pre-screening Visit, patients must be biopsied if the most recent FFPE sample obtained is not available anymore. During the course of the trial fresh tumor biopsies should be taken at the discretion of the investigator, if this does not pose a significant risk for the patient.
11) Ascites and pleural effusion samples should be taken during the course of the trial at the discretion of the investigator, if this does not pose a significant risk for the patient.
12) Tumor assessment will be performed during screening, at 6 weeks (±7 d) post infusion, then every 6 weeks (±7 d) for 50 weeks and every 12 weeks (±7 d) thereafter. Tumor response will be evaluated according to RECIST v1.1 and iRECIST.
13) Tumor assessment will be performed prior to or at d1, if screening was more than 4 weeks ago and in case of bridging chemotherapy.
14) All AEs will be reported from signing of the main ICF until 90 d after last IMP treatment. Afterwards only related AEs will be reported. Prior to signing of main ICF only SAEs related to a trial procedure will be reported.
15) At visit d1, blood samples will be collected prior to infusion of CAR-T. On RNA-LPX vaccination visits, blood samples will be collected prior to vaccination (blood collection on a weekend only in case of a CRS/ICANS).
16) At visit d1 blood samples will be collected prior to infusion of CAR-T, and 6 h (±1 h) post infusion. On RNA-LPX vaccination visits, blood samples will be collected prior to vaccination. If CRS/ICANS is suspected, the parameters should be assessed daily by the central laboratory for the whole duration of the CRS/ICANS. As soon as the CRS/ICANS is over, parameters will be analyzed at the visits indicated in the SoA again (refer to Section 6.6.4.1 of the Protocol).
17) Pregnancy tests will be performed for women of childbearing potential only. CAR-T dosing will only occur with a repeated negative pregnancy test (performed 5 d prior to CLDN6 CAR-T/CLDN6 CAR-T(A) administration).
18) After one year: The indicated biomarker assessments will only be performed if clinically indicated (after discussion with sponsor) – if CLDN6 CAR-T/CLDN6 CAR-T(A) are not detectable, biomarker assessments would cease with the exception of the qPCR assessment, and tumor biomarkers where relevant.
19) At visit d1, blood samples will be collected prior to infusion of CAR-T. On RNA-LPX administration visits, blood samples will be collected prior to, and 6h, and 24 h after administration of RNA-LPX. After d141, every 6 weeks (pre- and 6 h, 24 h, and 1 week) after) RNA-LPX vaccination, if no RNA-LPX vaccination, then together with tumor assessment.
20) On RNA-LPX administration visits, blood samples will be collected prior to administration of RNA-LPX. A blood tube for hematology assessments must be taken with all samples for qPCR, if not already specified for this time point in the SoA. After d141, every 6 weeks (pre-, and 1 week) after) RNA-LPX vaccination, if no RNA-LPX vaccination, then together with tumor assessment.
21) At visit d1, blood samples will be collected prior to infusion of CAR-T.
22) For CAR characterization, the second and third time points should be aligned with the tumor assessments at 6 and 12 weeks. Up to three unscheduled additional blood sample collections are allowed if indicated by qPCR.

DocUUID : 6a803e66-f054-4d62-ad9b-00b24cc3317b

23) At visit d1, blood samples will be collected prior to infusion of CAR-T.
24) An on treatment fresh tumor biopsy should be taken at the discretion of the investigator, if this does not pose a significant risk for the patient, ideally around d17 and at progression for molecular profiling and to determine CLDN6 expression and CAR-T infiltration into the tumor tissue.

DocUUID : 6a803e66-f054-4d62-ad9b-00b24cc3317b

10.5 Appendix 5: Censoring Rules

Table 5.1 Censoring Rules for PFS & DOR Based on Food & Drug Administration (FDA) Guidelines

Situation	Date Patient Has Event or is Censored[1]	Situation Outcome
Inadequate baseline assessment (not applicable for DOR)	Start Date (C1 D1)	Censored
Inadequate post-baseline assessment (not applicable for DOR)	Start Date (C1 D1)	Censored
Progression documented on or between scheduled tumor assessments	Date of first objective tumor assessment showing objective progression	Progressed (Event)
Progression documented not after 2 or more consecutive missing scheduled assessments<br>(*see text below table for calculation of* 2 or more consecutive missing scheduled assessments *)*	Date of PD for RECIST 1.1/ for iRECIST the date of first iUPD associated with confirmed progression.	Progressed (Event)

DocUUID : 6a803e66-f054-4d62-ad9b-00b24cc3317b

**Situation**	**Date Patient Has Event or is Censored[1]**	**Situation Outcome**
Death not after 2 or more consecutive missing scheduled assessments<br>(*see text below table for calculation of* 2 or more consecutive missing scheduled assessments*)*	Date of death	Death (Event)
Death without objective progression	Date of death	Death (Event)
Death or progression after 2 or more consecutive missing scheduled assessments<br>(*see text below table for calculation of* 2 or more consecutive missing scheduled assessments*)*	Date of last adequate assessment with evidence of no progression; if no adequate assessment exists then censored at date of first dose	Censored
New anticancer therapy started prior to progression or death	Date of last adequate assessment with evidence of no progression before anticancer therapy; if no adequate assessment exists then censored at date of first dose	Censored
No progression	Date of last adequate assessment with evidence of no progression; if no adequate assessment exists then censored at date of first dose	Censored
Lost to follow-up	censor at day of last tumor assessment	Censored

DocUUID : 6a803e66-f054-4d62-ad9b-00b24cc3317b

1: For date of censoring, if a tumor assessment takes place over a number of days (ie, superficial lesions one day, scans another), the last date is used as the assessment date.

<u>Calculation for determining if 2 or more consecutive tumour assessments have been missed.</u>

- Subject has 2 consecutive missed response assessments = subject has more than 2*6*7+7+7 days between consecutive response assessments prior to Week 50 or 2*12*7+7+7 after Week 50

Note: in 6*7+7+7: 6*7 is number of days between response assessments before Week 50 as per schedule 7+7 because the visit window is ±7 days.

DocUUID : 6a803e66-f054-4d62-ad9b-00b24cc3317b

Table 5.2 Event and Censoring Rules for OS Based on Food & Drug Administration (FDA) Guidelines

Situation	Date Patient Has Event or is Censored	Situation Outcome
Death	Date of death	Event
Alive	Date last known to be alive (date of last contact collected in the CRF). *Check this date vs the latest date for a patient, if discrepant then raise for resolution.*	Censored

Table 5.3 Event and Censoring Rules for TTF Based on Food & Drug Administration (FDA) Guidelines

Situation	Date Patient Has Event or is Censored	Situation Outcome
Treatment (CLDN6 RNALPX) discontinuation due to any reason including disease progression, treatment toxicity, add-on of new anti-cancer therapy, and death	Date of disease progression/ Date at which the treatment toxicity leading to treatment discontinuation is noticed/ Start date of new anti-cancer therapy/ Date of death.	Event
Completed the treatment	Date of end of treatment	Censored

DocUUID : 6a803e66-f054-4d62-ad9b-00b24cc3317b

10.6 Appendix 6: Dose escalation table

Number of evaluable patients with DLT at a given dose level after the first treatment cycle	Escalation decision rule
0 out of 3<br>OR<br>1 out of 6	Enter patients at the next higher dose level
1 out of 3	Enter more patients at this dose level to a total of at least 6 evaluable patients or 2 patients with DLT
2 out of 3<br>OR<br>at least 2 out of 6	Dose escalation will be stopped, the MTD will be considered to be reached at the one before the last dose level explored. The sponsor will decide, based on a discussion between the investigators and the sponsor at a dose escalation meeting, if additional patients need to be enrolled and at which dose to finalize the trial
Any condition which would require further clarification of safety	A cohort of 6 patients can eventually be extended and more patients can be enrolled in a cohort if decided by the sponsor based on a discussion between the investigators and the sponsor at a dose escalation meeting

DLT = dose-limiting toxicity; MTD = maximum tolerated dose.

DocUUID : 6a803e66-f054-4d62-ad9b-00b24cc3317b

10.7 Rules for assigning date of Overall response from Tumor assessment page

A date is not collected for the Overall assessment page

		Each Evaluation #		
Overall Timepoint Response	*Overall Timepoint Response date*	Target Lesion Date	Non-Target Lesion Date	New Lesion Date
PD	min(dt1, dt2, dt3)	dt1*	dt2*	dt3*
CR	max(dt1, dt2)	dt1	dt2	-
PR	dt1	dt1	any	-
SD (Not a CR, PR or PD)	min(dt1, dt2)	dt1	dt2	-
NA, NE	min(dt1, dt2, dt3)	dt1	dt2	dt3

** indicates a PD response by individual Target or Non-Target or appearance of New Lesion, otherwise corresponding date can be excluded while picking the earliest scan date.*

DocUUID : 6a803e66-f054-4d62-ad9b-00b24cc3317b
