# Supplementary material for: CLDN6-specific CAR-T cells plus amplifying RNA vaccine in relapsed or refractory solid tumors: the phase 1 BNT211-01 trial
Source: Nat Med. 2023 Oct 23;29(11):2844–53. doi: 10.1038/s41591-023-02612-0 (PMC10667102; doi:10.1038/s41591-023-02612-0)
Supplement: Supplementary file 2 — Reporting Summary [file 41591_2023_2612_MOESM2_ESM.pdf]

nature portfolio

Corresponding author(s): Ugur Sahin

Last updated by author(s): Aug 7, 2023

Reporting Summary

Nature Portfolio wishes to improve the reproducibility of the work that we publish. This form provides structure for consistency and transparency in reporting. For further information on Nature Portfolio policies, see our Editorial Policies and the Editorial Policy Checklist.

Statistics

For all statistical analyses, confirm that the following items are present in the figure legend, table legend, main text, or Methods section.

n/a	Confirmed	
☐	☒	The exact sample size (*n*) for each experimental group/condition, given as a discrete number and unit of measurement
☒	☐	A statement on whether measurements were taken from distinct samples or whether the same sample was measured repeatedly
☐	☒	The statistical test(s) used AND whether they are one- or two-sided<br>*Only common tests should be described solely by name; describe more complex techniques in the Methods section.*
☒	☐	A description of all covariates tested
☒	☐	A description of any assumptions or corrections, such as tests of normality and adjustment for multiple comparisons
☐	☒	A full description of the statistical parameters including central tendency (e.g. means) or other basic estimates (e.g. regression coefficient) AND variation (e.g. standard deviation) or associated estimates of uncertainty (e.g. confidence intervals)
☐	☒	For null hypothesis testing, the test statistic (e.g. *F*, *t*, *r*) with confidence intervals, effect sizes, degrees of freedom and *P* value noted<br>*Give P values as exact values whenever suitable.*
☒	☐	For Bayesian analysis, information on the choice of priors and Markov chain Monte Carlo settings
☒	☐	For hierarchical and complex designs, identification of the appropriate level for tests and full reporting of outcomes
☐	☒	Estimates of effect sizes (e.g. Cohen's *d*, Pearson's *r*), indicating how they were calculated

Our web collection on statistics for biologists contains articles on many of the points above.

Software and code

Policy information about availability of computer code

Data collection	No code was used
Data analysis	Data was analyzed and depicted using R programing (version 4.1.2) and GraphPad Prism 9

For manuscripts utilizing custom algorithms or software that are central to the research but not yet described in published literature, software must be made available to editors and reviewers. We strongly encourage code deposition in a community repository (e.g. GitHub). See the Nature Portfolio guidelines for submitting code & software for further information.

Data

Policy information about availability of data

All manuscripts must include a data availability statement. This statement should provide the following information, where applicable:

- Accession codes, unique identifiers, or web links for publicly available datasets
- A description of any restrictions on data availability
- For clinical datasets or third party data, please ensure that the statement adheres to our policy

This trial is currently ongoing. Upon completion of this clinical trial, summary-level results will be made public and shared in line with clinical data-sharing guidelines. Requests for access to aggregated data (the tables produced in line with the statistical analysis plan) will be reviewed and approved by the Safety Review Committee on the basis of scientific merit. The datasets generated and/or analyzed during the current study are not publicly available due to proprietary considerations beyond the data that was made available here. All data provided are anonymized to respect the privacy of patients who have participated in the trial, in line with applicable

laws and regulations. Data requests pertaining to the manuscript may be made to the corresponding author (U.S.; ugur.sahin@biontech.de). Requests will be processed within 16 weeks. The CAR-T (WO2020/161186) and CARVac (W2016/180778) technology is patented.

Research involving human participants, their data, or biological material

Policy information about studies with human participants or human data. See also policy information about sex, gender (identity/presentation), and sexual orientation and race, ethnicity and racism.

Reporting on sex and gender	Sex was ascertained at screening by the investigator. Given the study size, outcomes were not analyzed according to sex.
Reporting on race, ethnicity, or other socially relevant groupings	N/A
Population characteristics	Information on age, ECOG performance status, cancer type, and treatment history were collected at screening.
Recruitment	Participants were recruited by the investigators/clinical trial centers. To participate in this study and before any non-routine baseline or screening evaluation, the investigators at each study site ensured that each patient was fully informed of the study and had signed a written informed consent. Patients signed an informed consent form for the CLDN6 pre-screening, and again for enrollment to the trial (leukapheresis and treatment). Eligibility criteria are detailed in the manuscript. No bias emerging from recruitment is expected. Patients were not compensated for their participation in the study.
Ethics oversight	The study protocol was approved by institutional review boards of the authorities in Germany (Paul-Ehrlich-Institute) and the Netherlands (Central Committee on Research involving Human Subjects).

Note that full information on the approval of the study protocol must also be provided in the manuscript.

Field-specific reporting

Please select the one below that is the best fit for your research. If you are not sure, read the appropriate sections before making your selection.

☒ Life sciences ☐ Behavioural & social sciences ☐ Ecological, evolutionary & environmental sciences

For a reference copy of the document with all sections, see nature.com/documents/nr-reporting-summary-flat.pdf

Life sciences study design

All studies must disclose on these points even when the disclosure is negative.

Sample size	Sample size was determined according to the 3+3 dose escalation approach
Data exclusions	In accordance with the statistical analysis plan, one patient who received CAR-T cells at a dose of <DL1 was excluded from the efficacy set. 2 patients with tumor collection dates not available were excluded from a correlation analysis of the intensity of CLDN6 expression by IHC staining in tumor samples versus the age of the sample.
Replication	As this was a clinical study, analyses were performed on single (longitudinal) samples
Randomization	This was a phase 1 study that recruited according to the principles of a 3+3 dose escalation study.
Blinding	N/A, this was an open-label, non-blinded study.

Reporting for specific materials, systems and methods

We require information from authors about some types of materials, experimental systems and methods used in many studies. Here, indicate whether each material, system or method listed is relevant to your study. If you are not sure if a list item applies to your research, read the appropriate section before selecting a response.

Materials & experimental systems

n/a	Involved in the study
☒	☐ Antibodies
☒	☐ Eukaryotic cell lines
☒	☐ Palaeontology and archaeology
☒	☐ Animals and other organisms
☐	☒ Clinical data
☒	☐ Dual use research of concern
☒	☐ Plants

Methods

n/a	Involved in the study
☒	☐ ChIP-seq
☒	☐ Flow cytometry
☒	☐ MRI-based neuroimaging

Clinical data

Policy information about clinical studies

All manuscripts should comply with the ICMJE guidelines for publication of clinical research and a completed CONSORT checklist must be included with all submissions.

Clinical trial registration

NCT04503278

Study protocol

The study protocol was supplied with the manuscript

Data collection

The first patient was enrolled (to pre-screening) on September 16, 2020 and this interim analysis was conducted with a data cut-off date of 06 October 2022. Data were recorded by on-site clinical research associates in the EDC system, based on medical files and clinical examinations performed by qualified site personnel. A clinical research associate from the CRO ICON performed Source Data Verification. Safety laboratory analyses were performed in a central laboratory and integrated back in to the EDC system.

Outcomes

Primary endpoints:

• To assess the safety and tolerability of CLDN6 CAR-T +/- CARVac: Occurrence of treatment-emergent adverse events (TEAEs) within a patient including ≥ Grade 3, serious, fatal TEAEs by relationship
• To identify the maximum tolerated dose (MTD)/RP2D for each IMP: MTD is defined as the highest tolerated dose of CLDN6 CAR-T +/- CARVac where less than 33% of the patients experience a DLT. The RP2D of CLDN6 CART +/- CARVac is based on integrated evaluation of safety and other data for all dose levels tested.

Secondary endpoints:

• To describe the profile of soluble immune factors in CLDN6 CAR-T +/- CARVac
• To evaluate anti-tumor activity of CLDN6 CAR-T +/- CARVac according to response evaluation criteria in solid tumors version 1.1 (RECIST 1.1): Objective response rate (ORR) defined as the proportion of patients in whom a complete response (CR) or partial response (PR) (per RECIST 1.1) is observed as best overall response. Disease control rate (DCR) is defined as the proportion of patients in whom a CR or PR or stable disease (SD) (per RECIST 1.1, SD assessed at least 6 weeks after the first dose) is observed as best overall response. Duration of response (DOR) defined as the time from first objective response (CR or PR per RECIST 1.1) to first occurrence of objective tumor progression (PD per RECIST 1.1)/recurrence, or death from any cause, whichever occurs first.

Exploratory:
• To evaluate efficacy of CLDN6 CART +/- CLDN6 RNALPX according to:
o Progression-free survival (PFS) defined as the time from first dose of CLDN6 CAR-T to first objective PD per RECIST 1.1, or death from any cause, whichever occurs first
o PFS defined as the time from first dose of CLDN6 CAR-T to first objective iCPD, or death from any cause, whichever occurs first
o OS defined as the time from first dose of CLDN6 CAR-T/CLDN6 CAR-T to death from any cause
o Time to treatment failure (TTF) in patients treated with CLDN6 RNALPX, defined as time from first injection to discontinuation of treatment for any reason including disease progression, treatment toxicity, add-on of new anti-cancer therapy, and death
• Preliminary assessment of biomarkers that might act as pharmacodynamic, antitumor, and safety indicators of activity of CLDN6 CAR-T +/- CARVac: Detection, phenotypic characterization and functionality of CLDN6 CAR-T detected by flow cytometry in blood (and ascites, pleural effusion, if available)
• To characterize the in vivo cellular pharmacokinetics (PK) profile (levels, persistence, trafficking) of CLDN6 CART in target tissues (blood and other tissues if available)
• Presence of CLDN6 CAR-T in blood and other tissues, if available
o Maximum concentration (Cmax), time to maximum concentration (Tmax), area under the time-concentration curve (AUC) and other relevant PK parameters of CLDN6 CART in blood
• Additionally for patients crossing over from Part 1 to additional cohort in Part 1 (CLDN6 CAR-T + CARVac): reexpansion of CLDN6 CAR-T in blood and other tissues

Exploratory endpoints not reported in this manuscript:

o To evaluate anti-tumor activity of CLDN6 CAR-T +/- CARVAC according to immune response evaluation criteria in solid tumors (iRECIST) (not reported in this manuscript)

To assess the safety and tolerability of
CLDN6 CAR-T/CLDN6 CAR-T(A) +/-

CLDN6 RNA-LPX
